# Exploring the Performance of ChatGPT-4 in the Taiwan Audiologist Qualification Examination: Preliminary Observational Study Highlighting the Potential of AI Chatbots in Hearing Care

**DOI:** 10.2196/55595

**Published:** 2024-04-26

**Authors:** Shangqiguo Wang, Changgeng Mo, Yuan Chen, Xiaolu Dai, Huiyi Wang, Xiaoli Shen

**Affiliations:** 1 Human Communication, Learning, and Development Unit, Faculty of Education, The University of Hong Kong, Hong Kong, China (Hong Kong); 2 Department of Otorhinolaryngology, Head and Neck Surgery, Faculty of Medicine, The Chinese University of Hong Kong, Hong Kong, China (Hong Kong); 3 Department of Special Education and Counselling, The Education University of Hong Kong, Hong Kong, China (Hong Kong); 4 Department of Social Work, Hong Kong Baptist University, Hong Kong, China (Hong Kong); 5 Department of Medical Services, Children’s Hospital, Zhejiang University School of Medicine, Hangzhou, China; 6 Department of Health and Early Childhood Care, Ningbo College of Health School, Ningbo, China

**Keywords:** ChatGPT, medical education, artificial intelligence, AI, audiology, hearing care, natural language processing, large language model, Taiwan, hearing, hearing specialist, audiologist, examination, information accuracy, educational technology, healthcare services, chatbot, health care services

## Abstract

**Background:**

Artificial intelligence (AI) chatbots, such as ChatGPT-4, have shown immense potential for application across various aspects of medicine, including medical education, clinical practice, and research.

**Objective:**

This study aimed to evaluate the performance of ChatGPT-4 in the 2023 Taiwan Audiologist Qualification Examination, thereby preliminarily exploring the potential utility of AI chatbots in the fields of audiology and hearing care services.

**Methods:**

ChatGPT-4 was tasked to provide answers and reasoning for the 2023 Taiwan Audiologist Qualification Examination. The examination encompassed six subjects: (1) basic auditory science, (2) behavioral audiology, (3) electrophysiological audiology, (4) principles and practice of hearing devices, (5) health and rehabilitation of the auditory and balance systems, and (6) auditory and speech communication disorders (including professional ethics). Each subject included 50 multiple-choice questions, with the exception of behavioral audiology, which had 49 questions, amounting to a total of 299 questions.

**Results:**

The correct answer rates across the 6 subjects were as follows: 88% for basic auditory science, 63% for behavioral audiology, 58% for electrophysiological audiology, 72% for principles and practice of hearing devices, 80% for health and rehabilitation of the auditory and balance systems, and 86% for auditory and speech communication disorders (including professional ethics). The overall accuracy rate for the 299 questions was 75%, which surpasses the examination’s passing criteria of an average 60% accuracy rate across all subjects. A comprehensive review of ChatGPT-4’s responses indicated that incorrect answers were predominantly due to information errors.

**Conclusions:**

ChatGPT-4 demonstrated a robust performance in the Taiwan Audiologist Qualification Examination, showcasing effective logical reasoning skills. Our results suggest that with enhanced information accuracy, ChatGPT-4’s performance could be further improved. This study indicates significant potential for the application of AI chatbots in audiology and hearing care services.

## Introduction

In recent years, the rapid advancement of large language models (LLMs) has significantly expanded their usage in various domains. Among the leading artificial intelligence (AI) chatbots—such as Bard, Bing, and ChatGPT—there has been a notable increase in diverse applications in everyday life. Prominently, ChatGPT, launched by OpenAI in November 2022 [1], stands out in the realm of AI chatbots. This model, known for its proficiency in generating and comprehending human-like text, showcases remarkable natural language processing skills. It has the capability to grasp complex queries, furnish insightful responses, and participate in meaningful conversations, thus broadening the scope of AI’s practicality in everyday scenarios [2,3].

ChatGPT represents a significant advancement in the field of natural language processing, exemplifying the latest developments in LLMs, particularly within the subset of autoregressive language models. Such generative LLMs, including ChatGPT, are predominantly trained on extensive text corpora. They use the decoder element of a transformer model, a groundbreaking architecture introduced by Vaswani et al [4] in 2017. This model is adept at predicting subsequent tokens in text sequences, a capability that has been progressively refined in subsequent research [5,6]. The transformer model, upon which ChatGPT is built, has revolutionized natural language processing. Its core strength lies in its ability to process text sequences efficiently, facilitating tasks such as language translation, question answering, and text summarization. One of the key features of this architecture is the self-attention mechanism, which allows it to understand long-range dependencies between words in a sentence without the need for sequential processing. This feature not only enhances efficiency compared to older recurrent neural network architectures but also offers improved interpretability, linking the semantic and syntactic structures of language inputs more effectively [4]. In addition to these capabilities, ChatGPT has evolved to incorporate real-time and knowledge-based information through various plug-ins. The introduction of GPT-4 by OpenAI in 2023 has expanded ChatGPT’s proficiency to include processing both image and text inputs [1], marking a new milestone in the versatility and applicability of AI in diverse contexts.

ChatGPT has received considerable attention and exploration in its application within health care. The integration of ChatGPT into health care demonstrates its significant potential in enhancing patient education and handling general inquiries, marking it as a vital informational and supportive tool [7]. The broad applicability of AI chatbots in health care extends beyond patient interaction, serving clinicians, researchers, and students, with ChatGPT showing effectiveness in personalizing patient interactions and providing consumer health education [8,9]. This trend aligns with the overarching aim in health care AI to increase accessibility to medical knowledge and make care more affordable. Chatbots offer continuous health advice and support, potentially improving patient outcomes by reducing the need for in-person consultations. Additionally, they provide health care professionals with valuable insights for more informed patient care decision-making, though concerns regarding data transparency have been noted [10]. ChatGPT is capable of generating empathetic, high-quality responses to health-related queries, often comparable to those of physicians, and shows promise in producing emotionally aware responses with potential for continuous improvement [11,12]. In low- and middle-income countries, ChatGPT has great potential as a pivotal tool in public health efforts. Its advantages span various domains such as health literacy, screening, triage, remote support, mental health, multilingual communication, medical training, and professional support, addressing numerous challenges in these health care systems [13]. Furthermore, ChatGPT’s role as a supplementary educational tool in areas requiring aptitude, problem-solving, critical thinking, and reading comprehension has been highlighted. The ChatGPT-4 version, in particular, shows potential in applications such as discharge summarization and group learning, enhancing human-computer interaction through verbal fluency [14,15]. However, the need for embracing these advancements while ensuring patient safety and recognizing the limitations of AI in intricate clinical cases is emphasized [16].

The evolution of computational sciences in hearing care services and research has given rise to the field of computational audiology. This approach combines algorithms, machine learning, and data-driven modeling for audiological diagnosis, treatment, and rehabilitation, using biological, clinical, and behavioral theories to augment care for patients and professionals [17]. The rapid development of AI technologies, especially LLMs such as ChatGPT, has significantly contributed to this field’s growth. ChatGPT’s advanced capabilities position it as a potential tool for patient interaction, education, aural rehabilitation program, and preliminary diagnostics in audiology [18,19]. However, it is crucial to recognize its current limitations. While it can handle complex interactions, it is not a substitute for human expertise in specialized areas such as audiology and is limited in interpreting nuanced medical information or performing physical diagnostics. AI chatbots have shown immense potential in hearing health care, aiding patients, clinicians, and researchers. Their applications range from initial screenings, educational support, and teleaudiology services for patients, to data analysis and decision support for clinicians and researchers [19]. In countries with vast geographical areas and imbalanced hearing care resources, AI chatbots could significantly enhance the development of hearing care services. Very recently, explorations into the use of AI chatbots for answering questions pertaining audiological knowledge have shown that AI chatbots can serve as a tool to access basic audiological information [20]. However, the accuracy and reliability of information provided by these tools remain a concern [19].

Despite the significant potential of AI chatbots to enhance hearing care services, research in this area remains sparse. AI chatbots’ ability to understand questions and provide logical responses based on available information is crucial. This capability suggests promising applications in hearing care, including educational support, patient assistance in clinical settings, and aid for clinical staff. By engaging with AI chatbots, students, teachers, patients, and clinical personnel could significantly improve learning outcomes, patient care, and clinical practice efficiency. Therefore, this study starts from the most fundamental aspects to explore the performance of the current commercial version of ChatGPT-4 in taking an audiologist qualification examination (ie, the Taiwan Audiologist Qualification Examination). This investigation not only assesses the accuracy of responses to test questions but also explores the ability of the current AI chatbot to comprehend and logically respond to examination questions. These capabilities form the cornerstone for future integration of AI chatbots into educational support or clinical service assistance.

## Methods

### Materials

This study used the 2023 Taiwan Audiologist Qualification Examination [21]—a professional licensing examination for audiologists in Taiwan. Candidates of this examination are required to have a bachelor’s or masters’s degree in audiology and at least 6 months or 375 hours of clinical practice. The examination comprises six subjects: (1) basic auditory science, (2) behavioral audiology, (3) electrophysiological audiology, (4) principles and practice of hearing devices, (5) health and rehabilitation of the auditory and balance systems, and (6) auditory and speech communication disorders (including professional ethics). Each subject consists of 50 multiple-choice questions, except for behavioral audiology, which has 49 questions, totaling 299 questions in all. The examination papers featured 7 images, pivotal for answering 13 of the questions. Notably, these images were embedded directly within the PDF version of the examination rather than being provided as separate attachments. However, it is important to highlight that the images’ resolution was relatively low, and they were presented without color. When extracted and saved in JPEG format, the images ranged in size from 12.7 to 27.2 KB and had resolutions spanning from 82 to 150 DPI. All related PDFs are accessible for download from the official source [21].

### Prompt Engineering

**Figure 1.**
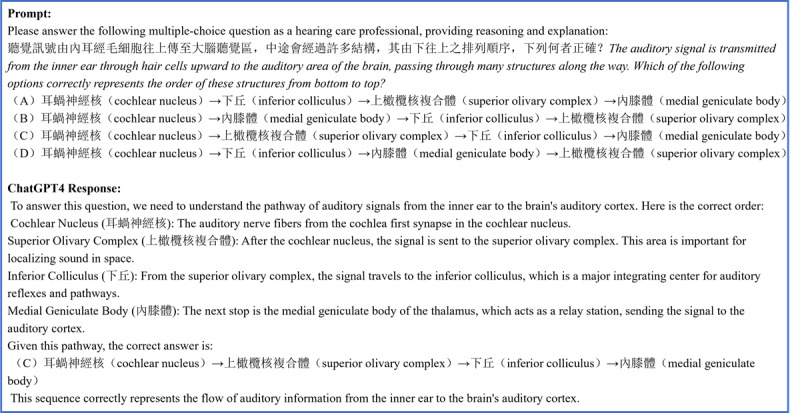
A template of a question posed to ChatGPT-4 and its corresponding responses. The example provided is the first question from the basic auditory science examination. The italicized English translations in the question stem are solely for reader comprehension and are not included in the actual prompt.

Recognizing the significant influence of prompt engineering (where “prompt” refers to the input text provided by the user, which the model responds to) on the outputs of generative LLMs, a standardized prompt format was used in this research: “Please answer the following multiple-choice question as a hearing care professional, providing reasoning and explanation.” This format was chosen to assess the logic and reasoning behind ChatGPT’s responses. The original examination questions, a blend of Traditional Chinese and English, often used bilingual terminology for specialized concepts. For this study, ChatGPT was instructed to respond in English. Additionally, ChatGPT was not informed that these questions were from the Taiwan Audiologist Qualification Examination to prevent it from sourcing specific information to increase the accuracy of its responses. An example of a prompt and response is illustrated in Figure 1.

### Procedure

All questions and correct answers were downloaded from the official website of the Taiwan Ministry of Examination in PDF format [21]. Subsequently, all questions were pre-edited in a Word (Microsoft Corp) document to avoid formatting issues. ChatGPT-4, enhanced with DALL-E (Decoder-Only Autoregressive Language and Image Synthesis), browsing, and analysis capabilities, was used for the test from December 10 to 12, 2023. A separate chat was used for each subject of the examination. Despite being within the same subject, the questions essentially had no overlapping content. For questions presented as images, both the image and the text format of the question were provided to leverage ChatGPT-4’s image recognition capabilities. It is important to note that the resolution of the images supplied in the test was relatively poor, which could have potentially impacted the accuracy of image recognition. Following the approach of Gilson et al [9], the reasons for errors in incorrectly answered questions were categorized as follows: (1) logical errors: the response correctly identifies relevant information but fails to translate this information into an appropriate answer; (2) information errors: ChatGPT either overlooks a key piece of information, whether present in the question stem or from external sources, or shows a lack of expected knowledge; and (3) statistical errors: the error is due to a miscalculation, including explicit arithmetic errors or incorrect estimations of statistical data. Authors SW and CM, both having a PhD in audiology, reviewed the original questions in Chinese and the GPT’s responses in English, and then compared ChatGPT-4’s responses to the official correct answers provided for the examination (all multiple-choice questions) to determine whether each question was answered correctly. They then performed a cross-check to ensure the accuracy of this step. Subsequently, SW and CM classified the incorrect answers into the 3 aforementioned categories and compared their classification results. In case of any discrepancies, they consulted with HW, who has a master’s degree in public health, to reach a consensus and make a final decision together.

### Ethical Considerations

This research did not involve human participants or private data and was therefore exempt from ethics approval by the ethics committee of Ningbo College of Health Sciences.

### Data Analysis

The data analysis for this study was straightforward and conducted using Excel (Microsoft Corp). Our primary objective was to calculate the accuracy rate of ChatGPT-4 when tasked with taking the Taiwan Audiologist Qualification Examination.

## Results

### Overall Performance

ChatGPT-4 demonstrated commendable performance in the Taiwan Audiologist Qualification Examination. The accuracy rates for the 6 subjects were as follows: 88% for basic auditory science, 63% for behavioral audiology, 58% for electrophysiological audiology, 72% for principles and practice of hearing devices, 80% for health and rehabilitation of the auditory and balance systems, and 86% for auditory and speech communication disorders (including professional ethics). The overall accuracy rate for the 299 questions was 75% (see Table 1). The examination’s passing criteria include an average accuracy rate of 60% across all subjects. Thus, ChatGPT-4 successfully passed this examination. Records of all ChatGPT-4's responses to the test questions can be found in the supplements (MultimediaAppendices 1^6^). A detailed review of ChatGPT 4’s responses revealed that errors were not caused by logical or statistical errors; instead, all incorrect answers resulted from information errors.

**Table 1.** Performance of ChatGPT-4 in the 2023 Taiwan Audiologist Qualification Examination.

	Questions, n	Correct responses, n	Accuracy rate, %
Basic auditory science	50	44	88
Behavioral audiology	49	31	63
Electrophysiological audiology	50	29	58
Principles and practice of hearing devices	50	36	72
Health and rehabilitation of the auditory and balance systems	50	40	80
Auditory and speech communication disorders (including professional ethics)	50	43	86
Total questions	299	233	75
Questions with images	13	8	62
Images	7	4	57

### Information Errors Leading to Incorrect Answers

#### Lack of Correct Information Sources

Most incorrect answers were due to ChatGPT-4 relying on inaccurate information. For example, in the basic auditory science examination, question 43 involved identifying an incorrect statement about temporal masking among options A, B, C, and D. Option B stated that forward masking occurs when a signal appears after the masking noise, even with a 200-millisecond gap between the 2 stimuli (which is a misconception). The correct answer was that this statement is false, but ChatGPT-4’s explanation suggested otherwise:

Forward Masking with 200 ms Gap (B): This is correct. Forward masking can occur when the signal appears after the masking noise, even with a gap as long as 200 ms.

This response indicates that ChatGPT-4 did not have accurate information to answer this question. As a matter of fact, no masking occurs when the time interval between the masker and the signal equals or exceeds 200 milliseconds [22]. Similarly, for option D, which stated that forward masking is more effective than backward masking, ChatGPT-4’s choice and explanation were as follows:

Forward Masking More Effective Than Backward Masking (D): This is typically incorrect. Backward masking is often found to be more effective than forward masking.

While this appears correct at first glance, it lacks the clarification that backward masking is more effective than forward masking when the same time interval exists between the masker and signal, with more masking occurring when the masker follows the signal [23]. However, from the perspective of the information available to ChatGPT-4, option D was the most reasonable choice.

#### Conflicting Information Sources

Multiple sources of information also led to incorrect responses from ChatGPT-4. For example, question 22 in the electrophysiological audiology examination asked about the most appropriate frequency for tympanometry in newborns, with the correct answer being 1000 Hz. Despite its simplicity and lack of complexity in reasoning, ChatGPT-4 provided an incorrect response:

(A) 112 Hz is typically used for infants and young children. Newborns and young children have a more compliant ear canal than adults, and lower frequencies such as 226 Hz can sometimes overestimate middle ear pressure and compliance. Therefore, a lower frequency probe tone is often recommended.

Considering the straightforward nature of this question, the authors expected an accurate answer from ChatGPT-4. Therefore, after the conclusion of all tests, the question was posed to ChatGPT-4 twice more in separate new dialogues to avoid information cross-over. Two completely different responses were generated by ChatGPT-4:

226 Hz Standard: The 226 Hz probe tone is the standard frequency used in clinical tympanometry for all ages, including newborns. This frequency has been proven to provide reliable and accurate measurements of middle ear function in this age group. It is effective in identifying middle ear fluid, a common issue in newborns, and other conditions that might affect hearing. … In conclusion, 226 Hz (option B) is the most suitable probe tone frequency for tympanometry in newborns, due to its effectiveness in assessing middle ear function accurately in this specific age group.

and

When performing tympanometry on a newborn, the most appropriate probe tone frequency is typically 1000 Hz (Option C). Let me explain why:

Age-Related Differences: The middle ear mechanics in infants, especially newborns, differ significantly from those in older children and adults. These differences are mainly due to the size and composition of the middle ear and ear canal, as well as the compliance of the tympanic membrane.

High-Frequency Probe Tones: A 1000 Hz probe tone is recommended for newborns and infants up to around six months of age. This higher frequency provides more accurate assessments of middle ear function in this age group, as it is less affected by the high compliance of the infant ear canal.

Therefore, the most appropriate option for tympanometry in newborns is 1000 Hz (Option C), as it offers the most reliable assessment of middle ear function in this specific age group.

This indicates that ChatGPT-4 may provide different answers each time based on varying sources of information, particularly when these sources have conflicts or inconsistencies.

### Image Information Recognition

In the examination, 13 questions could be answered only through the recognition of images to extract information. ChatGPT-4 correctly answered 8 of these questions. Images 1 to 4 are from the behavioral audiology subject, images 5 and 6 are from the electrophysiological audiology subject, and image 7 is from the principles and practice of hearing devices subject. Out of the 7 images provided in total, ChatGPT-4 successfully recognized 4. The criterion for determining successful recognition was assessing the accuracy of ChatGPT-4’s interpretation of image content and its ability to extract pertinent information for answering questions. Authors SW and CM independently evaluated this aspect and subsequently performed a cross-check of their assessments.

## Discussion

### Principal Findings

This study evaluated ChatGPT-4’s performance in the 2023 Taiwan Audiologist Qualification Examination. The eligibility criteria for this examination are having a degree in audiology or a related field and a minimum of 6 months or 375 hours of clinical practice. The minimum required accuracy rate to pass the examination is set at 60%. In the 2023 examination, 88.5% of candidates achieved this accuracy rate or higher, effectively passing the examination. ChatGPT-4 achieved an overall accuracy rate of 75%, meeting the passing criterion necessary for candidates to obtain the basic qualification for practicing as clinical audiologists in Taiwan. It performed notably well in subjects that required more analytical reasoning and contextual decision-making, such as health and rehabilitation of the auditory and balance systems and auditory and speech communication disorders (including professional ethics). The proficiency of LLMs in integrating and interpreting information logically was evident in subjects demanding contextual knowledge. However, in fields such as electrophysiological audiology, which depend more on precise knowledge points, the accuracy of ChatGPT-4 was challenged when confronted with incorrect or insufficient information. In our study, the original questions were in both Chinese and English. We requested ChatGPT to provide responses in English, and the translation between the 2 languages did not negatively impact either the comprehension or the accuracy of the responses. In addition, although this research was a preliminary examination of ChatGPT-4’s capabilities in image recognition within audiology examinations, it is important to note that the number of images used was limited, and their quality and resolution were suboptimal. Nevertheless, despite these constraints, ChatGPT-4 demonstrated a moderately acceptable level of image recognition performance, successfully identifying over half of the content within the images.

Comparative analysis with the existing literature indicates that LLMs such as ChatGPT have shown promising results in medical examinations [24-26], particularly GPT-4 [27]. The model’s ability to pass examinations that are challenging for many humans has been noted [9,28]. In our study, which involved multiple-choice questions, ChatGPT was tasked with not only selecting answers but also articulating the reasoning behind its choices. Notably, ChatGPT-4 has substantially reduced the incidence of logical and statistical errors that were more prevalent in its predecessors. Its accuracy rate in examinations based on multiple-choice questions has increased from 53.6% with GPT-3 and -3.5 to 75.1% with GPT-4 [27]. The absence of logical errors, in particular, suggests that ChatGPT-4 has an enhanced ability to understand questions accurately and make decisions that are logically coherent, using the information it has access to [9,27]. This advancement is especially relevant in the context of audiology examinations, especially in the multiple-choice question format, where statistical reasoning has traditionally been less emphasized. In this study, the primary challenge faced by ChatGPT-4 in accurately answering questions was identified as information errors, which primarily manifest in 2 distinct forms: a lack of correct information sources and the presence of conflicting information sources. The former issue directly impacts the ChatGPT’s performance; for several questions, ChatGPT-4 lacked the necessary correct information to either directly answer or logically deduce the correct responses. Despite its training on an extensive database of information [29,30], it became evident that ChatGPT-4 does not possess a comprehensive knowledge base required to flawlessly address specialized queries within the field of audiology, a discipline that demands a high degree of professional expertise. Conversely, the presence of conflicting information sources contributed to erroneous responses from ChatGPT. The model, although equipped with a wealth of information, was not developed with a focus on audiological knowledge or audiology best practices. This abundance of data, however, presents a challenge in verifying the accuracy and reliability of the information, especially when multiple sources offer conflicting viewpoints on widely discussed topics. This was exemplified in this study’s results, where a question on a fundamental concept in audiology—the use of 1000 Hz in tympanometry for children—resulted in ChatGPT-4 providing 3 distinct answers. Additionally, the lowest scores were observed in questions related to electrophysiological audiology. Apart from the issue of inaccurate sources of information, another possible reason is that information about electrophysiological audiology might be more specialized than that in other subjects, thereby restricting the amount of information that ChatGPT has access to. This is unlike the case with hearing aids or auditory rehabilitation, where a vast amount of information is readily available on the internet. Altogether, this highlights the necessity for further refinement of LLMs, emphasizing the integration of more precise and professionally relevant information. This approach will ensure that responses are derived from verified and accurate data sources, pointing to a crucial direction for future research in this area [31].

### AI Chatbots and Audiology

The introduction of advanced AI models such as ChatGPT-4 has significant implications in hearing health care [18]. ChatGPT’s capacity to process and analyze extensive data makes it a potentially useful tool for patients, clinicians, and researchers [19]. Our findings suggest that given training with reliable information, even the current iteration of ChatGPT-4 holds considerable promise for application in hearing care services. This potential is likely to increase alongside the continual advancements in LLMs. The results of this study show that when faced with professional-level audiology questions, AI chatbots can provide answers with a high accuracy rate and logical reasoning. Furthermore, their ability to mimic human-like responses suggests that they are capable of assisting in educational learning and hearing care awareness. However, this is contingent upon first building a fine-tuned model with accurate information sources.

In the context of patient care, AI chatbots could act as digital audiologists, providing answers to a range of hearing-related queries. Their easy accessibility may be beneficial for early hearing screenings and prompt intervention or medical attention. AI chatbots also have the potential to educate patients about hearing issues and offer psychological support for conditions such as tinnitus. They have been shown to have potential in managing mental health concerns and demonstrate a level of empathy that can surpass that of human physicians [11,32]. The development of AI chatbots as qualified audiologists could greatly enhance teleaudiology services. For clinicians, AI chatbots could serve as supportive tools, offering rapid references or recommendations based on current research and guidelines, aiding in diagnosis and treatment suggestions [33], and facilitating the creation of diagnostic or referral reports [34]. This is especially relevant in regions with limited hearing care resources [13], where AI chatbots could play a vital role in both the education of hearing care professionals and in augmenting clinical services. This enhancement could lead to improved overall quality and availability of hearing care services, ultimately benefiting individuals with hearing impairments. Similarly, researchers in auditory science could use AI chatbots to streamline their research processes. However, the effectiveness of these proposed applications depends on the thorough and complete validation of the chatbots’ functionality and information accuracy.

### Limitations

This study represents a preliminary exploration of an AI chatbot’s performance in an audiologist qualification examination. However, several limitations must be acknowledged. First, the selected examination questions were exclusively multiple-choice, with a subset requiring integrated information for reasoning. This format lacks open-ended questions that typically mirror the complexity of real-world clinical scenarios in hearing care, where audiologists address diverse and intricate issues beyond isolated knowledge points. Future research could extend to evaluating AI chatbots in handling complex audiology cases. Second, while this study included an assessment of ChatGPT-4’s image recognition capabilities, the quality of the images in the original test files was suboptimal. Additionally, the number of questions involving image information was limited, which constrained the ability of this study to draw substantial conclusions about this functionality.

### Conclusions

In conclusion, the findings of this study show that ChatGPT 4 achieved a 75% accuracy rate in the 2023 Taiwan Audiologist Qualification Examination, thus successfully passing it. The primary reason for ChatGPT-4’s incorrect responses was identified to be information errors, including both a lack of correct information sources and the presence of conflicting information sources. Therefore, a fine-tuned model containing accurate hearing care information sources has the potential to further enhance the feasibility of AI chatbot applications in hearing care services. However, passing the examination does not imply that ChatGPT-4 can become a qualified clinical audiologist in Taiwan; rather, it only indicates that ChatGPT-4 has some basic knowledge required for the audiology profession. Adequate clinical internship hours are also a crucial requirement for the actual practice of audiology in Taiwan, and its performance in handling real clinical cases remains unknown.

## Supplementary material

10.2196/55595 Multimedia Appendix 1 ChatGPT transcripts: basic auditory science.

10.2196/55595 Multimedia Appendix 2 ChatGPT transcripts for behavioral audiology.

10.2196/55595 Multimedia Appendix 3 ChatGPT transcripts for auditory and speech communication disorders (including professional ethics).

10.2196/55595 Multimedia Appendix 4 ChatGPT transcripts for electrophysiological audiology.

10.2196/55595 Multimedia Appendix 5 ChatGPT transcripts for principles and practice of hearing devices.

10.2196/55595 Multimedia Appendix 6 ChatGPT transcripts for health and rehabilitation of the auditory and balance systems.

## Abbreviations

AI: artificial intelligence
DALL-E: Decoder-Only Autoregressive Language and Image Synthesis
LLM: large language model
