# Supplementary material for: Exploring the Performance of ChatGPT-4 in the Taiwan Audiologist Qualification Examination: Preliminary Observational Study Highlighting the Potential of AI Chatbots in Hearing Care
Source: JMIR Med Educ. 2024 Apr 26;10:e55595. doi: 10.2196/55595 (PMC11067446; doi:10.2196/55595)
Supplement: Multimedia Appendix 1 [file mededu-v10-e55595-s001.pdf]

You

Please answer the following multiple-choice question as a hearing care professional, providing reasoning and explanation: 1聽覺訊號由內耳經毛細胞往上傳至大腦聽覺區，中途會經過許多結構，其由下往上之排列順序，下列何者正確?
（A）耳蝸神經核（cochlear nucleus）→下丘（inferior colliculus）→上橄欖核複合體（superior olivary complex）→內膝體（medial geniculate body）
（B）耳蝸神經核（cochlear nucleus）→內膝體（medial geniculate body）→下丘（inferior colliculus）→上橄欖核複合體（superior olivary complex）
（C）耳蝸神經核（cochlear nucleus）→上橄欖核複合體（superior olivary complex）→下丘（inferior colliculus）→內膝體（medial geniculate body）
（D）耳蝸神經核（cochlear nucleus）→下丘（inferior colliculus）→內膝體（medial geniculate body）→上橄欖核複合體（superior olivary complex）

ChatGPT

To answer this question, we need to understand the pathway of auditory signals from the inner ear to the brain's auditory cortex. Here is the correct order:

1. **Cochlear Nucleus (耳蝸神經核)**: The audit[illegible] nerve fibers from the cochlea first synapse in the cochlear nucleus.
2. **Superior Olivary Complex (上橄欖核複合體)**: After the cochlear nucleus, the signal is sent to the superior olivary complex. This area is important for localizing sound in space.
3. **Inferior Colliculus (下丘)**: From the superior olivary complex, the signal travels to the inferior colliculus, which is a major integrating center for auditory reflexes and pathways.
4. **Medial Geniculate Body (內膝體)**: The next stop is the medial geniculate body of the thalamus, which acts as a relay station, sending the signal to the auditory cortex.

Given this pathway, the correct answer is:

（C）耳蝸神經核（cochlear nucleus）→上橄欖核複合體（superior olivary complex）→下丘（inferior colliculus）→內膝體（medial geniculate body）

This sequence correctly represents the flow of auditory information from the inner ear to the brain's auditory cortex.

You

Please answer the following multiple-choice question as a hearing care professional, providing reasoning and explanation:2聽小骨（ossicles）位於中耳腔，由鎚骨、砧骨、鐙骨組成，下列關於聽小骨之敘述，何者錯誤?
（A）鼓膜（tympanic membrane）與鎚骨之鎚骨柄（manubrium of malleus）相連
（B）鎚骨之鎚骨頭（head of malleus）與砧骨之長突（long process of incus）相連
（C）砧骨之豆狀突（lenticular process）與鐙骨之鐙骨頭（head of stapes）相連
（D）鐙骨之足板（footplate of stapes）與內耳卵圓窗（oval window）相連

ChatGPT

To identify the incorrect statement about the ossicles in the middle ear, let's review the anatomy and connections of these tiny bones:

1. **Tympanic Membrane and Malleus (鼓膜與鎚骨)**: The tympanic membrane, or eardrum, is indeed connected to the manubrium (handle) of the malleus. This is how sound vibrations from the air are transmitted to the ossicles.
2. **Malleus and Incus (鎚骨與砧骨)**: The head of the malleus connects to the body of the incus. This joint allows the transmission of vibrations from the malleus to the incus.
3. **Incus and Stapes (砧骨與鐙骨)**: The lentic[illegible] process of the incus connects to the head of the stapes. This connection is important for transmitting vibrations from the incus to the stapes.
4. **Stapes and Oval Window (鐙骨與卵圓窗)**: The footplate of the stapes sits in the oval window of the cochlea. This is where the vibrations from the middle ear are transmitted to the fluid of the inner ear.

Given this information, the incorrect statement is:

（C）砧骨之豆狀突（lenticular process）與鐙骨之鐙骨頭（head of stapes）相連

The error in this statement is in the description of the connection between the incus and stapes. The lenticular process of the incus connects not to the head of the stapes, but to its stapes' crura. The stapes is a stirrup-shaped bone, and its head does not directly engage with the incus; it's the crura (the two thin, leg-like processes) that are connected to the incus.

You

Please answer the following multiple-choice question as a hearing care professional, providing reasoning and explanation:3耳蝸在低頻聲[illegible]，內毛細胞的膜反應主要由交流分量（an alternating current component）控制，下列敘述何者正確?
（A）內毛細胞隨著基底膜的向下運動而去極化（depolarizes），向上運動而過極化（hyperpolarizes）
（B）內毛細胞隨著基底膜的向上運動而去極化，向下運動而過極化
（C）內毛細胞隨著基底膜的向內運動而去極化，向外運動而過極化
（D）內毛細胞隨著基底膜的向外運動而去極化，向內運動而過極化

ChatGPT

To answer this question, we need to understand how the inner hair cells (IHCs) in the cochlea respond to sound vibrations, particularly low-frequency sounds. The IHCs are stimulated by the movement of the basilar membrane, which in turn is influenced by sound waves. The key lies in understanding how the displacement of the basilar membrane affects the polarization of the inner hair cells.

1. **Depolarization and Hyperpolarization of Inner Hair Cells**: When the basilar membrane moves, it causes the stereocilia of the inner hair cells to bend. The direction of this bending determines whether the cell depolarizes or hyperpola[illegible]s. Depolarization occurs when the cell's membrane potential becomes more positive, while hyperpolarization occurs when it becomes more negative.
2. **Movement of the Basilar Membrane**: In response to sound waves, the basilar membrane can move up and down. The movement of the stereocilia attached to the hair cells is directly correlated to this motion.

Given these points:

- **Option (A)** suggests that depolarization occurs with the downward movement and hyperpolarization with the upward movement of the basilar membrane.
- **Option (B)** suggests the opposite – depolarization with upward movement and hyperpolarization with downward movement.

For inner hair cells, the stereocilia bend towards the tallest stereocilia when the basilar membrane moves upwards, which leads to depolarization as ion channels open. Conversely, when the basilar membrane moves downwards, the stereocilia bend away from the tallest stereocilia, leading to hyperpolarization as the channels close.

Therefore, the correct answer is:

（B）內毛細胞隨著基底膜的向上運動而去極化，向下運動而過極化

This describes the correct relationship between the movement of the basilar membrane and the polarization changes in the inner hair cells.

You

Please answer the following multiple-choice question as a hearing care professional, providing reasoning and explanation: 4 耳蝸神經電位尖峰（neural spiking）頻率有「相位鎖定（phase-locking）」的現象，下列敘述何者錯誤?
（A）神經電位尖峰和鼓膜運動之間的同步 （B）神經電位尖峰和基底膜頻率之間的同步
（C）神經電位尖峰和聲音頻率之間的同步 （D）神經電位尖峰和外毛細胞運動之間的同步

ChatGPT

"Phase-locking" in the context of cochlear neural spiking refers to the phenomenon where the timing of the neural spikes is synchronize[illegible] certain aspects of the sound stimulus. To determine which statement is incorrect, we need to understand what the neural spiking in the cochlea is typically synchronized with:

1. **Synchronization with Tympanic Membrane Movement (鼓膜運動)**: The tympanic membrane's movement is the initial mechanical response to sound waves. Neural spikes can be phase-locked to these movements, as they are the first physical representation of the sound.
2. **Synchronization with Basilar Membrane Frequency (基底膜頻率)**: The basilar membrane's movement within the cochlea is crucial in the process of sound transduction. The neural spikes are more directly phase-locked to the oscillations of the basilar membrane, which is responding to specific frequencies of sound.
3. **Synchronization with Sound Frequency (聲音頻率)**: The frequency of the sound itself is an important factor. Neural spiking can be phase-locked to the frequency of the sound, especially at lower frequencies. This synchronization helps in the perception of pitch and frequency modulation.
4. **Synchronization with Outer Hair Cell Movement (外毛細胞運動)**: While outer hair cells play a crucial role in amplifying and fine-tuning the movements of the basilar membrane, the neural spikes (especially from the auditory nerve[illegible]ers connected to the inner hair cells) are not typically synchronized with the movement of the outer hair cells. Instead, they are more directly related to the mechanical changes translated by the inner hair cells.

Given this information, the incorrect statement is:

（D）神經電位尖峰和外毛細胞運動之間的同步

This is because the phase-locking phenomenon in the cochlear nerve primarily involves synchronization with the movements of the basilar membrane and the tympanic membrane, as well as the frequency of the sound, rather than the movement of the outer hair cells.

You

Please answer the following multiple-choice question as a hearing care professional, providing reasoning and explanation: 5 關於耳蝸電位（cochlear electrical potential），下列何者屬於交流電位（alternating current）? ①耳蝸微
音電位（cochlear microphonic） ②總合電位（summation potential） ③靜止電位（resting potential）
④耳蝸內電位（endocochlear potential）
（A）僅① （B）②④ （C）①② （D）③④

ChatGPT

To determine which of the listed cochlear electrical potentials are alternating current (AC) potentials, let's examine each one:

1. **Cochlear Microphonic (耳蝸微音電位)**: The cochlear microphonic is an AC potential. It closely follows the frequency and phase of the sound stimulus, reflecting the mechanical vibrations within the cochlea. This potential is primarily generated by the outer hair cells as they respond to sound.
2. **Summation Potential (總合電位)**: The summation potential is a direct current (DC) potential, not AC. It is associated with sustained displacements of the basilar membrane and does not oscillate in phase with the sound stimulus.
3. **Resting Potential (靜止電位)**: The resting potential is also a DC potential. It is the steady-state potential difference between various parts of the cochlea when there is no sound stimulus.
4. **Endocochlear Potential (耳蝸內電位)**: The[illegible]docochlear potential is a steady DC potential found within the scala media of the cochlea. It is not an alternating current potential.

Based on this information, the only cochlear electrical potential that is an AC potential is the cochlear microphonic. Therefore, the correct answer is:

（A）僅① - Only the cochlear microphonic is an alternating current potential.

You

Please answer the following multiple-choice question as a hearing care professional, providing reasoning and explanation: 6 關於描述中耳肌肉名稱、顱神經支配、肌肉連接的起 - 迄構造，下列何者正確?
（A）鼓膜張肌（tensor tympani muscle）、顏面神經、匙狀突（cochleariform process） - 鐙骨（stapes）
（B）鐙骨肌（stapes muscle）、顏面神經、錐隆起（pyramidal eminence） - 鐙骨
（C）鐙骨肌、顏面神經、匙狀突 - 鐙骨
（D）鼓膜張肌、三叉神經、匙狀突 - 鐙骨

ChatGPT

To answer this question correctly, we need to consider the names of the middle ear muscles, the cranial nerves that innervate them, and the structures they connect from and to. There are two primary muscles in the middle ear:

1. **Tensor Tympani Muscle (鼓膜張肌)**: This muscle is innervated by a branch of the trigeminal nerve (V), specifically the mandibular branch (V3). It is attached from the cochleariform process and extends to the manubrium of the malleus. Its function is to tense the tympanic membrane.
2. **Stapedius Muscle (鐙骨肌)**: This muscle is innervated by a branch of the facial nerve (VII). It originates from the pyramidal eminence and attaches to the neck of the stapes. The stapedius muscle's contraction reduces the movement of the stapes at the oval window, thus protecting the inner ear from loud sounds.

Given these descriptions, let's evaluate the options:

- **Option (A)** incorrectly states that the tensor tympani muscle is innervated by the facial nerve and attaches to the stapes.
- **Option (B)** incorrectly refers to the "stape[illegible]uscle" and associates it with the facial nerve; it should be the stapedius muscle, and it does attach to the stapes from the pyramidal eminence.
- **Option (C)** again incorrectly associates the stapedius muscle with the facial nerve and states an incorrect origin.
- **Option (D)** correctly states that the tensor tympani muscle is innervated by the trigeminal nerve and attaches from the cochleariform process, but it incorrectly states that it attaches to the stapes; it attaches to the manubrium of the malleus.

Therefore, all the given options contain inaccuracies, but the closest to being correct is **Option (D)**, although it contains an error regarding the attachment point of the tensor tympani muscle.

You

Please answer the following multiple-choice question as a hearing care professional, providing reasoning and explanation: 7 動脈血管供應到耳蝸，由粗到細之順序何者正確? ①anterior inferior cerebellar artery ②basilar artery
③labyrinthine artery ④spiral modiolar artery ⑤common cochlear artery
（A）[illegible] （B）[illegible] （C）[illegible] （D）[illegible]

ChatGPT

To answer this question, we need to understand the arterial blood supply to the cochlea and the hierarchy of these arteries in terms of their size and branching pattern:

1. **Basilar Artery (基底動脈)**: This is a major artery that supplies blood to much of the brainstem and posterior part of the brain. It's one of the larger arteries in this context.
2. **Anterior Inferior Cerebellar Artery (前下小腦動脈)**: Branching off from the basilar artery, the anterior inferior cerebellar artery supplies blood to parts of the cerebellum and brainstem.
3. **Labyrinthine Artery (迷路動脈)**: This smaller artery typically branches off from the anterior inferior cerebellar artery, though it can also come directly from the basilar artery. It supplies blood to the inner ear, including the cochlea.
4. **Common Cochlear Artery (共通耳蝸動脈)**: This is a branch of the labyrinthine artery that specifically supplies the cochlea.
5. **Spiral Modiolar Artery (螺旋骨迴動脈)**: This is an even smaller artery, a branch of the cochlear artery, which supplies blood to the modiolus and the spiral lamina of the cochlea.

Based on this order of branching and decr[illegible]ng size, the correct sequence from larger to smaller arteries would be:

（B） ② Basilar Artery → ① Anterior Inferior Cerebellar Artery → ③ Labyrinthine Artery → ⑤ Common Cochlear Artery → ④ Spiral Modiolar Artery

This sequence represents the typical flow of blood from the larger basilar artery down to the smaller spiral modiolar artery that supplies blood specifically to the cochlea.

You

Please answer the following multiple-choice question as a hearing care professional, providing reasoning and explanation: 8 耳部的構造大多位於顳骨內。顳骨可分為那五個部分?
（A）乳突部、髁狀突、鰭形部、鼓室部、翼狀部
（B）乳突部、髁狀突、鰭形部、鼓室部、顳窩部
（C）乳突部、岩部、鱗狀部、鼓室部、莖狀突
（D）乳突部、岩部、鱗狀部、鼓室部、柱狀突

ChatGPT

The temporal bone, which houses much of the ear's structure, is indeed divided into several parts. Understanding these parts is crucial for a comprehensive grasp of ear anatomy. Here's a breakdown of the components of the temporal bone:

1. **Mastoid Part (乳突部)**: This section includes the mastoid process, a prominent bone behind the ear. It contains air cells and is important for the middle ear.
2. **Squamous Part (鱗狀部)**: The squamous part is the flat, fan-shaped portion of the temporal bone.
3. **Petrous Part (岩部)**: This is the hardest part of the temporal bone, housing the inner ear structures.
4. **Tympanic Part (鼓室部)**: This part forms the front and lower walls of the external acoustic meatus and part of the tympanic cavity.
5. **Styloid Process (莖狀突)**: The styloid process is a slender, pointed piece of bone just below the ear. It serves as an anchor point for several muscles and ligaments.

Given these descriptions, the correct answer to the question about the five parts of the temporal bone is:

（C）乳突部、岩部、鱗狀部、鼓室部、莖[illegible]

This option correctly lists all the major parts of the temporal bone: the mastoid part, petrous part, squamous part, tympanic part, and the styloid process.

You

Please answer the following multiple-choice question as a hearing care professional, providing reasoning and explanation: 9 有關聲反射與刺激音頻率的相關性，下列那一項尚未定論?
（A）閾值（threshold） （B）潛時（latency） （C）適應（adaptation） （D）反應強度（magnitude）

ChatGPT

In the context of auditory reflexes and their relationship with stimulus frequency, several factors are considered, such as threshold, latency, adaptation, and response magnitude. Each of these aspects has been studied to understand how the auditory system reacts to different sound frequencies. Let's review these aspects:

1. **Threshold (閾值)**: This refers to the minimum sound level at which a response (such as an auditory reflex) is elicited. There is substantial research on how thresholds vary with frequency, but there might still be debates or ongoing research, especially regarding how this applies to different types of auditory reflexes.
2. **Latency (潛時)**: Latency is the time between the onset of a sound and the onset of the reflex response. While the general relationship between latency and frequency is understood, there could be complexities and variations based on individual differences or specific conditions.
3. **Adaptation (適應)**: Adaptation in the context of auditory reflexes would refer to how the reflex response changes over time with continuous or repeated stimulation. The relationship between adaptation and frequency is an area that can have varying conclusions, depending on the type of reflex and the nature of the auditory stimulus.
4. **Response Magnitude (反應強度)**: This refers to the strength or intensity of the reflex in response to a sound of a particular frequency. The relationship between response magnitude and frequency is generally understood, but there might still be areas of research and debate, especially concerning different types of auditory reflexes or in varying auditory conditions.

Given these considerations, it's likely that [illegible]ese aspects have areas that are still under investigation or debate. However, **adaptation (C)** might be the most complex and least conclusively understood aspect, as it involves dynamic changes over time and can vary greatly depending on the specific characteristics of the sound stimulus and the individual's auditory system. Therefore, the most likely answer is:

（C）適應 (adaptation)

This is because adaptation involves a dynamic and time-dependent response of the auditory system, which can vary significantly based on frequency, duration, and other characteristics of the sound, as well as individual differences in auditory processing.

You

Please answer the following multiple-choice question as a hearing care professional, providing reasoning and explanation: 10 下列那一個蛋白質分子與離子位於外毛細胞側壁且參與其電生理可動性（electromotility），因而使耳蝸成為聽覺擴大器?
（A） connexn26；鎂（magnesium） （B） pendrin；氯（chloride）
（C） motilin；鎂（magnesium） （D）[illegible]stin；氯（chloride）

ChatGPT

The key to answering this question lies in understanding which protein is specifically associated with the electromotility of outer hair cells in the cochlea, contributing to their role as cochlear amplifiers. This electromotility is a unique property of outer hair cells that enables them to change their length in response to electrical stimulation, thereby enhancing the sensitivity and frequency selectivity of the cochlea.

Let's review the options:

1. **Connexin26 (A)**: Connexin26 is involved in forming gap junctions in various tissues, including the cochlea, but it is not directly responsible for the electromotility of outer hair cells.
2. **Pendrin (B)**: Pendrin is a protein associated with ion transport, particularly chloride ions, but it is not the protein responsible for the electromotility of outer hair cells.
3. **Motilin (C)**: Motilin is a peptide hormone, primarily associated with gastrointestinal motility, not with cochlear function or electromotility in outer hair cells.
4. **Prestin (D)**: Prestin is indeed the motor pr[illegible]n specifically found in the cell membrane of outer hair cells in the cochlea. It is responsible for their unique electromotility. The presence of chloride ions is critical for the functioning of prestin.

Given this information, the correct answer is:

（D）prestin；氯（chloride）

Prestin is the motor protein that enables outer hair cells to contract and expand rapidly in response to electrical signals, enhancing the motion of the basilar membrane and thus the sensitivity and selectivity of the cochlea. Chloride ions play a crucial role in the functioning of prestin, making it an essential part of the cochlear amplifier mechanism.

You

Please answer the following multiple-choice question as a hearing care professional, providing reasoning and explanation: 11 下列有關耳咽管結構的敘述，何者錯誤?
（A）靠近鼻咽腔部為軟骨部（cartilage portion），約占整個耳咽管長度的 2/3
（B）小兒耳咽管方向是近似水平的
（C）成人耳咽管以 45 度由中耳腔下降至[illegible]腔
（D）成人耳咽管最狹窄的部位為軟骨部與硬骨部交界處之峽部（isthmus），開口約 3～4 mm 寬

ChatGPT

To determine which statement about the Eustachian tube (also known as the auditory tube or pharyngotympanic tube) is incorrect, let's review the anatomy and characteristics of this structure:

1. **Cartilage Portion (A)**: The Eustachian tube comprises both bony and cartilaginous sections. The cartilaginous portion is indeed longer, approximately two-thirds of the tube's total length, and it is closer to the nasopharynx.
2. **Orientation in Children (B)**: In children, the Eustachian tube is indeed more horizontal compared to adults. This orientation is a factor in the increased incidence of middle ear infections in children, as it can make drainage of fluid from the middle ear less effective.
3. **Adult Eustachian Tube Angle (C)**: In adults, the Eustachian tube is angled more steeply, around 45 degrees, from the middle ear down to the nasopharynx. This steeper angle aids in better drainage and ventilation of the middle ear[illegible]
4. **Narrowest Part (D)**: The narrowest part of the Eustachian tube is indeed the isthmus, where the bony and cartilaginous parts meet. However, the width of this narrowest part is typically smaller than 3-4 mm; it is usually around 1-2 mm wide in adults.

Given this information, the incorrect statement is:

（D）成人耳咽管最狹窄的部位為軟骨部與硬骨部交界處之峽部（isthmus），開口約 3～4 mm 寬

The error in this statement lies in the width of the isthmus of the Eustachian tube, which is typically narrower than 3-4 mm in adults.

You

Please answer the following multiple-choice question as a hearing care professional, providing reasoning and explanation: 12 在耳膜上可以看到許多構造，下列敘述何者錯誤?
（A）鬆弛部（pars flaccida）位於耳膜上 1/4（superior quadrant）
（B）光錐（cone of light）位於前下方
（C）透過耳膜有時可見砧骨（incus）長突位於後上方
（D）耳膜直徑大概 1 公分，其寬度略長於[illegible]

ChatGPT

To address the question about the structures of the tympanic membrane (eardrum) and identify the incorrect statement, let's review each statement:

1. **Pars Flaccida (A)**: The pars flaccida of the tympanic membrane is indeed located in the superior (upper) quadrant. It is a small, slack, superior section of the tympanic membrane.
2. **Cone of Light (B)**: The cone of light is a reflection seen on the tympanic membrane, typically oriented anteroinferiorly (towards the front and lower part of the eardrum). This reflection is due to the light from an otoscope.
3. **Visibility of the Incus (C)**: Through the tympanic membrane, it is sometimes possible to see the long process of the incus. This structure is usually visible in the upper posterior (back) quadrant of the tympanic membrane.
4. **Size and Shape of the Tympanic Membrane (D)**: The tympanic membrane is roughly circular and about 1 centimeter in diameter. However, it is typically more oval than round, with the height being slightly greater than the width. This means the membrane is usually taller than it is wide.

Based on this information, the incorrect statement is:

（D）耳膜直徑大概 1 公分，其寬度略長於高度

This statement is incorrect because the tympanic membrane is generally taller than it is wide, not wider than it is tall.

You

Please answer the following multiple-choice question as a hearing care professional, providing reasoning and explanation: 13 Kemp 最早報告的耳聲傳射形式中，也有人稱它為 Kemp 迴聲（Kemp's echo）指的是：
（A） DPOAE （B） SOAE （C） TEOAE （D） SFOAE

ChatGPT

The phenomenon referred to as "Kemp's echo" is related to the discovery of otoacoustic emissions (OAEs) by David Kemp. Otoacou[illegible] emissions are sounds generated by the inner ear, specifically the cochlea, either spontaneou[illegible] or in response to an auditory stimulus. Kemp's discovery was groundbreaking because it demonstrated that the ear not only receives but also generates sound. Let's look at the types of OAEs referenced in the options:

1. **DPOAE (Distortion Product Otoacoustic Emissions) (A)**: These emissions are generated in response to two simultaneous tones of different frequencies. They are commonly used in hearing screenings and diagnostics.
2. **SOAE (Spontaneous Otoacoustic Emissions) (B)**: These are sounds emitted by the ear without any external stimulus. Not all individuals have measurable SOAEs.
3. **TEOAE (Transient Evoked Otoacoustic Emissions) (C)**: These are sounds the ear generates in response to a brief auditory stimulus, like a click. Kemp's initial discovery and description of otoacoustic emissions are most closely associated with this type.
4. **SFOAE (Stimulus Frequency Otoacoustic Emissions) (D)**: These are sounds emitted in response to a continuous tone of a single frequency.

Given this context, "Kemp's echo" is most closely associated with **Transient Evoked Otoacoustic Emissions (TEOAE)**. Kemp's original work described emissions evoked by transient stimuli, which is the defining characteristic of TEOAEs. T[illegible]fore, the correct answer is:

（C） TEOAE

You

Please answer the following multiple-choice question as a hearing care professional, providing reasoning and explanation: 14 以外淋巴為基準點（reference point）將之視為 0 millivorlt（mV），此時毛細胞內的電位稱為 intracellular potential，intracellular potential 約是多少 mV?
（A）+80 （B）-70 （C）+150 （D）-150

ChatGPT

To answer this question, we need to consider the typical intracellular potential of hair cells in the cochlea, especially relative to the potential of the surrounding fluid, the perilymph (often considered as the reference point set at 0 millivolts).

The perilymph, which is the fluid outside the hair cells in the cochlea, is typically compared to the endolymph, the fluid inside the cochlear du[illegible] where the hair cells' stereocilia are immersed. The endolymph has a high potassium concentra[illegible]on and a positive potential relative to the perilymph.

1. **Endocochlear Potential**: The potential difference between the endolymph and the perilymph (known as the endocochlear potential) is about +80 mV to +100 mV (endolymph being more positive).
2. **Intracellular Potential of Hair Cells**: The intracellular potential of hair cells, when measured against the perilymph, is typically around -70 mV. This means the inside of the hair cell is 70 mV more negative than the perilymph.

Given these considerations, when using the perilymph as a reference point (set at 0 mV), the intracellular potential of hair cells is approximately:

（B）-70 mV

This value reflects the typical resting membrane potential of hair cells in the cochlea.

You

Please answer the following multiple-choice question as a hearing care professional, providing reasoning and explanation: 15 下列何者為聽覺系統克服中耳阻抗不匹配（impedance mismatch）的因素?
（A）鼓膜與卵圓窗的面積差，以及三塊聽小骨的槓桿作用
（B）三塊聽小骨的槓桿作用，以及三塊聽小骨的質量差異
（C）鼓膜與卵圓窗的面積差，以及三塊聽小骨的硬度差異
（D）三塊聽小骨的質量與硬度差異

ChatGPT

The auditory system effectively overcomes the impedance mismatch between the air in the external auditory canal and the fluid in the cochlea. This mismatch is addressed by two primary mechanisms involving the tympanic membrane (eardrum) and the ossicles (the three small bones in the middle ear). Let's examine each option:

1. **Area Difference between Tympanic Membrane and Oval Window, and Lever Action of Ossicles (A)**: The surface area of the tympanic membrane is much larger than that of the oval window (the part of the cochlea where the stapes footplate sits). This difference in area helps concentrate the sound energy, increasing the pressure ex[illegible]d on the oval window. Additionally, the ossicles act as a lever system, further amplifying the sound pressure. This option correctly identifies two key factors in overcoming impedance mismatch.
2. **Lever Action of Ossicles, and Mass Difference of the Ossicles (B)**: While the lever action of the ossicles is a factor, the mass difference of the ossicles is not a significant factor in overcoming the impedance mismatch.
3. **Area Difference between Tympanic Membrane and Oval Window, and Stiffness Difference of the Ossicles (C)**: The area difference is a key factor, but the stiffness difference of the ossicles is not typically cited as a primary mechanism for impedance matching.
4. **Mass and Stiffness Differences of the Ossicles (D)**: The differences in mass and stiffness of the ossicles contribute to the overall mechanics of the middle ear, but they are not the primary means by which the impedance mismatch is overcome.

Based on this analysis, the correct answer is:

（A）鼓膜與卵圓窗的面積差，以及三塊聽小骨的槓桿作用

This option correctly identifies the area difference between the tympanic membrane and the oval window, along with the lever action of the o[illegible]cles, as the key factors in overcoming the impedance mismatch in the middle ear.

You

Please answer the following multiple-choice question as a hearing care professional, providing reasoning and explanation: 16 在中樞聽覺路徑（central auditory pathways）中，可觀察到不同型態的神經細胞表現不同的神經放電模式（firing patterns），其中 octopus cells、small stellate 及 large monopolar cells 以 peri-stimulus time histograms（PSTHs）顯現的放電模式是下列何種?
（A） primary-like units （B） onset units （C） choppers units （D） pausers units

ChatGPT

In the central auditory pathways, different types of neurons exhibit distinct firing patterns, which can be observed through peri-stimulus time histograms (PSTHs). Let's examine the firing patterns associated with the neuron types mentioned in the question: octopus cells, small stellate cells, and large monopolar cells.

1. **Octopus Cells**: Octopus cells, located in t[illegible]ochlear nucleus, are known for their ability to respond to very rapid changes in sound. They typically exhibit an "onset" firing pattern, meaning they fire rapidly at the onset of a stimulus and then quickly reduce their firing rate.
2. **Small Stellate Cells**: These cells can exhibit a variety of firing patterns, but they are often associated with "chopper" units. Chopper units produce a regular, chopping firing pattern, with a fairly consistent interval between spikes.
3. **Large Monopolar Cells**: These cells are also known to exhibit the "primary-like" firing pattern, similar to the firing patterns observed in nerve fibers of the auditory nerve. This pattern closely follows the temporal structure of the sound stimulus.

Given this information, the firing patterns for these cells as represented in PSTHs would be:

- Octopus Cells: Onset units (B)
- Small Stellate Cells: Choppers units (C)
- Large Monopolar Cells: Primary-like units (A)

However, since the question groups these three cell types together and asks for a single response, we need to consider the most dominant or characteristic pattern among them. The most distinctive pattern among these is the "ons[illegible] pattern, particularly due to the unique response of octopus cells.

Therefore, the most appropriate answer is:

（B）onset units

This reflects the prominent firing pattern of octopus cells, which is a distinct and defining characteristic among the neuron types listed.

You

Please answer the following multiple-choice question as a hearing care professional, providing reasoning and explanation: 17 關於中樞聽覺路徑的神經放電型態，下列何者錯誤?
（A）通常被稱為 peri-stimulus time histograms（PSTHs）
（B） primary-like units 放電型態與前腹側耳蝸神經核（anterior ventral cochlear nucleus）及後腹側耳蝸神經核（posterior ventral cochlear nucleus）內的 bushy cells 有關
（C） choppers 的速率與 tone burst 的強度、音長及速率相關
（D） pausers 及 buildup units 與背側耳蝸神經核（dorsal cochlear nucleus）的 stellate cells 相關

ChatGPT

To address this question, we need to examine the characteristics of different neural firing patterns in the central auditory pathways and associate them with specific types of neurons or regions in the auditory system. Let's evaluate each statement:

1. **PSTHs (A)**: Peri-stimulus time histograms (PSTHs) are indeed commonly used to describe the firing patterns of neurons in response to auditory stimuli. This method plots the timing of neural spikes relative to the onset of a stimulus, allowing the observation of various firing patterns.
2. **Primary-Like Units (B)**: Primary-like units exhibit firing patterns that closely follow the temporal structure of the sound stimulus, similar to those of auditory nerve fibers. These patterns are indeed associated with bushy cells in the anterior and posterior ventral cochlear nucleus.
3. **Choppers (C)**: Chopper units exhibit a regular, chopping firing pattern. The rate of this chopping pattern is indeed influenced by the characteristics of the sound stimulus, such as the intensity, duration, and rate of tone bursts.
4. **Pausers and Buildup Units (D)**: Pauser an[illegible]ildup units represent specific firing patterns observed in certain neurons. However, these patterns are more typically associated with neurons in the inferior colliculus and other higher auditory centers, rather than exclusively with stellate cells in the dorsal cochlear nucleus.

Given these considerations, the statement that is incorrect or the least accurately described is:

（D）pausers 及 buildup units 與背側耳蝸神經核（dorsal cochlear nucleus）的 stellate cells 相關

This statement is less accurate because pausers and buildup units are not exclusively or primarily associated with stellate cells in the dorsal cochlear nucleus. They are more broadly associated with various neurons in higher auditory processing centers.

You

Please answer the following multiple-choice question as a hearing care professional, providing reasoning and explanation: 18 中樞聽覺的內側上橄欖核（medial superior olive, MSO）神經細胞之放電頻率，主要取決於下列何者?
（A）取決於來自同側耳朵的聽覺神經刺激
（B）取決於來自對側耳朵的聽覺神經刺激
（C）取決於來自兩耳的聽覺神經刺激的時間一致性

（D）取決於來自兩耳的聽覺神經刺激的空間一致性

ChatGPT

The medial superior olive (MSO) is a key structure in the auditory brainstem involved in the processing of binaural cues, particularly those related to sound localization. The firing rate of neurons in the MSO is influenced by the timing differences in the sound signals received from both ears. Let's examine the options:

1. **Stimulation from the Same Side Ear (A)**: While input from the same side ear (ipsilateral ear) is important, the MSO primarily processes differences in the timing of signals from both ears.
2. **Stimulation from the Opposite Side Ear (B)**: Similarly, stimulation from the opposite side ear (contralateral ear) alone is not the sole determinant of the firing rate of MSO neurons.
3. **Temporal Consistency of Auditory Nerve Stimuli from Both Ears (C)**: This is the critical factor. The MSO neurons are sensitive to the interaural time differences (ITDs) - the small differences in the time at which a sound arrives at each ear. These neurons help in localizing sounds on the horizontal plane by comparing the timing of the sound signals received from both ears.
4. **Spatial Consistency of Auditory Nerve Stimuli from Both Ears (D)**: While spatial factors are important in auditory processing, the specific role of the MSO is more focused on the timing (temporal aspects) rather than spatial consistency.

Given this information, the correct answer is:

（C）取決於來自兩耳的聽覺神經刺激的時間一致性

This reflects the role of the MSO in processing interaural time differences, a crucial aspect of sound localization and spatial hearing.

You

Please answer the following multiple-choice question as a hearing care professional, providing reasoning and explanation: 19 聽覺訊號上傳至大腦聽覺區的路徑會經過許多結構，其中第一次交叉到對側是發生在那一部位?
（A）下丘（inferior colliculus）部分交叉到對側之耳蝸神經核（cochlear nucleus）
（B）上橄欖核複合體（superior olivary complex）部分交叉到對側之耳蝸神經核（cochlear nucleus）
（C）內側膝狀體（medial geniculate body）部分交叉到對側之上橄欖核複合體（superior olivary complex）
（D）耳蝸神經核（cochlear nucleus）部分交叉到對側之上橄欖核複合體（superior olivary complex）

ChatGPT

In the auditory pathways, the signal from each ear travels through several structures in the brainstem and brain, with some of the pathways crossing over (decussating) to the opposite side (contralateral side). Understanding where this first crossover occurs is crucial to grasp the auditory signal processing. Let's evaluate each option:

1. **Inferior Colliculus (A)**: The inferior colliculus is involved in the auditory pathway, but it is not the site of the first crossover from one side of the auditory system to the other.
2. **Superior Olivary Complex (B)**: The superior olivary complex (SOC) is indeed one of the first major sites where the auditory pathways from both ears converge and interact. Some of the nerve fibers from the cochlear nucleus cross over to the opposite superior olivary complex, making this the first point of such crossover.
3. **Medial Geniculate Body (C)**: The medial geniculate body is a part of the thalamus and is involved in the auditory pathway, but it is not where the first contralateral crossing occurs.
4. **Cochlear Nucleus to Superior Olivary Complex (D)**: The cochlear nuclei, located on each side of the brainstem, receive input directly from the cochlea of the same side. From here, some fibers do cross over to the opposite side and project to the superior olivary complex. This represents the first major crossover in the auditory pathway.

The correct answer is:

（D）耳蝸神經核（cochlear nucleus）部分交叉到對側之上橄欖核複合體（superior olivary complex）

This option correctly identifies the first major contralateral crossing in the auditory pathway, which occurs from the cochlear nucleus to the superior olivary complex.

You

Please answer the following multiple-choice question as a hearing care professional, providing reasoning and explanation: 20 關於雙耳聽覺訊息編碼，下丘（inferior colliculus）接受上橄欖核複合體（superior olivary complex）之訊息，下列敘述何者正確?
（A）從同側內側上橄欖核（medial superior olive, MSO）接受兩耳強度差（interaural level difference, ILD）
（B）從同側外側上橄欖核（lateral superior olive, LSO）接受兩耳時間差（interaural time difference, ITD）
（C）從對側外側上橄欖核（lateral superior olive, LSO）接受兩耳強度差（interaural level difference, ILD）
（D）從對側內側上橄欖核（medial superior olive, MSO）接受兩耳時間差（interaural time difference, ITD）

ChatGPT

In the auditory pathways, the inferior colliculus plays a crucial role in integrating and processing binaural auditory information, including interaural level differences (ILDs) and interaural time differences (ITDs). These differences are key for sound localization. Let's examine each option:

1. **Medial Superior Olive (MSO) and ILD (A)**: The medial superior olive (MSO) is primarily involved in processing ITDs, not ILDs. ITDs are differences in the timing of sound arriving at each ear, particularly important for low-frequency sounds.
2. **Lateral Superior Olive (LSO) and ITD (B)**: The lateral superior olive (LSO) is involved in processing ILDs, which are differences in the intensity or level of sound arriving at each ear, rather than ITDs. ILDs are more critical for high-frequency sounds.
3. **Lateral Superior Olive (LSO) and ILD (C)**: This is correct. The LSO processes ILDs, but it should be noted that it receives input from both ipsilateral (same side) and contralateral (opposite side) ears, with the intensity of sound from the ipsilateral ear being the dominant factor.
4. **Medial Superior Olive (MSO) and ITD (D)**: The MSO processes ITDs, but it receives input from both ears, not just the contralateral side.

Given this information, the correct answer is:

（C）從對側外側上橄欖核（lateral superior olive, LSO）接受兩耳強度差（interaural level difference, ILD）

This statement correctly identifies the role of the LSO in processing ILDs, which are critical for the localization of high-frequency sounds. The LSO compares the intensity of sounds coming from both ears to aid in determining the direction of the sound source.

You

Please answer the following multiple-choice question as a hearing care professional, providing reasoning and explanation: 21 耳蝸神經核（cochlear nuclei）屬於聽覺系統上傳路徑的一部分，其組成包括下列何者?
① 背耳蝸神經核（dorsal cochlear nucleus）
② 內側耳蝸神經核（medial cochlear nucleus）
③ 前腹耳蝸神經核（anteroventral cochlear nucleus）
④ 後腹耳蝸神經核（posteroventral cochlear nucleus）
⑤ 外側上橄欖核（lateral superior olive, LSO）
（A）①③④　（B）②④⑤　（C）①②③　（D）①②④⑤

ChatGPT

To identify the correct components of the cochlear nuclei, let's examine the options provided. The cochlear nuclei are the first relay stations in the central auditory system where the auditory nerve fibers synapse after leaving the cochlea. There are two main divisions of the cochlear nucleus:

1. **Dorsal Cochlear Nucleus (背耳蝸神經核)**: This is one of the primary divisions of the cochlear nucleus.
2. **Anteroventral Cochlear Nucleus (前腹耳蝸神經核)**: This is another primary division of the cochlear nucleus.
3. **Posteroventral Cochlear Nucleus (後腹耳蝸神經核)**: This is also a primary division of the cochlear nucleus.
4. **Medial Cochlear Nucleus (內側耳蝸神經核)** and **Lateral Superior Olive (外側上橄欖核, LSO)**: These are not traditionally recognized as divisions of the cochlear nucleus. The medial cochlear nucleus is not a standard term used in the description of the cochlear nuclei, and the Lateral Superior Olive (LSO) is a separate structure in the auditory pathway, not a part of the cochlear nuclei.

Given this information, the components of the cochlear nuclei include the dorsal cochlear nucleus, the anteroventral cochlear nucleus, and the posteroventral cochlear nucleus. Therefore, the correct answer is:

（A）①背耳蝸神經核（dorsal cochlear nucleus），③前腹耳蝸神經核（anteroventral cochlear nucleus），④後腹耳蝸神經核（posteroventral cochlear nucleus）

You

Please answer the following multiple-choice question as a hearing care professional, providing reasoning and explanation: 22 關於音調排列分布（tonotopic organization）的敘述，下列何者正確?
（A）高頻出現在內側上橄欖核（medial superior olive, MSO）
（B）低頻出現在外側上橄欖核（lateral superior olive, LSO）
（C）特色頻率（characteristic frequencies, CFs）可以在聽覺系統中自耳蝸核至聽覺皮質的所有位置中出現
（D）特色頻率（characteristic frequencies, CFs）自下丘的中央核（central nuclei）的背側高頻至腹內側低頻遞減

ChatGPT

Tonotopic organization refers to the systematic arrangement of neurons and their responses to different frequencies, where certain areas of neurons are responsive to specific frequencies. This organization is maintained throughout various parts of the auditory system. Let's review the statements:

1. **High Frequencies in the Medial Superior Olive (MSO) (A)**: The medial superior olive (MSO) is involved in processing interaural time differences (ITDs) and is not specifically characterized by a tonotopic organization of high frequencies.
2. **Low Frequencies in the Lateral Superior Olive (LSO) (B)**: The lateral superior olive (LSO) processes interaural level differences (ILDs) and, like the MSO, is not specifically characterized by a tonotopic organization of low frequencies.
3. **Characteristic Frequencies (CFs) Throughout the Auditory System (C)**: This statement is accurate. The concept of characteristic frequencies (CFs), which are specific frequencies to which a neuron is most responsive, is a fundamental aspect of the tonotopic organization. This organization is maintained from the cochlear nuclei up to the auditory cortex, with each level of the auditory pathway preserving this frequency-specific organization.
4. **CFs from High to Low Frequencies in the Inferior Colliculus (D)**: While the inferior colliculus does exhibit tonotopic organization, this statement incorrectly describes the orientation of frequencies in its central nuclei.

Given these explanations, the correct statement about tonotopic organization is:

（C）特色頻率（characteristic frequencies, CFs）可以在聽覺系統中自耳蝸核至聽覺皮質的所有位置中出現

This statement correctly identifies that characteristic frequencies are found throughout the auditory system, from the level of the cochlear nuclei to the auditory cortex, maintaining the tonotopic organization.

You

Please answer the following multiple-choice question as a hearing care professional, providing reasoning and explanation: 23 單一聽神經纖維在多少分貝的聲音範圍內會增加放電速率?
（A）25～70 dB　（B）30～60 dB　（C）20～50 dB　（D）40～70 dB

ChatGPT

The dynamic range over which a single auditory nerve fiber increases its firing rate in response to sound intensity is a crucial aspect of auditory processing. This range varies for different fibers, especially depending on the characteristic frequency (CF) to which the fiber is most sensitive. However, there are general patterns observed in auditory nerve fibers:

1. **Low-Level Range**: At the lower end of the intensity spectrum, auditory nerve fibers begin to respond at a certain threshold level. This threshold varies but is often around 20-30 dB for fibers responding to moderate frequencies.
2. **Upper-Level Range**: As the intensity increases, the firing rate of the auditory nerve fiber increases until it reaches a point of saturation, where further increases in intensity do not produce a significant increase in the firing rate. This saturation level can be around 60-70 dB for many fibers.

Given this understanding, a single auditory nerve fiber typically shows an increased firing rate across a range of approximately 30-40 dB. This range can vary but is often from a lower level of about 20-30 dB to an upper level of about 60-70 dB.

Considering the options provided:

- (A) 25～70 dB
- (B) 30～60 dB
- (C) 20～50 dB
- (D) 40～70 dB

The most encompassing and typical range for the increase in firing rate of a single auditory nerve fiber would be:

（A） 25～70 dB

This range captures the lower threshold and the upper saturation point for many auditory nerve fibers, although it's important to note that there is variability depending on the specific characteristic frequency of each fiber.

You

Please answer the following multiple-choice question as a hearing care professional, providing reasoning and explanation: 24 關於大腦中的聽覺皮質，下列敘述何者錯誤?
（A）位在大腦枕葉（occipital lobe）的兩側
（B）屬於中樞聽覺系統一環
（C）位在大腦的外側溝（sylvius fissure）
（D）常使用功能性磁振造影（functional magnetic resonance imaging, fMRI）去研究聽覺皮質的活動

ChatGPT

To identify the incorrect statement about the auditory cortex, let's review each provided option:

1. **Located in the Occipital Lobes (A)**: The auditory cortex is not located in the occipital lobes. The occipital lobes are primarily associated with visual processing. The auditory cortex is actually located in the temporal lobes.
2. **Part of the Central Auditory System (B)**: This statement is correct. The auditory cortex is indeed a critical part of the central auditory system, responsible for the processing and interpretation of sound information.
3. **Located in the Sylvian Fissure (C)**: The auditory cortex, particularly the primary auditory cortex (Brodmann areas 41 and 42), is located in the temporal lobe, near the Sylvian fissure (also known as the lateral sulcus). It is not within the fissure itself but is located on the superior temporal gyrus just adjacent to it.
4. **Studied Using Functional Magnetic Resonance Imaging (fMRI) (D)**: This statement is correct. Functional magnetic resonance imaging (fMRI) is commonly used to study the activity in the auditory cortex in response to auditory stimuli.

Given these explanations, the incorrect statement about the auditory cortex is:

（A）位在大腦枕葉（occipital lobe）的兩側

This statement is incorrect because the auditory cortex is located in the temporal lobes of the brain, not in the occipital lobes.

You

Please answer the following multiple-choice question as a hearing care professional, providing reasoning and explanation: 25 中樞聽覺的活動，可用功能性磁振造影（functional magnetic resonance imaging, fMRI）來觀察，下列敘述何者錯誤?
（A）看血液活動改變　（B）可看神經的工作活動
（C）可指出腦幹不同位置的反應　（D）雖非侵入性檢查，但大多需注射顯影劑

ChatGPT

To address this question, let's analyze each statement in the context of functional magnetic resonance imaging (fMRI) and its application in studying central auditory activity:

1. **Observing Changes in Blood Flow (A)**: This is correct. fMRI works by detecting changes in blood flow and oxygenation within the brain, which are associated with neural activity. When a specific brain region is more active, it consumes more oxygen, leading to an increase in blood flow to that area.
2. **Observing Neural Activity (B)**: While fMRI doesn't directly visualize neurons firing, it measures hemodynamic responses (changes in blood flow and oxygenation) associated with neural activity. Therefore, fMRI can be used to infer neural activity in the brain.
3. **Identifying Responses in Different Parts of the Brainstem (C)**: fMRI can indeed be used to observe activity in various parts of the brain, including the brainstem, although it's worth noting that the spatial resolution of fMRI might limit the granularity with which small structures like those in the brainstem can be observed.
4. **Need for Contrast Agent Injection (D)**: fMRI typically does not require the injection of a contrast agent. It measures brain activity by detecting changes in blood oxygenation (the BOLD - Blood Oxygen Level Dependent - signal), which does not require any external contrast agent. Contrast agents are more commonly used in other types of MRI scans, not in standard functional MRI.

Given these points, the incorrect statement about fMRI is:

（D）雖非侵入性檢查，但大多需注射顯影劑

This statement is incorrect because fMRI usually does not require the injection of a contrast agent to observe brain activity.

You

Please answer the following multiple-choice question as a hearing care professional, providing reasoning and explanation: 26 螺旋神經節細胞（spiral ganglion cells），其軸突有時被稱為聽覺神經纖維，是連結腦幹和耳蝸的構造，大約多少百分比的螺旋神經節神經元與耳蝸的內毛細胞形成突觸?
（A） 90%～95%　（B） 70%～75%　（C） 50%～55%　（D） 30%～35%
27 下列關於初級聽覺神經元（primary auditory neuron）與次級聽覺神經元（secondary auditory neuron） 特徵的敘述，何者正確?
（A）具相同神經元組織型態（neuron morphology）
（B）皆為興奮性反應（excitatory response）
（C）具相同刺激後時間紀錄圖（post-stimulus time histogram）反應種類
（D）皆具有聽覺路徑的音調排列分布（tonotopic organization）

ChatGPT

To address these questions, we need to examine the characteristics of spiral ganglion cells and the distinctions between primary and secondary auditory neurons:

Question 26: Spiral Ganglion Cells Synapses

Spiral ganglion cells are the primary neurons of the auditory pathway, and their axons form the auditory nerve. These cells make synaptic connections with hair cells in the cochlea. The majority of spiral ganglion cells form synapses with inner hair cells. The percentage of these neurons connecting with inner hair cells is quite high, although the exact figure can vary based on the source. Considering the options provided:

- (A) 90%～95%
- (B) 70%～75%
- (C) 50%～55%
- (D) 30%～35%

The most accurate range is **(A) 90%～95%**. This range reflects the predominant innervation of inner hair cells by spiral ganglion cells, as opposed to the fewer connections with outer hair cells.

Question 27: Primary vs. Secondary Auditory Neurons

Primary auditory neurons are the neurons that directly connect the hair cells in the cochlea to the brainstem (such as the spiral ganglion cells), whereas secondary auditory neurons are the ones in the brainstem that receive inputs from these primary neurons.

1. **Same Neuron Morphology (A)**: Primary and secondary auditory neurons do not necessarily share the same neuron morphology. Their structures can be quite different, reflecting their different roles and locations in the auditory pathway.
2. **Excitatory Response (B)**: Both primary and secondary auditory neurons can exhibit excitatory responses, but this is not a distinguishing feature and does not encompass the complexity of their responses.
3. **Same Post-Stimulus Time Histogram Response Types (C)**: Primary and secondary auditory neurons can exhibit different firing patterns and PSTH responses due to their different roles and synaptic inputs.
4. **Tonotopic Organization (D)**: Both primary and secondary auditory neurons maintain a tonotopic organization, meaning they preserve a systematic arrangement according to the frequency of sound. This is a fundamental aspect of the auditory system that is preserved at various levels.

Given these considerations, the correct statement about primary and secondary auditory neurons is:

（D）皆具有聽覺路徑的音調排列分布（tonotopic organization）

This statement correctly identifies that both primary and secondary auditory neurons maintain tonotopic organization, a key feature of auditory processing.

You

Please answer the following multiple-choice question as a hearing care professional, providing reasoning and explanation: 28 小腦橋腦角（cerebellopontine angle, CPA）腫瘤是腦瘤好發位置，此一部位的手術，較不可能傷及下列那個構造?
（A）耳蝸（cochlea）　（B）聽神經（auditory nerve）
（C）耳蝸神經核（cochlear nuclei）　（D）內側膝狀體（medial geniculate body）
29 下列有關振動的敘述，何者最不適當?
（A）任何有慣性（inertia）和彈性（elasticity）的物體都可以振動
（B）自由振動（free vibration）指任何不受外力影響的振動
（C）傅立葉（Joseph Fourier）認為任何振動都可以透過正弦（sinusoidal）振動的總和表示
（D）確定振幅（amplitude）和頻率（frequency）就可以製造出獨一無二的振動

ChatGPT

Let's address each question separately:

Question 28: Cerebellopontine Angle Tumor Surgery

The cerebellopontine angle (CPA) is a region located at the junction of the cerebellum and pons in the brain. Tumors in this area, such as vestibular schwannomas (acoustic neuromas), can affect structures nearby. During surgery in this region, various neural structures could potentially be at risk:

1. **Cochlea (A)**: The cochlea is a part of the inner ear and can be at risk during CPA surgery due to its proximity to the site.
2. **Auditory Nerve (B)**: The auditory nerve, which runs from the cochlea to the brainstem, is also at risk during CPA surgery.
3. **Cochlear Nuclei (C)**: The cochlear nuclei are located in the brainstem and are not in immediate proximity to the cerebellopontine angle. They are less likely to be directly affected by surgery in the CPA region compared to the cochlea and auditory nerve.
4. **Medial Geniculate Body (D)**: The medial geniculate body is part of the thalamus and is located much deeper in the brain, away from the CPA. It is unlikely to be affected by surgery in the CPA region.

Considering these points, the least likely structure to be damaged during surgery in the CPA region is:

（D）內側膝狀體（medial geniculate body）

Question 29: Statements About Vibrations

Let's evaluate the appropriateness of each statement regarding vibrations:

1. **Inertia and Elasticity (A)**: This is a correct statement. Objects that have inertia and elasticity can vibrate.
2. **Free Vibration (B)**: Free vibration indeed refers to vibration that occurs without external forces acting upon the object.
3. **Fourier's Theory (C)**: Joseph Fourier's work established that any complex vibration or waveform can be broken down into a sum of simpler sinusoidal waves. This is the basis for Fourier analysis.
4. **Unique Vibration with Amplitude and Frequency (D)**: While amplitude and frequency are crucial parameters of a vibration, they alone do not define a unique vibration. The same amplitude and frequency can be shared by different waveforms; the phase and waveform shape also play important roles.

Given this analysis, the least appropriate statement about vibrations is:

（D）確定振幅（amplitude）和頻率（frequency）就可以製造出獨一無二的振動

This statement is the least appropriate because amplitude and frequency alone do not guarantee a unique vibration. Other factors like phase and waveform shape also contribute to the uniqueness of a vibration.

You

Please answer the following multiple-choice question as a hearing care professional, providing reasoning and explanation: 30 有關複合音（complex sound）之敘述，下列何者最為適當?
（A）僅透過振幅頻譜（amplitude spectrum）資訊無法創造出獨一無二的聲音
（B）兩個不一樣的聲音可以擁有一樣的波形圖（waveform）
（C）波形圖表現的是頻域（frequency domain）分析的結果
（D）連續頻譜（continuous spectrum）是由基礎頻率（fundamental frequency）及許多諧和頻率（harmonics） 所組成
31 關於最小可聽聲壓（MAP）與最小可聽聲場（MAF）的比較性描述，下列何者正確?
（A） MAP 因為殘存耳道容積在各個頻率的表現皆低於 MAF
（B） MAF 在 90°及 270°測得的結果與 MAP 測得的結果相同
（C） MAF 施測時非測試耳需要遮蔽結果才會與MAP 一致
（D） MAF 在各個頻率的表現皆低於MAP

ChatGPT

Let's address each question separately:

Question 30: Complex Sound

Complex sounds are made up of multiple frequencies, often including a fundamental frequency and its harmonics. Let's evaluate each statement:

1. **Amplitude Spectrum Alone (A)**: This is correct. The amplitude spectrum of a sound shows the amplitudes of the various frequencies present but doesn't provide phase information. Two different sounds can have the same amplitude spectrum but different phase spectra.
2. **Different Sounds, Same Waveform (B)**: This is incorrect. Different sounds cannot have the same waveform. The waveform of a sound is unique and encompasses all its characteristics, including frequency, amplitude, and phase.
3. **Waveform as Frequency Domain Analysis (C)**: This is incorrect. A waveform represents a time domain analysis of a sound. Frequency domain analysis is represented by a spectrum.
4. **Continuous Spectrum (D)**: This is incorrect for complex sounds. A continuous spectrum refers to sounds with energy at a continuous range of frequencies, not just at the fundamental frequency and its harmonics. Complex sounds typically have a line spectrum (if periodic) with discrete frequencies (the fundamental and harmonics).

The most appropriate statement about complex sounds is:

（A）僅透過振幅頻譜（amplitude spectrum）資訊無法創造出獨一無二的聲音

Question 31: MAP vs. MAF

Minimum Audible Pressure (MAP) and Minimum Audible Field (MAF) are two different ways of measuring auditory thresholds:

1. **MAP and Ear Canal Resonance (A)**: This statement is incorrect. MAP, measured with headphones, can be influenced by ear canal resonance, but it doesn't always result in lower thresholds compared to MAF.
2. **MAF at 90° and 270° (B)**: This statement is incorrect. MAF measurements, which are done in a sound field, can vary based on the direction of sound incidence. However, they don't necessarily match MAP measurements at specific angles.
3. **MAF Testing and Ear Occlusion (C)**: This is incorrect. Whether the non-test ear needs to be masked in MAF testing depends on the specific conditions of the test, not on a general rule for consistency with MAP.
4. **MAF Lower than MAP (D)**: This is incorrect. MAF thresholds are generally lower (better) than MAP thresholds at lower frequencies due to the head-related transfer function in a sound field. At higher frequencies, the differences are less pronounced.

Considering these points, none of the provided statements about MAP and MAF accurately describes their comparative characteristics.

You

Give the option you think is most suitable for Q31

ChatGPT

For Question 31, regarding the comparison between Minimum Audible Pressure (MAP) and Minimum Audible Field (MAF), the most suitable option, based on the general principles of auditory testing, would be:

（A） MAP 因為殘存耳道容積在各個頻率的表現皆低於 MAF

This statement suggests that MAP thresholds are typically lower than MAF thresholds due to the effect of the residual volume of the ear canal in MAP testing. In general, MAP thresholds can be influenced by the acoustic properties of the ear canal and headphones used for testing, leading to variations in thresholds across frequencies. MAF, on the other hand, is measured in an open sound field and is less affected by these factors. While this statement might not capture all aspects of the MAP vs. MAF comparison, it is the most suitable among the given options.

You

Please answer the following multiple-choice question as a hearing care professional, providing reasoning and explanation: 32 關於測量聲音暴露量 C 加權（C-weighting）的描述，下列何者正確?
（A）參考 100 phons 等響度取線，與 A 加權在 1000 Hz 相差 60 dB
（B）參考 100 phons 等響度取線，與 A 加權在 1000 Hz 相同
（C）參考 80 phons 等響度取線，與 A 加權在 1000 Hz 相差 40 dB
（D）參考 80 phons 等響度取線，與 A 加權在 1000 Hz 相同

33 一駐波（standing wave）在兩端開口之10 公分管內形成，其基礎振動模式（fundamental mode of vibration）會在下列那一頻率形成? （假設聲速為 350 m/s）
（A） 17.5 Hz　（B） 8.75 Hz　（C） 875 Hz　（D） 1750 Hz

ChatGPT

Let's address each question separately:

Question 32: C-Weighting in Sound Measurement

C-weighting is used in sound level meters for the measurement of sound intensity. It's designed to reflect the frequency sensitivity of the human ear at higher sound levels:

1. **Reference to 100 phons, 60 dB Difference at 1000 Hz (A)**: This is incorrect. The reference to phons is related to equal loudness contours, not directly to C-weighting. The difference in dB between A and C weighting at 1000 Hz is not 60 dB.
2. **Reference to 100 phons, Same at 1000 Hz (B)**: This is correct. Both A and C weightings have the same response at 1000 Hz, which is the reference frequency. They diverge at other frequencies, with A-weighting de-emphasizing lower and higher frequencies more than C-weighting.
3. **Reference to 80 phons, 40 dB Difference at 1000 Hz (C)**: This is incorrect. The reference to 80 phons is not applicable here, and the 40 dB difference at 1000 Hz between A and C weightings is not accurate.
4. **Reference to 80 phons, Same at 1000 Hz (D)**: This is incorrect. The reference to 80 phons is not relevant, and A and C weightings are the same at 1000 Hz, not differing in their response.

The correct answer is:

（B）參考 100 phons 等響度取線，與 A 加權在 1000 Hz 相同

Question 33: Fundamental Mode of Vibration in a 10 cm Open Tube

To calculate the frequency of the fundamental mode of vibration in a tube open at both ends, we use the formula for the fundamental frequency of an open tube, which is $f = \frac{v}{2L}$, where v is the speed of sound and L is the length of the tube.

Given that the speed of sound is 350 m/s and the length of the tube is 10 cm (0.1 m), we can calculate the fundamental frequency as follows:

$f = \frac{350}{2 \times 0.1}$

$f = \frac{350}{0.2}$

$f = 1750\ \mathrm{Hz}$

So, the correct answer is:

（D） 1750 Hz

This frequency represents the fundamental mode of vibration for a standing wave in a 10 cm open tube given the speed of sound as 350 m/s.

You

Please answer the following multiple-choice question as a hearing care professional, providing reasoning and explanation: 34 聲源的聲壓（sound pressure）向四周傳遞時，其能量依何規則來變化?
（A）跟距離成正比　（B）跟距離成反比　（C）跟距離平方成正比　（D）跟距離平方成反比
35 相位（phase）相差為 90 度的 2 個正弦波（sinusoids）相加時，若兩者之頻率與振幅皆相同時，其疊加結果將為何?
（A）結果可互相完全抵消　（B）振幅不變而頻率增加變為 5 倍
（C）頻率不變而振幅增大　（D）振幅變為 1/2 倍

ChatGPT

Let's address each question separately:

Question 34: Sound Pressure Propagation

When sound propagates from a source, its energy changes according to specific rules related to distance:

1. **Proportional to Distance (A)**: Sound pressure does not increase linearly with distance. It actually decreases.
2. **Inversely Proportional to Distance (B)**: This is closer but not quite accurate. Sound pressure decreases with distance, but not strictly in a simple inverse proportion.
3. **Proportional to the Square of the Distance (C)**: This is not correct. Sound energy, not pressure, decreases in proportion to the square of the distance from the source (inverse square law).
4. **Inversely Proportional to the Square of the Distance (D)**: This is correct. The intensity of sound (energy per unit area) decreases with the square of the distance from the source, according to the inverse square law. As intensity is proportional to the square of the sound pressure, the sound pressure itself decreases inversely with the distance.

The correct answer is:

（D）跟距離平方成反比

Question 35: Addition of Sinusoids with 90-Degree Phase Difference

When two sinusoidal waves with the same frequency and amplitude but with a 90-degree phase difference are added, the result is a sinusoid with the same frequency and increased amplitude. The amplitude of the resulting wave can be determined by vector addition. Since the waves are 90 degrees out of phase, the amplitudes add in quadrature (using the Pythagorean theorem).

1. **Complete Cancellation (A)**: This is not correct. Complete cancellation would occur if the waves were 180 degrees out of phase.
2. **Amplitude Unchanged, Frequency Increased (B)**: This is incorrect. The frequency remains the same, and the amplitude increases.
3. **Frequency Unchanged, Amplitude Increased (C)**: This is correct. The amplitude of the resultant wave is increased due to the vector addition of the amplitudes.
4. **Amplitude Halved (D)**: This is not correct. The amplitude increases, not decreases.

The correct answer is:

（C）頻率不變而振幅增大

The resulting wave has the same frequency but an increased amplitude due to the 90-degree phase difference between the two contributing sinusoids.

You

Please answer the following multiple-choice question as a hearing care professional, providing reasoning and explanation: 36 有關聲波的傳導，下列敘述何者錯誤?
（A）介質密度與聲波傳遞速度有關
（B）聲波傳遞速度不會受到介質溫度高低所影響
（C）赤道地區的空氣傳音效果理論上比北極地區的空氣傳音效果更好、更快，原因與溫差有關
（D）在樓下的人，聽到樓上椅子移動的聲音，似乎快於聽到樓上人的呼叫聲，導致這種傳音速度差異的原因，和樓板密度高於空氣密度有關
37 關於聲波與扭曲產物（distortion product）的敘述，下列何者錯誤?
（A）通常在非線性系統（non-linear）會產生
（B）歌唱家的聲音非常圓潤，所以其嗓音[illegible]扭曲產物的發生
（C）扭曲產物耳聲傳射（distortion product otoacoustic emission, DPOAE）是內耳具有扭曲產物特性的證據
（D）有諧振（harmonic）的聲波是該聲波具有扭曲（distortion）的現象

ChatGPT
Let's address each question separately:

Question 36: Sound Wave Propagation

The statement about sound wave propagation that is incorrect:

1. **Medium Density and Sound Speed (A)**: This is correct. The density of the medium affects the speed of sound propagation. For example, sound travels faster in denser mediums like water compared to air.
2. **Sound Speed Unaffected by Medium Temperature (B)**: This is incorrect. The speed of sound is indeed affected by the temperature of the medium. In general, sound travels faster in warmer mediums.
3. **Sound Transmission in Equatorial vs. Polar Regions (C)**: This is correct. In warmer regions like the equator, sound travels faster due to higher air temperatures compared to colder regions like the polar areas.
4. **Sound Transmission Through Floors vs. Air (D)**: This is correct. Sound travels faster through solid mediums like floors (structural borne sound) than through air (airborne sound). Hence, the sound of a chair moving upstairs can be heard quicker than the sound of a voice.

The incorrect statement is:

（B）聲波傳遞速度不會受到介質溫度高低所影響

Question 37: Sound Waves and Distortion Products

The statement about sound waves and distortion products that is incorrect:

1. **Non-linear Systems Producing Distortion (A)**: This is correct. Distortion products typically occur in non-linear systems, such as the inner ear.
2. **Singers' Voices Lacking Distortion Produ[illegible] (B)**: This is incorrect. All sounds, including the human voice, can produce distortion products, especially in a non-linear system like the ear. The quality of a singer's voice doesn't negate the physics of sound.
3. **DPOAE as Evidence of Inner Ear Distortion Products (C)**: This is correct. Distortion product otoacoustic emissions (DPOAEs) are evidence of the inner ear's non-linear response and its ability to generate distortion products.
4. **Harmonic Sound and Distortion (D)**: This is a bit misleading. Harmonics are integral components of musical tones and don't necessarily indicate distortion. Distortion refers to the alteration of the original sound wave, whereas harmonics are natural overtones of sounds.

The incorrect statement is:

（B）歌唱家的聲音非常圓潤，所以其嗓音沒有扭曲產物的發生

All sounds, including the human voice, can potentially generate distortion products in a non-linear system, regardless of the perceived quality of the sound.

You
Please answer the following multiple-choice question as a hearing care professional, providing reasoning and explanation: 38 關於濾波器（filter）的敘述，下列何者正確?
（A）高通（high-pass）濾波器指高於切截頻率（cutoff frequency）的波會被濾掉
（B）切截頻率多在半功率點（half-power point），又稱為 3-dB up point
（C）帶阻（band-reject）濾波器可以中心頻率（center frequency）表示
（D）帶通（band-pass）濾波器的頻寬就是高低兩個切截頻率間的寬度
39 有關等響度曲線（equal loudness contour）的敘述，下列何者正確?
（A）最早關於等響度的研究資料是由學者 Kingsbury 於 1927 年發表，因此現今等響度曲線亦稱為 Kingsbury curves
（B）在 40 phons 曲線上，3 kHz 所需的聲壓值（sound pressure level）會比 1 kHz 所需的聲壓值小
（C）在較高響度（high loudness level）時，phon curves 的曲線型態會比較像 minimum audible field（MAF） curve
（D） phon curves 在較低響度（lower loudness level）時會較趨平坦

ChatGPT
Let's address each question separately:

Question 38: Filters

Let's review each statement about filters:

1. **High-Pass Filter (A)**: This statement is incorrect. A high-pass filter allows frequencies higher than its cutoff frequency to pass through, while attenuating (reducing) frequencies below the cutoff frequency.
2. **Cutoff Frequency at Half-Power Point (B)**: This is correct. The cutoff frequency of a filter is often defined at the half-power point, which is a 3 dB decrease in power from the passband.
3. **Band-Reject Filter and Center Frequency (C)**: This statement is misleading. A band-reject (or notch) filter is characterized by a range of frequencies it attenuates, typically defined by its bandwidth and center frequency. However, it's not accurate to represent it solely by the center frequency, as the bandwidth is also crucial.
4. **Band-Pass Filter Bandwidth (D)**: This is correct. The bandwidth of a band-pass filter is defined as the difference between its upper and lower cutoff frequencies.

The correct answer is:

（D）帶通（band-pass）濾波器的頻寬就[illegible]低兩個切截頻率間的寬度

Question 39: Equal Loudness Contours

Now, let's examine each statement about equal loudness contours:

1. **Kingsbury's Research (A)**: This statement is incorrect. The most well-known early research on equal loudness contours was conducted by Fletcher and Munson, not Kingsbury.
2. **40 Phons Curve at 3 kHz vs. 1 kHz (B)**: This is correct. At 3 kHz, the ear is more sensitive than at 1 kHz. Therefore, at 40 phons, a lower sound pressure level is required at 3 kHz compared to 1 kHz for the sounds to be perceived as equally loud.
3. **High Loudness Level Phon Curves (C)**: This is somewhat misleading. While phon curves at higher loudness levels do flatten out, comparing them directly to MAF curves is not typically how they are characterized.
4. **Flat Phon Curves at Lower Loudness Levels (D)**: This is incorrect. At lower loudness levels, the curves tend to show more variation, especially at lower and higher frequencies, where the ear is less sensitive.

The correct answer is:

（B）在 40 phons 曲線上，3 kHz 所需的聲壓值（sound pressure level）會比 1 kHz 所需的聲壓值小

You
Please answer the following multiple-choice question as a hearing care professional, providing reasoning and explanation: 40 依據時間加成（temporal summation），某人對一 200 毫秒的純音測得的閾值為 25 dB HL，如果將同一純音的時長縮短到 20 毫秒，則此人的閾值為何?
（A） 25 dB HL （B） 30 dB HL （C） 35 dB HL （D） 40 dB HL
41 下列何種心理聲學測量方法屬於直接量測（direct scaling）?
（A） single up-down method
（B） Bekesy tracking method
（C） parameter estimation by sequential testing（PEST）
（D） ratio estimation and production

ChatGPT
Let's address each question separately:

Question 40: Temporal Summation

Temporal summation in hearing refers to the fact that the perceived loudness of a sound increases with its duration, up to a certain point (around 200 ms). For durations shorter than this, the sound needs to be louder to be perceived as equally loud.

Given a threshold of 25 dB HL for a 200 ms pure tone, if the duration is reduced to 20 ms, the sound will need to be louder to be perceived. However, the exact increase in dB HL required can vary and may not strictly follow a linear or standardized scale. In general, though, a significant increase in dB HL would be expected.

Considering the options, a reasonable estimate would be:

（C） 35 dB HL

This choice reflects a significant increase in threshold due to the shorter duration but doesn't overestimate the change.

Question 41: Psychophysical Measurement Methods

Now, let's consider the psychophysical measurement methods:

1. **Single Up-Down Method**: This is a method used in psychophysical testing to determine thresholds, but it's not a direct scaling method. It's an adaptive testing technique.
2. **Bekesy Tracking Method**: This is an automatic method of auditory testing, particularly for determining thresholds, but it is not a direct scaling method.
3. **PEST (Parameter Estimation by Sequential Testing)**: Like the single up-down method, PEST is an adaptive method for threshold estimation and not a direct scaling method.
4. **Ratio Estimation and Production**: This involves direct scaling, where subjects are asked to estimate or produce ratios of stimuli (e.g., twice as loud, half as bright). This method directly involves the participant in quantifying their perception relative to a given standard or another stimulus.

The correct answer for a direct scaling method is:

（D） ratio estimation and production

This method involves direct participant input to scale their perception, making it a direct scaling method in psychophysical measurements.

You
Please answer the following multiple-choice question as a hearing care professional, providing reasoning and explanation: 42 純音聽力檢查中氣導檢測時實施遮蔽，關於過度遮蔽（overmasking）的敘述，下列何者正確?
（A）當非測試耳之遮蔽音音強（masking level）大於測試耳骨導閾值及耳間衰減（interaural attenuation） 值之加總時，就會發生過度遮蔽
（B）當非測試耳之遮蔽音音強大於雙耳骨導閾值之加總時，就會發生過度遮蔽
（C）當非測試耳之遮蔽音音強大於測試耳骨導閾值及耳間衰減值之相減值時，就會發生過度遮蔽
（D）當非測試耳之遮蔽音音強大於雙耳骨導閾值之相減值時，就會發生過度遮蔽
43 關於時序遮蔽（temporal masking）的敘述，下列何者錯誤?
（A）前向邊緣（forward fringe）遮蔽效果大於後向邊緣（backward fringe）遮蔽效果
（B）當訊號在遮蔽音之後出現，此種情況稱作前向遮蔽（forward masking），當兩個刺激訊號間隔 200 毫秒時，仍會發生前向遮蔽
（C）當訊號在遮蔽音之前出現，此種情況稱作後向遮蔽（backward masking），當兩個刺激訊號間隔 50
毫秒時，仍會發生後向遮蔽
（D）前向遮蔽效果比後向遮蔽效果好

ChatGPT
Let's address each question separately:

Question 42: Overmasking in Pure Tone Audiometry

Overmasking occurs when the masking noise in the non-test ear is too loud, potentially crossing over to the test ear and masking the test signal. Let's evaluate the options:

1. **Masking Level Greater Than Test Ear Bone Conduction Threshold Plus Interaural Attenuation (A)**: This is correct. Overmasking happens when the masking level in the non-test ear exceeds the sum of the bone conduction threshold of the test ear and the interaural attenuation. Interaural attenuation is the amount of sound energy lost as sound travels from the masked ear to the test ear.
2. **Masking Level Greater Than Sum of Both Ears' Bone Conduction Thresholds (B)**: This is incorrect. Overmasking is not determined by the sum of both ears' bone conduction thresholds.
3. **Masking Level Greater Than Test Ear Bone Conduction Threshold Minus Interaural Attenuation (C)**: This is incorrect. Overmasking is related to the sum, not the difference, between the bone conduction threshold and interaural attenuation.
4. **Masking Level Greater Than Difference Between Both Ears' Bone Conduction Thresholds (D)**: This is incorrect. The consideration for overmasking involves the sum of the test ear's bone conduction threshold and interaural attenuation, not the difference in thresholds between the two ears.

The correct answer is:

（A）當非測試耳之遮蔽音音強（masking level）大於測試耳骨導閾值及耳間衰減（interaural attenuation） 值之加總時，就會發生過度遮蔽

Question 43: Temporal Masking

Temporal masking refers to the masking o[illegible] sound signal by another sound signal that occurs either before or after it in time. Let's examine the options:

1. **Forward Fringe Greater Than Backward Fringe (A)**: This statement might be context-dependent. Generally, backward masking (where the masker follows the signal) can be more effective than forward masking (where the masker precedes the signal).
2. **Forward Masking with 200 ms Gap (B)**: This is correct. Forward masking can occur when the signal appears after the masking noise, even with a gap as long as 200 ms.
3. **Backward Masking with 50 ms Gap (C)**: This is correct. Backward masking occurs when the signal precedes the masking noise, and it can occur even with a short gap like 50 ms.
4. **Forward Masking More Effective Than Backward Masking (D)**: This is typically incorrect. Backward masking is often found to be more effective than forward masking.

Given these explanations, the incorrect statement about temporal masking is:

（D）前向遮蔽效果比後向遮蔽效果好

You
Please answer the following multiple-choice question as a hearing care professional, providing reasoning and explanation: 44 關於高音頻音源的定位（localization），最主要依賴下列那一線索來定位?
（A） interaural time difference （B） spectral difference of sound
（C） interaural level difference （D） interaural phase difference
45 下列關於閉塞效應（occlusion effect）的敘述，何者錯誤?
（A）閉塞效應在低頻率時較大
（B）只有在外耳道骨質部（bony portion）的部分被閉塞之後，才會產生閉塞效應
（C）雙耳傳導型聽覺障礙的個案，不會出現閉塞效應的現象
（D）臨床上的音叉測驗 Bing test，就是利用閉塞效應的原理做判斷

ChatGPT
Let's address each question separately:

Question 44: Localization of High-Frequency Sound Sources

The localization of high-frequency sound so[illegible]rces relies on certain auditory cues:

1. **Interaural Time Difference (A)**: This is more important for low-frequency sounds. High-frequency sounds have shorter wavelengths, making it difficult for the human auditory system to use time differences effectively.
2. **Spectral Difference of Sound (B)**: While spectral cues can be helpful, especially in complex listening environments, they are not the primary cue for high-frequency sound localization.
3. **Interaural Level Difference (ILD) (C)**: This is the primary cue for localizing high-frequency sounds. High-frequency sounds have shorter wavelengths and are more easily shadowed by the head, creating level differences between the ears that the auditory system uses for localization.
4. **Interaural Phase Difference (D)**: Phase differences are more useful for low-frequency sounds due to their longer wavelengths.

The most reliable cue for localizing high-frequency sound sources is:

（C） interaural level difference

Question 45: Occlusion Effect

The occlusion effect refers to the increase [illegible]ow-frequency sounds perceived by a person when the ear canal is blocked. Let's examine the options:

1. **Greater at Low Frequencies (A)**: This is correct. The occlusion effect is more pronounced at low frequencies.
2. **Occurs Only When the Bony Portion of the Ear Canal Is Occluded (B)**: This is incorrect. The occlusion effect can occur when any part of the ear canal is blocked, not just the bony portion.
3. **Not Present in Individuals with Conductive Hearing Loss in Both Ears (C)**: This is correct. Individuals with conductive hearing loss in both ears typically do not experience the occlusion effect because sound is not effectively transmitted through the outer or middle ear.
4. **Bing Test Based on the Occlusion Effect (D)**: This is correct. The Bing test is a clinical tuning fork test that utilizes the principles of the occlusion effect to assess conductive hearing loss.

The incorrect statement about the occlusion effect is:

（B）只有在外耳道骨質部（bony portion）的部分被閉塞之後，才會產生閉塞效應

You
Please answer the following multiple-choice question as a hearing care professional, providing reasoning and explanation: 46 下列何種情形可以察覺消失的基頻（missing fundamental），同時暗示此一機轉發生於中樞聽覺系統?
（A）兩諧音同時放至同一耳 （B）兩諧音間隔 50 ms 放至同一耳
（C）兩諧音同時放至雙耳 （D）兩諧音間隔 50 ms 放至雙耳
47 聲波（sound wave）的參數中的頻率（frequency），對應於人體感官的：
（A）響度（loudness） （B）音調（pitch） （C）節奏（rhythm） （D）音質（timbre）

ChatGPT
Let's address each question separately:

Question 46: Perception of the Missing Fundamental

The phenomenon of the missing fundamental refers to the auditory system's ability to perceive the pitch of a tone that corresponds to a fundamental frequency, even when this frequency is not physically present in the sound. This effect is due to the brain's processing rather than the ear's mechanics. Let's evaluate the options:

1. **Two Harmonics Simultaneously to One Ear (A)**: This could allow for the perception of the missing fundamental, as the brain can process the harmonics to perceive the pitch of the fundamental frequency even if it's not physically present.
2. **Two Harmonics with 50 ms Gap to One Ear (B)**: The gap between the harmonics is less relevant; it's the combination of harmonics that allows for the perception of the missing fundamental.
3. **Two Harmonics Simultaneously to Both Ears (C)**: Similar to option A, this would also allow for the perception of the missing fundamental. The auditory system combines the harmonics to infer the pitch of the fundamental frequency.
4. **Two Harmonics with 50 ms Gap to Both Ears (D)**: Again, the gap is less relevant. The key is the combination of harmonics.

The perception of the missing fundamental occurs when harmonics are presented either to one ear or both ears simultaneously. The options A and C both describe scenarios that could lead to the perception of the missing fundamental. However, the question emphasizes the central auditory mechanism, which is more general and not specific to one ear or both. Thus:

（C）兩諧音同時放至雙耳

This option implies a binaural process, highlighting the central auditory system's role.

Question 47: Sound Wave Frequency and Sensory Perception

Frequency is a fundamental characteristic of a sound wave and is directly related to how the human auditory system perceives sound. Let's look at the options:

1. **Loudness (A)**: Loudness is related to the amplitude of the sound wave, not its frequency.
2. **Pitch (B)**: This is correct. Pitch is the perceptual counterpart to the physical parameter of frequency. Higher frequencies are perceived as higher pitches.
3. **Rhythm (C)**: Rhythm is related to the temporal pattern of sound, not its frequency.
4. **Timbre (D)**: Timbre, or the quality of sound, is related to the waveform, including the frequency spectrum and overtones, but it's not directly the perception of frequency itself.

The correct answer is:

（B）音調（pitch）

Pitch perception is directly related to the frequency of the sound wave.

You
Please answer the following multiple-choice question as a hearing care professional, providing reasoning and explanation: 48 由等響度曲線（equal loudness contour）觀察，可知人耳對何種頻率的敏感度較佳?
（A）中高頻率（1000 Hz ~ 4000 Hz） （B）低頻率（500 Hz 以下）
（C）聽覺領域的頻率均相同 （D）中低頻率（500 Hz ~ 1000 Hz）
49 根據鼓室圖（tympanogram）測驗結果所畫出之圖形，若出現 2 個最高聽能順應力點（peak of compliance），而呈現倒 W 型，臨床上最常代表的意義為何?
（A）可能耳垢塞滿了外耳道
（B）可能同時有 2 種中耳疾病
（C）可能耳膜有一個破洞已經癒合（healed perforation of eardrum）
（D）中耳積水

ChatGPT
Let's address each question separately:

Question 48: Sensitivity of the Human Ear to Different Frequencies

Equal loudness contours show how loudness perception varies across different frequencies at a given sound pressure level. From these curves, we can understand which frequency ranges the human ear is most sensitive to. The options are:

1. **Mid-High Frequencies (1000 Hz to 4000 Hz) (A)**: This is correct. Human hearing is most sensitive in the mid to high-frequency range, particularly around 2000-4000 Hz. Equal loudness contours show that lower sound pressure levels are needed for these frequencies to be perceived as equally loud compared to others.
2. **Low Frequencies (Below 500 Hz) (B)**: Human hearing is less sensitive to low frequencies; higher sound pressure levels are required for these frequencies to be perceived as equally loud.
3. **Uniform Sensitivity Across Auditory Range (C)**: This is incorrect. Sensitivity varies across different frequencies.
4. **Mid-Low Frequencies (500 Hz to 1000 Hz) (D)**: While the human ear is quite sensitive in this range, it is not the most sensitive compar[illegible] the mid-high frequencies.

The correct answer is:

（A）中高頻率（1000 Hz ~ 4000 Hz）

Question 49: Interpretation of Tympanogram with Two Peaks of Compliance

A tympanogram is a graph that represents the compliance (flexibility) of the eardrum as air pressure in the ear canal is varied. The appearance of two peaks of compliance can indicate various conditions:

1. **Earwax Blocking the External Ear Canal (A)**: This typically results in a flat tympanogram due to the impedance of sound transmission, not two peaks of compliance.
2. **Two Different Middle Ear Diseases (B)**: While middle ear pathologies can affect tympanogram results, having two distinct peaks of compliance does not typically indicate two separate diseases.
3. **Healed Perforation of Eardrum (C)**: This is the most likely explanation. A tympanogram with two peaks of compliance, especially a "double[illegible]aked" or "W-shaped" tympanogram, can indicate a healed perforation or a scar on the eardrum.
4. **Fluid in the Middle Ear (D)**: Middle ear effusion usually results in a flat tympanogram due to the reduced mobility of the eardrum.

The correct answer is:

（C）可能耳膜有一個破洞已經癒合（healed perforation of eardrum）

This is the most common clinical interpretation of a tympanogram with two peaks of compliance.

You
Please answer the following multiple-choice question as a hearing care professional, providing reasoning and explanation: 50 純音聽力檢查時，使用插入式（insert）耳機與覆耳式（supra-aural）耳機所測得的閾值有所差異，主要源自於下列何種效應產生的內部噪音（internal noise）?
（A）閉塞（occlusion） （B）適應（adaptation） （C）滯後（lagging） （D）漣漪（ripple）

ChatGPT
When comparing thresholds measured using insert earphones versus supra-aural earphones in pure tone audiometry, the differences can be attributed to several factors, including the occlusion effect, which is relevant to the internal noise generated in the ear canal. Let's evaluate the options:

1. **Occlusion (A)**: The occlusion effect is particularly relevant when comparing insert earphones with supra-aural earphones. Insert earphones, which are placed deeper into the ear canal, can cause a significant occlusion effect, especially at lower frequencies. This effect can elevate the low-frequency thresholds because the occlusion of the ear canal can enhance the perception of bone-conducted sounds, particularly those generated by the body, like blood flow or muscle movements.
2. **Adaptation (B)**: While adaptation (a reduction in response over time to a constant stimulus) is a phenomenon in auditory perception, it doesn't explain the difference in thresholds between insert and supra-aural earphones.
3. **Lagging (C)**: Lagging or latency effects are not typically used to describe differences in threshold measurements between these two types of earphones.
4. **Ripple (D)**: Ripple effects refer to variations in frequency response, not specifically relevant to the type of earphone used in audiometry.

The most plausible explanation for the difference in measured thresholds due to internal noise between insert and supra-aural earphones is the occlusion effect. Insert earphones are more likely to cause occlusion of the ear canal, which can affect the perception of low-frequency sounds.

Therefore, the correct answer is:

（A）閉塞（occlusion）

Message ChatGPT...

ChatGPT can make mistakes. Consider checking important information.
