# Supplementary material for: Exploring the Performance of ChatGPT-4 in the Taiwan Audiologist Qualification Examination: Preliminary Observational Study Highlighting the Potential of AI Chatbots in Hearing Care
Source: JMIR Med Educ. 2024 Apr 26;10:e55595. doi: 10.2196/55595 (PMC11067446; doi:10.2196/55595)
Supplement: Multimedia Appendix 2 [file mededu-v10-e55595-s002.pdf]

You
Please answer the following multiple-choice question as a hearing care professional, providing reasoning and explanation (please using English to reply): 1 關於嬰幼兒聽力評估的個案史內容，下列敘述何者最不適當?
（A）多半由嬰幼兒的家長或主要照顧者提供個案史的訊息
（B）調查孕期及產程的危險因子是為了解聽力損失的原因
（C）嬰幼兒的溝通模式通常不在個案史的詢問範圍裡
（D）了解嬰幼兒的個案史可以協助聽力師選擇測試的方式

ChatGPT
在回答這個關於嬰幼兒聽力評估的個案史內容的問題時，我會從聽力保健專業的角度來分析每一個選項：

（A）多半由嬰幼兒的家長或主要照顧者提供個案史的訊息：這個選項是適當的。嬰幼兒無法自己表達，所以家長或主要照顧者提供的信息對於了解嬰幼兒的健康狀況至關重要。

（B）調查孕期及產程的危險因子是為了解聽力損失的原因：這也是一個合理的做法。孕期和產程中的一些危險因素，比如感染、藥物暴露[illegible]生時的低體重等，可能會對嬰幼兒的聽力造成影響。

（C）嬰幼兒的溝通模式通常不在個案史的詢問範圍裡：這個選項是不適當的。了解嬰幼兒的溝通模式是非常重要的，因為它可以提供關於他們聽力狀態的重要線索。例如，如果一個嬰兒對聲音的反應較少，或是在語言發展上有遲緩，這可能是聽力問題的跡象。

（D）了解嬰幼兒的個案史可以協助聽力師選擇測試的方式：這是正確的。了解個案的詳細背景資料可以幫助聽力專業人員選擇最合適的評估方法和工具。

綜上所述，選項（C）「嬰幼兒的溝通模式通常不在個案史的詢問範圍裡」是最不適當的敘述。

You
I hope you give your answer in English.

ChatGPT
Certainly, let's analyze each option in English regarding the content of case history for the hearing assessment of infants and toddlers:

(A) The case history information is mostly provided by the infant's or toddler's parents or primary caregivers: This option is appropriate. Since infants and toddlers cannot express themselves, the information provided by parents or primary caregivers is crucial for understanding the child's health status.

(B) Investigating risk factors during pregnancy and childbirth to understand the causes of hearing loss: This is also a reasonable approach. Some risk factors during pregnancy and childbirth, such as infections, exposure to certain medications, or low birth weight, can affect an infant's hearing.

(C) The communication patterns of infants and toddlers are usually not included in the case history inquiries: This option is inappropriate. Understanding the communication patterns of infants and toddlers is vital because it can provide important clues about their hearing status. For instance, if an infant shows reduced reactions to sounds or has delayed language development, this might indicate a hearing problem.

(D) Understanding the case history of an infant or toddler can assist the audiologist in choosing the method of testing: This is correct. Know[illegible]g the detailed background of the case can help hearing professionals select the most suita[illegible] assessment methods and tools.

Based on the above analysis, option (C) "The communication patterns of infants and toddlers are usually not included in the case history inquiries" is the most inappropriate statement.

You
Please answer the following multiple-choice question as a hearing care professional, providing reasoning and explanation: 2　下列何者是聽力師在進行行為聽力學評估前蒐集個案史，最主要的目的?
（A）通過個案史訪談過程，了解個案和個案的重要他人之間的互動困難
（B）通過訪談，觀察個案的行為表現，以確認個案是否為非器質性聽損
（C）根據個案的回答，建構可能的聽力障礙，並與後續的聽檢結果相互驗證
（D）根據個案的回答，快速找出個案主訴的問題，並立即解答個案問題

ChatGPT
As a hearing care professional, let's analyze each option to determine the primary purpose of collecting case history before conducting [illegible]havioral audiological assessment:

(A) Through the case history interview process, understand the difficulties in interaction between the case and significant others: While understanding interpersonal interactions can provide context, it is not the primary goal of collecting case history in the context of a behavioral audiological assessment. This information may be more relevant to broader aspects of therapy and intervention, but it's not central to the audiological evaluation itself.

(B) Through the interview, observe the case's behavioral performance to confirm whether the hearing loss is non-organic: Observing behavioral performance can be a part of the assessment, particularly in ruling out non-organic (psychogenic) hearing loss. However, this is typically more specific and might not be the primary goal of a general case history collection, which is broader in scope.

(C) Based on the case's responses, construct potential hearing impairments and verify them with subsequent hearing test results: This option aligns closely with the main purpose of collecting case history in audiological assessments. The information gathered helps to form hypotheses about possible hearing issues, which can then be tested and either confirmed or revised based on the results of the hearing tests.

(D) Based on the case's responses, quickly identify the main complaint of the case and immediately answer the case's questions: While addressing the patient's concerns is important, the primary objective of case history collection is not immediate problem-solving or answering questions. It's more about gathering comprehensive information to guide the assessment and intervention planning.

Considering these analyses, the most suitable option is (C) "Based on the case's responses, construct potential hearing impairments and verify them with subsequent hearing test results." This option best reflects the primary goal of collecting case history in behavioral audiological assessments, which is to formulate hypotheses about the patient's hearing condition that can be tested and refined through further evaluation.

You
Please answer the following multiple-choice question as a hearing care professional, providing reasoning and explanation: 3　一位在工廠工作 18 年，聽力不好之個案，下列陳述的病史中，何者有較高的耳蝸後病變（retrocochlear patho[illegible]）懷疑指數?
（A）左耳較右耳差，且持續性耳鳴，偶有眩暈　（B）對於較大聲的關門聲會極度不舒適，無法忍受
（C）耳朵容易有悶脹感，需經常捏鼻子鼓氣　（D）左耳較差且近 2 週有耳痛和耳鳴的現象
4 關於耳垢栓塞（impacted cerumen）的敘述，下列何者最不適當?
（A）常引起傳導性聽損
（B）會有搔癢感、耳鳴及外耳炎
（C）當整個耳道被堵住時，會出現約 55 分貝的聽損
（D）淋浴時，可能會突然聽力下降

ChatGPT
To address these questions, let's analyze each option from the perspective of a hearing care professional:

Question 3

A case of a person who has worked in a factory for 18 years and has hearing difficulties. Which statement in their medical history suggests a higher suspicion index for retrocochlear pathology?

(A) Worse hearing in the left ear than in the [illegible]ight, with persistent tinnitus and occasional vertigo: This option indicates asymmetrical hearing loss combined with tinnitus and vertigo, which can be suggestive of a retrocochlear pathology, such as an acoustic neuroma or other issues affecting the auditory nerve or brainstem.

(B) Extreme discomfort with louder noises such as door slamming, intolerable: This describes hyperacusis, which can be associated with a variety of auditory disorders but is not specifically indicative of retrocochlear pathology.

(C) A feeling of fullness in the ears, often needing to pop ears by pinching the nose: This is more indicative of Eustachian tube dysfunction or middle ear issues, not retrocochlear pathology.

(D) The left ear is worse and there have been symptoms of ear pain and tinnitus for the past 2 weeks: Ear pain combined with tinnitus could be due to a number of causes, including infection, but it doesn't specifically suggest retrocochlear pathology.

The most indicative of retrocochlear pathology in these options is (A) because of the asymmetry in hearing loss, persistent tinnitus, and ver[illegible] which are more aligned with retrocochlear disorders.

Question 4

Regarding impacted cerumen, which statement is the most inappropriate?

(A) Often causes conductive hearing loss: This is true. Impacted cerumen can block the ear canal and cause conductive hearing loss.

(B) Can cause itching, tinnitus, and otitis externa: These are also common symptoms associated with impacted cerumen.

(C) When the entire ear canal is blocked, a hearing loss of about 55 dB can occur: This statement might be exaggerated. While significant hearing loss can occur with complete occlusion, 55 dB is quite specific and may not apply to all cases.

(D) Hearing loss may suddenly worsen during a shower: This can happen if water causes the cerumen to swell, further blocking the ear canal.

The most inappropriate statement seems to be (C) due to the specificity of the 55 dB hearing loss claim, which is not a universally applicable figure for all cases of ear canal blockage due to cerumen impaction.

You
Please answer the following multiple-choice question as a hearing care professional, providing reasoning and explanation: 5　關於純音聽力評估的再測信度（test-retest reliability），下列敘述何者錯誤?
（A）會受個案的決策過程、生理或身體噪音、反應標準等因素影響
（B）耳機擺放位置不影響施測結果
（C）儀器校正會影響施測結果
（D）任一耳的 2k、3k、4k Hz 聽力閾值變化≥10 dB 可當作判斷標準
6 根據 PTA 的檢查結果，無法得知下列何項聽覺表現?
（A）兩耳對各測試頻率的聽覺敏感度（B）可區辨傳導型或感音神經性聽損
（C）可以提供輔具選配的參考　（D）可以確認受測者的溝通能力

ChatGPT
Let's analyze each option for these two multiple-choice questions:

Question 5: About the test-retest reliability of pure tone audiometry, which statement is incorrect?

(A) Influenced by the subject's decision-making process, physiological or body noises, response standards, etc.: This statement is correct. The test-retest reliability of pure tone audiometry can indeed be affected by these factors.

(B) The positioning of the headphones does not affect the test results: This statement is incorrect. The positioning of the headphones can significantly impact the test results. Poorly placed headphones can lead to inaccurate thresholds, which will affect test-retest reliability.

(C) Instrument calibration affects the test results: This is true. Proper calibration of audiometric equipment is essential for accurate hearing assessments.

(D) A change in hearing thresholds at 2k, 3k, 4k Hz of ≥10 dB in any ear can be used as a criterion for judgment: This is a general guideline us[illegible] in audiometry to indicate significant changes in hearing thresholds, so it's a correct statement.

The incorrect statement here is (B) "The positioning of the headphones does not affect the test results."

Question 6: According to the results of PTA (Pure Tone Audiometry), which auditory performance cannot be known?

(A) The auditory sensitivity of both ears to each test frequency: PTA does provide this information, so this statement is incorrect in the context of the question.

(B) Differentiation between conductive and sensorineural hearing loss: PTA can help differentiate these types of hearing loss based on air and bone conduction results, so this statement is also incorrect here.

(C) Provides reference for hearing aid fitting: This is true. PTA results are used to determine the degree and configuration of hearing loss, which is essential for hearing aid selection.

(D) Confirmation of the testee's communic[illegible] ability: This is the correct answer. PTA measures auditory sensitivity at certain frequencies and does not directly assess the individual's overall communicative ability, which includes understanding speech in various listening environments.

So, the performance that cannot be known from PTA results is (D) "Confirmation of the testee's communicative ability."

You
Please answer the following multiple-choice question as a hearing care professional, providing reasoning and explanation: 7　下列何種玩具最適合用於視覺增強檢查時，吸引嬰幼兒目光維持在正前方?
（A）滾珠台（B）動物手指偶　（C）遙控小汽車　（D）小豬學習撲滿
8 聽力檢查時，病患到底要面對還是背對檢查者，具有爭議。針對支持病患面對檢查者的理由，下列何者錯誤?
（A）病患能隨時觀察檢查者的信息，避免漏掉任何提示線索
（B）有些檢查項目，檢查者能夠觀察病患的臉跟口型，避免誤判
（C）檢查者可採遮口的方式，避免透露太多訊息
（D）檢查者只要掌握並控制好檢查技巧，面對病患其實是不錯的方式

ChatGPT
Let's analyze each option for these two multiple-choice questions:

Question 7: Which of the following toys is most suitable for use in a visual reinforcement audiometry (VRA) test to attract and maintain the attention of infants and toddlers directly in front?

(A) Marble Run: While engaging, a marble run might be too complex and could distract the child's gaze in various directions as they follow the movement of the marbles.

(B) Animal Finger Puppets: These can be very effective in capturing and holding a young child's attention. They can be easily manipulated to keep the child's focus straight ahead.

(C) Remote Control Car: This could attract a child's attention, but its movement might lead the child's gaze away from the front, which is not ideal for a VRA test.

(D) Piggy Bank Learning Toy: While educat[illegible]l, it might not be as effective in maintaining a child's attention directly in front, especially for very young children.

The most suitable option for maintaining a child's gaze directly in front during a VRA test is (B) "Animal Finger Puppets," as they are visually appealing and can be used effectively to focus the child's attention straight ahead.

Question 8: Regarding the argument for patients facing the examiner during a hearing test, which of the following reasons is incorrect?

(A) The patient can observe the examiner's cues at all times, avoiding missing any hints: This statement is somewhat controversial as ideally, the patient should not be getting any visual cues from the examiner during a pure tone audiometry test.

(B) For some test items, the examiner can observe the patient's face and mouth movements to avoid misjudgment: This is correct, especially in speech audiometry where visual cues can be important.

(C) The examiner can use a covered mouth t[illegible]chnique to avoid revealing too much information: This is a technique used to prevent lip-rea[illegible]g in speech audiometry, which is a valid point.

(D) As long as the examiner controls and masters the testing technique, facing the patient is actually a good method: This statement is too general and doesn't address specific reasons why facing the patient might be advantageous in certain types of hearing tests.

The incorrect statement here is (A) "The patient can observe the examiner's cues at all times, avoiding missing any hints," as in many types of hearing tests, especially pure tone audiometry, visual cues from the examiner should be minimized to ensure accurate results.

You
Please answer the following multiple-choice question as a hearing care professional, providing reasoning and explanation: 9　有關噪音性聽損之敘述，下列何者錯誤?
（A）暫時閾移（temporary threshold shift, TTS）是一種暴露在高音量後，所出現的暫時聽覺敏感度變化
（B）低頻噪音暴露所造成的閾移會比高頻噪音暴露時還要來得大
（C）最大的暫時閾移會出現在所侵犯的噪音頻帶附近再多出半個八音帶的頻率處
（D）等效靜音（equivalent quiet）表示[illegible]音量不會造成暫時閾移
10　臨床上閉塞效應（occlusion effect）以[illegible]個頻率最明顯?
（A） 250 Hz　（B） 500 Hz　（C） 1000 Hz （D） 2000 Hz

ChatGPT
Let's analyze each option for these two multiple-choice questions:

Question 9: Concerning noise-induced hearing loss, which statement is incorrect?

(A) Temporary Threshold Shift (TTS) is a temporary change in auditory sensitivity after exposure to loud sounds: This statement is correct. TTS refers to a temporary reduction in hearing sensitivity following exposure to loud noise.

(B) Threshold shift caused by low-frequency noise exposure is greater than that caused by high-frequency noise exposure: This statement is incorrect. Typically, noise-induced hearing loss first affects the higher frequencies, particularly around 3000-6000 Hz. High-frequency noise exposure usually results in a greater thresh[illegible]d shift.

(C) The greatest temporary threshold shift occurs at frequencies near the noise band plus half an octave: This is correct. TTS often affects frequencies at and around the frequency of the noise exposure.

(D) Equivalent quiet means the exposure level will not cause a temporary threshold shift: This is a correct statement. "Equivalent quiet" refers to a sound level that is not expected to cause hearing damage.

The incorrect statement is (B) "Threshold shift caused by low-frequency noise exposure is greater than that caused by high-frequency noise exposure."

Question 10: Clinically, at which frequency is the occlusion effect most prominent?

(A) 250 Hz
(B) 500 Hz
(C) 1000 Hz
(D) 2000 Hz

The occlusion effect is most prominent at lower frequencies. It is the sensation of increased loudness of a person's own voice that occurs when the ear canal is blocked. This effect is typically most noticeable at frequencies below 1000 Hz.

Therefore, the correct answer is (A) 250 Hz, as it is within the low-frequency range where the occlusion effect is most noticeable.

You
Please answer the following multiple-choice question as a hearing care professional, providing reasoning and explanation: 11　頭戴式耳機所導致的外耳道塌陷時，下列敘述何者錯誤?
（A）很明顯在低頻處呈現氣骨導差　（B）受試者的再測信度低
（C）此一外耳道塌陷狀況易導致誤診　（D）較常出現在年紀大的受試者
12　下列對遮蔽的敘述，何者錯誤?
（A）過度遮蔽是因為以耳機覆蓋非測試耳時，可能出現閉塞效應，因而增加非測試耳的骨導信號音量
（B）如果在給予 70 分貝，就會過度遮蔽的狀況下，最大遮蔽級則約為 65 分貝
（C）耳間衰減量變小，並不會使遮蔽的高[illegible]變短
（D）當高原期越寬，對遮蔽閾值的效度就可以越確定

ChatGPT
Let's analyze each option for these two multiple-choice questions:

Question 11: Concerning the collapse of the external auditory canal caused by headphones, which statement is incorrect?

(A) A noticeable air-bone gap at low frequencies: This is correct. When the external auditory canal collapses, it can affect the transmission of sound through the ear canal, leading to an apparent air-bone gap, especially at low frequencies.

(B) Low test-retest reliability in subjects: This is also correct. The variability introduced by the collapse can lead to inconsistencies in test results, affecting reliability.

(C) This condition of the external auditory canal collapse can lead to misdiagnosis: This is true. The collapse can mimic or exaggerate cert[illegible] hearing loss patterns, potentially leading to incorrect diagnoses.

(D) More common in older test subjects: This statement is incorrect. External auditory canal collapse due to headphone pressure is not necessarily more common in older individuals. It can occur in any individual depending on the shape and resilience of their ear canal, but it's not specifically age-related.

The incorrect statement is (D) "More common in older test subjects."

Question 12: Regarding masking, which statement is incorrect?

(A) Over-masking occurs when covering the non-test ear with headphones may increase the bone conduction signal volume in the non-test ear due to the occlusion effect: This is a correct explanation of over-masking.

(B) If over-masking occurs at 70 dB, then the maximum masking level would be about 65 dB: This statement is incorrect. If over-masking is occurring at 70 dB, it means that the level of masking noise is too high, potentially crossing over to the test ear. The maximum effective masking level would be determined based on interaural [illegible]uation and the air-bone gap, not just a fixed value below the over-masking level.

(C) Reducing interaural attenuation does not shorten the plateau period of masking: This is correct. The plateau period is the range of masking levels where the threshold of the test ear remains constant. Changes in interaural attenuation do not typically alter this plateau.

(D) The wider the plateau period, the more certain the validity of the masking threshold: This is true. A wide plateau indicates a consistent threshold despite changes in masking level, supporting the validity of the threshold obtained.

The incorrect statement is (B) "If over-masking occurs at 70 dB, then the maximum masking level would be about 65 dB."

You
Please answer the following multiple-choice question as a hearing care professional, providing reasoning and explanation: 13 關於聽檢儀校正項目與工具的配對，下列何者錯誤?
（A）氣導校正，使用 6 c.c.耦合器（coup[illegible]
（B）骨導校正，使用 2 c.c.耦合器
（C）言語信號校正，使用聽檢儀上音量計（VU meter）
（D）聲場校正，使用擴音器將訊號音播放至檢查室內，以音壓計收音
14　長期使用某些藥物會造成不可逆之耳毒性聽損，但下列何者除外?
（A）奎寧（quinine）（B）阿斯匹林（aspirin）
（C）西司普拉定（cisplatinum）　（D）鏈黴素（streptomycin）

ChatGPT
Let's analyze each option for these two multiple-choice questions:

Question 13: Regarding the calibration of audiometric equipment and the correct pairing of items and tools, which statement is incorrect?

(A) Air conduction calibration using a 6 cc coupler: This is correct. A 6 cc coupler is typically used for calibrating air conduction transducers like headphones.

(B) Bone conduction calibration using a 2 cc coupler: This statement is incorrect. Bone conduction transducers are calibrated using a specific [illegible]ice known as a bone vibrator, not a 2 cc coupler, which is generally used for hearing aids.

(C) Speech signal calibration using the audiometer's Volume Unit (VU) meter: This is correct. The VU meter on the audiometer is used to calibrate the level of speech signals.

(D) Sound field calibration using an amplifier to play signals into the test room and recording with a sound level meter: This is correct. Sound field calibration involves playing test signals through speakers and measuring the sound level in the test area with a sound level meter.

The incorrect statement is (B) "Bone conduction calibration using a 2 cc coupler."

Question 14: Concerning irreversible ototoxic hearing loss, which of the following drugs is an exception?

(A) Quinine: Quinine can cause irreversible hearing loss.

(B) Aspirin: While high doses of aspirin can cause hearing loss, this is usually reversible once the medication is discontinued.

(C) Cisplatinum: Cisplatinum is well-known for causing irreversible ototoxic hearing loss.

(D) Streptomycin: Streptomycin is an aminoglycoside antibiotic that can cause irreversible hearing loss.

The exception, where the hearing loss is typically reversible, is (B) "Aspirin."

You
Please answer the following multiple-choice question as a hearing care professional, providing reasoning and explanation: 15 測試骨導閾值時，偶爾因為音量增大到某一程度造成該頻率聲音經過骨導器振動而放射，又迴授到外耳道經過氣傳導而產生較佳的骨導閾值，這種誤差會影響原本應該是感音性聽力損失，而變成為混音性聽損。這種誤差最常見於那個頻率?
（A）≤ 250 Hz　（B） 500 Hz　（C） 1000 Hz　（D） ≥ 2000 Hz

16　使用語音辨識閾值（SRT）做為基本聽力評估的一部分，其原因不包含下列何者?
（A）交叉驗證純音閾值（B）測量溝通障礙的程度
（C）可做超閾值測驗鑑別診斷聽神經瘤　（D）詐聾者的鑑定方法之一

ChatGPT
Let's analyze each option for these two multiple-choice questions:

Question 15: During bone conduction threshold testing, occasionally, the sound volume at certain frequencies can cause the bone conductor to vibrate and radiate sound, which then feeds back into the external auditory canal through air conduction, resulting in a better bone conduction threshold. This error can affect the diagnosis by misclassifying what should be sensorineural hearing loss as conductive or mixed hearing loss. At which frequency is this error most common?

(A) ≤ 250 Hz
(B) 500 Hz
(C) 1000 Hz
(D) ≥ 2000 Hz

The phenomenon described here is most likely to occur at lower frequencies. This is because at lower frequencies, the vibration of the bone conductor can more easily cause radiation of sound that might be picked up by air conduction. Therefore, the most common frequency for this error is likely in the lower frequency range, such as (A) ≤ 250 Hz.

Question 16: In using the Speech Recognition Threshold (SRT) as part of a basic auditory evaluation, which of the following is not a reason for its use?

(A) Cross-verification of pure tone thresholds: SRT is used to cross-verify the pure tone audiometry results, ensuring they are consistent with speech thresholds.

(B) Measurement of the degree of communication disability: While SRT provides information about speech understanding at threshold levels, it doesn't directly measure the extent of communication disability in real-life situations.

(C) Suprathreshold testing for differential diagnosis of acoustic neuroma: SRT is not typically used for differential diagnosis of acoustic neuromas. Tests like acoustic reflexes and auditory brainstem response (ABR) are more relevant in this c[illegible]xt.

(D) A method for identifying malingerers: SRT can be used to assess the consistency and reliability of responses, which can help in identifying malingerers.

The option that is not a reason for using SRT is (C) "Suprathreshold testing for differential diagnosis of acoustic neuroma." SRT is primarily used for threshold measurement and cross-verification of audiometric results, rather than for diagnosing conditions like acoustic neuroma.

You
Please answer the following multiple-choice question as a hearing care professional, providing reasoning and explanation: 17 下列何者並非 speech recognition score（SRS）之主要測試目的?
（A）助聽器效益評估　（B）不舒適響度級（UCL）的探查
（C）聽覺病灶的鑑別診斷　（D）協助聽能復健之目標設定
18　做 CNC 語音聽力檢查時，計分方式也可有很大影響。下列敘述何者錯誤?
（A）全字計分，可反映出病患預期之字詞
（B）全字計分，容易錯誤評估病患聽到的聲學線索
（C）音素計分，對病患的語言理解較能掌握
（D）音素計分，對病患的語音線索可以更[illegible]的測量

ChatGPT
Let's analyze each option for these two multiple-choice questions:

Question 17: Which of the following is not a primary purpose of the speech recognition score (SRS)?

(A) Assessment of hearing aid benefit: SRS is indeed used to evaluate the effectiveness of hearing aids in improving speech understanding.

(B) Investigation of Uncomfortable Loudness Level (UCL): SRS typically does not involve assessing UCL. UCL is determined through loudness discomfort level testing, which is different from speech recognition testing.

(C) Differential diagnosis of auditory pathologies: SRS can assist in the differential diagnosis of auditory pathologies by evaluating how well a person can understand speech, which can be indicative of certain types of hearing impairments.

(D) Assisting in setting goals for auditory rehabilitation: SRS can be used to set specific goals in auditory rehabilitation by understanding the patient's current speech recognition abilities.

The option that is not a primary purpose of SRS is (B) "Investigation of Uncomfortable Loudness Level (UCL)."

Question 18: Regarding CNC word recognition testing, which statement about scoring methods is incorrect?

(A) Whole-word scoring reflects the patient's anticipated word recognition: Whole-word scoring indeed reflects the patient's ability to correctly identify the entire word, which can indicate their anticipated word recognition performance.

(B) Whole-word scoring can lead to an overestimation of the acoustic cues the patient heard: This is true. Whole-word scoring does not account for the patient possibly guessing correctly based on partial information.

(C) Phoneme scoring is more representati[illegible] the patient's language comprehension: Phoneme scoring, where individual sounds or phonemes in the word are scored, can give a more detailed insight into the patient's auditory processing and language comprehension abilities.

(D) Phoneme scoring allows for more accurate measurement of the patient's auditory cues: This is also true. Phoneme scoring helps in understanding which specific phonetic elements are being perceived and which are not.

The incorrect statement is (B) "Whole-word scoring can lead to an overestimation of the acoustic cues the patient heard." Whole-word scoring actually reflects the patient's ability to understand words as a whole and may not necessarily overestimate the acoustic cues heard. It simply does not provide as detailed information about the perception of individual phonemes as phoneme scoring does.

You
Please answer the following multiple-choice question as a hearing care professional, providing reasoning and explanation: 19 語音聽力測試結果受使用的測驗材料影響，下列敘述何者錯誤?
（A）字詞熟悉度有明顯的影響
（B）字詞中某個音素沒有聽清楚，會自動[illegible]一個字替代
（C）聽到相似音多的字詞，比相似音少的字詞容易辨識
（D）可以使用等效性詞表替換使用
20 臨床上測試語音辨識閾值（SRT）所得之測試結果，通常以下列何種單位表示?
（A） dB HL （B） dB SL （C） dB SPL （D）百分比

ChatGPT
Let's analyze each option for these two multiple-choice questions:

Question 19: Speech audiometry results are influenced by the test material used. Which statement is incorrect?

(A) The familiarity of words has a significant impact: This is correct. Familiarity with the test words can greatly influence the results of speech audiometry.

(B) If a certain phoneme in a word is not heard clearly, it might automatically be substituted with another word: This is a common occurrence in speech recognition tests. If a listener misses a phoneme, they might guess a similar-sour[illegible] word.

(C) Words with many similar sounds are easier to recognize than those with fewer similar sounds: This statement is incorrect. In reality, words with more similar sounds (phonetically dense words) are generally more difficult to recognize accurately than words with fewer similar sounds, as they are more likely to be confused with other words.

(D) Equivalent word lists can be used interchangeably: This is correct. Equivalent word lists are designed to have similar levels of difficulty and phonetic distribution, allowing them to be used interchangeably in testing.

The incorrect statement is (C) "Words with many similar sounds are easier to recognize than those with fewer similar sounds."

Question 20: Clinically, the results of a Speech Recognition Threshold (SRT) test are typically represented in which unit?

(A) dB HL (Decibels Hearing Level): This is correct. SRT is usually measured and reported in dB HL. It represents the minimum hearing leve[illegible] which speech is recognized as meaningful symbols 50% of the time.

(B) dB SL (Decibels Sensation Level): This is not typically used for SRT but rather for suprathreshold speech testing.

(C) dB SPL (Decibels Sound Pressure Level): While SPL is a measure of sound pressure, SRT results are conventionally reported in dB HL.

(D) Percentage: While speech recognition or discrimination scores are often reported in percentages, SRT is usually reported in dB HL.

Therefore, the correct answer is (A) "dB HL."

You
Please answer the following multiple-choice question as a hearing care professional, providing reasoning and explanation: 21 有關語言察覺閾值（speech detection thresholds）敘述，下列何者錯誤?
（A）語音察覺閾值結果表現優於語音接收[illegible]表現
（B）受測者須聽到語音訊號 50%以上
（C）與純音 500 Hz、1000 Hz、2000 Hz 頻率相關性較高
（D）常用於無法獲得語音接收閾值時
22　執行語音聽力檢查時，若使用現場發聲唸出雙音節字詞，需藉由音量計（VU meter）上的指針監控音量平穩性。下列敘述何者正確?
（A）音量計指針在唸出雙音節字詞時，最大不超過 10 dB 即可
（B）音量計指針在唸出雙音節字詞時，每個音節的音量都須維持在 0 dB 上
（C）音量計指針在唸出雙音節字詞時，只要第一音節的重音音量須維持在 0 dB 上
（D）音量計指針在唸出雙音節字詞時，只要第二音節的重音音量須維持在 0 dB 上

ChatGPT
Let's analyze each option for these two multiple-choice questions:

Question 21: Concerning speech detection thresholds (SDTs), which statement is incorrect?

(A) Speech detection threshold results are better than speech reception threshold results: This is correct. The speech detection threshold (S[illegible]) is the level at which speech is just detectable, whereas the speech reception threshold (S[illegible]) is the level at which speech is understood.

Therefore, SDT is typically lower (better) than SRT.

(B) The test subject must detect the speech signal more than 50% of the time: This is correct. SDT is defined as the lowest level at which the subject can detect the presence of speech 50% of the time.

(C) High correlation with pure tone frequencies of 500 Hz, 1000 Hz, and 2000 Hz: This is correct. SDT generally has a good correlation with the average thresholds at these frequencies, as they are important for the perception of speech.

(D) Often used when it is not possible to obtain a speech reception threshold: This is correct. SDT is particularly useful in individuals who are unable to participate in more complex speech understanding tests.

All statements are correct, but if one is to be chosen as the least accurate, it might be (B) "The test subject must detect the speech signal more than 50% of the time" because SDT is usually defined as the level at which speech is just detecta[illegible] not necessarily understood or recognized more than 50% of the time.

Question 22: When performing a speech audiometry test using live voice for bisyllabic words, the stability of the volume is monitored with a VU meter. Which statement is correct?

(A) The VU meter needle should not exceed a maximum of 10 dB when saying bisyllabic words: This is incorrect. The goal is usually to keep the needle around 0 dB, not within a 10 dB range.

(B) The VU meter needle should maintain 0 dB for each syllable of the bisyllabic words: This is generally the correct approach. For live voice testing, maintaining a consistent level around 0 dB for each syllable ensures that the speech stimulus is presented at a consistent and appropriate level.

(C) The VU meter needle should maintain 0 dB only for the first syllable of the bisyllabic words: This is incorrect. Consistency across all syllables is important for accurate testing.

(D) The VU meter needle should maintain [illegible] only for the second syllable of the bisyllabic words: This is also incorrect for the same reason as (C).

The correct statement is (B) "The VU meter needle should maintain 0 dB for each syllable of the bisyllabic words." This ensures consistent and accurate presentation of the speech stimulus during the test.

You

Please answer the following multiple-choice question as a hearing care professional, providing reasoning and explanation: 23 關於語音信號校正，下列敘述何者錯誤?
（A）耳機語音信號的校正可透過參考等效聽閾音壓級（RETSPLs）作為實際輸出音壓級參考值
（B）以錄音方式呈現的語音測試材料，一般是以 1000 Hz 70 dB 的純音為校正音
（C）透過校正後語音呈現於 TDH49/TDH50 耳機時 0 dB HL 輸出音量實際約為 10 dB SPL
（D）以現場語音呈現的語音測試材料，可透過音量計（VU meter）來監測實際輸出音量

24 下列何種情況比較不會影響遮蔽位階差（masking level difference, MLD）之數值?
（A）周邊聽力損失的程度 （B）腦幹部位的損傷 （C）老化的聽覺系統 （D）聽覺皮質部位的損傷

ChatGPT

Let's analyze each option for these two multiple-choice questions:

Question 23: Concerning speech signal calibration, which statement is incorrect?

(A) Calibration of earphone speech signals can be done using Reference Equivalent Threshold Sound Pressure Levels (RETSPLs) as a reference for actual output sound pressure levels: This is correct. RETSPLs provide a reference for calibrating the output level of earphones.

(B) For recorded speech test materials, a pure tone of 1000 Hz at 70 dB is generally used as the calibration tone: This is also correct. This method is commonly used to ensure that the recorded speech material is presented at a consistent and appropriate level.

(C) When calibrated speech is presented through TDH49/TDH50 headphones, a 0 dB HL output is approximately equal to 10 dB SPL: This statement is incorrect. The actual SPL corresponding to 0 dB HL depends on the RETSPLs for the sp[illegible]cific headphones and transducer used, and it's not typically around 10 dB SPL.

(D) For live voice speech test materials, the actual output level can be monitored with a Volume Unit (VU) meter: This is correct. A VU meter is used to ensure that the live voice is delivered at a consistent level.

The incorrect statement is (C) "When calibrated speech is presented through TDH49/TDH50 headphones, a 0 dB HL output is approximately equal to 10 dB SPL."

Question 24: Which of the following conditions is less likely to affect the value of the masking level difference (MLD)?

(A) Degree of peripheral hearing loss: Peripheral hearing loss can affect MLD measurements, as it impacts the ability to perceive differences in phase or time cues used in these tests.

(B) Damage to the brainstem area: Brainstem damage can significantly affect MLD, as it may disrupt the auditory pathways essential for processing binaural cues.

(C) Aging auditory system: Aging can affe[illegible]LD, particularly as it relates to the processing of binaural cues and temporal processing.

(D) Damage to the auditory cortex area: While the auditory cortex is important for processing complex sounds, its role in basic binaural processing like that measured by MLD is less direct compared to structures like the brainstem.

The condition less likely to have a significant impact on MLD values is (D) "Damage to the auditory cortex area." The other conditions have more direct influences on the auditory pathways and processes involved in MLD.

You

Please answer the following multiple-choice question as a hearing care professional, providing reasoning and explanation: 25 執行Weber test 時，聲音會偏向個案優耳側聽到，此一現象稱為下列何者?
（A） recruitment （B） UCL （C） stenger effect （D） occlusion effect
26 下列何項檢查項目原理是運用音強差異閾（difference limen for intensity, DLI）的能力?
（A） Alternate Binaural Loudness Balance（ABLB）test
（B） Short Increment Sensitivity Index（[illegible]）
（C） Bekesy audiometry
（D） Stenger test

ChatGPT

Let's analyze each option for these two multiple-choice questions:

Question 25: During the Weber test, if the sound is perceived as louder in the better-hearing ear, this phenomenon is known as which of the following?

(A) Recruitment: Recruitment is a phenomenon associated with sensorineural hearing loss, where there's an abnormal growth of loudness. However, it's not specifically related to the Weber test.

(B) UCL (Uncomfortable Loudness Level): UCL refers to the level at which sound becomes uncomfortably loud for an individual, and is not related to the lateralization of sound in the Weber test.

(C) Stenger effect: The Stenger principle st[illegible]s that if two sounds of the same frequency but different intensities are presented to both e[illegible]s simultaneously, only the louder sound will be perceived. This is not the principle demonstrated in the Weber test.

(D) Occlusion effect: The occlusion effect is the increase in perceived loudness of bone-conducted sound when the outer ear is blocked. However, this is not the principle at work in the Weber test.

The phenomenon where the sound is perceived as louder in the better-hearing ear during the Weber test does not have a specific name from the options given. The Weber test is used to determine the type of hearing loss (conductive vs. sensorineural), and in cases of unilateral sensorineural hearing loss, the sound is typically perceived in the better-hearing ear. However, this phenomenon itself is not named as any of the options provided.

Question 26: Which of the following test items is based on the principle of utilizing the ability of difference limen for intensity (DLI)?

(A) Alternate Binaural Loudness Balance (ABLB) test: This test measures the loudness balance between ears but is not specifically based [illegible] the DLI.

(B) Short Increment Sensitivity Index (SISI): The SISI test measures an individual's ability to detect small changes in intensity, which is directly related to the DLI. It's often used in the diagnosis of cochlear pathology, specifically for identifying recruitment in sensorineural hearing loss.

(C) Bekesy audiometry: Bekesy audiometry is a method of measuring hearing thresholds, but it does not primarily focus on DLI.

(D) Stenger test: The Stenger test is used in cases of unilateral or asymmetrical hearing loss to determine if a patient is feigning hearing loss. It does not specifically rely on the DLI.

The test that utilizes the ability of DLI is (B) "Short Increment Sensitivity Index (SISI)."

You

Give the option you think is most suitable for Q25

ChatGPT

For question 25, regarding the Weber test, the most suitable option among those provided is:

(D) Occlusion effect.

However, it's important to note that this isn't entirely accurate. In the Weber test, when a tuning fork is placed on the forehead or central part of the skull, the sound is typically perceived as louder in the ear that has better hearing in cases of sensorineural hearing loss, or in the affected ear in cases of conductive hearing loss. This phenomenon isn't precisely described by any of the options given, including the occlusion effect, which refers to the increased loudness of bone-conducted sound when the outer ear is blocked.

In clinical practice, the result of the Weber test is interpreted based on lateralization of the sound, but this specific phenomenon doesn't have a distinct name like 'recruitment' or 'Stenger effect'. The options provided do not accurately represent the phenomenon observed in the Weber test.

You

Please answer the following multiple-choic[illegible]uestion as a hearing care professional, providing reasoning and explanation: 27 有關耳蝸死[illegible]（cochlear dead region）的描述，下列何者錯誤?
（A）在基底膜上，內毛細胞或聽神經纖維無法正常運作的區域稱之
（B）由於異頻聆聽（off-frequency listening）的因素，仍可察覺到耳蝸死區頻率的聲音
（C）當感覺神經性聽力損失的聽力圖形太過陡峭傾斜或有些頻率的聽閾達 70 dB 以上時，即要懷疑是否存在耳蝸死區
（D）聽力圖本身即可作為是否存在耳蝸死區的信賴指標
28 有關 Stenger 氏測驗，下列敘述何者正確?
（A）相同頻率的音量優耳+10 dB SL，劣耳-10 dB SL 同時放出聲音，病人如果有反應是 Stenger negative
（B）相同頻率的音量優耳-10 dB SL，劣耳+10 dB SL 同時放出聲音，病人如果有反應是 Stenger positive
（C）相同頻率的音量優耳+10 dB SL，劣耳-10 dB SL 同時放出聲音，病人如果沒有反應是 Stenger negative
（D）相同頻率的音量優耳-10 dB SL，劣耳+10 dB SL 同時放出聲音，病人如果沒有反應是 Stenger positive

ChatGPT

Let's analyze each option for these two mult[illegible]ple-choice questions:

Question 27: Concerning cochlear dead regions, which statement is incorrect?

(A) Refers to areas on the basilar membrane where the inner hair cells or auditory nerve fibers do not function normally: This is a correct description of cochlear dead regions.

(B) Due to off-frequency listening, sounds at the frequency of the cochlear dead region can still be perceived: This is correct. Even if there's a dead region, a person might still perceive sounds at those frequencies due to the functioning of adjacent areas.

(C) A steeply sloping audiogram or thresholds above 70 dB at some frequencies may suggest the presence of cochlear dead regions: This is correct. A steeply sloping audiogram or high thresholds might indicate the possibility of cochlear dead regions.

(D) The audiogram itself can be a reliable indicator of the presence of cochlear dead regions: This statement is incorrect. While an audiogram can suggest the possibility of cochlear dead regions, it cannot definitively confirm their presence. Additional specialized tests are required for accurate identification.

The incorrect statement is (D) "The audiogram itself can be a reliable indicator of the presence of cochlear dead regions."

Question 28: Regarding the Stenger test, which statement is correct?

(A) If the same frequency is presented at +10 dB SL to the better ear and -10 dB SL to the poorer ear and the patient responds, it is Stenger negative: This is incorrect. In the Stenger test, if the patient with feigned unilateral hearing loss hears the tone in the 'poorer' ear but does not respond (pretending not to hear), the test is Stenger positive.

(B) If the same frequency is presented at -10 dB SL to the better ear and +10 dB SL to the poorer ear and the patient responds, it is Stenger positive: This is incorrect. The presentation levels are not typically set in this way for the Stenger test.

(C) If the same frequency is presented at +10 dB SL to the better ear and -10 dB SL to the poorer ear and the patient does not respond, it is Stenger negative: This is incorrect. In this situation, a lack of response when the patient should [illegible] heard the tone in the 'poorer' ear indicates a Stenger positive test, suggesting non-organic hearing loss.

(D) If the same frequency is presented at -10 dB SL to the better ear and +10 dB SL to the poorer ear and the patient does not respond, it is Stenger positive: This is incorrect for the same reasons as (B).

The correct description of the Stenger test is closest to (C), but with clarification: If the same frequency is presented at a louder level (e.g., +10 dB SL) to the poorer ear and a quieter level to the better ear, and the patient with feigned unilateral hearing loss does not respond (pretending not to hear the tone), the test is Stenger positive. This suggests that the patient is feigning hearing loss in the 'poorer' ear.

You

Please answer the following multiple-choice question as a hearing care professional, providing reasoning and explanation: 29 12 歲陳姓女學生，主訴上課聽不清楚老師及同學說話，純音聽力檢查結果：右耳中度至重度聽力損失，左耳中度聽力損失；語音辨識閾右耳 35 分貝，左耳 40 分貝；語音辨識率右耳使用 40 分貝測試為 88%，[illegible]耳使用 45 分貝測試為 92%，下列何者為此個案最有可能的診斷?
（A）非器質性聽力損失 （B）有響音重振現象
（C）有聽覺敏感（supersensitivity） （D）有聽覺過敏（hyperacusis）
30 對 2 歲半的小華施行視覺增強聽力檢查法時，下列敘述何者最不恰當?
（A）可以使用比較複雜、會亮、會動的視覺增強物
（B）習慣化發生時，先休息 10 分鐘再重新進行測驗
（C）為了避免習慣化的出現，可以在習慣化前縮短增強物的持續時間
（D）純音聽檢時最常採取「上 10 下 10 分貝」或「上 10 下 20 分貝」法

ChatGPT

Let's analyze each option for these two multiple-choice questions:

Question 29: A 12-year-old female student named Chen complains of difficulty hearing her teachers and classmates in class. Audiometric results: moderate to severe hearing loss in the right ear and moderate hearing loss in the left ear; speech recognition threshold of 35 dB in the right ear and 40 dB in the left ear; speech recognition [illegible]ore of 88% at 40 dB in the right ear and 92% at 45 dB in the left ear. Which of the following is the most likely diagnosis for this case?

(A) Non-organic hearing loss: This diagnosis typically presents with inconsistent or implausible audiometric findings. In this case, the findings seem consistent and plausible, making non-organic hearing loss less likely.

(B) Recruitment phenomenon: Recruitment is associated with sensorineural hearing loss and is characterized by an abnormal growth in perceived loudness. However, recruitment itself cannot be diagnosed solely based on the information given.

(C) Auditory supersensitivity: This term is not commonly used in clinical audiology to describe a specific condition, making it an unlikely diagnosis based on the given data.

(D) Hyperacusis: Hyperacusis is an increased sensitivity to everyday sounds, but the audiometric findings described do not specifically indicate hyperacusis.

Given the consistent audiometric findings showing moderate to severe hearing loss and the relatively good speech recognition scores [illegible]elevated levels, the most likely diagnosis among the given options is (B) "Recruitment phenomenon." However, it's important to note that a definitive diagnosis would typically require additional testing and information.

Question 30: When conducting a visual reinforcement audiometry (VRA) test on a 2-and-a-half-year-old child named Xiao Hua, which of the following statements is most inappropriate?

(A) Complex, bright, and moving visual reinforcers can be used: This is appropriate for VRA, as engaging visual stimuli are effective in maintaining a young child's attention.

(B) If habituation occurs, take a 10-minute break before resuming testing: This is an appropriate strategy. Children can become habituated to stimuli, and a break can help reset their attention.

(C) To avoid habituation, shorten the duration of visual reinforcers before habituation sets in: This can be an effective strategy to maintain the child's engagement and responsiveness.

(D) The most common approach in pure tone audiometry is the 'up 10 dB, down 10 dB' or 'up 10 dB, down 20 dB' method: This statement is ab[illegible]the testing procedure for pure tone audiometry, not specifically related to VRA. While it's a correct statement about pure tone testing methods, it's not directly relevant to the use of visual reinforcers in VRA.

The most inappropriate statement in the context of visual reinforcement audiometry is (D) "The most common approach in pure tone audiometry is the 'up 10 dB, down 10 dB' or 'up 10 dB, down 20 dB' method," as it doesn't pertain to the specifics of using visual reinforcers in VRA.

You

Please answer the following multiple-choice question as a hearing care professional, providing reasoning and explanation: 31 下列何者並非行為觀察（Behavior observation）聽力檢查之主要測試目的?
（A）與其他檢測（例如：ABR）做交叉比對 （B）檢測結果為最小反應位階
（C）觀察嬰幼兒對聲音之反應 （D）獲取分耳分頻之聽力閾值

ChatGPT

Let's analyze each option for question 31, w[illegible]h is about the primary purpose of behavioral observation audiometry:

(A) Cross-compare with other tests (e.g., ABR): Behavioral observation can be used to supplement and cross-compare results with objective tests like Auditory Brainstem Response (ABR). This helps in confirming the findings and providing a comprehensive assessment.

(B) The test result is the minimum response level: Behavioral observation audiometry is aimed at observing the minimum response or the lowest level at which a response to sound is observed. This is a correct purpose of the test.

(C) Observe the infant's or young child's reaction to sound: One of the main purposes of behavioral observation audiometry is to observe the reactions of infants and young children to sound, as they cannot provide verbal feedback or perform more standardized audiometric tasks.

(D) Obtain frequency-specific hearing thresholds for each ear: Behavioral observation audiometry is not typically used for obtaining detailed, frequency-specific thresholds for each ear. It is more about observing general responses to sound rather than precise thresholds.

The option that is not a primary goal of behavioral observation audiometry is (D) "Obtain frequency-specific hearing thresholds for each ear." Behavioral observation audiometry is more about observing responses to sound in general and is not designed for detailed, frequency-specific threshold determination.

You

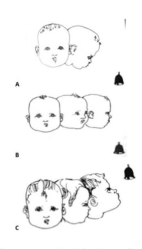

Please answer the following multiple-choice question as a hearing care professional, providing reasoning and explanation: 32 嬰幼兒聽覺發展的順序，下列何者正確?

（A） ①→②→③ （B） ②→①→③ （C） ③→②→① （D） ②→③→①

ChatGPT

In the image, there are three drawings of infant heads labeled as ①, ②, and ③, likely intended to represent different stages of an infant's auditory response development.

The typical progression of auditory development in infants is as follows:

1. The infant's response to sound begins with a startle reflex, which is a general, whole-body response to a sudden, loud noise. This is [illegible]y basic form of auditory awareness and typically develops first.
2. As the infant's auditory system matures, they begin to turn their head or eyes towards the direction of a sound. This shows a more sophisticated level of auditory perception as the infant is not only aware of the sound but also begins to localize it.
3. The final stage is when the infant starts to recognize and respond to softer sounds or voices, particularly those that are familiar, such as the voice of a parent or caregiver. This indicates a further advanced level of auditory development, which includes sound discrimination and recognition.

Based on the standard developmental progression, infants typically go from a broad startle response to a more refined head-turning response and then to the recognition of specific sounds or voices. If the numbers on the heads in the image correspond to these developmental stages, then the correct sequence would be the one that starts with the startle reflex, followed by head turning, and ends with sound recognition.

Without seeing the image, I can only infer that the sequence from broad, nonspecific responses to specific, targeted responses is the correct [illegible]elopmental order. If the numbers correspond to these stages, then the correct answer would be:

(A) ①→②→③

This is assuming that ① corresponds to the startle reflex, ② to head turning, and ③ to the recognition of specific sounds or voices.

You

Please answer the following multiple-choice question as a hearing care professional, providing reasoning and explanation: 33 在嬰幼兒視覺增強聽力檢查（VRA）時，建議每個刺激音及反應判斷的最佳測量時長（duration）為何?
（A） 1秒 （B） 2秒 （C） 3秒 （D） 4秒
34 小明足月產、目前 20 個月大；小華早產 6 週、目前 7 個月大。下列何種行為聽檢法分別適合小明和小華?
（A） CPA、ABR （B） CPA、BOA （C） VRA、ABR （D） VRA、BOA

ChatGPT

Question 33:

In Visual Reinforcement Audiometry (VRA), which is used primarily for infants and toddlers, the duration of stimulus presentation and the time window for response judgment should be brief enough to maintain the child's attention but long enough to elicit a reliable response. The recommended duration typically ranges from very short to several seconds. For VRA, a stimulus duration of approximately 2 seconds is often recommended. This is because a stimulus of this length is usually sufficient to attract the child's attention and elicit a head turn toward the sound without being so long that the child loses interest. Therefore, the best measurement duration for each stimulus and response judgment in VRA would be:

(B) 2 seconds

Question 34:

For behavioral hearing tests, the choice of procedure depends on the age and developmental status of the child.

- Conditioned Play Audiometry (CPA) is ge[illegible]lly suitable for toddlers and preschoolers, typically between the ages of 2 and 5 years, who can follow instructions and engage in a game-like activity. Since Xiao Ming is 20 months old, he is approaching the lower end of the age range where CPA might be attempted, depending on his individual development and ability to engage in play.
- Behavioral Observation Audiometry (BOA) is often used for younger infants who are not yet able to participate in more structured testing like CPA or VRA. It involves observing the child's natural responses to sounds, such as startle or change in activity.
- Visual Reinforcement Audiometry (VRA) is suitable for infants and young toddlers, typically between 6 months and 2 years of age, who can turn their heads towards a sound and be reinforced visually.
- Auditory Brainstem Response (ABR) testing is an objective measure that does not require behavioral responses and is suitable for all ages, including newborns and infants who cannot reliably participate in behavioral tests.

Given that Xiao Ming is close to 2 years old, he might be transitioning from VRA to CPA, depending on his cognitive and developmental level. Xiao Hua, being 7 months old (adjusted for prematurity), is likely still within the age range for VRA, which can be used to get reliable responses through head-turning.

Therefore, the suitable behavioral hearing tests for Xiao Ming and Xiao Hua would be:

(C) VRA for Xiao Ming, considering he may not yet be ready for CPA, and ABR for Xiao Hua, who is much younger and was born prematurely. However, if Xiao Hua's development is consistent with his chronological age and he can turn his head toward sounds, VRA would be suitable. Without specific details about their development, it is not possible to make a definitive recommendation.

You

Please answer the following multiple-choice question as a hearing care professional, providing reasoning and explanation: 35 對於嬰幼兒聽力檢查之敘述，下列何者最不適當?
（A） 6 週到 4 個月大後的階段，一般會出現聽動眼反射（auro-oculogyric reflex）或朝向音源的轉頭動作
（B） 4 個月左右的嬰兒，較易觀察到的反應是 Moro reflex、聽瞼反射（auropalpebral reflex）等
（C） 9 到 13 個月的嬰幼兒對於顫音、語音和噪音器（noise maker）三種方式給聲音刺激，觀察他們的反應，一般而言用語音所需的音量較大
（D）嬰兒在聽到聲音時的吸吮反應，開始[illegible]吮、加速或停止等都有可能
36 關於 conditioned play audiometry 的使[illegible]之敘述，下列何者錯誤?
（A）適用年齡為 2 至 4 歲
（B）施測音量的選擇應保持彈性，不一定需要使用減 10 加 5 分貝的原則
（C）初始音量應給予 40 ~ 50 分貝或參考 SRT 音量
（D）高頻聽損為臨床常見，故以 4000 Hz 為優先施測的頻率

ChatGPT

Question 35: Concerning infant hearing assessments, which statement is most inappropriate?

(A) From 6 weeks to 4 months of age, infants generally exhibit the auro-oculogyric reflex or turn their heads toward sound sources. This is a correct statement. At this age, infants begin to show orienting responses to sound.

(B) At around 4 months of age, the responses that are easier to observe include the Moro reflex and the auropalpebral reflex. This statement is not entirely appropriate as by around 4 months of age, the Moro reflex, which is a startle reflex present at birth, begins to disappear. The auropalpebral reflex, a blink reflex to soun[illegible]ay still be observable.

(C) From 9 to 13 months of age, infants may respond to stimuli like warble tones, speech, and noise makers, typically requiring a louder volume for speech. This statement seems plausible, as infants at this age may require louder sounds to respond, especially in a non-clinical setting with background noise.

(D) An infant's sucking response to sound, such as starting to suck, sucking faster, or stopping, is possible. This is true, as changes in sucking behavior can be a response to auditory stimuli in very young infants.

The least appropriate statement here is (B) "At around 4 months of age, the responses that are easier to observe include the Moro reflex," because the Moro reflex typically disappears by this age.

Question 36: Regarding the use of conditioned play audiometry, which statement is incorrect?

(A) Appropriate for ages 2 to 4 years. This i[illegible]orrect, as conditioned play audiometry is typically used for children in this age range.

(B) The level of the test tones should be flexible, not necessarily using the down 10 up 5 dB principle. This is correct. While the down 10 up 5 dB method is a common approach in pure tone audiometry, flexibility in presentation levels can be important in testing young children.

(C) The initial test level should be 40–50 dB or refer to the SRT level. This is a reasonable starting point for the test, especially if the child's SRT is known.

(D) High-frequency hearing loss is common, so 4000 Hz should be prioritized for testing. This statement is incorrect because, in conditioned play audiometry, it is typical to begin with frequencies that are easier for the child to hear and respond to, such as 1000 Hz or 2000 Hz. Starting with a frequency where hearing loss is common may not be the most effective way to establish the child's response behavior at the beginning of the test.

The incorrect statement is (D) "High-frequency hearing loss is common, so 4000 Hz should be prioritized for testing."

You

Please answer the following multiple-choice question as a hearing care professional, providing reasoning and explanation: 37 在 VRA 測試中，關於聽檢室內助手聽力師的描述，下列何者錯誤?
（A）可協助觀察嬰兒反應
（B）協助受測嬰兒之視線維持在中線位置
（C）位置安排在受測嬰兒之側後方，以免擋住主責聽力師的視線
（D）可手持多彩無聲之玩具
38 Moore（1995）文獻建議視覺增強聽力檢查（VRA）測試中，可加入控制試驗確認測試結果可信度， 若假性反應超過控制試驗多少百分比時，應考慮重測?
（A） 10% （B） 15% （C） 20% （D） 25%

ChatGPT

Question 37: In a VRA test, which description of the role of the assistant audiologist inside the test booth is incorrect?

(A) Can assist in observing the infant's responses: This is correct. An assistant can help monitor the infant's responses to auditory stimuli.

(B) Helps keep the test infant's gaze centered: This is correct. The assistant can help maintain the infant's attention toward the front where stimuli will be presented.

(C) Is positioned behind and to the side of the test infant to avoid blocking the primary audiologist's view: This is correct. It is important that the primary audiologist has a clear view of the infant's responses.

(D) Can hold colorful, noiseless toys: This statement is incorrect in the context of VRA testing. While the assistant may use toys to help keep the infant's attention forward, they should not hold them during the actual test as this could be distracting or could provide a visual cue that coincides with the auditory stimulus, potentially confounding the test results. The toys used in VRA are usually controlled and presented as part of the reinforcement contingent on the child's correct localization of the sound.

The incorrect statement is (D) "Can hold colorful, noiseless toys."

Question 38: According to Moore (1995), when should one consider retesting if false responses exceed a certain percentage during control trials in a VRA test?

Control trials are conducted without presenting a sound stimulus to ensure that the child is not responding at times when no sound is presented (false positives). If the rate of false positives is too high, it can suggest that the responses in the test may not be reliable indicators of hearing.

While I cannot access external sources to verify the specific percentage recommended by Moore (1995), in clinical practice, a threshold for false positives that would warrant retesting is often set at a level where confidence in the test results becomes questionable. Commonly, a percentage of 20% or more may be used as a rule of thumb in various psychological and sensory testing paradigms, including hearing tests, to indicate an unacceptable level of false responses.

Given the options provided and without being able to confirm the specific percentage from Moore (1995), a conservative and logical threshold to consider retesting would be:

(C) 20%

This percentage is often used as a guideli[illegible] clinical settings, though the specific criteria may vary based on the clinician's judgment and the standard operating procedures of the testing facility.

You

Please answer the following multiple-choice question as a hearing care professional, providing reasoning and explanation: 39 顫音、語音、窄頻噪音用於 0 ~ 12 個月嬰幼兒行為聽檢時，其檢測閾值間之關係，下列敘述何者正確?
（A）窄頻噪音 > 顫音 > 語音 （B）窄頻噪音 > 語音 > 顫音
（C）語音 > 窄頻噪音 > 顫音 （D）語音 > 顫音 > 窄頻噪音
40 關於進行兒童行為聽力評估的敘述，下列何者錯誤?
（A）檢查開始前，聽力師應與前來施測的兒童建立良好的互動關係
（B）兒童聽力檢查應使用插入式耳機避免耳道塌陷
（C）兒童施測的時間不宜過久，施測方法可彈性選擇
（D）施測時應一次完成 250 Hz ~ 4000 Hz 的頻率，方能提供完整的聽力訊息

ChatGPT

Question 39:

The detection thresholds for warble tones, speech, and narrowband noise during behavioral hearing assessments for 0 to 12-month-old infants can vary due to the different spectral properties of these stimuli and the infant's auditory development. Typically, speech thresholds are lower (better) than warble tone thresholds, which are in turn lower than narrowband noise thresholds because speech has more frequency components and is usually more interesting or engaging to the infant, which can elicit a stronger response. Warble tones are also more engaging than narrowband noise and can be easier for infants to detect. Therefore, the correct relationship between the detection thresholds for these stimuli is:

(B) Narrowband noise > Speech > Warble tones

This assumes that the '>' symbol indicates "higher thresholds" (or worse hearing) for the stimulus to the left compared to the stimulus to the right.

Question 40:

In the context of pediatric behavioral hearin[illegible] assessments:

(A) Correct: The audiologist should establish a good rapport with the child before beginning the test to ensure cooperation and accurate results.

(B) Correct: Insert earphones can be used to prevent the collapse of the ear canal, which can be a concern with conventional supra-aural headphones, especially in children with soft cartilage.

(C) Correct: The attention span of children is limited, so the test duration should be as brief as possible, and the methodology should be flexible to maintain the child's engagement.

(D) Incorrect: While it is ideal to test across the full frequency range to obtain a complete picture of a child's hearing, it is not always feasible to do so in one continuous testing session, particularly with very young children. It may be necessary to break up the testing into shorter sessions or to prioritize certain frequencies based on the child's age and ability to respond. Additionally, testing very low frequencies like 250 Hz can be challenging in young children due to their limited response to these tones.

Therefore, the incorrect statement is (D) "Te[illegible]ting should be completed in one session from 250 Hz to 4000 Hz to provide complete hearing in[illegible]mation."

You

Please answer the following multiple-choice question as a hearing care professional, providing reasoning and explanation: 41 依照美國聽力學會（AAA, 2011）與美國聽語學會（ASHA, 1997）的

聽篩指引，學齡前與學齡兒童施
行純音聽力篩檢時，下列何種情況需做轉介？
（A）任一耳任一篩檢頻率未通過
（B）任一耳任一篩檢頻率未通過，並經過複篩仍未通過
（C）任一耳兩個或以上篩檢頻率未通過
（D）任一耳兩個或以上篩檢頻率未通過，並經過複篩仍未通過
42 若需長時間於潮濕悶熱的噪音環境下工作，且考慮配戴者的舒適性，則下列護具何者是最理想的選擇？
（A）耳罩 （B）耳塞 （C）半耳式耳塞 （D）耳塞與耳罩合併使用

ChatGPT

Question 41:

According to hearing screening guidelines from professional associations like the American Academy of Audiology (AAA, 2011) and the American Speech-Language-Hearing Association (ASHA, 1997), referral following a hearing screening is typically indicated if there is a failure to pass the screening at any frequency in either ear. However, most programs recommend a rescreening first before making a referral to rule out temporary conditions or errors in the initial screening. The referral is typically made if the child does not pass upon rescreening.

(B) "Any one ear at any screening frequency not passed, and upon rescreen, still not passed" is the situation that requires a referral. This accounts for the possibility of temporary conditions or errors during the first screening and ensures that referrals are made based on more consistent findings.

Question 42:

When working for extended periods in a hot, humid, and noisy environment, the ideal hearing protection should be effective at noise reduction while also being comfortable to wear for long durations.

(A) Earmuffs provide good attenuation and [illegible] comfortable for some users, but they can be hot and might not be the best option for a humid environment.
(B) Earplugs are effective at reducing noise levels and are less likely to contribute to overheating compared to earmuffs.
(C) Semi-insert earplugs provide a compromise between earplugs and earmuffs but may offer less protection than full earplugs.
(D) Using earplugs in combination with earmuffs provides the greatest level of protection and can be considered in extremely noisy environments. However, this combination might be the least comfortable in hot and humid conditions due to increased heat retention.

Considering the need for comfort in a hot and humid environment, the best option would likely be:

(B) "Earplugs," because they are less likely to retain heat compared to earmuffs and still provide adequate noise reduction. Comfort is crucial in maintaining compliance with the use of hearing protection over long periods.

You

Please answer the following multiple-choi[illegible]uestion as a hearing care professional, providing reasoning and explanation: 43 關於職業性噪音聽損，下列敘述何者錯誤？
（A）聽損的過程是緩慢漸進性的
（B）典型的臨床特徵為兩側對稱的高頻聽損
（C）最大的聽損點常出現在 4k Hz
（D）間歇性噪音比持續性噪音傷害更大
44 關於高膽紅素血症（hyperbilirubinemia）的敘述，下列何者最不適當？
（A）黃疸通常在出生後的前 3 天達高峰
（B）血液中的膽紅素濃度過高，會穿過血腦障壁造成核黃疸（kernicterus）
（C）可藉由照光或換血來避免或降低傷害出現
（D）可能會造成周邊或中樞聽覺處理異常

ChatGPT

Question 43:

The progression of occupational noise-induced hearing loss (NIHL) typically follows a specific pattern. Let's evaluate each statement:

(A) The process of hearing loss is slow and progressive: This is correct. NIHL often develops gradually over years of exposure to harmf[illegible]oise levels.

(B) A typical clinical feature is bilateral symmetrical high-frequency hearing loss: This is correct. NIHL usually affects both ears equally and often first impacts the higher frequencies.

(C) The greatest hearing loss often occurs at 4 kHz: This is correct and is known as the "4 kHz notch," a hallmark of early NIHL.

(D) Intermittent noise causes more damage than continuous noise: This statement is incorrect. Continuous exposure to high levels of noise is typically more damaging than intermittent noise. Intermittent noise allows for periods of "rest" for the ears, which can mitigate the extent of hearing damage to some degree.

The incorrect statement is (D) "Intermittent noise is more damaging than continuous noise."

Question 44:

Concerning hyperbilirubinemia, let's examine the appropriateness of each statement:

(A) Jaundice usually peaks during the first [illegible]ays after birth: This is correct. Physiological jaundice typically peaks around the second or third day after birth.

(B) High concentrations of bilirubin in the blood can cross the blood-brain barrier and cause kernicterus: This is also correct. Kernicterus is a serious condition that can result from high levels of bilirubin in the blood.

(C) Hyperbilirubinemia can be prevented or the damage can be reduced through phototherapy or exchange transfusion: This is true. These treatments help to lower bilirubin levels in the blood.

(D) It may cause peripheral or central auditory processing abnormalities: This is correct. Hyperbilirubinemia, particularly when it leads to kernicterus, can result in auditory neuropathy or other types of hearing impairment.

All the statements presented are appropriate descriptions of hyperbilirubinemia and its effects. However, if one must be chosen as the least appropriate based on common understanding, it might be necessary to consider the context in which statement (D) "It may cause peripheral or central auditory processing abnormalities" [illegible]aid. While hyperbilirubinemia can lead to these issues, the wording might imply that it's a common outcome, which is not the case with current medical management. Nonetheless, without additional context, all options are factually correct regarding hyperbilirubinemia.

You

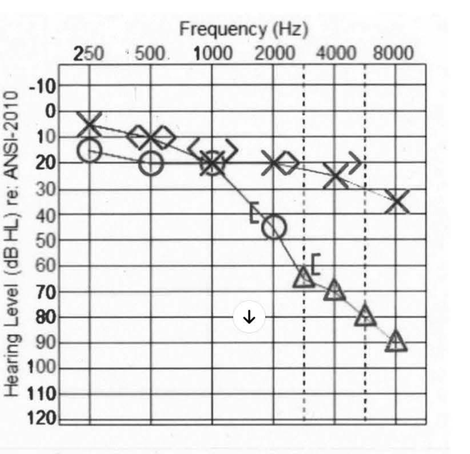

	SRT	WRS %	@ HL
Right	20	64%	50
Right		40% (m)	70
Left	20	88%	50
Left		100% (m)	70

Please answer the following multiple-choice question as a hearing care professional, providing reasoning and explanation: 65 歲女性，使用英文為母語，主訴右耳悶脹但不會痛，也沒有頭暈。耳鳴已久且右耳比左耳嚴重，右耳有點開始聽不清楚。圖中聽力報告為使用 NU-6 之測驗材料結果，請依序回答第 45 題至第 47 題：45 此個案的聽力圖結果判讀下列何者正確？
（A） L't high tone CHL； R't mild to severe SNHL
（B） L't WNL except 8k Hz； R't mild to p[illegible]und mixed type HL
（C） L't mild SNHL； R't mild to severe mixed type HL
（D） L't high tone HL； R't mild to severe SNHL
46 此個案右耳的回跌指數（rollover index, RI）為何？
（A） 0.29 （B） 0.38 （C） 0.6 （D） 0.48
47 此個案右耳最可能存在什麼問題？
（A）外耳或中耳異常 （B）老年性聽損 （C）耳蝸後病變 （D）耳蝸死區

ChatGPT

Based on the audiogram provided, let's address each question:

Question 45: What is the correct interpretation of this individual's audiogram results?

Reviewing the audiogram, we see the following patterns for the left (L't) and right (R't) ears:

- The left ear shows thresholds within normal limits (WNL) up to 2000 Hz, with a drop at 8000 Hz.
- The right ear displays thresholds that start in the mild hearing loss range and progress to severe within the speech frequencies, with a prof[illegible]d loss noted at 8000 Hz. The presence of air-bone gaps, particularly at 500 Hz and 1000 Hz, indicates a conductive component, hence a mixed hearing loss (a combination of sensorineural and conductive hearing loss).

Given these observations, the correct interpretation of the audiogram would be:

(B) L't WNL except 8k Hz; R't mild to profound mixed type HL

Question 46: What is the rollover index (RI) for the right ear of this individual?

The rollover index (RI) is calculated using the formula RI = (1 - (WRS at highest intensity/WRS at most comfortable level)). This index is used to evaluate the possibility of retrocochlear pathology, with higher values suggesting a greater likelihood of such pathology.

Looking at the provided data:

- The Word Recognition Score (WRS) at the most comfortable level (50 dB HL) is 64%.
- The WRS at a higher intensity (70 dB HL) is 40%.

Using the RI formula: RI = 1 - (40/64) = 1 - 0[illegible]5 = 0.375

When rounding to two decimal places, the RI would be:

(B) 0.38

Question 47: What is the most likely problem present in the right ear of this individual?

Considering the audiogram and additional clinical information:

- The audiogram shows a mixed type hearing loss in the right ear, which suggests both a sensorineural and conductive component.
- There's a significant drop in word recognition performance at a higher intensity (rollover phenomenon), which could indicate a retrocochlear problem.
- The patient complains of aural fullness but no pain or vertigo, which may point away from typical middle ear pathology.
- The significant tinnitus and the disparity in clarity between the ears are also considered.

Combining these clues, the most likely pro[illegible]n for the right ear, given the audiometric data and the patient's symptoms, would be:

(C) Retrocochlear pathology

Retrocochlear pathology includes problems beyond the cochlea, such as the auditory nerve or brainstem, which can sometimes present as a mixed hearing loss on an audiogram and is often associated with a rollover effect in speech audiometry.

You

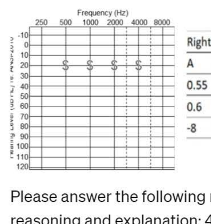

Right
A
0.55
0.6
-8

Acoustic Reflex Thresholds

EAR		
Probe	Tone	500
R	R	85
R	L	
L	L	NR
L	R	

Please answer the following multiple-choi[illegible]uestion as a hearing care professional, providing reasoning and explanation: 48 菲菲現年 28 個月，媽媽覺得菲菲是女孩，說話應該比較早，但詞彙量好像比哥哥 2 歲多時還略少一些，故到醫院檢查。菲菲在聽檢室內會不停的將插入式耳機拔出來，你做了各種嘗試後，得到如下所示的檢查結果。下列何者是最合宜的判斷及處置？

（A）菲菲雙耳聽力沒有問題，暫時不需要處置，若日後有疑慮再來檢查
（B）菲菲可能有單側傳導性聽損，轉介給耳鼻喉科醫師做進一步診斷
（C）菲菲可能有單側感音性聽損，應立即加測對側聲反射，以利判斷
（D）檢查結果不一致無法判斷，應儘速安排 OAE、ABR 等電生理檢查

ChatGPT

Based on the provided audiogram and tympanometry results, let's analyze Fei Fei's hearing assessment:

The audiogram shows normal hearing thresholds at all tested frequencies (250 Hz to 8000 Hz) for both ears, indicated by the 'O' symbols falling within the normal range (≤20 dB HL).

The tympanometry results show a Type A tympanogram for both ears, which suggests normal middle ear pressure and compliance. This [illegible]cally indicates that there is no middle ear pathology, such as fluid or infection, that would cause a conductive hearing loss.

The acoustic reflex thresholds are present at fairly high levels in the right ear when it is both the probe ear and the contralateral ear. There are no responses marked for the left ear when it is the probe ear, indicated by "NR" (no response). The absence of acoustic reflexes could be due to various reasons, including the child's cooperation, equipment limitations, or a potential issue with the reflex arc. However, given the normal hearing thresholds and tympanograms, a profound pathology is less likely.

Considering the information:

(A) Fei Fei's bilateral hearing does not appear to have a problem based on the audiogram, and no immediate action seems necessary. However, it is important to consider the mother's concerns about speech and language development, which can be influenced by factors other than hearing, such as environmental, developmental, or speech/language disorders.

(B) Fei Fei does not seem to have a conductive hearing loss since the tympanogram is Type A, which would not suggest a referral to an ot[illegible]yngologist based solely on these results.

(C) There is no indication of sensorineural hearing loss in the audiogram, so immediate further testing for contralateral acoustic reflexes may not be necessary.

(D) The inconsistency mentioned might refer to the difficulty in obtaining reliable acoustic reflex thresholds, but the audiogram is consistent and suggests normal hearing. However, given the concerns about language development and the possible difficulty in obtaining accurate results due to the child's behavior, electrophysiological tests like Otoacoustic Emissions (OAE) and Auditory Brainstem Response (ABR), which do not require behavioral responses, could be considered for a more comprehensive evaluation.

Given the context, the most appropriate decision and action would be:

(D) "The results are inconsistent and cannot be determined; an OAE and ABR or other electrophysiological tests should be arranged promptly."

This would ensure a comprehensive assessment of Fei Fei's auditory system is completed, considering the mother's concerns about [illegible]anguage development and the challenges faced during the test. It's also essential to monitor Fei Fei's language development and consider a speech-language evaluation if concerns persist.

You

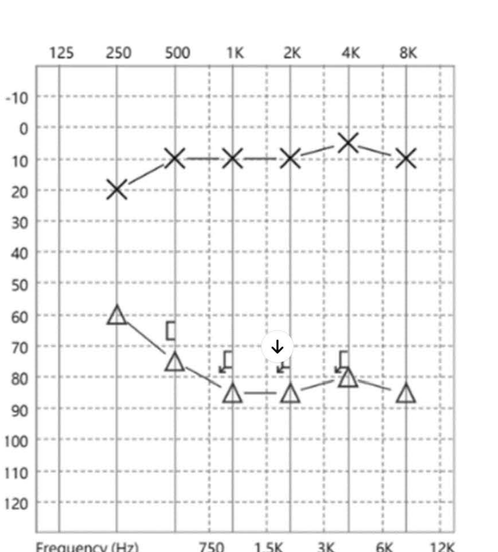

Please answer the following multiple-choice question as a hearing care professional, providing reasoning and explanation: 49 劉小姐 27 歲，已被診斷為右耳聽神經瘤 4 公分。純音聽力如下圖所示。依據美國聽語學會（ASHA, 1988）對語音辨識閾（SRT）和語音察覺閾（SDT）的描述，劉小姐的語音聽力檢查結果，下列何者最適當？
（A）右耳 SRT 5 dB HL，左耳 SRT 55 dB HL （B）右耳 SRT 10 dB HL，左耳 SDT 100 dB HL
（C）右耳 SRT 60 dB HL，左耳 SRT 5 dB HL （D）右耳 SDT 75 dB HL，左耳 SRT 10 dB HL
50 承上題，由純音聽閾和語音測試的相關性，推估劉小姐的單字詞聽辨率（WRS）結果，下列何者正確？
（A）右耳 96%，左耳 12% （B）右耳 20%，左耳 60% （C）右耳 8%，左耳 100% （D）右耳 88%，左耳 92%

ChatGPT

To answer these questions, I will need to analyze the audiogram provided. However, I cannot view images directly. Based on a description, if the audiogram indicates a significant hearing loss in one ear consistent with a diagnosis of an acoustic neuroma (vestibular schwannoma), typically the ear with the tumor would show a poorer threshold compared to the other ear.

Question 49:

Speech Recognition Threshold (SRT) is the lowest level at which speech can not only be detected but also recognized and repeated back correctly by the listener. For a large acoustic neuroma, we would expect the SRT to be significantly elevated in the affected ear. The Speech Detection Threshold (SDT) is the level at which speech can be detected but not necessarily understood or recognized.

Given a right ear acoustic neuroma:

(A) This option would not be appropriate a[illegible]e right ear would likely have a much higher (worse) SRT due to the tumor.
(B) This option seems more plausible as the right ear would have a poorer SRT and the left ear, if unaffected, would have a normal or near-normal SRT.
(C) This option is contradictory to the typical presentation of acoustic neuroma, as the affected ear would not have a better SRT than the unaffected ear.
(D) The SDT being 75 dB HL for the right ear suggests significant hearing loss, which might be consistent with the presence of an acoustic neuroma, but the SRT is not likely to be better than the SDT.

Based on the general patterns associated with acoustic neuroma, the most appropriate option, without seeing the actual audiogram, would likely be:

(B) "Right ear SRT 10 dB HL, Left ear SDT 100 dB HL"

This assumes a typographical error in the options provided, as typically the SDT will be better (a lower dB value) than the SRT, since detection is a lower level of auditory processing than recognition.

Question 50:

Word Recognition Score (WRS) is the ability to correctly repeat back words that are presented at a comfortable listening level above the SRT. A large acoustic neuroma would likely result in a significantly reduced WRS in the affected ear due to distortion and reduced clarity of sound. The unaffected ear would likely have a WRS that is close to 100% if the hearing is within normal limits or near-normal.

Given a right ear acoustic neuroma, the WRS might be:

(A) This is unlikely as the affected ear would not perform better than the unaffected ear.
(B) This might be possible if the right ear is affected by the tumor and has a significant loss, while the left ear has a moderate loss.
(C) This is likely the correct pattern, with the right ear (affected by the tumor) having a very poor WRS and the left ear having a perfect or near-perfect WRS.
(D) This option is incorrect as it suggests better hearing in the affected ear than the unaffected ear, which is inconsistent with the presence of a large acoustic neuroma.

Without the actual audiogram and speech audiometry results, the most likely correct option would be:

(C) "Right ear 8%, left ear 100%"

This assumes the right ear is significantly impaired due to the tumor, leading to a very low WRS, and the left ear is unaffected with a normal WRS.

Message ChatGPT...
ChatGPT can make mistakes. Consider checking important information.
