# Supplementary material for: Exploring the Performance of ChatGPT-4 in the Taiwan Audiologist Qualification Examination: Preliminary Observational Study Highlighting the Potential of AI Chatbots in Hearing Care
Source: JMIR Med Educ. 2024 Apr 26;10:e55595. doi: 10.2196/55595 (PMC11067446; doi:10.2196/55595)
Supplement: Multimedia Appendix 3 [file mededu-v10-e55595-s003.pdf]

You

Please answer the following multiple-choice question as a hearing care professional, providing reasoning and explanation: 1 有關聽覺障礙患者的特徵，下列敘述何者最為適切?
（A）王祥是大學時期因為腦傷導致聽損，其語音辨識能力與先天聽損者無異
（B）小江是屬於感音型聽損，聽到的某些聲音可能被扭曲
（C）小陳為耳硬化症患者，屬於習語前感音型聽損患者
（D）李爺爺是屬於老化型聽損，最主要是屬於傳導型聽損

ChatGPT

為了解答這個關於聽覺障礙的選擇題，我們需要考慮每個選項中描述的情況，並對應到聽力學的知識。

（A）王祥是大學時期因為腦傷導致聽損，其語音辨識能力與先天聽損者無異

- 腦傷可能導致聽損，但其影響的程度和類型可以因人而異。語音辨識能力受到多種因素影響，包括聽損的類型、傷害的區域、以及患者的康復和調適能力。因此，無法一概而論地說腦傷導致的聽損患者的語音辨識能力會與先天聽損者相同。

（B）小江是屬於感音型聽損，聽到的某些聲音可能被扭曲

- 這是對的。感音型聽損通常是由於內耳或聽神經的問題造成，這可能會導致聲音的扭曲或不清晰，尤其是在嘈雜的環境中。

（C）小陳為耳硬化症患者，屬於習語前感音型聽損患者

- 耳硬化症通常導致傳導型聽損，而非感音型聽損。它影響的是中耳結構，尤其是骨鏈，而不是內耳或聽神經。

（D）李爺爺是屬於老化型聽損，最主要是屬於傳導型聽損

- 老化型聽損，或稱為老年性聽力下降，通常是感音型聽損。它與年齡相關，主要影響內耳的結構和功能。

基於上述分析，最適當的選項是（B）小江是屬於感音型聽損，聽到的某些聲音可能被扭曲。

You

please give answers in english

ChatGPT

To answer the multiple-choice question regarding characteristics of patients with hearing impairments, let's consider each option:

(A) Wang Xiang suffered hearing loss due to a brain injury in college, and his speech recognition ability is no different from someone with congenital hearing loss.

- Brain injuries can lead to hearing loss, but the extent and type of impairment can vary. Speech recognition ability is influenced by several factors, including the type and severity of hearing loss, the area of the brain affected, and the individual's rehabilitation and adaptation capabilities. It's not accurate to generalize that someone with hearing loss due to brain injury will have the same speech recognition ability as someone with congenital hearing loss.

(B) Xiao Jiang has sensorineural hearing loss, and some sounds may be distorted.

- This is a correct statement. Sensorineural [illegible]earing loss usually results from problems in the inner ear or auditory nerve, leading to distortion or lack of clarity in sounds, especially in noisy environments.

(C) Xiao Chen has otosclerosis and is considered a patient with pre-lingual sensorineural hearing loss.

- Otosclerosis typically leads to conductive hearing loss, not sensorineural. It affects the middle ear structures, especially the ossicles (small bones in the ear), not the inner ear or auditory nerve.

(D) Grandpa Li has age-related hearing loss, primarily conductive in nature.

- Age-related hearing loss, or presbycusis, is usually sensorineural, not conductive. It is associated with aging and mainly affects the structures and function of the inner ear.

Based on this analysis, the most appropriate option is (B) Xiao Jiang has sensorineural hearing loss, and some sounds may be distorted.

You

Please answer the following multiple-choice question as a hearing care professional, providing reasoning and explanation: 2 關於特定型語言障礙（specific language impairment）兒童的典型特徵，下列敘述何者最不適切?
（A）智力和工作記憶的缺損
（B）視覺任務呈現執行功能的缺損
（C）社交技能不佳
（D）腦造影檢查通常呈現大腦語言處理區域的左右對稱性
3 關於「溝通障礙」之概念，下列敘述何者最不適切?
（A）溝通障礙會影響個體接收和傳遞訊息的能力
（B）溝通障礙不至於影響手勢的溝通表現
（C）溝通障礙可能影響個體的聽能、語言或言語處理
（D）溝通障礙不包含溝通差異（communication differences）

ChatGPT

為了回答這些關於語言障礙和溝通障礙的選擇題，我們將逐一分析每個選項：

關於特定型語言障礙（SLI）兒童[illegible]型特徵：

(A) 智力和工作記憶的缺損

- 特定型語言障礙（SLI）的孩童通常智力正常，且工作記憶的缺損並非SLI的典型特徵。SLI主要影響的是語言能力，而非智力或工作記憶。

(B) 視覺任務呈現執行功能的缺損

- SLI孩童在某些非語言性的執行功能上可能會有缺損，但這不是最典型的特徵。視覺任務的執行功能缺損更多與其他類型的學習障礙相關。

(C) 社交技能不佳

- 社交技能不佳可以是SLI兒童的一個特徵，但這通常是因為他們在語言溝通方面的挑戰，而非社交技能本身的缺陷。

(D) 腦造影檢查通常呈現大腦語言處理區域的左右對稱性

- SLI兒童的大腦語言處理區域可能會有特殊[illegible]神經生物學特徵，但左右對稱性不是典型的特徵。這個描述不夠明確，需要更多的研究來確定。

最不適當的選項是（A）智力和工作記憶的缺損，因為這不是SLI兒童的典型特徵。

關於「溝通障礙」之概念：

(A) 溝通障礙會影響個體接收和傳遞訊息的能力

- 這是對的。溝通障礙影響個體在接收和表達語言、言語、非語言性溝通等方面的能力。

(B) 溝通障礙不至於影響手勢的溝通表現

- 實際上，溝通障礙可能會影響手勢和其他非語言性溝通方式。手勢是溝通的重要組成部分，特別是對於那些言語能力受限的人。

(C) 溝通障礙可能影響個體的聽能、語言或言語處理

- 這也是對的。溝通障礙可以包括聽力問題、語言理解和表達困難，以及言語聲音和節奏的問題。

(D) 溝通障礙不包含溝通差異（communica[illegible] differences）

- 這是錯誤的。溝通障礙和溝通差異是不同的概念。溝通障礙指的是功能上的限制，而溝通差異則是指不同文化或語言背景下的正常溝通方式差異。

最不適當的選項是（D）溝通障礙不包含溝通差異（communication differences），因

You

please give all answers in English

ChatGPT

Certainly, let's analyze the options in English for the questions regarding specific language impairment (SLI) in children and the concept of communication disorders.

Specific Language Impairment (SLI) in Children:

(A) Deficits in intelligence and working memory

- Children with SLI typically have normal inte[illegible]gence, and deficits in working memory are not a classic feature of SLI. SLI primarily affects language abilities, not intelligence or working memory.

(B) Deficits in executive functioning in visual tasks

- While some children with SLI may have deficits in certain non-linguistic executive functions, this is not the most typical feature. Deficits in executive functioning for visual tasks are more commonly associated with other types of learning disabilities.

(C) Poor social skills

- Poor social skills can be a feature in children with SLI, but this is usually due to their challenges in language communication, rather than a direct deficiency in social skills themselves.

(D) Brain imaging typically shows symmetry in the brain's language processing areas

- Children with SLI might have unique neurobiological characteristics in the brain's language processing areas, but symmetry is not a typical feature. This description is not clear and requires more research for confirmation.

The least appropriate option is (A) Deficits in intelligence and working memory, as it is not a typical feature of children with SLI.

Concept of Communication Disorders:

(A) Communication disorders affect an individual's ability to receive and convey messages

- This is correct. Communication disorders impact the ability to receive and express language, speech, and non-verbal communication.

(B) Communication disorders do not affect gestural communication performance

- In reality, communication disorders can impact gestural and other forms of non-verbal communication. Gestures are an important part of communication, especially for those with limited verbal abilities.

(C) Communication disorders can affect an individual's hearing, language, or speech processing

- This is also correct. Communication disorders can include hearing problems, difficulties in understanding and expressing language, and issues with the sound and rhythm of speech.

(D) Communication disorders do not include communication differences

- This is incorrect. Communication disorders and communication differences are distinct concepts. Communication disorders refer to functional limitations, whereas communication differences refer to normal variations in communication styles due to different cultural or linguistic backgrounds.

The least appropriate option is (D) Communication disorders do not include communication differences, as it incorrectly conflates the two distinct concepts.

You

Please answer the following multiple-choice question as a hearing care professional, providing reasoning and explanation: 4 兒童在發展語音或閱讀能力的過程中會逐漸發展出音韻覺識能力，下列何者最能表示兒童已發展出音韻覺識能[illegible]
（A）兒童能聽出「沙」和「叉」是不同的[illegible] （B）兒童聽到小鳥在叫知道那是鳥的叫聲
（C）兒童說：「豆子和肚子都有ㄉ的聲音」 （D）兒童能仿說「弟弟好棒」四個字
5 關於嗓音的音高與音量，下列敘述何者最不適切?
（A）一般對話（如餐桌上的談話）的音量，平均約 60 dB
（B）肺部氣壓每增加一倍，音量會增加 2～4 dB
（C）成年男性的平均基頻約 125 Hz，代表聲帶振動每秒 125 次
（D）聲帶振動的速率會影響嗓音的音高

ChatGPT

為了解答這些關於兒童語音發展和嗓音的音高與音量的問題，我們將逐一分析每個選項：

4. 兒童發展音韻覺識能力的表示：

(A) 兒童能聽出「沙」和「叉」是不同的語音

- 這個選項直接涉及到音韻覺識的能力，即兒童能夠辨識和區分不同的語音。這是音韻覺識的一個關鍵指標。

(B) 兒童聽到小鳥在叫知道那是鳥的叫聲

- 這更多涉及到聲音識別能力而非音韻覺識。音韻覺識與語言的聲音有關，與動物的叫聲辨識不直接相關。

(C) 兒童說：「豆子和肚子都有ㄉ的聲音」

- 這也是音韻覺識的一個

You

Please answer the following multiple-choice question as a hearing care professional, providing reasoning and explanation: 4 兒童在發展語音或閱讀能力的過程中會逐漸發展出音韻覺識能力，下列何者最能表示兒童已發展出音韻覺識能力?
（A）兒童能聽出「沙」和「叉」是不同的語音 （B）兒童聽到小鳥在叫知道那是鳥的叫聲
（C）兒童說：「豆子和肚子都有ㄉ的聲音」 （D）兒童能仿說「弟弟好棒」四個字
5 關於嗓音的音高與音量，下列敘述何者最不適切?
（A）一般對話（如餐桌上的談話）的音量，平均約 60 dB
（B）肺部氣壓每增加一倍，音量會增加 2～4 dB
（C）成年男性的平均基頻約 125 Hz，代[illegible]振動每秒 125 次
（D）聲帶振動的速率會影響嗓音的音高

ChatGPT

To answer these multiple-choice questions as a hearing care professional, let's analyze each option:

4. Indicators of Phonological Awareness Development in Children:

(A) The child can hear that "沙" (sha) and "叉" (cha) are different sounds

- This option directly relates to phonological awareness, which is the ability to recognize and differentiate between distinct sounds in language. This is a key indicator of phonological awareness.

(B) The child hears a bird chirping and knows it's a bird sound

- This is more about sound recognition rather than phonological awareness. Phonological awareness is related to the sounds of lan[illegible]e, not identifying animal sounds.

(C) The child says: "豆子 (dou zi) and 肚子 (du zi) both have the sound ㄉ"

- This is also an indication of phonological awareness. The child is recognizing and verbalizing the common phonetic component in different words, which

You

Please answer the following multiple-choice question as a hearing care professional, providing reasoning and explanation: 4 兒童在發展語音或閱讀能力的過程中會逐漸發展出音韻覺識能力，下列何者最能表示兒童已發展出音韻覺識能力?
（A）兒童能聽出「沙」和「叉」是不同的語音 （B）兒童聽到小鳥在叫知道那是鳥的叫聲
（C）兒童說：「豆子和肚子都有ㄉ的聲音」 （D）兒童能仿說「弟弟好棒」四個字
5 關於嗓音的音高與音量，下列敘述何者最不適切?
（A）一般對話（如餐桌上的談話）的音量，平均約 60 dB
（B）肺部氣壓每增加一倍，音量會增加 2～4 dB
（C）成年男性的平均基頻約 125 Hz，代表聲帶振動每秒 125 次
（D）聲帶振動的速率會影響嗓音的音高

ChatGPT

Let's analyze these options:

4. Indicators of Phonological Awareness in Children:

(A) The child can hear that "沙" (sha) and "叉" (cha) are different sounds.

- This demonstrates phonological awareness, as the child can distinguish between different phonemes (basic units of sound).

(B) The child hears a bird chirping and knows it's a bird sound.

- This is more related to general sound recognition, not specifically phonological awareness in language.

(C) The child says: "豆子 (dou zi) and 肚子 (du zi) both have the sound ㄉ."

- This shows an awareness of common phonetic elements in different words, indicating phonological awareness.

(D) The child can imitate the phrase "弟弟好棒" (di di hao bang - "little brother is great").

- Imitating a phrase indicates speech development but does not specifically demonstrate phonological awareness.

The best indicator of phonological awareness among these options is (C), where the child recognizes and articulates a common phonetic component in different words.

5. Statements about Voice Pitch and Volume:

(A) The average volume of normal conversation (like at a dinner table) is about 60 dB.

- This is a reasonable estimate for conversation volume.

(B) Lung pressure doubling results in a 2-4 dB increase in volume.

- This statement simplifies the relationship between lung pressure and volume, which can be more complex in reality.

(C) The average fundamental frequency for adult males is about 125 Hz, representing 125 vocal fold vibrations per second.

- This is a typical fundamental frequency range for adult males and correctly describes the relation to vocal fold vibration rate.

(D) The rate of vocal fold vibration affects the pitch of the voice.

- This is correct; the pitch of the voice is directly related to the frequency of vocal fold vibrations.

The least appropriate statement is (B). The relationship between lung pressure and sound volume is not as straightforward as doubling lung pressure resulting in a specific dB increase in volume. The increase in volume due to increased lung pressure can vary greatly depending on several factors, including vocal tract configuration and individual physiological differences.

You

Please answer the following multiple-choic[illegible] question as a hearing care professional, providing reasoning and explanation: 6 下列有關吶吃（dysarthria）的敘述，何者最不適切?
（A）為概括性診斷名稱
（B）語法結構不佳
（C）詞彙及閱讀能力均佳
（D）控制言語產生肌肉中樞或周邊神經系統受到干擾所致

7 有關溝通障礙的治療作法，下列敘述何者最不適切?
（A）不一定每次都為個案示範目標行為
（B）視情況提示個案應有的反應或回答
（C）非口語提示比口語提示更能促進語言結構的學習
（D）給予增強或回饋，但需適時褪除

ChatGPT

Let's analyze these options related to dysarthria and the treatment of communication disorders:

6. Statements about Dysarthria:

(A) It is a general diagnostic term.

- This is correct. Dysarthria refers to a group of motor speech disorders caused by impairments in the muscles used for speech.

(B) Poor grammatical structure.

- Dysarthria primarily affects the physical production of speech, not the grammatical structure of language. It's more about speech clarity and control rather than language structure.

(C) Good vocabulary and reading abilities.

- This is often true. Dysarthria impacts speech production but does not necessarily affect cognitive abilities like vocabulary and reading.

(D) Caused by disturbances in the central or peripheral nervous system that control the muscles for producing speech.

- This is an accurate description of dysarthria, which is caused by neurological impairments affecting speech muscle control.

The least appropriate statement is (B) Poor grammatical structure, as dysarthria affects the physical aspects of speech rather than the linguistic constructs like grammar.

7. Treatment Approaches for Communication Disorders:

(A) It's not necessary to demonstrate the target behavior every time for the client.

- This can be true in therapy. While modeling is important, it's not always required in every session, depending on the client's level and therapy goals.

(B) Provide cues for the appropriate response or answer, depending on the situation.

- This is a common practice in therapy, where the therapist provides prompts or cues to guide the client.

(C) Non-verbal cues are more effective than verbal cues in promoting the learning of language structure.

- This statement is not universally true and depends on the individual case. Both verbal and non-verbal cues can be effective, and their use depends on the specific needs of the client.

(D) Provide reinforcement or feedback, but it should be faded appropriately.

- This is a standard practice in therapy. Reinforcement and feedback are crucial, but therapists also work towards reducing their use as the client gains independence.

The least appropriate statement is (C) Non-verbal cues are more effective than verbal cues in promoting the learning of language structure, as it oversimplifies the complexity of therapy approaches and does not apply universally to all cases.

You

Please answer the following multiple-choice question as a hearing care professional, providing reasoning and explanation: 8 華語聲母的語音可以根據構音位置、發音方式、喉頭是否震動加以分類，下列描述何者最為適切?
（A）發ㄈ音時，構音位置位於下唇與上齒 [illegible]屬於雙唇音
（B）發ㄊ音時，發音方式為氣流受口腔某[illegible]分的阻塞，屬於塞音
（C）發ㄌ音時，發音方式為口腔裡某兩部分互相接近，氣流從狹縫裡摩擦出來，屬於擦音
（D）發ㄖ音時，構音位置為舌尖前與前硬顎，屬於舌尖前音
9 專業團隊進行輔助溝通系統使用的評估時，需考量使用者的那些能力? ①語言能力 ②社交能力 ③操作能力 ④策略運用能力
（A）僅①③ （B）僅①②③ （C）僅①②④ （D）①②③④

ChatGPT

Let's analyze these options regarding Mandarin Chinese initials and the assessment of Augmentative and Alternative Communication (AAC) systems:

8. Classification of Mandarin Chinese Initials:

(A) When producing the sound ㄈ, the articulation is between the lower lip and upper teeth, categorized as a bilabial sound.

- This is incorrect. The sound ㄈ (pinyin: f) in Mandarin is actually a labiodental sound, produced by the contact of the lower lip and the upper teeth.

(B) When producing the sound ㄊ, the method of articulation involves the airflow being blocked by two parts of the mouth, categorized as a plosive sound.

- This is correct. The sound ㄊ (pinyin: t) is a plosive (or stop) sound, made by stopping airflow and then releasing it.

(C) When producing the sound ㄌ, the method of articulation involves the close approximation of two parts of the mouth, with airflow creating friction through a narrow gap, categorized as a fricative sound.

- This is incorrect. The sound ㄌ (pinyin: l) is an alveolar nasal, where the airflow is directed through the nose, not a fricative.

(D) When producing the sound ㄖ, the articulation is between the front part of the tongue and the hard palate, categorized as an apical sound.

- This is correct. The sound ㄖ (pinyin: r) is an apical sound, produced with the tongue tip near the hard palate.

The most appropriate description is (B) When producing the sound ㄊ, the method of articulation involves the airflow being blocked by two parts of the mouth, categorized as a plosive sound.

9. Assessment of AAC System Usage by a Professional Team:

The assessment should consider the user's abilities in:

(A) Only language and operational abilities.
(B) Only language, social, and operational abilities.
(C) Only language, social, and strategic abilities.
(D) Language, social, operational, and strategic abilities.

When assessing the use of AAC systems, it's important to consider all aspects of communication, including:

1. Language Ability: Understanding and using language.
2. Social Ability: Engaging in social interactions.
3. Operational Ability: Physically operating t[illegible]AC device or system.
4. Strategic Ability: Effectively using the system to communicate in various situations.

Therefore, the most appropriate option is (D) Language, social, operational, and strategic abilities. All these aspects are crucial for successful AAC system use.

You

Please answer the following multiple-choice question as a hearing care professional, providing reasoning and explanation: 10 有關溝通障礙介入原則的敘述，下列何者最不適切?
（A）介入係以增進個案的溝通技能為最高目標
（B）介入目標應關注個案能自動化表現出所學的溝通行為
（C）介入應針對缺失部分而非整體能力
（D）實證本位介入可確保謹慎且符合專業倫理的服務
11 下列何者為多數口吃者的好發時期?
（A）成人期 （B）青少年期 （C）學齡期 （D）幼兒期

ChatGPT

Let's analyze these options regarding the [illegible]ciples of intervention for communication disorders and the typical onset period for stuttering:

10. Statements about Intervention Principles for Communication Disorders:

(A) The highest goal of intervention is to improve the individual's communication skills.

- This is correct. The primary aim of intervention in communication disorders is to enhance communication abilities.

(B) Intervention goals should focus on the individual's ability to automatically demonstrate learned communication behaviors.

- This is also correct. Effective intervention aims to make the learned communication skills automatic and natural for the individual.

(C) Intervention should target specific deficits rather than overall abilities.

- This statement might be misleading. While it's important to address specific deficits, considering the individual's overall communication ab[illegible]s is also crucial for a holistic approach.

(D) Evidence-based intervention ensures careful and ethically sound services.

- This is true. Evidence-based practices are fundamental in providing interventions that are both effective and ethically responsible.

The least appropriate statement is (C) Intervention should target specific deficits rather than overall abilities. A comprehensive approach that considers the overall communication abilities of the individual is generally more effective.

11. Typical Onset Period for Most People Who Stutter:

(A) Adulthood
(B) Adolescence
(C) School Age
(D) Early Childhood

Stuttering typically begins in early childho[illegible] Most children who stutter start showing signs of stuttering between the ages of 2 and 5 year[illegible] This is a critical period for speech and language development.

Therefore, the correct answer is (D) Early Childhood. This is the period when stuttering commonly begins for the majority of individuals who experience it.

You

Please answer the following multiple-choice question as a hearing care professional, providing reasoning and explanation: 12 有關耳聲傳射（otoacoustic emissions, OAE），下列敘述何者最不適切?
（A）是因內耳外毛細胞移動所產生的中高能量聲音，通常也當作回音
（B）從內耳傳到中耳再到外耳
（C）使用中強度的聲音刺激外耳道時，若有出現反應，則為聽力正常或僅是輕度以下聽損
（D）可使用於新生兒聽力篩檢
13 關於突發性聽力損失（sudden hearing loss）的診斷與描述，下列敘述何者最不適切?
（A）臨床聽力檢查判斷是以三日內 0.5 kHz、1 kHz、2 kHz 的純音平均聽力閾值上升超過 35 分貝為依據
（B）此病症好發於中年，患者常常一覺醒[illegible]然發現一側耳朵被摀住聽不清楚
（C）聽力損失的範圍從輕度至極重度都有[illegible]
（D）有些患者在兩週內聽力可部分或完全恢復

ChatGPT

Let's analyze these options related to otoacoustic emissions (OAE) and the diagnosis and characteristics of sudden hearing loss:

12. Statements about Otoacoustic Emissions (OAE):

(A) OAEs are medium to high energy sounds produced by the movement of outer hair cells in the inner ear, often regarded as echoes.

- This is correct. OAEs are sounds generated by the vibrations of the outer hair cells in the cochlea.

(B) OAEs travel from the inner ear to the middle ear and then to the outer ear.

- This is also correct. The emissions generated in the inner ear travel back through the middle ear to the outer ear, where they can be measur[illegible].

(C) When stimulated with moderate intensity sounds in the ear canal, the presence of a response indicates normal hearing or mild hearing loss at most.

- This is true. OAEs are typically present in individuals with normal hearing or mild hearing loss. Their absence can indicate hearing impairment.

(D) OAEs can be used for newborn hearing screening.

- This is correct. OAEs are commonly used in newborn hearing screenings as they are non-invasive and can be measured passively.

The least inappropriate statement is (C). While the presence of OAEs generally indicates normal hearing or mild hearing loss, it's important to note that their absence doesn't always confirm hearing loss, as they can be absent in cases of severe ear canal blockage or middle ear pathology.

13. Statements about Sudden Hearing Loss:

(A) Clinical audiometric evaluation for diag[illegible]sis is based on a rise in the pure tone average hearing threshold over 35 decibels within t[illegible]e days at frequencies of 0.5 kHz, 1 kHz, and 2 kHz.

- This is a standard criterion for diagnosing sudden hearing loss.

(B) The condition often occurs in middle age, and patients frequently wake up to find one ear suddenly muffled or unclear.

- This is also true. Sudden hearing loss often affects one ear and patients may notice it upon waking.

(C) The range of hearing loss can vary from mild to profound.

- This is correct. Sudden hearing loss can range in severity.

(D) Some patients may experience partial or complete recovery of hearing within two weeks.

- This is true. Spontaneous recovery can occur in some cases of sudden hearing loss.

The least inappropriate statement is (A). T[illegible]criterion for diagnosing sudden hearing loss is based on a significant, rapid decrease in hea[illegible]ing ability, often defined as a 30 dB or greater loss in hearing sensitivity over three contiguous audiometric frequencies within a 72-hour period. The specific frequencies and the amount of hearing loss (35 dB vs 30 dB) may vary slightly in different diagnostic criteria, but the general concept is correct.

‹ 2 / 2 ›

You

Please answer the following multiple-choice question as a hearing care professional, providing reasoning and explanation: 14 急性中耳炎（acute otitis media）常好發於學齡前兒童，關於此病症的影響與描述，下列何者最不適切?
（A）幼童時期因為耳咽管關閉功能不完全，且連結到鼻咽腔的角度也較水平所導致
（B）常見的幼童症狀為突然的耳痛，且影響到日常生活與睡眠
（C）其感染源可能為病毒或是細菌，通常病程少於 21 天
（D）通常建議立即使用鼓膜切開術或是放置通氣管（grommet）來平衡中耳腔壓力

15 當聽閾值在 16 至 25 分貝範圍內，兒童於言語和語言發展過程會遺漏某些細微的聲學訊息，但大人則會運用知識和經驗，自行填補遺漏的訊息。此過程稱為下列何者?
（A）聽覺閉鎖（auditory closure） （B）聽覺開放（auditory opening）
（C）聽覺想法（auditory idea） （D）[illegible]漏（auditory missing）

ChatGPT

Let's analyze these options regarding acute otitis media and the concept related to auditory processing:

14. Statements about Acute Otitis Media in Pre-School Children:

(A

(A) Acute otitis media in young children is often due to incomplete closure function of the eustachian tube, and the angle connected to the nasopharynx is more horizontal.

- This is correct. In young children, the eustachian tube is more horizontal and less functional, leading to an increased risk of acute otitis media.

(B) Common symptoms in young children include sudden ear pain, affecting daily life and sleep.

- This is also correct. Acute ear pain is a co[illegible]on symptom of acute otitis media, and it can significantly affect a child's daily activities and sleep.

(C) The infection can be caused by viruses or bacteria and usually lasts less than 21 days.

- This statement is accurate. Acute otitis media can be caused by both viruses and bacteria, and its duration is typically short.

(D) Immediate use of myringotomy or the insertion of grommets (ventilation tubes) to balance the pressure in the middle ear is usually recommended.

- This is not always the case. The decision to perform myringotomy or insert grommets depends on the severity and frequency of the infections, as well as the response to other treatments. These interventions are not typically the first line of treatment.

The least appropriate statement is (D) Immediate use of myringotomy or the insertion of grommets to balance the pressure in the middle ear is usually recommended, as this is not the standard initial treatment approach for acute otitis media.

15. Concept for Auditory Processi[illegible] with Hearing Thresholds between 16 to [illegible]

25 Decibels:

(A) Auditory closure
(B) Auditory opening
(C) Auditory idea
(D) Auditory missing

The term "auditory closure" refers to the ability to fill in missing auditory information, a skill that adults often use to comprehend speech even when some sounds are missing or distorted. Children, especially those with mild hearing loss, may struggle with this, missing subtle acoustic cues that are crucial for language development.

Therefore, the correct term is (A) Auditory closure. It describes the process where adults, using their knowledge and experience, can fill in missing auditory information, a skill that may be less developed in children.

You

Please answer the following multiple-choice question as a hearing care professional, providing reasoning and explanation: 16 對於聽力損失學生的教學策略，下列敘述何者最不適切？
（A）在教室上課時，要儘量讓學生清楚看到老師的表情
（B）學生聽不懂時，可以用寫下來的方式，有助其理解
（C）減少同儕教導的模式，以避免標示學生的障礙
（D）應減少環境噪音，例如可加裝窗簾
17 有關長時間噪音暴露所導致的噪音型聽力損失，可從生理與變化的時效性質及後續效應探討，下列敘述何者最不適切？
（A）因為中耳聽反射（acoustic reflex）有潛時（latency）與反射適應（reflex adaptation）的現象，所以對於高頻的噪音保護功能會顯不足
（B）噪音導致的聽力衰退在某些情形下只是暫時的，即休息後便可復原，稱之為暫時性聽閾升高（temporary threshold shift, TTS）
（C）聽力衰退原因如果是長時間暴露於高音量下所導致，其噪音類型通常是會造成不愉悅的噪音（如工廠機具、交通工具）而非令人舒暢的音樂（如樂團演奏）
（D）噪音型聽力損失在聽力方面所呈現的特質多半是雙側且對稱的感音神經性聽力損失，但是初期很少會直接造成極重度聽力損失等級

ChatGPT

Let's analyze these options regarding teaching strategies for students with hearing loss and the effects of prolonged noise exposure on hearing:

16. Teaching Strategies for Students with Hearing Loss:

(A) Ensure the student can clearly see the teacher's face during classroom lessons.

- This is appropriate as visual cues like lip-reading and facial expressions are important for students with hearing loss.

(B) If the student does not understand, writing things down can help them comprehend.

- This is also a suitable strategy. Written communication can aid understanding when auditory information is not fully accessible.

(C) Reduce peer teaching models to avoid highlighting the student's disability.

- This statement might be less appropriate. Peer teaching can be beneficial for inclusive education and social interaction. The key is to imple[illegible]t it sensitively and appropriately, ensuring it doesn't inadvertently isolate or stigmatize the student with hearing loss.

(D) Reduce environmental noise, such as by installing curtains.

- This is a good strategy. Minimizing background noise can help students with hearing loss focus better.

The least appropriate statement is (C) Reduce peer teaching models to avoid highlighting the student's disability. Rather than reducing these models, it's important to adapt teaching methods to be inclusive and supportive of all students' needs.

17. Statements about Noise-Induced Hearing Loss:

(A) The acoustic reflex in the middle ear has latency and reflex adaptation, making it less protective against high-frequency noise.

- This is true. The acoustic reflex can help protect against some types of noise, but it is less effective against high-frequency sounds.

(B) Noise-induced hearing loss can be temporary in some cases, known as temporary threshold shift (TTS), where hearing recovers after rest.

- This is also true. Temporary threshold shift is a short-term reduction in hearing sensitivity following exposure to loud noise.

(C) If hearing loss is caused by long-term exposure to high-volume noise, the noise is typically unpleasant (like machinery or traffic) rather than enjoyable music (like a symphony).

- This statement is misleading. While it's true that industrial noise can cause hearing loss, prolonged exposure to loud music, even if it's perceived as enjoyable, can also lead to hearing damage.

(D) Noise-induced hearing loss typically presents as bilateral and symmetrical sensorineural hearing loss, but it rarely causes profound hearing loss in the early stages.

- This is correct. Noise-induced hearing loss usually affects both ears equally and tends to develop gradually.

The least appropriate statement is (C) If hearing loss is caused by long-term exposure to high-volume noise, it typically results from unpleasant noise rather than enjoyable music. Noise-induced hearing loss can occur from any prolonged exposure to high sound levels, regardless of the source's perceived pleasantness.

You

Please answer the following multiple-choice question as a hearing care professional, providing reasoning and explanation: 18 關於中耳膽脂瘤（cholesteatoma）的特徵，下列敘述何者最不適切？
（A）慢性中耳炎會誘發膽脂瘤的形成，常為雙側同時發生
（B）生成至一定程度後會侵蝕鄰近構造，可能會造成傳導性或是感音神經性聽力損失
（C）中耳或是乳突處若受到膽脂瘤所造成的炎性反應影響，亦可能造成顏面神經麻痺
（D）目前無特定藥物可以治療，主要是以手術介入排除為主要方式
19 關於聽覺電位反應的聽覺晚潛時反應（auditory late-latency response）之敘述，下列何者最為適切？
（A）晚潛時的反應波發生在 30～500 ms
（B）晚潛時的 P1 波形即為中潛時的 Na 反[illegible]
（C）晚潛時的反應波振幅比聽性腦幹反應[illegible]中潛時反應的振幅小很多
（D）不匹配負向波（mismatch negativity）需要患者在潛眠狀態下進行測試

ChatGPT

Let's analyze these options regarding cholesteatoma characteristics and auditory late-latency responses:

18. Statements about Cholesteatoma:

(A) Chronic middle ear infections can induce the formation of cholesteatoma, often occurring bilaterally.

- This statement is partially true. While chronic middle ear infections can lead to cholesteatoma, it usually occurs unilaterally rather than bilaterally.

(B) As it grows, it can erode nearby structures, potentially causing conductive or sensorineural hearing loss.

- This is correct. Cholesteatoma can erode s[illegible]uctures in the middle ear, leading to various types of hearing loss.

(C) Inflammation caused by cholesteatoma in the middle ear or mastoid process can also lead to facial nerve paralysis.

- This is also true. Cholesteatoma can cause complications such as facial nerve paralysis due to its location and the potential for erosion of surrounding structures.

(D) There are no specific medications for treatment, and the main approach is surgical intervention.

- This is correct. Cholesteatoma typically requires surgical treatment, as there are no effective medications for it.

The least appropriate statement is (A) Chronic middle ear infections can induce the formation of cholesteatoma, often occurring bilaterally. Cholesteatoma more commonly occurs unilaterally.

19. Statements about Auditory La[illegible] Latency Responses:

(A) Late-latency responses occur between 30–500 ms.

- This is true. Late-latency responses in auditory evoked potentials occur in this time window.

(B) The late-latency P1 wave is the same as the middle-latency Na wave.

- This is incorrect. The P1 wave in late-latency responses and the Na wave in middle-latency responses are distinct components occurring at different times.

(C) The amplitude of late-latency response waves is much smaller than that of auditory brainstem responses and middle-latency responses.

- This statement might be misleading. The amplitude of late-latency responses can vary, but directly comparing it to brainstem and middle-latency responses is not straightforward due to differences in the nature of these responses.

(D) Mismatch negativity requires the patient to be in a state of drowsiness during testing.

- This is incorrect. Mismatch negativity can be recorded in both awake and sleeping states, and it does not require the patient to be drowsy.

The least appropriate statement is (D) Mismatch negativity requires the patient to be in a state of drowsiness during testing. Mismatch negativity is a component of auditory late-latency responses that can be elicited regardless of the patient's state of consciousness.

You

Please answer the following multiple-choice question as a hearing care professional, providing reasoning and explanation: 20 關於聽神經病變（auditory neuropathy spectrum disorder, ANSD）之臨床表徵，下列何者最不適切？
（A）耳聲傳射（OAE）結果為正常，但聽性誘發腦幹反應（nABR）會呈現異常
（B）在 nABR 檢查中，使用不同極相測得之cochlear microphonic 呈現相位顛倒
（C）語音測試結果會較純音聽力檢查結果落差甚大，尤其在噪音之下表現更差
（D）通常可利用助聽器顯著改善溝通能力及學習表現
21 有關聽力障礙的敘述，下列何者最不適切？
（A）美國職業安全衛生署（OSHA）的指引中，限定噪音暴露時間量並要求每年須進行聽力檢查
（B） Temporary threshold shift（TTS）[illegible]時性的聽力減退
（C） Permanent threshold shift（PTS）通常是在高頻的範圍（3000～6000 Hz）聽力損失
（D）老年人聽力損失會影響其周邊聽覺系統，而非中樞聽覺系統

ChatGPT

Let's analyze these options regarding Auditory Neuropathy Spectrum Disorder (ANSD) and hearing impairment:

20. Clinical Characteristics of Auditory Neuropathy Spectrum Disorder (ANSD):

(A) Normal Otoacoustic Emissions (OAE) results, but abnormal Auditory Brainstem Response (ABR).

- This is a typical finding in ANSD, where outer hair cell function (measured by OAEs) is normal, but there is a disruption in the transmission of sound along the auditory pathway (reflected in abnormal ABR).

(B) In ABR testing, cochlear microphonics s[illegible]w phase reversal when using different polarities.

- This can occur in ANSD, as cochlear microphonics represent the function of outer hair cells and can be preserved even when neural transmission is disrupted.

(C) Speech testing results show a greater discrepancy compared to pure tone audiometry, especially poor performance in noise.

- This is characteristic of ANSD. Patients often have disproportionate difficulty with speech comprehension, particularly in noisy environments.

(D) Hearing aids usually significantly improve communication ability and learning performance.

- This statement is less appropriate. The effectiveness of hearing aids in ANSD can be variable. Some individuals with ANSD may not benefit significantly from conventional hearing aids due to the nature of the disorder affecting neural transmission of sound.

The least appropriate statement is (D) Hearing aids usually significantly improve communication ability and learning performance. The effe[illegible]ness of hearing aids in ANSD is less predictable compared to other types of hearing loss.

21. Statements about Hearing Impairment:

(A) OSHA guidelines limit noise exposure and require annual hearing tests.

- This is correct. OSHA (Occupational Safety and Health Administration) sets regulations for noise exposure in the workplace and mandates hearing conservation programs.

(B) Temporary threshold shift (TTS) refers to temporary hearing reduction.

- This is true. TTS is a short-term decrease in hearing sensitivity, often recovering after a period away from loud noise.

(C) Permanent threshold shift (PTS) usually occurs in high frequencies (3000–6000 Hz).

- This is also correct. PTS often affects higher frequencies initially, a typical pattern in noise-induced hearing loss.

(D) Hearing loss in older adults affects the peripheral auditory system, not the central auditory system.

- This statement is less appropriate. Age-related hearing loss (presbycusis) primarily affects the peripheral auditory system (inner ear); however, changes in the central auditory system can also occur with aging.

The least appropriate statement is (D) Hearing loss in older adults affects the peripheral auditory system, not the central auditory system. Age-related changes can involve both the peripheral and central auditory systems.

You

Please answer the following multiple-choice question as a hearing care professional, providing reasoning and explanation: 22 下列那一種症狀是掃頻 Bekesy 聽力檢查（sweep-frequency Bekesy audiometry）時，出現連續音與脈衝音兩條曲線一開始即分離？
（A）耳蝸後異常 （B）傳導型聽力損失 （C）功能性聽力損失 （D）梅尼爾氏症
23 關於瓦登柏格氏症（Waardenburg syndrome），下列敘述何者最不適切？
（A）常伴隨虹膜異色症 （B）聽損程度從[illegible]到重度都有可能
（C）常合併心臟方面疾病 （D）常有兩眼內眥過寬

ChatGPT

Let's analyze these options regarding sweep-frequency Bekesy audiometry and Waardenburg syndrome:

22. Symptom Indicated by Separation of Continuous and Pulsed Tone Curves at the Start in Sweep-Frequency Bekesy Audiometry:

(A) Retrocochlear abnormality
(B) Conductive hearing loss
(C) Non-organic (functional) hearing loss
(D) Ménière's disease

In sweep-frequency Bekesy audiometry, a distinctive pattern where the curves for continuous and pulsed tones separate right from the start typically indicates non-organic (functional) hearing loss. This pattern suggests that the patien[illegible]earing loss is not due to an organic pathology but rather to psychological or functional factors.

Therefore, the correct answer is (C) Non-organic (functional) hearing loss.

23. Statements about Waardenburg Syndrome:

(A) Often accompanied by heterochromia iridum (different colored irises).

- This is true. Waardenburg syndrome is often associated with pigmentation anomalies, including heterochromia.

(B) Hearing loss can range from mild to severe.

- This is also correct. Waardenburg syndrome can be associated with varying degrees of hearing loss.

(C) Often accompanied by cardiac diseases.

- This is less accurate. While there can be o[illegible] associated anomalies, cardiac diseases are not commonly linked with Waardenburg syndrome.

(D) Often characterized by a wider distance between the inner corners of the eyes.

- This is true. Dystopia canthorum, or a wider distance between the eyes, is a characteristic feature of Waardenburg syndrome.

The least appropriate statement is (C) Often accompanied by cardiac diseases, as this is not a typical feature of Waardenburg syndrome.

You

Please answer the following multiple-choice question as a hearing care professional, providing reasoning and explanation: 24 下列有關先天感音性聽力損失的敘述，何者最不適切？
（A）可藉藥物改善聽力 （B）不一定與基因有關
（C）巨細胞病毒源自母體在懷孕期的感染 （D）對說話、語言與認知發展有負面影響
25 Usher 氏症候群的感覺神經性聽損，初期聽力為正常或輕度，之後會逐漸變差，屬於漸進性聽損，此症狀最可能屬於下列何者？
（A）第一型 （B）第二型 （C）第三型 （[illegible] 第四型

ChatGPT

Let's analyze these options regarding congenital sensorineural hearing loss and Usher syndrome:

24. Statements about Congenital Sensorineural Hearing Loss:

(A) Hearing can be improved with medication.

- This statement is generally inappropriate. Congenital sensorineural hearing loss is typically irreversible, and there are no medications that can effectively restore hearing in these cases.

(B) Not necessarily related to genetics.

- This is true. While genetics can play a significant role in congenital sensorineural hearing loss, there are also other causes such as infections, prematurity, and environmental factors.

(C) Cytomegalovirus (CMV) results from maternal infection during pregnancy.

- This is also true. CMV is a common viral ca[illegible]e of congenital sensorineural hearing loss, transmitted from mother to fetus during pregnancy.

(D) Negatively affects speech, language, and cognitive development.

- This is correct. Congenital sensorineural hearing loss can significantly impact the development of speech, language, and cognitive abilities if not adequately managed.

The least appropriate statement is (A) Hearing can be improved with medication, as congenital sensorineural hearing loss generally cannot be treated with medication.

25. Usher Syndrome and Progressive Hearing Loss:

Usher syndrome is characterized by hearing loss and visual problems. The type of Usher syndrome determines the onset and progression of hearing loss:

(A) Type I

- Individuals with Type I typically have profound hearing loss from birth and balance issues.

(B) Type II

- Individuals with Type II usually have moderate to severe hearing loss from birth, which is not progressive or only mildly progressive.

(C) Type III

- Individuals with Type III have normal or nearly normal hearing at birth, with progressive hearing loss over time.

(D) Type IV

- There is no Type IV Usher syndrome. The classification includes only Types I, II, and III.

Given the description of initial normal or mild hearing loss with subsequent progressive worsening, the most likely type of Usher syndrome in this scenario is (C) Type III.

You

Please answer the following multiple-choice question as a hearing care professional, providing reasoning and explanation: 26 關於年長者聽力損失特質的描述，下列何者最不適切？
（A）除了聽力損失外，亦經常伴隨中樞聽覺處理方面的問題
（B）常發生雙側對稱型高頻率聽覺敏感度退化
（C）對於聽覺障礙程度的主觀感受常隨年齡的增加而提高
（D）隨著聽損程度越嚴重則認知功能測驗的分數越低
27 有關傳導性聽力障礙的敘述，下列何者最不適切？
（A）多數不是永久性質
（B）多數需要醫藥治療
（C）耳硬化影響的主要是年輕成人，尤其是男性
（D）主要影響是響度（loudness）或可聽度（audibility）

ChatGPT

Let's analyze these options regarding characteristics of hearing loss in older adults and conductive hearing loss:

26. Characteristics of Hearing Los[illegible] in Older Adults:

(A) Besides hearing loss, there are often problems with central auditory processing.

- This is correct. Age-related hearing loss (presbycusis) can be accompanied by difficulties in central auditory processing, affecting the understanding of speech, especially in noisy environments.

(B) Commonly bilateral symmetric high-frequency hearing sensitivity decline.

- This is also true. Presbycusis typically involves a bilateral and symmetrical loss of hearing sensitivity, particularly for high frequencies.

(C) Subjective perception of hearing impairment often increases with age.

- This statement might be misleading. While awareness of hearing loss can increase, some older adults may underreport or not fully recognize their hearing difficulties.

(D) As hearing loss severity increases, cognitive function test scores tend to be lower.

- There is some evidence suggesting a cor[illegible]ion between hearing loss and decreased cognitive function in older adults, but this relationship is complex and not fully understood.

The least appropriate statement is (C) Subjective perception of hearing impairment often increases with age. Older adults may not always accurately perceive or acknowledge the extent of their hearing loss.

27. Statements about Conductive Hearing Loss:

(A) Most cases are not permanent.

- This is true. Many forms of conductive hearing loss can be treated or resolve spontaneously.

(B) Most cases require medical or surgical treatment.

- While medical or surgical treatment can be necessary, not all cases of conductive hearing loss require these interventions. Some may resolve with conservative management or are temporary.

(C) Otosclerosis primarily affects young adults, especially males.

- This statement is incorrect. Otosclerosis more commonly affects young to middle-aged adults and is more prevalent in females.

(D) The primary impact is on loudness or audibility.

- This is correct. Conductive hearing loss primarily affects the transmission of sound to the inner ear, leading to a reduction in the loudness of sounds.

The least appropriate statement is (C) Otosclerosis primarily affects young adults, especially males. Otosclerosis more frequently affects females and is not specifically skewed towards males.

You

Please answer the following multiple-choice question as a hearing care professional, providing reasoning and explanation: 28 關於聽性腦幹反應（ABR）的描述，下列何者最不適切？
（A） ABR 對於聽覺異常路徑評估主要是針對聽覺毛細胞後的神經路徑為主
（B） ABR 檢查所使用的刺激音分為滴答聲（click）與純音爆破音（tone burst），對於極重度以上聽損無法估計閾值
（C）臨床上一般使用 85～90 dB nHL 高[illegible]滴答聲 ABR 來做神經學診斷設定
（D） ABR 檢查所使用的滴答聲（click）刺激音頻寬較廣，可針對不同頻率的神經刺激同步放電反應進行聽覺評估
29 有關聽力與語言篩檢的敘述，下列何者最為適切？
（A）篩檢結果具備診斷評估的效力
（B）須經由他人進行篩檢及轉介，不可自我篩檢
（C）篩檢結果可做為那些個案應接受進一步評估的建議
（D）語言治療師不可以執行聽力篩檢

ChatGPT

Let's analyze these options regarding Auditory Brainstem Response (ABR) testing and hearing and language screening:

28. Statements about Auditory Brainstem Response (ABR):

(A) ABR primarily evaluates the neural pathways beyond the hair cells in the cochlea.

- This is correct. ABR assesses the auditory nerve and brainstem pathways, not the cochlear hair cells directly.

(B) ABR uses click and tone burst stimuli, but cannot estimate thresholds for profound hearing loss.

- This is partially true. While ABR is less effective in estimating thresholds in cases of profound hearing loss, it can still provide valuable information about auditory nerve and brainstem function in these cases.

(C) Clinically, a high-intensity click ABR of 85–90 dB nHL is used for neurological diagnosis.

- This is accurate. High-intensity click stimuli are commonly used in ABR testing for neurological assessments.

(D) ABR uses broad frequency range click stimuli, allowing for the assessment of auditory responses to different frequency nerve stimulations.

- This statement is misleading. While click stimuli do cover a broad range of frequencies, they are not used to assess frequency-specific responses. Tone bursts are used for frequency-specific assessments.

The least appropriate statement is (D) ABR uses broad frequency range click stimuli, allowing for the assessment of auditory responses to different frequency nerve stimulations. Click stimuli are broad-frequency, but they don't allow for frequency-specific auditory nerve stimulation assessments.

29. Statements about Hearing and Language Screening:

(A) Screening results have diagnostic evaluation validity.

- This is incorrect. Screening identifies individuals who may benefit from further evaluation, but it does not provide a diagnosis.

(B) Screening and referral must be conducted by others, self-screening is not possible.

- This is not entirely true. While professional screening is ideal, some forms of self-screening or preliminary checks can be done, especially with today's technology.

(C) Screening results can be used to recom[illegible]nd which individuals should receive further evaluation.

- This is the most accurate statement. The purpose of screening is to identify those who may need a more comprehensive evaluation.

(D) Speech therapists cannot perform hearing screenings.

- This is not accurate. Speech therapists often perform hearing screenings, particularly in settings like schools, as part of their assessment process.

The least appropriate statement is (A) Screening results have diagnostic evaluation validity. Screening is a preliminary step and does not provide a definitive diagnosis.

You

Please answer the following multiple-choice question as a hearing care professional, providing reasoning and explanation: 30 有關兒童語言（language）篩檢的項目，下列何者最不適切？
（A）理解的詞彙量是否過少 （B）表達[illegible]是否合乎語法
（C）是否能依功能將物品分類 （D）是否[illegible]與他人對話並維持話題

31 動態評量為溝通障礙兒童評估方法之一，其評估內容與實施順序，下列何者最為適切？
（A）教學→測試→再測試 （B）測試→教學→測試 （C）教學→測試→教學 （D）測試→教學→再教學

ChatGPT

Let's analyze these options regarding language screening in children and the methodology of dynamic assessment:

30. Items in Language Screening for Children:

(A) Whether the child's vocabulary comprehension is too limited.

- Assessing vocabulary size is an important part of language screening in children, as limited vocabulary can indicate language delays.

(B) Whether the child's expressive content follows grammatical rules.

- Checking for grammatical accuracy in a child's expression is another key aspect of language screening.

(C) Whether the child can categorize objects functionally.

- The ability to categorize objects shows cognitive and language processing skills, relevant in language development.

(D) Whether the child can converse with others and maintain a topic.

- Conversational skills and the ability to stay on topic are crucial for social language use and are appropriate for screening.

The least appropriate item for language screening is (C) Whether the child can categorize objects functionally. While important for cognitive development, it's less directly related to language ability compared to the other options.

31. Dynamic Assessment Method[illegible]gy for Children with Communication Disorders:

(A) Teaching → Testing → Retesting
(B) Testing → Teaching → Testing
(C) Teaching → Testing → Teaching
(D) Testing → Teaching → Reteaching

Dynamic assessment typically involves an interactive process where the assessor evaluates how a child learns and responds to instruction, rather than merely what the child knows or can do at a single point in time. This process often includes a pretest, an intervention or teaching phase, and a post-test.

The most appropriate sequence for dynamic assessment is (B) Testing → Teaching → Testing. This sequence allows the assessor to evaluate the child's initial performance, provide intervention or instruction, and then reassess to see how the child has responded to the teaching.

You

Please answer the following multiple-choic[illegible]question as a hearing care professional, providing reasoning and explanation: 32 下列何者較不屬於嬰幼兒展現其溝通意圖的方式？
（A）手勢動作 （B）與溝通對象有眼神接觸
（C）使用聲音加上語調 （D）獨自活動時的自言自語
33 關於語音習得，下列敘述何者最為適切？
（A）聲母較韻母早習得 （B）出現在詞彙的第一個位置的語音，較容易習得
（C）語音的使用頻率與習得的順序沒有關係 （D）兒童語音習得的進程差不多，個體差異不大

ChatGPT

Let's analyze these options regarding communication intention in infants and toddlers and the acquisition of speech sounds:

32. Less Likely Indicators of Communication Intent in Infants and Toddlers:

(A) Gestures
(B) Eye contact with the communication partner
(C) Using sounds with intonation
(D) Talking to oneself during solitary play

Gestures, eye contact, and using sounds with intonation are all direct and deliberate ways that infants and toddlers show their intention to communicate. They are active attempts to engage others. In contrast, talking to oneself during solitary play is more about self-expression and exploration rather than a directed communication intent towards others.

Therefore, the less likely indicator of communication intent in infants and toddlers is (D) Talking to oneself during solitary play.

33. Appropriate Statement about Speech Sound Acquisition:

(A) Consonants are acquired earlier than vowels.

- This is generally not accurate. Vowels are typically acquired before many consonants.

(B) Sounds that appear in the first position of words are easier to acquire.

- This is often true. Children tend to acquire and produce sounds that occur at the beginning of words more easily.

(C) The frequency of use of sounds does not relate to the order of acquisition.

- This is incorrect. There is a relationship between the frequency of a sound in spoken language and the ease and order of its acquisition.

(D) The process of speech sound acquisition is similar among children, with little individual variation.

- This is not entirely accurate. While there are general patterns in speech sound acquisition, there is considerable individual variation among children.

The most appropriate statement is (B) Sounds that appear in the first position of words are easier to acquire. This aligns with observations in speech development where children often master sounds at the beginning of words before those in medial or final positions.

You

Please answer the following multiple-choi[illegible]uestion as a hearing care professional, providing reasoning and explanation: 34 下列那些主障礙可能導致病患出現吞嚥障礙的問題？①特定型語言障礙 ②失智症 ③憂鬱症
④脊柱裂 ⑤中風 ⑥咽喉癌 ⑦皮爾羅賓氏症候群（Pierre Robin syndrome）
（A）僅②③④⑤⑥⑦ （B）僅①②⑤ （C）僅③⑤⑥⑦ （D）僅①②③④⑥
35 關於常用的 44100Hz 聲波數位化或取樣頻率（sound wave digitizing or sampling frequency）與下列何者沒有直接關係？
（A）人耳聽力範圍 （B）語音頻率範圍
（C）傅立葉轉換（Fourier transform） （D）奈奎斯特取樣理論（Nyquist sampling theorem）

ChatGPT

Let's analyze these options regarding the primary disorders that can lead to dysphagia (swallowing disorders) and the relevance of a 44,100 Hz sampling frequency in digital audio:

34. Primary Disorders that May Lead to Dysphagia:

1. Specific Language Impairment (SLI)
2. Dementia
3. Depression
4. Spina Bifida
5. Stroke
6. Throat Cancer
7. Pierre Robin Syndrome

Among these options, specific language impairment (SLI) and depression are less likely to directly cause dysphagia. SLI primarily affects language processing, and while depression can have physical manifestations, it's not typically associated with swallowing disorders. In contrast, dementia, stroke, throat cancer, and Pierre Robin syndrome can directly affect the neuromuscular control required for swallowing. Spina bifida, depending on its severity and related complications, may also affect swallowing.

Therefore, the most appropriate option is (A) Only 2, 3, 4, 5, 6, 7. However, it should be noted that depression (3) might indirectly impact swallowing due to general health deterioration, but it's not a direct cause like the others.

35. The 44,100 Hz Sampling Frequency and Its Irrelevance To:

(A) Human hearing range

- The 44,100 Hz sampling rate is directly related to the human hearing range. It's chosen based on the Nyquist theorem to adequately cover the audible spectrum, typically up to 20 kHz.

(B) Speech frequency range

- The speech frequency range is within the human hearing range, and the 44,100 Hz sampling rate is more than sufficient to capture speech frequencies.

(C) Fourier transform

- Fourier transform is a mathematical process used in signal processing, including when dealing with sampled audio data. The 44,100 Hz sampling rate can be relevant to the application of Fourier transforms in audio processing.

(D) Nyquist sampling theorem

- The 44,100 Hz rate is directly related to the Nyquist theorem, which states that to accurately sample a signal, the sampling rate should be at least twice the highest frequency in the signal.

The option that has the least direct relation to the 44,100 Hz sampling frequency is (B) Speech frequency range. While speech is within the audible range that this sampling rate can capture, the choice of 44,100 Hz is more directly related to the broader range of human hearing and the technical aspects of signal processing, rather than specifically to the frequency range of speech.

You

Please answer the following multiple-choice question as a hearing care professional, providing reasoning and explanation: 36 語言測驗時，施測者對兒童說「把會發亮的東西指出來。」其主要評量的能力，下列何者最為適切?
（A）語用功能 （B）象徵語言理解 （C）接受性詞彙 （D）音韻覺識
37 關於嬰兒導向式言語（Infant-directed speech）的特徵，下列何者最不適切?
（A）較短的語句（utterance）長度 （B）較少使用詢問式語句（questioning）
（C）頻繁的慣例性口語（verbal rituals） （D）增加副語言（paralinguistic）的變化

ChatGPT

Let's analyze these options regarding the abilities assessed in a language test and the characteristics of infant-directed speech:

36. Abilities Assessed in the Language Test with the Instruction "Point out the thing that lights up":

(A) Pragmatic function

- Pragmatic function refers to the use of language in social contexts. The instruction given does not specifically assess this.

(B) Symbolic language comprehension

- Symbolic language comprehension involves understanding abstract concepts and symbols. The instruction to identify an object with a specific property (lighting up) could be seen as assessing this to a degree, but it's more straightforward and concrete.

(C) Receptive vocabulary

- Receptive vocabulary refers to understanding the meaning of words. The instruction is assessing the child's understanding of specific words and concepts ("lighting up").

(D) Phonological awareness

- Phonological awareness is the recognition of sounds and patterns in spoken language. This instruction is not assessing phonological skills.

The most appropriate ability being assessed is (C) Receptive vocabulary, as the task involves understanding the meaning of specific words in the instruction.

37. Characteristics of Infant-Directed Speech:

(A) Shorter utterance length

- This is a common feature of infant-directed speech, which tends to be concise and simple for easier comprehension by the infant.

(B) Less use of questioning sentences

- This is incorrect. Infant-directed speech often includes a higher frequency of questions to engage the infant's attention and response.

(C) Frequent use of verbal rituals

- Common in infant-directed speech, these repetitive and patterned phrases help infants learn language structure and predictability.

(D) Increased paralinguistic variation

- This is correct. Infant-directed speech often involves exaggerated intonation, pitch, and rhythm to engage the infant's attention and convey emotion.

The least appropriate characteristic of infant-directed speech is (B) Less use of questioning sentences. In contrast, questioning is often used to stimulate interaction and language development.

‹ 2 / 2 ›

You

Please answer the following multiple-choice question as a hearing care professional, providing reasoning and explanation: 38 關於嬰幼兒早期語言發展的敘述，下列何者最為適切?
（A）牙牙學語階段（babbling）約始於嬰兒 2～3 個月時
（B）聽力損失的嬰兒不會經歷牙牙學語階段
（C）幼兒的前 50 個字詞鮮少出現鼻音
（D）嬰兒約於 8～12 個月開始使用手勢動作溝通
39 小綠因為說話問題而前往醫院就診，醫師表示小綠主要問題不是因為肌力不足或肌肉不協調所造成， 而是在於說話機制的規劃困難，則小綠最可能的診斷為下列何者?
（A）吶吃（dysarthria） （B）唇顎裂（cleft palate）
（C）腦性麻痺（cerebral palsy） （D）言語失用症（apraxia of speech）

ChatGPT

Let's analyze these options regarding early language development in infants and toddlers and the likely diagnosis for the speech problem described:

38. Statements about Early Language Development in Infants and Toddlers:

(A) The babbling phase starts at about 2–3 months of age.

- This is a bit early. Babbling typically begins around 6 months of age. Before that, infants engage in cooing and other vocal play.

(B) Infants with hearing loss do not experience a babbling phase.

- This is not accurate. Infants with hearing loss may still babble, but their babbling might be delayed or different in quality compared to hearing infants.

(C) The first 50 words of toddlers rarely contain nasals.

- This statement is not entirely accurate. Nasal sounds like /m/ and /n/ are often among the first sounds children produce and can be found in early vocabulary.

(D) Infants start using gesture communication around 8–12 months.

- This is correct. Infants typically begin to use gestures such as pointing or waving to communicate before they develop spoken language.

The most appropriate statement is (D) Infants start using gesture communication around 8–12 months.

39. Likely Diagnosis for the Speech Problem Described:

(A) Dysarthria

- Dysarthria is characterized by muscle weakness or incoordination, which does not match the description given.

(B) Cleft Palate

- Cleft palate primarily causes physical abnormalities affecting speech production, different from what is described.

(C) Cerebral Palsy

- While cerebral palsy can affect muscle coordination, it's not exclusively about planning difficulties in speech mechanisms.

(D) Apraxia of Speech

- Apraxia of speech is a motor speech disorder where the issue lies in the planning and sequencing of speech movements, not muscle strength or coordination. This matches the description provided.

Given the information that the primary issue is with the planning of speech mechanisms rather than muscle strength or coordination, the most likely diagnosis for Xiao Lu is (D) Apraxia of Speech.

You

Please answer the following multiple-choice question as a hearing care professional, providing reasoning and explanation: 40 下列何者是 1880 年米蘭第二屆國際聾人教育會議（International Congress on the Education of the Deaf） 的重要決議?
（A）文法手語的研發 （B）對聽損孩童的口語訓練優於手語
（C）啟聰學校以手語作為主要教學語言 （D）為達有效溝通，任何溝通形式皆可
41 有關自然手語（natural sign language）的敘述，下列何者最為適切? ①為聾校教學所發展的語言
②無語法規則 ③和口說語言的發展進程相似 ④臺灣手語的基本手形逾 50 個以上 ⑤是一種視覺 - 肢體管道的語言
（A）僅③④⑤ （B）僅①②⑤ （C）僅①③④⑤ （D）僅①②③④

ChatGPT

Let's analyze these options regarding the resolutions of the 1880 Milan International Congress on the Education of the Deaf and the characteristics of natural sign language:

40. Important Resolutions of the 1880 Milan International Congress on the Education of the Deaf:

(A) Development of grammatical sign language
(B) Oral training for children with hearing loss is superior to sign language
(C) Use of sign language as the primary language of instruction in schools for the deaf
(D) Any form of communication is acceptable for effective communication

The 1880 Milan Congress was a pivotal event in deaf education history, known for its promotion of oralism - the teaching of speech and lip-reading over sign language. The congress passed resolutions advocating for the use of oral methods in educating deaf children, deeming oral education superior to manual (sign language) methods.

Therefore, the most appropriate answer is (B) Oral training for children with hearing loss is superior to sign language. This resolution led to a significant decline in the use of sign language in deaf education for many years.

41. Characteristics of Natural Sign Language:

1. Developed for teaching in schools for the deaf
2. Lacks grammatical rules
3. Similar developmental trajectory to spoken languages
4. Taiwanese Sign Language has more than 50 basic handshapes
5. A visual-gestural language

Natural sign languages are fully developed languages with their own grammar and syntax, not merely a creation for educational purposes. They evolve naturally within deaf communities and have a developmental trajectory similar to spoken languages. Sign languages like Taiwanese Sign Language have a rich vocabulary, including numerous handshapes, and are visual-gestural in nature.

The most appropriate answer is (A) Only 3, 4, 5. Natural sign languages are not created for educational purposes, and they certainly have their own grammatical rules. They share similarities with spoken language development, have a rich set of handshapes and signs, and are visual-gestural languages.

You

Please answer the following multiple-choice question as a hearing care professional, providing reasoning and explanation: 42 依據文化及族群認同理論而發展出的聾人認同階段中，關於「文化上的聽人階段」之敘述，下列何者最為適切?
（A）此階段為聾人認同階段的第二階段
（B）此階段的聾人以口語作為主要的溝通模式
（C）此階段的聾人可以同時融入聽人文化和聾人文化
（D）此階段的聾人看待自己是沒有醫學問題的人
43 有關雙語雙文化（bilingual-bicultural model）之敘述，下列何者最不適切?
（A）雙語的第一語言是指聾人的手語 （B）雙語是指口語和書面語
（C）以臺灣手語教育聽損學生 （D）確保學生接觸聽人與聾人文化

ChatGPT

Let's analyze these options regarding the stages of Deaf identity development and the bilingual-bicultural model in Deaf education:

42. Descriptions of the "Culturally Hearing" Stage in Deaf Identity Development:

(A) This stage is the second stage of Deaf identity development.

- The order of the stages can vary among individuals, and the "culturally hearing" stage is not specifically defined as the second stage. The stages reflect a personal journey rather than a fixed sequence.

(B) Deaf individuals in this stage primarily use spoken language as their mode of communication.

- This is true. In the "culturally hearing" stage, Deaf individuals often use spoken language and may not be fully involved in the Deaf community or use sign language extensively.

(C) Deaf individuals in this stage can simultaneously integrate into both hearing and Deaf cultures.

- This description is more aligned with a later stage of identity development where individuals may comfortably navigate both Deaf and hearing cultures.

(D) Deaf individuals in this stage view themselves as people without a medical problem.

- This perspective can be part of the "culturally hearing" stage, but it's more about embracing Deaf identity without viewing it as a deficiency or disability.

The most appropriate description of the "culturally hearing" stage is (B) Deaf individuals in this stage primarily use spoken language as their mode of communication.

43. Statements about the Bilingual-Bicultural Model:

(A) The first language in bilingualism is sign language for the Deaf.

- This is true in the context of Deaf education, where sign language is often promoted as the first language.

(B) Bilingualism refers to spoken and written language.

- This is less accurate. In the context of Deaf education, bilingualism usually refers to proficiency in sign language and the written/spoken language of the surrounding hearing community.

(C) Uses Taiwanese Sign Language to educate students with hearing loss.

- This is a correct application of the model, where a local sign language (e.g., Taiwanese Sign Language) is used as one of the languages in education.

(D) Ensures students are exposed to both hearing and Deaf cultures.

- This is a key aspect of the bilingual-bicultural model, fostering an understanding and appreciation of both Deaf and hearing cultures.

The least appropriate statement is (B) Bilingualism refers to spoken and written language. In the context of Deaf education and the bilingual-bicultural model, bilingualism typically means proficiency in both sign language and the spoken/written language of the majority culture.

You

Please answer the following multiple-choice question as a hearing care professional, providing reasoning and explanation: 44 研究發現手語能力與中文閱讀能力有高度的正相關，其可能的原因以下列何者最為適切?
（A）以自然手語為第一語言，有助於中文閱讀
（B）以中文手勢（俗稱文法手語）為第一語言，有助於中文閱讀
（C）以國語口手語為第一語言，有助於中文閱讀
（D）以嬰幼兒手勢為第一語言，有助於中文閱讀
45 甲聽力師與乙患者有結怨，甲受法院委託為乙鑑定聽力，甲想陷害乙而呈報不實的鑑定結果，導致法院無法獲得正確的資訊。甲此一行為可能會面臨的罰則，下列何者錯誤?
（A）處新臺幣 2 萬元以上 10 萬元以下罰鍰
（B）處 1 個月以上 1 年以下停業處分或廢止其執業執照
（C） 7 年以上有期徒刑
（D）若涉及刑事責任須移送該管檢察機關依法辦理

ChatGPT

Let's analyze these options regarding the relationship between sign language ability and Chinese reading skills, and the potential legal consequences for a hearing professional submitting false assessment reports:

44. Possible Reasons for a High Correlation Between Sign Language Proficiency and Chinese Reading Skills:

(A) Using natural sign language as the first language aids in Chinese reading.

- This is plausible. Natural sign language, which has its own grammatical structure and conceptual framework, can provide a strong foundation for language development, including reading skills in a second language like Chinese.

(B) Using Chinese sign language (commonly referred to as Signed Exact Chinese or grammar sign language) as the first language aids in Chinese reading.

- This can also be true. Chinese sign language that aligns closely with Chinese grammar might directly support the understanding of Chinese reading due to its closer alignment with the syntax and structure of written Chinese.

(C) Using Mandarin Mouth Hand Sign as the first language aids in Chinese reading.

- Mandarin Mouth Hand Sign combines elements of spoken Mandarin with signs. While it could aid in Chinese reading, the correlation might not be as strong as with a natural sign language.

(D) Using infant sign language as the first language aids in Chinese reading.

- Infant sign language is more about basic communication and less about developing complex language skills that would directly aid in reading.

The most appropriate reason is likely (A) Using natural sign language as the first language aids in Chinese reading, as a strong foundation in a first language (like a natural sign language) supports overall language development, including reading skills in other languages.

45. Potential Penalties for a Hearing Professional Submitting False Assessment Reports:

(A) A fine of NT$20,000 to NT$100,000.
(B) Suspension of business for 1 month to 1 year or revocation of the professional license.
(C) Imprisonment for over 7 years.
(D) Referral to the prosecutorial authority for criminal liability.

Providing false reports in a professional capacity, especially in legal contexts, is a serious ethical and legal violation. While the exact penalties depend on the jurisdiction and specific laws, typical consequences might include fines, professional censure, and potentially criminal charges if the deception has significant legal ramifications.

The option (C) Imprisonment for over 7 years seems excessively severe for the described scenario unless the false reporting led to particularly serious consequences that warranted such a punishment. However, without specific legal references, it's challenging to categorically label it as incorrect. The other options (A, B, D) are more typical forms of professional and legal repercussions for such misconduct.

You

Please answer the following multiple-choice question as a hearing care professional, providing reasoning and explanation: 46 依據聽力師法第 31 條，助聽器驗配的從業人員可以執行聽覺輔助器評估調整所必要之聽力測試，但於幾歲以下之患者應依醫囑而為之?
（A）三歲 （B）六歲 （C）十二歲 （D）十八歲
47 吳聽力師因移民國外，將其聽力師證照借予弟弟繼續經營聽力所業務，經查屬實可採下列何種處罰?
（A） 10 萬元罰鍰並限期停業 （B）廢止聽力師證書
（C） 3 萬元罰鍰並限期改善 （D） 2 萬元以上 10 萬元以下罰鍰

ChatGPT

To provide accurate answers, we need to reference the specific legal stipulations in the "Hearing Aid Dispensing Personnel Act" and related regulations for the scenarios described. However, since I cannot access external databases or legal documents, I will provide a general understanding based on common practices in audiology and professional ethics.

46. Age Limit for Hearing Tests Conducted by Hearing Aid Dispensing Personnel Without a Physician's Order:

The specific age limit for hearing tests to be conducted by hearing aid dispensing personnel without a physician's order can vary based on legal and professional guidelines in different regions. Typically, younger children, especially those below school age, are considered to require more specialized pediatric audiological assessment, often under a physician's guidance or in collaboration with a pediatric audiologist. This is due to the complexities involved in accurately assessing hearing in young children, who may not be able to reliably participate in standard hearing tests.

Based on common practices, it would be more appropriate for hearing tests on very young children, such as those under three or six years of age, to be conducted under a physician's order. Therefore, the most likely answers could be (A) Three years old or (B) Six years old, with (A) Three years old being more cautious.

47. Penalty for a Hearing Professional Lending Their License to a Family Member:

Lending a professional license to a family member for the continuation of business is a serious violation of professional ethics and legal regulations. Such actions typically result in significant penalties, including fines and potential revocation of the professional license. The severity of the penalty would depend on the specific regulations governing the practice of audiology in the region.

Options like a substantial fine (possibly in the range of the amounts specified in options (A), (C), and (D)) and revocation of the professional license (option (B)) are plausible penalties for such misconduct. Revoking the license is a common repercussion for serious ethical breaches in many professional fields.

Given the seriousness of the violation, options (B) Revocation of the hearing professional's certificate and (D) A fine ranging from NT$20,000 to NT$100,000 are likely penalties, with (B) being more severe and thus more likely in case of such a significant breach of professional conduct.

You

Please answer the following multiple-choice question as a hearing care professional, providing reasoning and explanation: 48 陳聽力師因為一場重病後導致難以有效執行業務，遂立即停止提供專業服務，此行為符合專業倫理規範中那一項守則?
（A）聽語人員與個案 （B）聽語人員與執照 （C）聽語人員與社會 （D）聽語人員與共同工作者
49 依據聽力師法及其施行細則，有關申請設立聽力所，下列敘述何者錯誤?
（A）應檢附聽力師證書正本及影本；正本驗畢後發還
（B）應檢附聽力師法第 17 條第 2 項所定聽力師之執行業務證明文件
（C）負責之聽力師以在地方主管機關指定之機構執行業務 2 年以上者為限
（D）應檢附國民身分證正本及影本；正本驗畢後發還

ChatGPT

Given that I do not have access to the specific rules and regulations of the "Hearing Aid Dispensing Personnel Act" or similar professional guidelines, I will provide an answer based on general principles in healthcare ethics and professional conduct.

48. Ethical Principle Upheld by a Hearing Professional Ceasing Practice Due to Illness:

In the scenario described, the hearing professional ceases to provide services due to an illness that impairs their ability to perform effectively. This decision aligns with ethical principles related to ensuring the welfare and safety of clients and maintaining professional competence. In the context of professional ethics guidelines, this behavior typically corresponds to principles related to the responsibilities of healthcare providers to their clients and their profession.

(A) Responsibilities to clients (patients)
(B) Responsibilities regarding licensure
(C) Responsibilities to society
(D) Responsibilities to colleagues

The most fitting ethical principle in this scenario is (A) Responsibilities to clients (patients), as it prioritizes client safety and welfare by ensuring services are provided only when the professional is fully capable of doing so.

49. Incorrect Statement Regarding the Requirements for Establishing a Hearing Clinic:

Without the specific details of the "Hearing Aid Dispensing Personnel Act," I can only provide a general perspective based on common requirements for establishing a healthcare facility:

(A) Original and copy of the hearing professional's certificate, with the original returned after verification.
(B) Proof of professional activities as specified in Article 17, Paragraph 2 of the Hearing Aid Dispensing Personnel Act.
(C) The responsible hearing professional must have worked for at least 2 years in an institution designated by the local authority.
(D) Original and copy of the national ID card, with the original returned after verification.

Typically, regulations for establishing a healthcare facility include providing proof of qualifications (such as a professional certificate) and identity verification. However, the requirement for a specific duration of work in a designated institution (as stated in option (C)) is less common and may be incorrect, as it imposes an additional experience requirement that is not a standard practice in many regions.

Therefore, the most likely incorrect statement is (C) The responsible hearing professional must have worked for at least 2 years in an institution designated by the local authority. This seems to be a more specific and potentially unusual requirement compared to the other options.

You

Please answer the following multiple-choice question as a hearing care professional, providing reasoning and explanation: 50 小同是一位聽力師，他除了把個案的資料帶回家，並和家人抱怨個案沒有錢，還買這麼貴的助聽器。他的這種行為違反了那些專業倫理?
（A）保密權、轉介權 （B）無歧視權、同意權 （C）保密權、無歧視權 （D）無歧視權、轉介權

ChatGPT

小同，作为聽力師，其行为涉及以下几个关键的专业伦理问题：

1. 保密权 (Confidentiality)：把个案的资料带回家并与家人讨论，违反了个案的保密权。作为聽力師，他应当遵守保密原则，不得未经授权泄露个案信息。
2. 无歧视权 (Non-discrimination)：对个案的经济状况发表负面评论，涉嫌歧视。专业人员应公正对待所有个案，不应因个案的经济状况或其他个人特征而有所偏见。

综上，小同的行为违反了保密权和无歧视权的专业伦理。因此，最符合的选择是 (C) 保密权、无歧视权。

You

please give your answer in English

ChatGPT

Xiao Tong, as a hearing professional, has violated several key ethical principles in his conduct:

1. Confidentiality: Taking client information home and discussing it with family members breaches the confidentiality of the client. As a hearing professional, Xiao Tong is obligated to maintain the confidentiality of client information and should not disclose it without proper authorization.
2. Non-discrimination: Making negative comments about a client's financial situation and their decision to purchase expensive hearing aids can be considered discriminatory. Professionals should treat all clients fairly and without bias based on their financial status or any other personal characteristics.

Given these considerations, Xiao Tong's behavior violates both the principles of confidentiality and non-discrimination. Therefore, the most fitting option is (C) Confidentiality, Non-discrimination.

Message ChatGPT...

ChatGPT can make mistakes. Consider checking important information.
