# Supplementary material for: Exploring the Performance of ChatGPT-4 in the Taiwan Audiologist Qualification Examination: Preliminary Observational Study Highlighting the Potential of AI Chatbots in Hearing Care
Source: JMIR Med Educ. 2024 Apr 26;10:e55595. doi: 10.2196/55595 (PMC11067446; doi:10.2196/55595)
Supplement: Multimedia Appendix 4 [file mededu-v10-e55595-s004.pdf]

ChatGPT 4

You

Please answer the following multiple-choice question as a hearing care professional, providing reasoning and explanation: 1病人在急性中耳炎（acute otitis media）症狀消失後，發現鼓室圖呈現平坦且 ECV 在正常範圍內，最有可能為下列何者？
（A）中耳積水（otitis media with effusion）（B）耳膜穿孔（tympanic membrane perforation）
（C）耳咽管功能不良（Eustachian tube dysfunction）（D）聽小骨沾黏（ossicular adhesion）

ChatGPT

在解答這個問題時，我們需要考慮每個選項與病人的症狀和鼓室圖（tympanogram）結果之間的關聯。

1. 中耳積水（otitis media with effusion, OME）：這是急性中耳炎後常見的狀況。OME時，中耳內積聚非感染性液體，導致鼓室圖呈現平坦。然而，耳鼓通常是完整的，而不是穿孔。因此，這個選項符合病人的症狀和鼓室圖的描述。
2. 耳膜穿孔（tympanic membrane perforation）：耳膜穿孔通常導致鼓室圖呈現高壓（ECV增大），因為氣體可以自由地進出中耳腔。這與病例中提到的正常ECV範圍不符。
3. 耳咽管功能不良（Eustachian tube dysfunction）：這可能導致中耳壓力不平衡，進而影響鼓室圖的結果。然而，耳咽管功能不良通常不會導致鼓室圖完全平坦，除非伴有其他併發症，如OME。
4. 聽小骨沾黏（ossicular adhesion）：這通常是慢性中耳炎或其他慢性疾病的結果，導致聽小骨之間的沾黏或固定。這種情況可能導致鼓室圖異常，但通常不會出現完全平坦的曲線。

綜上所述，最符合病人鼓室圖呈現平坦且ECV在正常範圍內的狀況是（A）中耳積水（otitis media with effusion）。

You

I hope you give your answer in English.

ChatGPT

Certainly, let's analyze the question and each option in English.

The question describes a patient who, after the symptoms of acute otitis media (AOM) have subsided, exhibits a flat tympanogram with a normal Ear Canal Volume (ECV). We need to determine the most likely condition from the given options:

1. **Otitis Media with Effusion (OME)**: After an episode of AOM, it's common for fluid to accumulate in the middle ear without infection, known as OME. This condition typically results in a flat tympanogram because the fluid dampens the movement of the eardrum and middle ear structures. The ECV would remain normal as there's no perforation or blockage in the ear canal. This option aligns well with the described tympanogram findings.
2. **Tympanic Membrane Perforation**: A perforation in the tympanic membrane usually results in an increased ECV on a tympanogram because air can freely pass into the middle ear. The tympanogram wouldn't typically appear flat; instead, it would show a high-volume pattern. This doesn't match the described tympanogram findings.
3. **Eustachian Tube Dysfunction**: This condition can cause pressure imbalances in the middle ear, affecting the tympanogram. However, Eustachian tube dysfunction alone doesn't usually result in a completely flat tympanogram unless it's accompanied by a complication like OME.
4. **Ossicular Adhesion**: This is typically a result of chronic ear conditions, where the ossicles (small bones in the middle ear) become adhered or fixed to each other. It can lead to abnormal tympanogram findings, but a completely flat curve is not typical for ossicular adhesion alone.

Given these considerations, the most likely condition for a patient with a flat tympanogram and normal ECV following acute otitis media is **(A) Otitis Media with Effusion (OME)**.

You

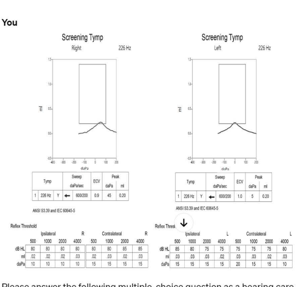

Please answer the following multiple-choice question as a hearing care professional, providing reasoning and explanation: 2　40 歲女性突然發生左側貝爾氏麻痺（Bell's palsy），其雙側聲反射及鼓室圖如下圖所示。下列敘述何者正確？
（A）病灶在第VII 對腦神經之鐙骨肌分支的遠端　（B）病灶在左側聽神經瘤
（C）該面神經麻痺可能為非器質因素所造成　（D）病灶在第V 對腦神經

ChatGPT

The question pertains to a 40-year-old female with sudden onset of Bell's palsy on the left side, with accompanying acoustic reflex and tympanogram results provided in the image. To determine the correct statement from the options, we need to interpret the tympanogram and reflex threshold results, and understand the pathophysiology of Bell's palsy.

Bell's palsy is a form of facial nerve paralysis which typically involves the facial muscles and does not directly affect the hearing apparatus. However, the facial nerve (VII cranial nerve) also carries nerve fibers that are responsible for the stapedial reflex, which involves the stapes muscle in the middle ear.

Now, let's examine the given options:

(A) The lesion is distal to the stapedius muscle branch of the VII cranial nerve. If this were the case, we would expect to see an absent acoustic reflex on the affected side because the facial nerve would be unable to activate the stapedius muscle. The tympanogram is normal, indicating that the middle ear mechanics are intact, which is not inconsistent with this option.

(B) The lesion is on the left acoustic neuroma. An acoustic neuroma (vestibular schwannoma) would more likely affect the VIII cranial nerve (auditory nerve), not the VII (facial nerve), and would typically present with hearing loss, not facial paralysis. Also, an acoustic neuroma might not affect the acoustic reflexes, unless it was large enough to impact the facial nerve indirectly.

(C) The facial nerve paralysis could be caused by non-organic factors. Non-organic factors refer to psychological or functional causes rather than physical or organic disease. Bell's palsy is typically considered an organic condition, likely caused by viral infection or inflammation of the facial nerve.

(D) The lesion is in the V cranial nerve. The V cranial nerve is the trigeminal nerve, which is primarily responsible for sensation in the face and motor functions such as biting and chewing. It is not typically associated with Bell's palsy, which affects the VII cranial nerve.

From the tympanogram, we can see that both ears present with a Type A tympanogram, which is normal, indicating normal middle ear pressure and mobility of the tympanic membrane. The acoustic reflex thresholds are elevated or absent at all frequencies in the left ear, which is consistent with a dysfunction of the stapedius muscle reflex arc, possibly due to a facial nerve disorder on the left side.

Therefore, based on the provided tympanogram and acoustic reflex results, the most likely correct answer is:

(A) The lesion is distal to the stapedius muscle branch of the VII cranial nerve.

This is because the presence of a normal tympanogram suggests that the middle ear mechanics are intact, but the absence of acoustic reflexes on the affected side suggests a lesion affecting the reflex arc, which is controlled by the facial nerve. Since the facial nerve innervates the stapedius muscle, and Bell's palsy involves the facial nerve, a lesion affecting the branch that goes to the stapedius muscle could result in the absence of acoustic reflexes as seen in the results.

You

Please answer the following multiple-choice question as a hearing care professional, providing reasoning and explanation: 3　35 歲成人最近車禍受傷後聽力變差，檢查發現中耳腔有積血，鼓室圖最可能是下列那一型？
（A） Type As （B） Type Ad （C） Type B （D） Type C
4 鼓室圖高峰時，下列有關儀器所偵測到探管音聲壓值（sound pressure level）的敘述，何者正確？
（A）聲壓值最高 （B）聲壓值最低 （C）聲壓值會不斷變動 （D）聲壓值為零

ChatGPT

To answer these questions, let's go through the basics of tympanometry and the types of tympanograms that can result from it:

1. A tympanogram is a graphic representation of the relationship between the air pressure in the ear canal and the compliance of the tympanum (eardrum) and middle ear.
2. **Type A** tympanogram shows normal pressure and compliance, indicating normal middle ear function.
   - **Type As** (shallow or stiff) indicates a less compliant (stiffer) middle ear system than normal.
   - **Type Ad** (deep or disarticulated) indicates higher than normal compliance, often seen in ossicular chain discontinuity.
3. **Type B** tympanogram shows very little or no compliance, indicating a possible fluid in the middle ear, a perforation of the tympanic membrane, or impacted earwax.
4. **Type C** tympanogram indicates negative ear pressure, suggestive of Eustachian tube dysfunction but without fluid accumulation.

For question 3, a 35-year-old adult who has decreased hearing after a car accident and has been found to have blood in the middle ear (hemotympanum) would most likely have a **Type B** tympanogram. This is because the presence of blood would prevent the eardrum from moving normally, resulting in flat tympanometry with no peak, which is characteristic of a Type B tympanogram.

For question 4, when a tympanogram shows a peak, it is the point where the compliance of the middle ear is at its highest and the air pressure in the ear canal is equal to the pressure in the middle ear. This would correspond with the **lowest** sound pressure level needed to achieve the maximum acoustic admittance because the system is most compliant and thus most easily moved by the probe tone. Therefore, the correct answer is:

(B) The sound pressure level is lowest.

You

Please answer the following multiple-choice question as a hearing care professional, providing reasoning and explanation: 5　關於ABR 產生之生理機轉，下列敘述何者最不適當？
（A）第 I 波主要是由聽神經產生
（B）第 II 波主要是由耳蝸神經核產生
（C）根據神經元的幾何走向，第V 波應該不是由下丘產生
（D）短聲能使聽神經同步放電，因此常用於ABR 檢查
6 根據美國聽力學會（2011）的指引，下列何種鼓室圖檢查結果不適合作為兒童中耳篩檢唯一的轉介標準？
（A） Type C （B） Type B
（C） Type As （D）鼓室寬度（tympanometric width）≥ 250 daPa

ChatGPT

For question 5, regarding the physiological mechanism of Auditory Brainstem Response (ABR):

(A) **Wave I** of the ABR is typically generated by the auditory nerve (cranial nerve VIII), specifically by the distal portion of the nerve near the cochlea.
(B) **Wave II** is thought to be generated by the cochlear nucleus or by the proximal portion of the auditory nerve.
(C) **Wave V** is believed to be generated by the midbrain region, specifically the lateral lemniscus and inferior colliculus. If a statement suggests that Wave V should not be generated by the lower colliculus (inferior colliculus), it is incorrect, as the inferior colliculus is indeed involved in the generation of Wave V.
(D) **Clicks or short sounds** are used in ABR testing because they can cause the auditory nerve to fire in synchrony, which is necessary for generating clear waveforms in the ABR.

The least appropriate statement is (C) "According to the geometric orientation of neurons, Wave V should not be generated by the inferior colliculus." This is incorrect because Wave V is associated with the midbrain region and the inferior colliculus's contribution to the auditory pathway is well-established in the generation of ABR Wave V.

For question 6, regarding the tympanogram results that are not suitable as the sole criterion for referral in pediatric middle ear screening according to the American Academy of Audiology (2011):

(A) **Type C** tympanogram indicates negative middle ear pressure, which can be associated with Eustachian tube dysfunction. While it may warrant follow-up, it's not always an immediate cause for referral on its own.
(B) **Type B** tympanogram indicates a flat response, which could mean fluid in the middle ear, a perforated eardrum, or presence of a foreign body. This type of result is typically considered a reason for immediate referral.
(C) **Type As** tympanogram shows normal middle ear pressure but reduced compliance, indicating a stiffer system. It can be associated with otosclerosis or tympanosclerosis and may not always require immediate referral.
(D) **Tympanometric width** of ≥ 250 daPa suggests a flaccid eardrum or a highly compliant middle ear system. It is associated with pathologies such as ossicular disarticulation or a monomeric tympanic membrane.

The tympanogram result that is not suitable as the sole criterion for referral would be (A) **Type C** because it can occur with transient Eustachian tube dysfunction, which can be common in children, especially with colds or allergies, and might resolve without medical intervention. However, this doesn't preclude the need for follow-up if the condition persists.

You

Please answer the following multiple-choice question as a hearing care professional, providing reasoning and explanation: 7　中耳包含鐙骨肌（stapedius muscle）與鼓膜張肌（tensor tympani muscle），分別由十二對腦神經中的那兩對所控制？
（A）顏面神經、三叉神經 （B）顏面神經、滑車神經
（C）耳蝸前庭神經、三叉神經 （D）耳蝸前庭神經、滑車神經
8 當測試耳有耳蝸感音性聽力損失時，下列有關耳聲傳射對側抑制的敘述，何者正確？
（A）輕度以下的聽損，記錄到的抑制量比正常聽力耳低
（B）提高記錄的刺激音量可以有效記錄到抑制作用
（C）無法記錄到任何抑制作用
（D） 50 dB HL 的聽損仍可觀察到抑制作用

ChatGPT

For question 7, the control of the muscles within the middle ear is as follows:

- The **stapedius muscle** is controlled by the facial nerve, which is the seventh cranial nerve.
- The **tensor tympani muscle** is controlled by the mandibular branch of the trigeminal nerve, which is the fifth cranial nerve.

Therefore, the correct answer is:
(A) The facial nerve (VII cranial nerve) and the trigeminal nerve (V cranial nerve).

For question 8, concerning contralateral suppression of otoacoustic emissions (OAEs) during testing for cochlear (sensory) hearing loss:

(A) When mild hearing loss is present, the amount of suppression observed in the OAEs can be less than that of a normal hearing ear, as the emissions may already be reduced due to the cochlear damage.
(B) Increasing the level of the stimulus sound may not necessarily result in observable suppression if the cochlea is damaged; it depends on the extent and nature of the hearing loss.
(C) It's not correct to say that no suppression can be recorded; even with cochlear hearing loss, some degree of suppression may be detectable depending on the severity and frequency range of the loss.
(D) Suppression can still be observed in cases of 50 dB HL hearing loss; it again depends on the specific characteristics of the individual's hearing loss.

The most accurate statement is:
(D) Suppression effects can still be observed with a 50 dB HL hearing loss. Even though the hearing loss may affect the strength or integrity of the otoacoustic emissions, some level of contralateral suppression can often be detected unless the hearing loss is profound.

You

Please answer the following multiple-choice question as a hearing care professional, providing reasoning and explanation: 9　下列感音神經性聽力損失程度中，DPOAEs 振幅大小和行為聽檢閾值之關係，何者最為適當？
（A） 60 dB HL 以上時，DPOAEs 振幅和行為聽閾值無明顯相關
（B） 60 ~ 75dB HL 時，當行為聽閾值越低，DPOAEs 振幅通常越小
（C） 35 ~ 50 dB HL 時，當行為聽閾值越高，DPOAEs 振幅通常越大
（D） 50 dB HL 以下時，DPOAEs 振幅和行為聽閾值無明顯相關
10　下列何種測試方法不能用於檢測耳咽管功能？
（A）伐爾沙瓦氏操作法（Valsalva maneuver）
（B）充氣 - 放氣程序檢查（inflation–deflation procedure test）
（C）湯恩比操作法（Toynbee maneuver）
（D）氣室耳鏡檢查（pneumatic otoscopy）

ChatGPT

For question 9, Distortion Product Otoacoustic Emissions (DPOAEs) are sounds generated by the outer hair cells in the cochlea when they are stimulated by two pure-tone frequencies. The relationship between the amplitude of DPOAEs and the behavioral audiometric thresholds can inform us about the degree of sensorineural hearing loss:

(A) At levels above 60 dB HL, DPOAE amplitudes can be very low or absent. As the degree of hearing loss increases, the outer hair cells may be so damaged that they do not produce measurable emissions. Therefore, there may be no significant correlation between DPOAE amplitude and behavioral thresholds at this level.

(B) Between 60–75 dB HL, as the behavioral audiometric threshold increases (worsening hearing), the amplitude of DPOAEs typically decreases because the outer hair cells are progressively more affected.

(C) Between 35–50 dB HL, the relationship between DPOAE amplitude and behavioral thresholds is such that higher thresholds (worse hearing) usually result in lower DPOAE amplitudes.

(D) Below 50 dB HL, there is usually a clear correlation between DPOAE amplitudes and behavioral thresholds. Higher DPOAE amplitudes are generally associated with lower behavioral thresholds (better hearing).

The most appropriate relationship is:
(B) Between 60–75 dB HL, as the behavioral hearing threshold gets worse (higher threshold), the DPOAE amplitude typically decreases.

For question 10, the tests that are used to assess Eustachian tube function are:

(A) The Valsalva maneuver, which involves attempting to blow out with the nose and mouth closed, can indicate whether the Eustachian tube can open properly.

(B) The inflation-deflation test, where the middle ear is inflated with air and then deflated, can assess the function of the Eustachian tube in terms of pressure equalization.

(C) The Toynbee maneuver, which involves swallowing with the nostrils pinched closed, helps to evaluate the opening function of the Eustachian tube.

(D) Pneumatic otoscopy, while it can assess the mobility of the tympanic membrane, it does not directly test the function of the Eustachian tube. It is more indicative of middle ear pressure at the moment of examination rather than the functional status of the Eustachian tube.

Thus, the test that cannot be used to assess Eustachian tube function is:
(D) Pneumatic otoscopy.

You

Please answer the following multiple-choice question as a hearing care professional, providing reasoning and explanation: 11 在沒有聲源刺激下偵測到的耳聲傳射是下列何者？
（A） SOAEs （B） TEOAEs （C） DPOAEs （D） SFOAEs
12　關於聽性腦幹反應於術中監測之相關應用，下列敘述何者錯誤？
（A）聽性腦幹反應可用於聽神經瘤移除之術中監測
（B）手術室中的電子儀器雜訊，會干擾聽性腦幹反應之量測
（C）若聽性腦幹反應與耳蝸電圖併用於術中監測，可代表周邊及中樞聽覺系統的反應
（D）由於病患處於麻醉狀態，因此手術室噪音不會造成影響

ChatGPT

For question 11, Otoacoustic Emissions (OAEs) are sounds generated by the inner ear that can be measured with a sensitive microphone in the ear canal. There are different types of OAEs:

(A) **Spontaneous Otoacoustic Emissions (SOAEs)** are sounds that are emitted by the ear without an external stimulus. They are present in some but not all individuals.

(B) **Transient Evoked Otoacoustic Emissions (TEOAEs)** are sounds that are emitted in response to a very brief stimulus, typically a click or tone burst.

(C) **Distortion Product Otoacoustic Emissions (DPOAEs)** are generated when the ear is stimulated simultaneously by two pure tones, and the ear itself produces a third tone as a result of the cochlea's non-linear response.

(D) **Stimulus Frequency Otoacoustic Emissions (SFOAEs)** are measured while a continuous pure tone stimulus is presented.

The correct answer for the type of OAEs that can be detected without an external sound stimulus is:
(A) SOAEs.

For question 12, regarding the use of Auditory Brainstem Response (ABR) in intraoperative monitoring:

(A) ABR can be used to monitor the integrity of the auditory nerve during the removal of an acoustic neuroma.

(B) Electronic equipment in the operating room can indeed introduce noise that interferes with the measurement of ABR.

(C) Combining ABR with electrocochleography (ECoG) during surgery can provide information on both peripheral and central auditory system responses.

(D) Operating room noise can affect ABR measurements, but it is not because the patient is under anesthesia and unable to hear it. The noise can interfere with the equipment's ability to detect the ABR, not with the patient's ability to produce an ABR. Anesthesia can affect the morphology and threshold of ABR, but the statement seems to imply that the noise has no effect due to anesthesia, which is not correct.

The incorrect statement is:
(D) Operating room noise can indeed impact ABR measurements regardless of the patient's anesthetized state because it can introduce artifacts that complicate the interpretation of the ABR waveforms.

You

Please answer the following multiple-choice question as a hearing care professional, providing reasoning and explanation: 13 TEOAEs 反應波強度，通常以下列何種單位表示？
（A） dB （B） dB SPL （C） dB HL （D） dB nHL

14　關於現今臨床上耳蝸電圖（ECochG）的應用，下列敘述何者最不適當？
（A）當 ABR 第 I 波太小時，可嘗試用耳蝸電圖識別聽神經的反應
（B）能協助診斷聽神經譜系病變
（C）能使用 SP 來評估內淋巴水腫
（D）常規地用於嬰幼兒聽力閾值的評估

ChatGPT

For question 13, the response amplitude of Transient Evoked Otoacoustic Emissions (TEOAEs) is typically represented in:

(A) **dB**: A general unit of measurement for the intensity of sound.
(B) **dB SPL (Sound Pressure Level)**: A unit that specifies the pressure level of a sound relative to a reference value and is commonly used in the measurement of acoustic signals.
(C) **dB HL (Hearing Level)**: This unit is used to compare an individual's hearing to the average normal hearing thresholds. It is often used in pure-tone audiometry but not typically for OAEs.
(D) **dB nHL (normalized Hearing Level)**: This unit is often used for auditory evoked potentials but not typically for OAEs.

The correct unit commonly used for representing the amplitude of TEOAEs is:
(B) dB SPL

For question 14, regarding the application of Electrocochleography (ECochG) in clinical settings:

(A) ECochG can indeed be used to identify responses from the auditory nerve when Wave I of the ABR is too small or absent.
(B) It can assist in the diagnosis of auditory nerve and cochlear diseases.
(C) The summating potential (SP) can be used to evaluate endolymphatic hydrops, which is associated with Meniere's disease.
(D) While ECochG can be used in infants for diagnostic purposes, it is not typically used as a routine evaluation for hearing thresholds in infants due to the invasiveness of the test and the difficulty in obtaining reliable responses in this population.

The least appropriate statement is:
(D) ECochG is not commonly used for routine assessment of hearing thresholds in infants. Other non-invasive methods such as OAEs and ABR are typically preferred for this purpose.

You

Please answer the following multiple-choice question as a hearing care professional, providing reasoning and explanation: 15 進行爆破音聽性腦幹反應（tone burst ABR）檢查時，設定參數斜率修飾（ramping）之作用為何？
（A）降低頻譜擴散（splatter）並增加頻率特異性
（B）增加低通濾波器的敏感度，降低電訊干擾（electrical artifact）
（C）增加爆破音的頻譜範圍，可以獲得更多神經放電反應
（D）降低非刺激音誘發腦波（EEG）訊號干擾的問題
16　關於單側小耳症合併先天性耳道閉鎖（microtia with congenital aural atresia）幼兒患者之聽覺評估，下列敘述何者最不恰當？
（A）必須使用聽性腦波反應之氣導與骨導閾值差來得知傳導聽損程度
（B）為降低刺激音跨傳到優耳的影響，必須使用插入式耳機，才能測得準確的聽力閾值
（C）出生不到 1 個月的新生兒由於頭骨尚未完全閉合，因此優耳不需要使用噪音來遮蔽
（D）使用耳垂而非乳突作為電極放置的位置，較能凸顯第 I 波出來

ChatGPT

For question 15, when performing a tone burst Auditory Brainstem Response (ABR) test, the purpose of setting a ramping parameter (which controls the rise/fall time of the stimulus) is:

(A) To reduce spectral splatter and increase frequency specificity. Ramping makes the onset and offset of the tone burst more gradual, which can help to concentrate the energy of the stimulus more narrowly around its central frequency, reducing the spread of energy across the frequency spectrum.

(B) Increasing the sensitivity of a low-pass filter to reduce electrical artifact is not the purpose of ramping. Filtering is a separate parameter adjusted during ABR testing to reduce the effect of electrical noise.

(C) Increasing the spectral range of the tone burst to obtain more neural responses is not the purpose of ramping. A wider spectral range is not typically desired when specificity of frequency response is needed.

(D) Reducing interference from non-stimulus-induced EEG signals is not the direct purpose of ramping. While ramping may help with the overall quality of the ABR recording by reducing spectral splatter, it is not specifically aimed at EEG noise.

The correct function of ramping is:
(A) To reduce spectral splatter and increase frequency specificity.

For question 16, regarding auditory assessment in children with unilateral microtia and congenital aural atresia:

(A) It is necessary to use the difference between air conduction and bone conduction thresholds in Auditory Brainstem Response (ABR) testing to determine the degree of conductive hearing loss.

(B) To minimize cross-hearing to the better ear, it is indeed important to use insert earphones for accurate threshold determination.

(C) Not using noise to mask the better ear in newborns under 1 month of age because their cranial bones have not yet fully closed may not be appropriate. Even if the bones are not fully closed, cross-conduction can still occur, and masking may be necessary to obtain accurate results.

(D) Placing the electrode on the earlobe instead of the mastoid to highlight Wave I of the ABR is not a typical practice. Electrode placement for ABR is standardized and is typically on the mastoid or high on the forehead (vertex), not the earlobe, to obtain clear recordings.

The least appropriate statement is:
(C) The fact that the cranial bones are not fully closed in newborns does not mean that masking the better ear is unnecessary. Cross-conduction can still occur, and proper masking is important to prevent it and to obtain accurate threshold information.

You

Please answer the following multiple-choice question as a hearing care professional, providing reasoning and explanation: 17 關於聽性穩定狀態反應（ASSR）的臨床應用，下列敘述何者錯誤？
（A）使用較高（ > 70 Hz）的調變速率（modulation rate）進行測驗，較不受清醒程度影響
（B）使用較低（ < 50 Hz）的調變速率進行測驗，測得的反應多由周邊聽覺系統所產生
（C）使用相同載波頻率（carrier frequency），調變速率越高，潛時越短
（D）使用相同調變速率，載波頻率越高，潛時越短
18　聽覺誘發電生理檢查中，有關刺激音參數影響反應的敘述，下列何者正確？
（A）刺激音時長越長，越容易記錄到早潛時反應
（B）在一定時間內，刺激音時長越長，刺激音間隔（ISI）也會增加
（C）刺激速率改變，不影響刺激音間隔
（D）要誘發潛時越晚的反應，需要使用刺激速率較慢的刺激音

ChatGPT

For question 17, regarding the clinical application of Auditory Steady State Responses (ASSR):

(A) Tests using higher modulation rates (>70 Hz) are indeed less affected by the state of alertness. Higher modulation rates can produce a response even when a patient is not fully sedated or is in light sleep.

(B) Lower modulation rates (<50 Hz) tend to reflect the response generated more from the peripheral auditory system, as the modulation rate is closer to the frequency range of the natural physiological activity of the auditory system.

(C) The latency of the response is not typically related to the modulation rate when using the same carrier frequency. Latency in ASSR is usually defined as the phase lag of the response relative to the phase of the modulating tone, and it does not typically get shorter with higher modulation rates.

(D) Higher carrier frequencies do not necessarily result in shorter latencies with the same modulation rate. The latency of the response is more complex and depends on various factors including neural conduction time and synaptic delays.

The incorrect statement is:
(C) Using the same carrier frequency, higher modulation rates do not necessarily result in shorter latencies.

For question 18, regarding the effects of stimulus parameters on responses in electrophysiological hearing tests:

(A) Longer stimulus duration does not make it easier to record early latency responses. Early latency responses are best recorded with brief stimuli that have a fast rise/fall time.

(B) If the stimulus duration is lengthened within a given time frame, the inter-stimulus interval (ISI) must be increased to accommodate the longer duration, otherwise, the total number of stimuli per second would change.

(C) Changing the stimulus rate will affect the ISI. The ISI is the time between the end of one stimulus and the beginning of the next. If the rate increases, the ISI decreases, and vice versa.

(D) To elicit later latency responses, it is often necessary to use a slower stimulus rate. This allows the neural responses more time to develop and can prevent the overlap of responses that can occur with faster rates.

The correct statement is:
(D) To elicit later latency responses, a slower stimulus rate is typically used.

You

Please answer the following multiple-choice question as a hearing care professional, providing reasoning and explanation: 19 欲評估一名個案在未專注聆聽時，是否能區辨兩種不同刺激音，使用下列何項檢查最為合適？
（A） MLR （B） MMN （C） P300 （D） ECochG
20 有關短聲誘發聽性腦幹反應（click evoked ABR）於正常聽力者測試的敘述，下列何者正確？
（A）屬於早期反應，潛時約 15 ~ 50 ms
（B）反應波向上，非倒轉（non-inverting）電極應置於頭頂
（C）刺激強度 85 dB nHL 時，振幅約 5 μvolt
（D）潛時會比用低頻爆破音（low-frequency tone burst）長

ChatGPT

For question 19, when evaluating whether a subject can differentiate between two different sounds without actively listening, the following tests are considered:

(A) **MLR (Middle Latency Response)**: This is an electrophysiological test that assesses the auditory pathway up to the midbrain level. While it reflects auditory processing, it is not typically used to assess sound discrimination.

(B) **MMN (Mismatch Negativity)**: This test is used to assess the brain's automatic response to changes in auditory stimuli. MMN is elicited by an infrequent, deviant stimulus amid a series of frequent, standard stimuli and is used to assess auditory discrimination without the need for the subject's active attention.

(C) **P300**: This is an event-related potential elicited in the process of decision making and is usually measured by requiring the subject to actively listen and respond to the stimuli.

(D) **ECochG (Electrocochleography)**: This is a test that measures the electrical potentials generated in the cochlea and auditory nerve in response to sound. It is not used for assessing discrimination of sound without focused listening.

The most appropriate test to assess passive sound discrimination is:
(B) MMN

For question 20, regarding the characteristics of click-evoked ABR in normal-hearing individuals:

(A) Click-evoked ABRs are indeed early responses, with wave latencies typically falling between approximately 1.5 to 10 ms (not 15-50 ms). Therefore, the timing provided is incorrect.

(B) The non-inverting electrode is usually placed on the vertex (the top of the head), and the inverting electrode is placed on the mastoid or earlobe. Therefore, the statement is incorrect as it stands.

(C) The amplitude of the ABR response at a stimulus intensity of 85 dB nHL can be in the range of microvolts (μV), but specifying an amplitude of about 5 μV is not accurate for all individuals because there can be considerable variability in response amplitudes.

(D) Latencies for click-evoked ABRs are generally shorter because clicks are broadband stimuli that tend to stimulate a broad region of the cochlea simultaneously, leading to a synchronous neural response. Low-frequency tone bursts have longer latencies because they stimulate a more apical region of the cochlea which has a longer traveling wave delay.

The correct statement is:
(D) The latencies for click-evoked ABRs are shorter than those elicited by low-frequency tone bursts.

You

Please answer the following multiple-choice question as a hearing care professional, providing reasoning and explanation: 21 有關聽性腦幹反應（ABR）反應波來源的敘述，下列何者正確？
（A）第 I 波是由聽神經近端之電位而來
（B）嬰幼兒因為聽神經比較短，造成第 II 波常消失或不顯著
（C）第V 波來源是同側中腦的外側蹄系（lateral leminiscus）至下丘（inferior colliculus）之間
（D）每一個反應波皆有其獨立的神經來源
22 為新生兒進行鼓室圖檢查時，下列那一探管音頻率最合適？
（A） 112 Hz （B） 226 Hz （C） 1000 Hz （D） 2048 Hz

ChatGPT

For question 21, regarding the sources of the waves in an Auditory Brainstem Response (ABR) test:

(A) **Wave I** of the ABR is indeed generated by the near portion of the auditory nerve (the distal portion of the nerve near the cochlea).

(B) In infants, the auditory nerve is not significantly shorter than in adults. The disappearance or insignificance of Wave II in infants is not typically due to the length of the auditory nerve, but rather to the immaturity of the auditory pathway or difficulties in differentiating the waves due to overlapping latencies.

(C) **Wave V** of the ABR is believed to originate from the lateral lemniscus up to the level of the inferior colliculus in the midbrain. This statement correctly identifies the general source of Wave V.

(D) While each ABR wave is thought to have a primary generator, the neural generators of ABR waves are complex and can involve multiple structures. Therefore, stating that each wave has a single, independent neural source is an oversimplification.

The correct statement is:
(C) Wave V originates from the lateral lemniscus to the inferior colliculus in the midbrain.

For question 22, regarding the appropriate probe tone frequency for tympanometry in newborns:

(A) **112 Hz** is typically used for infants and young children. Newborns and young children have a more compliant ear canal than adults, and lower frequencies such as 226 Hz can sometimes overestimate middle ear pressure and compliance. Therefore, a lower frequency probe tone is often recommended.

(B) **226 Hz** is the standard probe tone frequency used in tympanometry for older children and adults. However, for newborns, this frequency may not always provide accurate results due to the high compliance of their ear canals.

(C) **1000 Hz** and (D) **2048 Hz** are not standard frequencies used in tympanometry for newborns or adults.

The most appropriate probe tone frequency for tympanometry in newborns is:
(A) 112 Hz.

You
Please answer the following multiple-choice question as a hearing care professional, providing reasoning and explanation: 23 下列關於聽覺晚潛時反應（LLR）的敘述，何者錯誤?
（A） LLR 之 P1 相當於MLR 之 Pb
（B）兒童的 LLR 反應以 P1 及 N2 為主
（C）與爆破音相比，使用語音誘發之 LLR 其 N1 及 P2 潛時較短
（D）在聽覺處理異常（APD）的兒童身上測量可發現 P1 及 N2 之潛時異常延長
24 以聽性穩定狀態反應（ASSR）預測聽損者之行為聽力閾值，下列那一頻率最不準確?
（A） 500 Hz （B） 1000 Hz （C） 2000 Hz （D） 4000 Hz

ChatGPT
For question 23, regarding the characteristics of Late Latency Responses (LLR) in auditory processing:

(A) The P1 component of the LLR is considered equivalent to the Pb component of the Middle Latency Response (MLR). This equivalence is based on their similar latency and general response characteristics.

(B) In children, the LLR typically is characterized by prominent P1 and N2 components. These components are significant in the study of auditory processing, especially in developmental studies.

(C) Using speech stimuli for LLR typically results in longer, not shorter, N1 and P2 latencies compared to non-speech stimuli like tone bursts. Speech sounds are complex and require more processing time, leading to longer latencies.

(D) In children with Auditory Processing Disorders (APD), abnormalities in the latencies of components like P1 and N2 can be observed, which may include prolonged latencies.

The incorrect statement is:
(C) Using speech to evoke LLR results in lo[illegible]r N1 and P2 latencies compared to using tone bursts.

For question 24, regarding the accuracy of Auditory Steady State Responses (ASSR) in predicting behavioral hearing thresholds at different frequencies:

(A) **500 Hz**: ASSR is generally accurate across a range of frequencies, but at lower frequencies like 500 Hz, there can sometimes be less agreement with behavioral thresholds due to factors such as middle ear resonance and the nature of the steady-state response at lower frequencies.

(B) **1000 Hz**: ASSR is quite reliable at this frequency and tends to correlate well with behavioral thresholds.

(C) **2000 Hz** and (D) **4000 Hz**: ASSR is also generally accurate at these higher frequencies, often showing a good correlation with behavioral hearing thresholds.

The frequency where ASSR predictions are least accurate is typically:
(A) 500 Hz. This lower frequency can present more challenges in correlating ASSR results with behavioral hearing thresholds.

You
Please answer the following multiple-choice question as a hearing care professional, providing reasoning and explanation: 25 關於各種聽性誘發反應的測量及應用，下列敘述何者正確?
（A）若 ABR 的第 III 波辨識不易，可嘗試以 ECochG 輔助
（B） ABR 與 LLR 及 P300 一樣，無論患者檢查時睡眠惺忪或緊張兮兮，只要操作得宜，也不會過度影響結果
（C）增加短聲的刺激速率至 21.1 次 / 秒，對 ABR 的結果沒有影響，但可能無法誘發出 LLR
（D） ECochG、ABR、LLR 及 P300 均可在 100 ms 的視窗內記錄到一組完整的波形
26 聽性腦幹反應（ABR）使用插入式耳機和覆耳式耳機的比較，下列敘述何者錯誤?
（A）插入式耳機較不易造成假性傳導性聽損
（B）插入式耳機具有較大的耳間衰減值
（C）覆耳式耳機播放的短聲有較少的餘響（ringing）
（D）覆耳式耳機較容易對第 I 波產生干擾

ChatGPT
For question 25, regarding the measureme[illegible] and application of various auditory evoked responses:

(A) If Wave III of the ABR is difficult to identify, using Electrocochleography (ECochG) may not necessarily assist in its identification. ECochG is more focused on the responses from the cochlea and early part of the auditory nerve, not specifically on the brainstem waveforms like Wave III of the ABR.

(B) The ABR is relatively insensitive to the state of alertness of the patient, unlike LLR and P300. The ABR can be reliably recorded whether the patient is awake, sleepy, or even under light anesthesia. However, LLR and especially P300 are affected by the patient's level of alertness and attention.

(C) Increasing the rate of click stimuli to 21.1/s can affect the ABR by reducing the amplitude and increasing the latency of the waves. This rate is too fast for LLR to be elicited effectively.

(D) ECochG, ABR, LLR, and P300 do not all produce a complete set of waveforms within a 100 ms window. While ABR typically falls within this range, LLR and P300 have longer latency components that extend beyond 100 ms.

The correct statement is:
(B) The ABR is relatively insensitive to the patient's state of alertness, unlike LLR and P300.

For question 26, comparing insert earphones and circumaural (over-the-ear) headphones in ABR testing:

(A) Insert earphones are less likely to cause a false conductive hearing loss appearance because they don't exert pressure on the ear that might affect the middle ear function.

(B) Insert earphones generally provide greater interaural attenuation (ear-to-ear crosstalk) than circumaural headphones. This reduces the likelihood of sound being heard by the non-test ear.

(C) Circumaural headphones might have more ringing (residual sound after the stimulus) compared to insert earphones, not less. This is due to their design and the larger air volume they enclose.

(D) There is no evidence suggesting that circumaural headphones are more likely to interfere with Wave I of the ABR than insert earphones.

The incorrect statement is:
(C) Circumaural headphones can have more residual sound (ringing) compared to insert earphones.

You
Please answer the following multiple-choice question as a hearing care professional, providing reasoning and explanation: 27 當低通濾波器（low pass filter）的切截頻率（cutoff frequency），從 10000 Hz 降低到 3000 Hz，對 ABR
波形會造成什麼影響?
（A）增加雜訊的影響，潛時不變、波幅變大 （B）增加雜訊的影響，潛時延長、波幅變小
（C）降低雜訊的影響，潛時不變、波幅不變 （D）降低雜訊的影響，潛時縮短、波幅變大
28 有關刺激速率對正常聽覺個案聽性腦幹反應的影響，下列敘述何者正確?
（A）刺激速率變慢潛時延長 （B）增加刺激速率導致第 III 波與第 IV 波合併
（C）增加刺激速率對第 III 波沒有影響 （D）增加刺激速率 I 波和V 波仍可區辨

ChatGPT
For question 27, regarding the impact of reducing the cutoff frequency of a low-pass filter from 10,000 Hz to 3,000 Hz on the Auditory Brainstem Response (ABR) waveform:

- Lowering the cutoff frequency of the low-pass filter in ABR testing means that high-frequency components of the signal above 3,000 Hz will be filtered out. This would generally reduce the amount of high-frequency noise in the recording.

(A) **Increased noise effect, unchanged latency, increased amplitude**: Reducing the cutoff frequency will reduce, not increase, high-frequency noise. It shouldn't significantly affect the latency or necessarily increase the amplitude of the ABR waves.

(B) **Increased noise effect, prolonged latency, decreased amplitude**: As stated before, reducing the cutoff frequency will decrease high-frequency noise. It won't necessarily prolong latency or decrease amplitude.

(C) **Reduced noise effect, unchanged latency, unchanged amplitude**: Lowering the cutoff frequency will indeed reduce high-frequency noise. Generally, this should not significantly change the latency or amplitude of the ABR waves.

(D) **Reduced noise effect, shortened latency, increased amplitude**: While the reduction of the cutoff frequency will reduce noise, it won't necessarily shorten the latency or increase the amplitude of the ABR waves.

The correct answer is:
(C) Reducing the cutoff frequency to 3,000 Hz will decrease the high-frequency noise without significantly changing the latency or amplitude of the ABR waves.

For question 28, concerning the effects of stimulus rate on the ABR in individuals with normal hearing:

(A) **Slower stimulus rate, prolonged latency**: A slower stimulus rate doesn't necessarily prolong the latency of the ABR waves. It can allow for clearer separation of the waves but doesn't change their inherent latencies.

(B) **Increased stimulus rate, merging of Wave III and Wave IV**: Increasing the stimulus rate can lead to a blending of the later waves (III, IV, [illegible] V), as the nervous system doesn't have as much time to recover between stimuli.

(C) **Increased stimulus rate, no effect on Wave III**: Increasing the stimulus rate can affect all waves, including Wave III, typically causing them to decrease in amplitude and possibly merge with other waves.

(D) **Increased stimulus rate, Wave I and Wave V still distinguishable**: Even with an increased stimulus rate, Waves I and V are generally still distinguishable, though the amplitude may be reduced, and the inter-wave intervals may be affected.

The correct answer is:
(B) Increasing the stimulus rate can lead to the merging of Wave III and Wave IV in the ABR.

You
Please answer the following multiple-choice question as a hearing care professional, providing reasoning and explanation: 29 施行聽性腦幹反應（ABR）時發現第 I 波不明顯或無法判讀時，下列何者不是有效的解決方法?
（A）加大音強 （B）加快刺激速率
（C）使用疏極（rarefaction）刺激音 （D）使用耳道電極
30 以雙耳間第V 波潛時差異（interaural latency difference, ILD）作為聽性腦幹反應的診斷依據，下列敘述何者正確?
（A）不能單獨作為耳蝸後病變診斷的依據 （B）正常成人可能的 ILD 為 2 ms
（C）通常做為耳蝸型聽力損失的診斷依據 （D）對耳蝸後病變的偵測敏感度相當低

ChatGPT
For question 29, regarding potential solutions when Wave I of the Auditory Brainstem Response (ABR) is not prominent or unreadable:

(A) **Increasing the intensity of the stimulus**: This is often a valid approach to enhance the visibility of ABR waves, including Wave I, as a louder sound can produce a clearer neural response.

(B) **Increasing the stimulus rate**: Increasing the rate of stimuli can actually make the ABR waves less distinct due to the reduced recovery time for the auditory nerve between stimuli. This approach is unlikely to improve the visibility of Wave I and may further complicate the interpretation of the ABR.

(C) **Using rarefaction clicks**: Rarefaction polarity clicks can sometimes produce clearer ABR waveforms, including Wave I, compared to condensation or alternating clicks. This is because the rarefaction clicks can synchronize neural firing more effectively in some cases.

(D) **Using ear canal electrodes**: Using ear canal electrodes closer to the cochlea can sometimes enhance the amplitude of the ABR waves, including Wave I. This is because the closer proximity to the cochlea can pick up the neural responses more effectively.

The option that is not an effective solution to improve the visibility of Wave I in ABR testing is:
(B) Increasing the stimulus rate.

For question 30, regarding the use of the interaural latency difference (ILD) of Wave V in ABR for diagnostic purposes:

(A) **Cannot be used alone for diagnosing retrocochlear pathology**: This is true. While ILD can be a useful indicator, it should not be used as the sole criterion for diagnosing retrocochlear pathologies such as acoustic neuromas or [illegible]tiple sclerosis.

(B) **Normal adults can have an ILD of 2 ms**: This statement is incorrect. The normal ILD is usually less than 0.2 ms. An ILD of 2 ms is significantly outside the normal range and could indicate a pathology.

(C) **Commonly used for diagnosing cochlear hearing loss**: ILD is more indicative of retrocochlear, not cochlear, problems. Cochlear hearing loss usually does not result in significant ILD.

(D) **Low sensitivity in detecting retrocochlear pathology**: ILD can be a sensitive indicator of retrocochlear pathology, particularly when there is a significant difference between the ears. It is not correct to say that it has low sensitivity for detecting such pathologies.

The correct statement is:
(A) ILD cannot be used alone for diagnosing retrocochlear pathology.

You
Please answer the following multiple-choi[illegible]uestion as a hearing care professional, providing reasoning and explanation: 31 高頻感音性聽損對於 click-ABR 波形之影響，下列何者最為明顯?
（A）第 I 波 （B）第 III 波 （C）第V 波 （D）第VI 波
32 關於 ABR 及 ASSR 之臨床應用比較，下列敘述何者正確?
（A） ASSR 評估嬰幼兒之正常聽力閾值較 ABR 更為準確
（B） ABR 評估極重度聽損嬰幼兒之聽力閾值較 ASSR 更為準確
（C） ABR 較 ASSR 更能評估聽覺神經傳導路徑之異常
（D） ABR 不能像 ASSR 一樣評估不同頻率之聽力閾值

ChatGPT
For question 31, regarding the impact of high-frequency sensorineural hearing loss on click-evoked Auditory Brainstem Response (ABR) waveforms:

- Click stimuli are broad-spectrum, short-duration sounds that predominantly stimulate the basal end of the cochlea, where high-frequency sounds are processed. High-frequency sensorineural hearing loss can impact the responses generated by these hair cells.

(A) **Wave I**: Represents the response from the distal portion of the auditory nerve, near the cochlea. High-frequency hearing loss can [illegible]t Wave I, but it is not usually the most significantly affected component.

(B) **Wave III**: Represents the cochlear nucleus in the brainstem. The effect of high-frequency hearing loss on Wave III is typically less direct compared to Wave I.

(C) **Wave V**: This wave is often the most robust and easiest to identify in ABR testing and can be affected by high-frequency hearing loss. The impact on Wave V can include increased latency and reduced amplitude, making it a significant indicator of such hearing loss in ABR.

(D) **Wave VI**: This wave is less commonly used in clinical analysis and interpretation of ABR, and its changes are not as significant as those observed in Wave V for high-frequency hearing loss.

The most significant impact of high-frequency sensorineural hearing loss in click-ABR is typically seen in:
(C) Wave V.

For question 32, comparing the clinical applications of ABR and ASSR:

(A) **ASSR for normal hearing thresholds in infants**: ASSR is useful for estimating hearing thresholds across a range of frequencies, but it is not necessarily more accurate than ABR for normal hearing in infants. Both tests have their own advantages and limitations.

(B) **ABR for severe hearing loss in infants**: ABR is highly sensitive and is often used for detecting even profound levels of hearing loss in infants. It can be more reliable than ASSR for detecting very severe hearing loss.

(C) **ABR for assessing auditory nerve pathway**: ABR is indeed more focused on the auditory nerve and brainstem pathways and is more sensitive to pathologies along these pathways compared to ASSR.

(D) **ABR for frequency-specific thresholds**: While ABR is typically used for a broad assessment of hearing sensitivity and is not as frequency-specific as ASSR, tone-burst ABR can provide some frequency-specific information.

The correct statement is:
(C) ABR is more capable than ASSR in ass[illegible]g abnormalities in the auditory nerve conduction pathway.

You

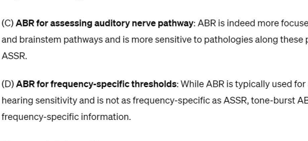

Please answer the following multiple-choice question as a hearing care professional, providing reasoning and explanation: 33 下圖是以奇球刺激法（oddball paradigm）施作電生理檢查 P300 的波形圖，下圖中那一條波形具有
P300?
（A）波形 1 （B）波形 2 （C）波形 3 （D）波形 4

ChatGPT
The P300 wave is an event-related potential elicited in the process of decision making and is typically measured by requiring the subject to actively listen and respond to the stimuli. In an oddball paradigm, the subject is presented with a series of low-probability target tones mixed with high-probability non-target (or "standard") tones. The P300 is usually elicited by the target tones and is characterized by a positive deflection in voltage at a latency of roughly 300 milliseconds.

In the provided graph, we can identify the P300 wave by looking for this characteristic deflection following the target stimulus. It should be most prominent when the subject is attending to the stimuli, rather than ignoring them.

- **Waveform 1 (波形 1)** shows a response to the standard stimulus when the subject is ignoring the stimuli. There should be no prominent P300 since it is not a target stimulus and the subject is not attending.
- **Waveform 2 (波形 2)** shows a response to [illegible] standard stimulus when the subject is attending. Even though attention is given, as this is the standard stimulus, a P300 is not typically elicited in response to it.
- **Waveform 3 (波形 3)** shows a response to the target stimulus when the subject is attending. This is where we would expect to see the P300, as the target stimulus is being attended to, and there is indeed a noticeable positive deflection at the appropriate latency.
- **Waveform 4 (波形 4)** shows the response to the target stimulus when the subject is ignoring the stimuli. We would not expect a prominent P300 here, as the subject is not actively attending to the target.

Therefore, the waveform that exhibits the P300 is:
(C) Waveform 3 (波形 3)

You
Please answer the following multiple-choice question as a hearing care professional, providing reasoning and explanation: 34 耳蝸電圖（ECochG）診斷美尼爾氏病的敘述，下列何者錯誤?
（A） ECochG 診斷美尼爾氏病的敏感度（[illegible]sitivity）約為 90%
（B） ECochG 診斷美尼爾氏病的特異度（specificity）良好
（C）隨著病程緩解，ECochG 的變化可能回復正常
（D）臨床上可同時安排VEMP 檢查，評估內淋巴水腫的範圍
35 ECochG 中的何種組成成分相當於 ABR 之 wave I?
（A） summating potential（SP） （B） cochlear microphonic（CM）
（C） compound action potential（CAP） （D） SP 和 CM

ChatGPT
For question 34, regarding the use of Electrocochleography (ECochG) in the diagnosis of Meniere's disease:

(A) The statement that ECochG has a sensitivity of approximately 90% for Meniere's disease may be overestimated. Sensitivity can vary widely depending on the study and the criteria used for diagnosis.

(B) ECochG does have good specificity for Meniere's disease when there is a significant difference between the summating potential (SP) and the action potential (AP). A large SP/AP ratio is indicative of Meniere's disease, but not exc[illegible]vely.

(C) It is true that as Meniere's disease progresses or fluctuates, the changes in ECochG can potentially return to normal, especially during periods when symptoms are not present.

(D) Vestibular Evoked Myogenic Potentials (VEMP) testing can be used alongside ECochG to assess the extent of endolymphatic hydrops, which is the underlying pathology in Meniere's disease.

The statement that might be considered incorrect due to overgeneralization and variability in sensitivity is:
(A) ECochG's sensitivity for Meniere's disease is approximately 90%.

For question 35, regarding which component of ECochG corresponds to Wave I of the Auditory Brainstem Response (ABR):

(A) The **summating potential (SP)** is a direct current (DC) response that reflects the status of the hair cells in the cochlea and is not equivalent to Wave I.

(B) The **cochlear microphonic (CM)** is an alternating current (AC) response that mirrors the frequency of the stimulus and represents the hair cells' mechanical response. It is not equivalent to Wave I.

(C) The **compound action potential (CAP)** represents the synchronous discharge of the auditory nerve fibers and is considered to be the equivalent of Wave I of the ABR, as they both reflect the activity of the auditory nerve.

(D) Both SP and CM are components of ECochG, but neither corresponds to Wave I of the ABR.

The component of ECochG that is equivalent to Wave I of the ABR is:
(C) Compound action potential (CAP).

You
Please answer the following multiple-choice question as a hearing care professional, providing reasoning and explanation: 36 承上題，該組成成分之來源，為下列何者?
（A） outer hair cell （B） auditory nerve （C） facial nerve （D） cochlear nucleus
37 參照 2007 年JCIH 對嬰幼兒聽障之處理[illegible]，下列敘述何者錯誤?
（A）聽篩未過，3 個月內應完成確診檢查
（B）若確定有聽損，氣導與骨導爆破音 ABR 兩者皆必須做，以確認聽損程度及種類
（C）若懷疑有聽損，而爆破音 ABR 無反應，可以不用做短聲誘發 ABR，應儘早開始介入
（D）若病人有 hyperbilirubinemia，必須測試密極及疏極短聲誘發 ABR

ChatGPT
For question 36, the source of the component of ECochG that corresponds to Wave I of the Auditory Brainstem Response (ABR), which is the compound action potential (CAP), is:

(A) **Outer hair cells**: These cells are responsible for amplifying sound and do not generate the CAP. They are involved in producing other responses measured by ECochG, like the cochlear microphonic (CM).

(B) **Auditory nerve**: The CAP represents the synchronous firing of the auditory nerve fibers in response to sound stimuli and is the equivalent of Wave I of the ABR, which also reflects auditory nerve activity.

(C) **Facial nerve**: This nerve controls the muscles of facial expression and does not contribute to the CAP or any auditory evoked potentials.

(D) **Cochlear nucleus**: This is a collection of neurons in the brainstem where auditory nerve fibers project. While it is involved in the auditory pathway, it is not the source of the CAP or Wave I.

The source of the compound action potential (CAP) is:
(B) Auditory nerve.

For question 37, regarding the 2007 Joint Committee on Infant Hearing (JCIH) guidelines for the management of hearing loss in infants:

(A) The JCIH recommends that infants who do not pass the newborn hearing screening should have diagnostic audiologic evaluation by 3 months of age.

(B) If a hearing loss is confirmed, it is important to determine the type and degree of hearing loss. This may involve both air and bone conduction testing with ABR to differentiate between sensorineural and conductive components.

(C) If there is a suspicion of hearing loss and no responses are obtained from tone-burst ABR, it is not accurate to say that click-evoked ABR should be skipped. Diagnostic evaluation should be comprehensive, and additional tests may be needed to fully characterize the hearing loss before early intervention begins.

(D) In patients with hyperbilirubinemia who are at risk for auditory neuropathy spectrum disorder (ANSD), it is important to test both condensation and rarefaction click-evoked ABRs to assess the integrity of the auditory pathway thoroughly.

The incorrect statement, which is not in line with the JCIH guidelines, is:
(C) If suspected hearing loss is present and tone-burst ABR shows no response, it is necessary to perform a click-evoked ABR to rule out auditory neuropathy and other conditions before proceeding to early intervention.

You
Please answer the following multiple-choice question as a hearing care professional, providing reasoning and explanation: 38 國小三年級[illegible]婷在學校的聽力篩檢雙耳均未通過，但家長質疑在新生兒聽篩通過，而此次為何未通過，下列何項說明最不適當?
（A）新生兒聽力篩檢之 aABR 為快篩方式，可能會錯過輕度聽損
（B）為避免聽篩的可信度問題，須儘快去醫院做完整電生理聽力檢查
（C）中耳狀況也可能影響聽力，應至醫院檢查確認
（D）新生兒聽篩雖通過，仍有晚發性聽力損失的可能
39 有關新生兒聽力篩檢方式之描述，下列何者正確?
（A）行為觀察法（BOA）為客觀性檢查，容易出現信度問題
（B）用制約遊戲式聽力檢查（CPA），對嬰兒也很適合
（C） OAEs 施測快速簡易，可測到內毛細胞
（D） AABR 施測快速簡易，可以測到耳蝸後部位

ChatGPT
For question 38, regarding the concern about a third-grade student, Xiaoting, who failed a school hearing screening despite passing the newborn hearing screening:

(A) The automated Auditory Brainstem Response (aABR) used in newborn hearing screening is a rapid screening method that may miss mild hearing loss. This is a valid point; aABR is sensitive to moderate to profound hearing loss but may [illegible]t detect mild hearing loss.

(B) To avoid issues with the reliability of the screening, it is appropriate to suggest a comprehensive audiological assessment in a clinical setting.

(C) Middle ear conditions can indeed affect hearing. Conditions such as otitis media with effusion are common in children and can cause temporary hearing loss.

(D) Late-onset hearing loss is a possibility, as hearing loss can occur at any age due to various reasons, including genetic factors, infections, noise exposure, and other health conditions.

The least appropriate explanation for Xiaoting's failed screening, given that she passed the newborn hearing screening, is:
(A) While aABR is a quick screening tool that might miss mild hearing loss, it is still designed to be highly sensitive and specific for detecting hearing loss in newborns. Therefore, suggesting that it could "miss" mild hearing loss might not be entirely accurate as it casts doubt on the validity of the screening process. However, it's important to note that no hearing screening method is 100% accurate, and children can indeed develop [illegible]aring loss after passing a newborn screening.

For question 39, regarding descriptions of newborn hearing screening methods:

(A) **Behavioral Observation Audiometry (BOA)** is a subjective test that relies on observing a baby's response to sounds, which can indeed present reliability issues; however, it is typically not used as a screening method for newborns due to its subjectivity.

(B) **Conditioned Play Audiometry (CPA)** is not suitable for infants as it requires a child to be old enough to understand and follow play instructions, usually around the age of 2-5 years.

(C) **Otoacoustic Emissions (OAEs)** testing is quick and easy and measures the function of the outer hair cells in the cochlea, which is correct.

(D) **Automated Auditory Brainstem Response (AABR)** testing is also a quick and easy screening method, but it measures the function of the auditory nerve and brainstem pathways, not specifically the "post-cochlear" sites.

The correct statement is:
(C) OAEs testing is fast and simple and ca[illegible]asure the function of the cochlear outer hair cells.

You
Please answer the following multiple-choice question as a hearing care professional, providing reasoning and explanation: 40 目前使用自動聽性腦幹反應檢查作為嬰幼兒聽力篩檢工具，其儀器設定之音量為何?
（A） 25 dB nHL （B） 35 dB nHL （C） 40 dB nHL （D） 45 dB nHL

41 臺北市新生兒聽力篩檢人數假設有 3000 位嬰兒，使用 aABR 和 OAEs 兩種篩檢工具，其中 20 名嬰兒有聽損未通過，有 5 名有聽損但通過篩檢；2900 名嬰兒通過且沒有聽損，75 名沒通過但沒有聽損。下列何者為此次篩檢之敏感度（sensitivity）?
（A） 90% （B） 80% （C） 75% （D） 70%

ChatGPT

You
Please answer the following multiple-choice question as a hearing care professional, providing reasoning and explanation: 40 目前使用自動聽性腦幹反應檢查作為嬰幼兒聽力篩檢工具，其儀器設定之音量為何?
（A） 25 dB nHL （B） 35 dB nHL （C） 40 dB nHL （D） 45 dB nHL

41 臺北市新生兒聽力篩檢人數假設有 3000 位嬰兒，使用 aABR 和 OAEs 兩種篩檢工具，其中 20 名嬰兒有聽損未通過，有 5 名有聽損但通過篩檢；2900 名嬰兒通過且沒有聽損，75 名沒通過但沒有聽損。下列何者為此次篩檢之敏感度（sensitivity）?
（A） 90% （B） 80% （C） 75% （D） 70%

ChatGPT
For question 40, the intensity setting for automated Auditory Brainstem Response (aABR) used in newborn hearing screenings is typically around:

(A) 25 dB nHL
(B) 35 dB nHL
(C) 40 dB nHL
(D) 45 dB nHL

The typical intensity level used for aABR screening is meant to identify infants who may have moderate or greater degrees of hearing loss. The level is generally set at a level where a baby with normal hearing would definitely respond.

The correct setting is usually:
(D) 45 dB nHL.

This level is chosen to ensure that the screening is sensitive enough to detect most clinically significant hearing losses without producing too many false-positive results.

For question 41, to calculate the sensitivity of the hearing screening program, we use the formula:

Sensitivity = True Positives / (True Positives + False Negatives)

From the given data:

- True Positives (babies with hearing loss who failed the screening) = 20
- False Negatives (babies with hearing loss [illegible] passed the screening) = 5

Now let's calculate the sensitivity:

Sensitivity = 20 / (20 + 5) = 20 / 25

Let's convert this fraction to a percentage to find the correct answer.
The sensitivity of the hearing screening program in this scenario is:
(B) 80%. [>-]

You
Please answer the following multiple-choice question as a hearing care professional, providing reasoning and explanation: 42 短暫誘發耳聲傳射（TEOAEs）在正常聽力新生兒與成人的差異，下列敘述何者正確?
（A）新生兒耳聲傳射的頻率範圍較成人寬
（B）新生兒耳聲傳射的波幅較小
（C）新生兒的殘存噪音（residual noise）量較小
（D）新生兒從出生後 48 小時，耳聲傳射的波幅逐漸下降
43 關於嬰幼兒聽力檢查評估的敘述，下列[illegible]最為適當?
（A）主要以電生理檢查取代行為聽力檢查
（B） 6 歲以前的聽力診斷以電生理檢查為主
（C） 2 歲以後可以進行制約遊戲式聽力檢查（CPA）
（D）視覺增強聽力檢查（visual reinforcement audiometry）是出現視覺刺激後再給予聲音增強

ChatGPT
For question 42, regarding the differences in Transient Evoked Otoacoustic Emissions (TEOAEs) between normal-hearing newborns and adults:

(A) **Frequency range of TEOAEs in newborns compared to adults**: The frequency range of TEOAEs in newborns is not necessarily broader than in adults. The cochlea's frequency response can change as the ear grows, but this doesn't necessarily mean a broader range in newborns.

(B) **Amplitude of TEOAEs in newborns**: It is generally observed that the amplitude of TEOAEs in newborns can be higher compared to adults due to the healthier condition of the outer hair cells and the smaller volume of the ear canal in newborns.

(C) **Residual noise in newborns**: Newborns typically have less residual noise in TEOAEs measurements compared to adults due to less environmental cooperation issues and often less ear canal debris which can affect the measurement.

(D) **Change in TEOAE amplitude after birth**: There is no consistent evidence to suggest that the amplitude of TEOAEs in newborns declines significantly within 48 hours after birth. However, it is common practice to wait at least 12-24 hours post-birth for fluid to clear from the ear canal before conducting TEOAEs to ensure accurate results.

The correct statement about the difference in TEOAEs between normal-hearing newborns and adults is:
(B) Newborns tend to have a higher amplitude of TEOAEs compared to adults.

For question 43, concerning the assessment of hearing in infants and young children:

(A) **Electrophysiological tests replacing behavioral tests**: Electrophysiological tests are often used for very young children because they don't require behavioral responses, but they don't entirely replace behavioral tests when the [illegible]d is old enough to participate in them.

(B) **Use of electrophysiological tests for diagnosis under 6 years old**: While electrophysiological tests are an important diagnostic tool, especially for very young children who can't provide behavioral responses, they are part of a battery of tests and not used exclusively.

(C) **Use of Conditioned Play Audiometry (CPA)**: CPA can be used for children as young as 2 years old and is effective up to the preschool age. It involves training the child to perform an activity each time a sound is heard.

(D) **Visual Reinforcement Audiometry (VRA)**: VRA involves rewarding the child with a visual stimulus (like a toy or video) when they turn toward a sound, not after presenting a visual stimulus.

The most appropriate statement regarding hearing evaluation in infants and young children is:
(C) Conditioned Play Audiometry (CPA) can be conducted for children 2 years and older.

You
Please answer the following multiple-choice question as a hearing care professional, providing reasoning and explanation: 44 針對發展遲[illegible]幼兒進行聽力篩檢時，不建議使用 ABR 的主要原因為何?
（A）無法測得分頻的聽閾值　（B）中樞聽覺傳導路徑的異常
（C）儀器成本太高　（D）檢測成效過低
45 臨床上幼兒檢查報告顯示聽性腦幹反應（ABR）及耳聲傳射（OAEs）皆無反應，下列何項檢查最能協助聽神經病變（auditory neuropathy）的判斷?
（A）同側聽反射檢查　（B）鼓室圖檢查
（C）聽性穩定狀態反應（ASSR）　（D）對側聽反射檢查

ChatGPT
For question 44, the reason ABR might not be recommended for screening hearing in developmentally delayed children is:

(A) **Inability to obtain frequency-specific thresholds**: While ABR is excellent for detecting the presence of a hearing loss, it is not as effective for obtaining detailed frequency-specific information, which is often necessary for fitting hearing aids and planning intervention.

(B) **Abnormalities in the central auditory p[illegible]hway**: ABR primarily assesses the integrity of the auditory nerve and lower brainstem pathways, not the higher auditory processing centers. Abnormal ABR results in a child with developmental delays might indicate a retrocochlear pathology, but it doesn't necessarily reflect central auditory processing disorders.

(C) **High cost of equipment**: While the cost of ABR equipment is high, it is a standard tool in audiology clinics, and the cost is not typically a barrier for its use in clinical settings.

(D) **Low testing efficacy**: ABR is a highly effective test for assessing auditory nerve function and is not known for low efficacy.

The primary reason not to use ABR for hearing screening in developmentally delayed children is:
(A) It does not provide frequency-specific thresholds, which are often critical in the diagnostic and rehabilitation process for this population.

For question 45, when a clinical report shows no responses for both ABR and OAEs, the test that could be most helpful in the diagnosis of auditory neuropathy (a condition where the inner ear can receive sound, but the transmission of signals from the inner ear to the brain is impaired) is:

(A) **Ipsilateral acoustic reflex testing**: This [illegible] measures the contraction of the middle ear muscles in response to sound but is not definitive for diagnosing auditory neuropathy.

(B) **Tympanometry**: This test evaluates middle ear function, which can help rule out middle ear pathologies but does not confirm auditory neuropathy.

(C) **Auditory Steady State Response (ASSR)**: ASSR can provide frequency-specific threshold information and can be used to assess the integrity of the auditory pathway beyond the cochlea, which is useful in diagnosing auditory neuropathy.

(D) **Contralateral acoustic reflex testing**: Like the ipsilateral reflex, this test is not definitive for diagnosing auditory neuropathy.

The test that can best assist in the diagnosis of auditory neuropathy when both ABR and OAEs show no responses is:
(C) Auditory Steady State Response (ASSR).

You
Please answer the following multiple-choice question as a hearing care professional, providing reasoning and explanation: 46 小乖因車禍耳朵受傷，去看耳鼻喉科，診斷為聽小骨斷裂。最不可能出現下列何種檢查結果?
（A） PTA 結果為輕度聽損，鼓室圖為Type Add
（B） ABR 結果為中度聽損，鼓室圖為Type As
（C） PTA、ABR 結果為中度聽損，鼓室圖為Type E
（D） PTA 結果為輕度聽損，高頻鼓室圖尖峰多，聽反射異常
47 一個 6 個月大的嬰幼兒個案，出生時雖通過新生兒聽力篩檢，但因中耳炎來接受聽力評估，其 1000 Hz 鼓室圖以及聽性腦幹反應（ABR）最可能呈現何種型態?
（A）平坦，ABR 在正常範圍內　（B）尖峰，ABR 在正常範圍內
（C）平坦，ABR 不在正常範圍內　（D）尖峰，ABR 不在正常範圍內

ChatGPT
For question 46, considering an injury that has led to ossicular chain discontinuity, such as from a car accident, we would expect certain findings on audiological tests:

(A) **Pure Tone Audiometry (PTA) showing mild hearing loss and a Type Ad tympanogram**: Type Ad (deep) tympanograms are associated v[illegible] high compliance of the middle ear system, such as with ossicular chain discontinuity. However, the result showing only mild hearing loss with this tympanogram type is inconsistent, as ossicular chain disruption typically leads to a conductive hearing loss that is often more than mild.

(B) **ABR showing moderate hearing loss and a Type As tympanogram**: Type As (shallow) tympanograms suggest a stiff middle ear system, which could occur if the ossicular chain is fixed or immobilized, not necessarily disrupted. This could be consistent with an ossicular chain fracture.

(C) **PTA and ABR showing moderate hearing loss and a Type E tympanogram**: Type E tympanograms are not standard and don't provide a clear expected result. This could be a typographical error or a misunderstanding of tympanogram types.

(D) **PTA showing mild hearing loss, high-frequency tympanogram with multiple peaks, and abnormal acoustic reflex**: Multiple peaks in a high-frequency tympanogram are not a typical finding and don't correspond to a known tympanogram type. Acoustic reflexes could be abnormal in the case of ossicular discontinuity.

The least likely finding in the case of ossicular chain discontinuity would be:
(A) PTA results indicating mild hearing loss with a Type Ad tympanogram. While the Type Ad tympanogram could be consistent with ossicular discontinuity, the associated hearing loss would likely be more than mild.

For question 47, a 6-month-old infant who passed the newborn hearing screening but is now being evaluated for hearing due to otitis media, the 1000 Hz tympanogram and Auditory Brainstem Response (ABR) would most likely show:

(A) **Flat tympanogram, ABR within normal range**: Otitis media commonly results in a flat tympanogram due to the presence of fluid in the middle ear, which impedes the movement of the tympanic membrane and ossicles. If the middle ear infection affects conductive hearing mechanisms, it might not affect the ABR, which measures the neural response and could still be within the normal range.

(B) **Peaked tympanogram, ABR within normal range**: A peaked tympanogram would be unexpected in the presence of middle ear [illegible]sion.

(C) **Flat tympanogram, ABR not within normal range**: A flat tympanogram is expected with middle ear effusion, but the ABR could still be within the normal range unless there is a co-existing sensorineural hearing loss or the conductive component is affecting the ABR results.

(D) **Peaked tympanogram, ABR not within normal range**: Again, a peaked tympanogram would be unexpected in the presence of middle ear effusion, and an abnormal ABR would suggest a neural component to the hearing loss, which is not typical in isolated otitis media.

The most likely pattern for a child with otitis media would be:
(A) Flat tympanogram, ABR within normal range.

You
Please answer the following multiple-choice question as a hearing care professional, providing reasoning and explanation: 48 10 個月大嬰兒曾因新生兒黃疸接受換血治療，鼓室圖正常但高音強之短聲誘發聽性腦幹反應檢查呈現無反應，接下來應優先安排：
（A）耳聲傳射（OAEs）及視覺增強聽覺檢查（visual reinforcement audiometry）
（B）聽性穩定狀態反應（ASSR）
（C）依短聲誘發聽性腦幹反應（click evoked ABR）結果選配助聽器
（D）爆破音聽性腦幹反應（tone burst ABR）
49 下列何項聽力檢查對於聽性腦幹反應的神經診斷判讀最為重要?
（A）聲微增敏感指數檢查（short increment sensitivity index, SISI）、鼓室圖
（B）閾上適應測驗（suprathreshold adaptation test）、字詞辨識測驗（word recognition score）
（C）聽反射檢查、字詞辨識測驗
（D）純音聽力檢查、鼓室圖

ChatGPT
For question 48, in a 10-month-old infant who has undergone exchange transfusion for neonatal jaundice and presents with a normal tympanogram but no response to a high-intensity click-evoked Auditory Brainstem Response (ABR), the next steps in assessment would likely include:

(A) **Otoacoustic Emissions (OAEs) and Visual Reinforcement Audiometry (VRA)**: OAEs can be used to assess the function of the cochlear outer hair cells, which if present, may suggest normal cochlear function and the possibility of aud[illegible]ry neuropathy. VRA is a behavioral test that might be difficult to interpret reliably at 10 months due to the developmental age of the child.

(B) **Auditory Steady State Response (ASSR)**: This test can provide frequency-specific threshold information, which is essential for confirming the type and degree of hearing loss, especially if auditory neuropathy is suspected.

(C) **Selection of hearing aids based on click-evoked ABR results**: If the ABR showed no response, it suggests a significant hearing impairment, but further assessment is needed to confirm the type and degree of loss before hearing aids can be considered.

(D) **Tone burst ABR**: This test can provide frequency-specific threshold information, similar to ASSR, and might be useful if the click-evoked ABR was not informative due to its broad frequency range.

The priority should be to confirm the type and degree of hearing loss, and also rule out or confirm auditory neuropathy, which is a risk associated with neonatal jaundice and exchange transfusion. Therefore, the next step would be:
(B) Auditory Steady State Response (ASSF[illegible]

For question 49, regarding the most important tests for the neurodiagnostic interpretation of Auditory Brainstem Response (ABR):

(A) **Short Increment Sensitivity Index (SISI) and Tympanometry**: SISI is a test for cochlear function, specifically for detecting recruitment, which is associated with cochlear pathology. Tympanometry assesses middle ear function. Neither directly informs ABR interpretation in terms of neural pathways.

(B) **Suprathreshold adaptation test and Word Recognition Score (WRS)**: These tests are more relevant to cochlear function and speech processing, not directly to the neurodiagnostic interpretation of ABR.

(C) **Acoustic reflex testing and WRS**: The acoustic reflex involves the auditory nerve and brainstem reflex pathways, which are also involved in ABR, making it somewhat relevant. However, WRS again is more about cochlear and higher auditory pathway function.

(D) **Pure Tone Audiometry and Tympanom[illegible]y**: Pure tone audiometry provides a frequency-specific threshold of hearing, which can be compared with ABR findings to determine if there is a neural component to any hearing loss identified. Tympanometry assesses the middle ear function, which is necessary to ensure that ABR results are not influenced by middle ear pathology.

The most important tests for neurodiagnostic interpretation of ABR would be:
(D) Pure Tone Audiometry and Tympanometry. These tests provide essential information about the auditory threshold and middle ear status, which helps in interpreting ABR results in the context of the auditory pathway's neural integrity.

You
Please answer the following multiple-choice question as a hearing care professional, providing reasoning and explanation: 50 聽力篩檢工具的特異性（specificity）為 60%，其代表意義下列何者正確?
（A） false negative 60%　（B） true negative 60%　（C） false positive 60%　（D） true positive 60%

ChatGPT
Specificity in the context of a hearing screening tool (or any diagnostic test) refers to the ability of the test to correctly identify those who do not have the condition (in this case, hearing loss). In other words, it is the proportion of true negatives among all individuals who are actually healthy or normal. A specificity of 60% means that 60% of those without hearing loss are correctly identified by the screening as not having hearing loss.

Breaking down the options:

(A) **False negative 60%**: This would mean that 60% of the individuals with hearing loss are incorrectly identified as not having hearing loss. This is related to sensitivity, not specificity.

(B) **True negative 60%**: This is correct. It means that 60% of the individuals without hearing loss are correctly identified by the test as not having hearing loss.

(C) **False positive 60%**: A false positive rate of 60% would imply that 60% of individuals without hearing loss are incorrectly identified as having it, which is the opposite of specificity.

(D) **True positive 60%**: This would mean that 60% of individuals with hearing loss are correctly identified as having it. This is related to sensitivity, not specificity.

Therefore, the correct interpretation of a specificity of 60% is:
(B) True negative 60%.

Message ChatGPT...

ChatGPT can make mistakes. Consider checking important information.
