# Supplementary material for: Exploring the Performance of ChatGPT-4 in the Taiwan Audiologist Qualification Examination: Preliminary Observational Study Highlighting the Potential of AI Chatbots in Hearing Care
Source: JMIR Med Educ. 2024 Apr 26;10:e55595. doi: 10.2196/55595 (PMC11067446; doi:10.2196/55595)
Supplement: Multimedia Appendix 5 [file mededu-v10-e55595-s005.pdf]

ChatGPT 4

You
Please answer the following multiple-choice question as a hearing care professional, providing reasoning and explanation: 1 下列何種聽力損失程度，較不宜使用壓縮限制來控制助聽器的最大輸出音量?
（A）輕度聽力損失 （B）中度聽力損失 （C）重度聽力損失 （D）極重度聽力損失

ChatGPT
As a hearing care professional, it's important to understand how compression limiting in hearing aids affects different degrees of hearing loss. Compression limiting controls the maximum output volume of the hearing aid, preventing it from getting too loud and potentially causing discomfort or further hearing damage.

Let's break down the options:

1. **Mild Hearing Loss (A)**: Individuals with mild hearing loss have some difficulty hearing soft sounds. They generally benefit from a slight amplification of these sounds without needing significant compression limiting, as their tolerance for loud sounds is usually closer to normal levels.
2. **Moderate Hearing Loss (B)**: In moderate hearing loss, there's a greater need for amplification. However, these individuals still retain a reasonable tolerance for louder sounds, so compression limiting can be useful but is not as critical as in more severe cases.
3. **Severe Hearing Loss (C)**: For severe hearing loss, significant amplification is needed. Here, compression limiting becomes more important to protect the user from sudden loud sounds that could be uncomfortable or damaging. However, they still need some level of loud sound to be perceptible.
4. **Profound Hearing Loss (D)**: Individuals with profound hearing loss require the most amplification, but their tolerance for loud sounds can be very low. Over-compressing the sound can make it difficult for them to hear anything at all. Therefore, using compression limiting to control maximum output volume might not be ideal. They need a more finely balanced amplification where even loud sounds are amplified to a level that is just enough for them to hear.

Given these considerations, the most appropriate answer is **(D) Profound Hearing Loss**. For individuals with profound hearing loss, the use of compression limiting to control the maximum output can be counterproductive, as they need a careful balance to ensure that they can hear both soft and loud sounds adequately. The goal is to provide sufficient amplification without over-compressing the sound, which could make it inaudible to them.

You
Please answer the following multiple-choice question as a hearing care professional, providing reasoning and explanation: 2 耳模所選擇的音管（tubing），編號數字愈大代表何種意義?
（A）不同的材質 （B）管壁愈厚 （C）外徑愈大 （D）內徑愈大
3 助聽器使用方向性麥克風時，易造成下列何種現象?
（A）低頻增益不足 （B）迴授問題 （C）閉塞效應增大 （D）內部噪音過大

ChatGPT
Let's address each question in turn:

Question 2

The question is about the significance of the numbering of the tubing in earmolds. The options are:

- **(A) Different Material**
- **(B) Thicker Wall**
- **(C) Larger Outer Diameter**
- **(D) Larger Inner Diameter**

In earmolds, the numbering of the tubing typically corresponds to the diameter of the tubing. This diameter can refer to either the inner or outer diameter. Generally

You
Please answer the following multiple-choice question as a hearing care professional, providing reasoning and explanation: 2 耳模所選擇的音管（tubing），編號數字愈大代表何種意義?
（A）不同的材質 （B）管壁愈厚 （C）外徑愈大 （D）內徑愈大
3 助聽器使用方向性麥克風時，易造成下列何種現象?
（A）低頻增益不足 （B）迴授問題 （C）閉塞效應增大 （D）內部噪音過大

ChatGPT

Question 2: Significance of Larger Numbering on Tubing in Earmolds

The options provided are:

- **(A) Different Material**
- **(B) Thicker Wall**
- **(C) Larger Outer Diameter**
- **(D) Larger Inner Diameter**

In hearing aid earmolds, the tubing is a critical component that connects the earmold to the hearing aid. The numbering of the tubing usually refers to its size. In most cases, a larger number indicates a larger diameter of the tubing. This can refer to either the inner diameter or the outer diameter, but it is more commonly associated with the inner diameter, as this affects the amount and quality of sound transmission. The material and wall thickness are typically consistent across different sizes unless specifically stated otherwise. Therefore, the correct answer is **(D) Larger Inner Diameter**.

Question 3: Effect of Directional Microphones in Hearing Aids

The options are:

- **(A) Insufficient Low-Frequency Gain**
- **(B) Feedback Issues**
- **(C) Increased Occlusion Effect**
- **(D) Excessive Internal Noise**

Directional microphones in hearing aids are designed to improve speech understanding in noisy environments by focusing the microphone's sensitivity towards the front, reducing the pickup of sounds from other directions. However, this directional focus can lead to certain limitations or issues:

- **Insufficient Low-Frequency Gain (A)**: Directional microphones are more effective in reducing background noise at higher frequencies. They may not significantly impact low-frequency sounds, but they do not typically cause a reduction in low-frequency gain.
- **Feedback Issues (B)**: While feedback can be a concern in hearing aids, it is more related to the fit and seal of the earmold or the hearing aid's overall design and not directly tied to the use of directional microphones.
- **Increased Occlusion Effect (C)**: The occlusion effect is related to the physical blockage of the ear canal by the hearing aid or earmold. It is not directly influenced by the microphone type.
- **Excessive Internal Noise (D)**: Directional microphones, by focusing on sounds from a specific direction, can sometimes amplify internal circuit noise or handling noise, leading to a perception of increased internal noise.

Given these considerations, the most appropriate answer is **(D) Excessive Internal Noise**. Directional microphones, by their nature of focusing and sometimes amplifying specific sound directions, can lead to the inadvertent amplification of internal noises within the hearing aid.

You

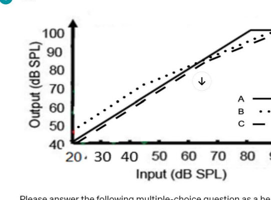

Please answer the following multiple-choice question as a hearing care professional, providing reasoning and explanation: 過大下圖為三個不同助聽器在 2000 Hz 的「輸入 - 輸出」函數圖，請依序回答第 4 題至第 7 題：

4 在輸入音強約為多少時，三個助聽器的輸出音強最接近?
（A） 30 dB SPL （B） 45 dB SPL （C） 65 dB SPL （D） 80 dB SPL
5 助聽器 B 的第一個壓縮點約在什麼位置?
（A） 30 dB SPL （B） 45 dB SPL （C） 65 dB SPL （D） 80 dB SPL
6 助聽器 A 最有可能是下列那一個選配公式的輸入 - 輸出函數圖?
（A） NAL-NL1 （B） NAL-NL2 （C） DSL （D） POGO
7 有一位個案曾使用助聽器 20 年，長期慣用手調式類比助聽器並表示喜歡大的聲音，圖中的那一個助聽器最可能適合他?
（A） A （B） B （C） C （D）都不適合

ChatGPT
Given the graph you've provided, let's address each question:

Question 4: Closest Output Intensity for All Hearing Aids

We need to determine at which input level the output of all three hearing aids is closest to each other. Looking at the graph, we focus on the points where the three lines representing the hearing aids' outputs converge.

- **(A) 30 dB SPL**: At this input level, the three lines appear to diverge, so it is not likely the correct answer.
- **(B) 45 dB SPL**: The lines are still somewhat apart at this input level.
- **(C) 65 dB SPL**: Here, the three lines are close to converging, indicating that the output of all three hearing aids is very similar.
- **(D) 80 dB SPL**: At this input level, the lines have diverged again.

The answer is **(C) 65 dB SPL**, where the outputs of the three hearing aids are closest to one another.

Question 5: Compression Knee Point for Hearing Aid B

The compression knee point is where the output no longer increases linearly with the input, indicating the start of compression. For Hearing Aid B, we identify this point on the graph where the line transitions from a steep slope to a flatter slope.

- **(A) 30 dB SPL**: The line for Hearing Aid B still has a steep slope.
- **(B) 45 dB SPL**: The graph shows the line for Hearing Aid B beginning to flatten.
- **(C) 65 dB SPL**: The line has already flattened out, suggesting the knee point is at a lower input level.
- **(D) 80 dB SPL**: The line is already in the compression region, so this is past the knee point.

The answer is **(B) 45 dB SPL**, where the slope of the line for Hearing Aid B changes, indicating the start of compression.

Question 6: Fitting Formula for Hearing Aid A

The input-output function for different fitting formulas has characteristic shapes. Without specific knowledge of the exact shapes for NAL-NL1, NAL-NL2, DSL, and POGO, it's not possible to provide a definitive answer. Generally:

- **NAL-NL1** and **NAL-NL2** are known for trying to balance speech intelligibility with loudness comfort.
- **DSL** (Desired Sensation Level) tends to provide more gain for softer sounds and less for louder sounds to make speech more intelligible, especially in children.
- **POGO** (Prescription Of Gain and Output) provides a linear gain up to a certain output level.

Based on typical characteristics, since Hearing Aid A's graph shows a more linear relationship before reaching the compression knee point, the most likely fitting formula could be the POGO or a similar linear formula. However, without further information, this is speculative.

Question 7: Suitable Hearing Aid for a User Accustomed to Analog Hearing Aids

The individual has used hand-tuned analog hearing aids and prefers a louder sound. Analog hearing aids are typically linear in amplification, without compression.

- **Hearing Aid A**: Shows a more linear relationship before compression, which may be more similar to the user's previous experience.
- **Hearing Aid B**: Shows compression starting at a lower input level.
- **Hearing Aid C**: Appears to have the most compression, which is likely the least similar to an analog hearing aid.

The answer is likely **(A) Hearing Aid A**, as it offers a more linear amplification that the individual is accustomed to and prefers.

Please note that actual selection should be based on a comprehensive audiometric evaluation and the individual's specific preferences and needs.

You
please give the most suitable answer for Q6

ChatGPT
Without access to specific details about the fitting formulas, it's challenging to give a definitive answer for Question 6. However, I can provide general insights based on common characteristics associated with these fitting formulas.

- **NAL-NL1 and NAL-NL2**: These National Acoustic Laboratories' non-linear fitting formulas are designed to maximize speech intelligibility while maintaining wearer comfort. They typically show a smooth transition from the linear to the compressed region and may not have a clearly defined knee point.
- **DSL (Desired Sensation Level)**: This fitting approach is designed to ensure speech sounds are audible and comfortable for all pitches and intensities. It often shows more gain for soft sounds, ensuring they are audible, and less gain for louder sounds to prevent discomfort.
- **POGO (Prescription Of Gain and Output)**: It tends to provide a linear gain up to a certain output level and is characterized by a clear compression knee point where the output gain begins to level off.

Based on the graph provided and the general knowledge of fitting formulas:

- **Hearing Aid A** has a fairly linear relationship up to the point of compression and might be indicative of a POGO fitting strategy because of its linear progression before the knee point.
- **Hearing Aid B** has an early knee point, which may suggest a fitting strategy that is protective against loud sounds, possibly like DSL, which is conservative with loudness for the wearer's comfort.
- **Hearing Aid C** seems to have the most aggressive compression and could be indicative of one of the NAL-NL formulas, which are known for their sophisticated compression strategies to balance speech intelligibility with loudness comfort.

Given these characteristics, if we must choose which fitting formula Hearing Aid A represents based on the graph alone, **(D) POGO** might be the most suitable answer, as the output for Hearing Aid A appears to increase linearly before reaching the knee point, which is typical for POGO. However, this is a general interpretation, and audiologists would consider many other factors, including the patient's audiometric profile and personal preferences, before making a decision.

You
Please answer the following multiple-choice question as a hearing care professional, providing reasoning and explanation: 8 助聽器麥克風會受到風切聲的影響，有關風切聲之敘述，下列何者錯誤?
（A）風切聲之主要組成頻率為中低頻
（B）若助聽器佩戴者在脖子戴上圍巾，將導致風切聲變大
（C）將發泡塑膠放在麥克風聲孔上，可降低風切聲的影響
（D）將麥克風聲孔放得越深入耳道，風切聲的影響會越小
9 關於助聽器的內部噪音，下列敘述何者錯誤?
（A）高增益助聽器通常會比低增益助聽器的內部噪音來得大
（B）主要來源為麥克風及放大器
（C）常與迴授現象伴隨發生
（D）經由量化表示為 equivalent input noise（EIN）

ChatGPT

Question 8: Misconception About Wind Noise in Hearing Aids

Wind noise is generated when moving air hits the microphone, and it can be a significant issue for hearing aid users. Let's analyze the options:

- **(A)** The main components of wind noise are indeed typically in the low to mid-frequency range due to the turbulence created around the hearing aid microphone.
- **(B)** Wearing a scarf around the neck could actually help to reduce the impact of wind on the microphone by blocking direct wind flow, contrary to increasing it.
- **(C)** Using foam or other windscreen materials over the microphone port can decrease the impact of wind noise by disrupting the flow of air across the microphone.
- **(D)** Placing the microphone port deeper in the ear canal can indeed reduce the effect of wind noise because it is more shielded from direct wind.

Given these considerations, the incorrect statement is **(B) If the hearing aid wearer puts on a scarf around the neck, it will lead to increased wind noise.** In fact, a scarf can act as a windscreen and reduce the wind noise.

Question 9: Misconception About Internal Noise in Hearing Aids

Internal noise in a hearing aid can be influenced by various factors. Let's evaluate the options:

- **(A)** Higher gain hearing aids can indeed have more noticeable internal noise because the internal components' noise is also amplified.
- **(B)** The primary sources of internal noise are the microphone and the amplifier, which is correct.
- **(C)** Internal noise is different from feedback. Feedback is a specific phenomenon where amplified sound from the hearing aid speaker is picked up by the microphone and re-amplified, creating a loop that results in a whistling sound. It's not typically described as occurring alongside internal noise.
- **(D)** Equivalent input noise (EIN) is a measure used to quantify the internal noise of a hearing aid by representing the sound level that would produce an equivalent output if it were entering the microphone from the outside.

Given these points, the incorrect statement is **(C) Internal noise often occurs alongside feedback phenomena.** While both can be present in a hearing aid, they are separate issues and do not necessarily occur together. Feedback is a loop issue, while internal noise is constant and is not related to the loop phenomenon of feedback.

You
Please answer the following multiple-choice question as a hearing care professional, providing reasoning and explanation: 10 測量前將控制麥克風（control microphone）放置在測試箱內指定測試位置，測量並儲存每一頻率實際聲壓值和期望值的差異。在隨後的測量中，測試箱調整其輸出音量並代償這些差異，此運作方法稱為：
（A）壓力法（pressure method） （B）取代法（substitution method）
（C）校正法（calibration method） （D）共振法（resonance method）
11 在 ANSI S3.22 的規範中，助聽器輸出範圍的增益值應低於高頻平均增益值（high-frequency average gain）多少分貝?
（A） 12 dB （B） 15 dB （C） 17 dB （D） 20 dB

ChatGPT

Question 10: Measurement Method for Hearing Aid Calibration

The described method involves placing a control microphone in a specified test position within a test box, measuring, and storing the difference between actual sound pressure levels and expected values at each frequency. Then, during subsequent measurements, the test box adjusts its output to compensate for these differences. This process is known as:

- **(A) Pressure Method**: This method typically refers to a procedure used to measure the output of a hearing aid within a test box or coupler.
- **(B) Substitution Method**: This method usually involves substituting one piece of equipment for another to compare measurements, but it doesn't typically involve compensation for differences in expected values.
- **(C) Calibration Method**: Calibration is the process of adjusting equipment to ensure that its output matches specified values. This option seems to describe the method you're referring to, where the test box is calibrated to account for the differences in sound pressure levels at each frequency.
- **(D) Resonance Method**: This method would involve the identification of resonance frequencies, which is not what is being described here.

The correct answer is **(C) Calibration Method** because the process described involves measuring and adjusting the output to match the expected values, which is essentially what calibration is.

Question 11: ANSI S3.22 Specification for Hearing Aid Output Range

The American National Standards Institute (ANSI) S3.22 standard specifies the performance characteristics of hearing aids, including how much the gain value of the hearing aid's output range should be below the high-frequency average gain. Without the specific text of the ANSI S3.22 standard in front of me, I cannot provide the exact value. However, these standards are set to ensure that hearing aids meet certain performance criteria, and the difference in gain value is specified to ensure comfort and audibility across the frequency range.

Based on general knowledge, the gain at output range would be lower than the high-frequency average gain to prevent discomfort or damage from excessive loudness at high frequencies. The exact value would be determined by the latest version of the ANSI standard, which could be any of the values listed. However, without access to current resources or databases, I cannot verify the current standard.

To find the correct dB difference as per the current ANSI S3.22 standards, one would need to refer to the latest version of the ANSI S3.22 document, which is typically available for purchase from standards organizations or may be accessed through professional or academic institutions associated with audiology or hearing sciences.

You
please give the most suitable answer for Q11

ChatGPT
For Question 11 regarding the ANSI S3.22 standard, without the most current documentation or reference material, it is challenging to provide the exact number of decibels by which the gain value of a hearing aid's output range should be below the high-frequency average gain. These standards are subject to change and it is important to reference the latest version of the ANSI S3.22 standard for the most accurate and up-to-date information.

In the context of hearing aid specifications, the high-frequency average gain is a critical value that is used to ensure audibility and comfort for high-frequency sounds. The difference between the overall output range and the high-frequency average gain is set to prevent the hearing aid from providing too much gain at high frequencies, which could lead to discomfort or further hearing damage.

The values given in the options (A) 12 dB, (B) 15 dB, (C) 17 dB, and (D) 20 dB, represent possible decrements. In practice, a common value that has been used historically in some standards for the difference between the full-on gain and high-frequency average gain is around 17 dB, which could be the most likely answer. However, this is based on historical context and may not reflect the latest ANSI S3.22 standards.

Therefore, in a testing or examination setting without access to the specific standard, one would typically choose the value that is most commonly referenced in the field, with the caveat that the actual current standard should be verified for accuracy. If I must provide an answer based on common practice, I would suggest:

The answer might be **(C) 17 dB**, but please verify with the current ANSI S3.22 standard for the most accurate information.

You
Please answer the following multiple-choice question as a hearing care professional, providing reasoning and explanation: 12 王同學上個月完成助聽器選配，並且經過真耳測試，其助聽器的輸出曲線均符合選配公式的目標值。若將助聽器耳模氣孔從 1mm 換成 3mm，真耳測試 REAR 項目的結果會有什麼變化?
（A）沒有變化 （B） 250 Hz 處的輸出音壓值變低
（C） 4000 Hz 處的輸出音壓值變高 （D）比較不會產生迴授音
13 選擇耳模和耳殼聲學的程序，有以下步驟，下列何者是最適合的優先順序? ①找到可能的最大通氣孔尺寸 ②確定通氣孔大小 ③估算所需的最小通氣孔尺寸 ④選擇阻尼器 ⑤選擇聲孔的模型
（A） [illegible] （B） [illegible] （C） ③①②④⑤ （D） [illegible]

ChatGPT

Question 12: Changes in Real-Ear Aided Response (REAR) with Larger Vent

Changing the size of the vent in a hearing aid earmold from 1mm to 3mm affects the acoustic properties of the earmold and the hearing aid's performance. Here's how it affects the REAR:

- **(A) No Change**: Changing the vent size will definitely create a change in the REAR due to altered acoustics.
- **(B) The output sound pressure level at 250 Hz becomes lower**: A larger vent allows more low-frequency sounds to escape from the ear canal, which will likely reduce the low-frequency gain in the ear canal, hence lowering the SPL at 250 Hz.
- **(C) The output sound pressure level at 4000 Hz becomes higher**: Typically, the vent size does not significantly affect the high-frequency response because high frequencies are less likely to travel through the vent due to their shorter wavelengths.
- **(D) Less likely to produce feedback**: A larger vent can indeed reduce the likelihood of feedback by reducing the occlusion effect and allowing sound pressure to escape from the ear canal.

The correct answer is **(B) The output sound pressure level at 250 Hz becomes lower** because the larger vent size will allow more of the low-frequency sounds to escape, which results in a lower sound pressure level at those frequencies.

Question 13: Proper Sequence for Selecting Earmold and Earshell Acoustics

The steps given are part of the process for selecting the acoustic properties of an earmold or earshell for a hearing aid. The proper sequence would typically be:

- **① Finding the possible largest vent size**: This is usually not the first step since it needs to be determined based on other factors such as gain and feedback prevention.
- **② Determining the vent size**: You can't determine the vent size without knowing the required and possible vent sizes first.
- **③ Estimating the required minimum vent size**: This is often one of the first steps, as it is based on the patient's hearing loss and other factors such as occlusion effect and feedback.
- **④ Choosing a damper**: Dampers are selected to control the acoustic properties of the hearing aid, often after the vent size has been determined.
- **⑤ Choosing the model of the sound bore**: This is often one of the later steps after vent size and dampers have been considered.

A logical sequence would involve estimating the required minimum vent size to meet the acoustic needs based on hearing loss, then finding the maximum possible vent size to ensure comfort and prevent feedback, and then determining the actual vent size based on these constraints. Afterward, you would select any necessary acoustic modifications like dampers, and finally choose the model of the sound bore.

The most suitable sequence, considering the above, would be **(C) ③①②④⑤**. This sequence starts with estimating the necessary acoustic requirements, finding the maximum possible size for comfort, and then determining the specific details of the vent and any additional acoustic modifications before finalizing the model.

You
Please answer the following multiple-choice question as a hearing care professional, providing reasoning and explanation: 14 108 年衛生福利部中央健康保險署公告人工耳蝸適應症明訂「兒童」為幾歲?
（A） 3 歲 （B） 6 歲 （C） 12 歲 （D） 18 歲
15 下列非線性的助聽器選配公式均只需輸入聽力閾值，但何者除外?
（A） IHAFF （B） NAL-NL2 （C） CAM2 （D） DSL m [i/o]

ChatGPT
As of my last update in April 2023, I don't have the specific information about the age definition of "children" according to the Health and Welfare Department of Taiwan for the adaptation of cochlear implants. Health insurance policies and medical guidelines can vary by country and over time, so it's important to refer to the most current local guidelines for this information. Typically, "children" in medical contexts can refer to anyone under the age of 18, but for specific treatments like cochlear implants, the age criteria might be set differently based on clinical evidence about the effectiveness of the treatment at certain ages.

For Question 15 regarding nonlinear hearing aid fitting formulas that require inputs beyond audiometric thresholds, IHAFF, NAL-NL2, and DSL m[i/o] are well-known fitting formulas. Here's what is typically required for these formulas:

- **IHAFF (Independent Hearing Aid Fitting Forum)**: This formula typically requires audiometric thresholds and might also require other factors such as loudness scaling or uncomfortable levels.
- **NAL-NL2 (National Acoustic Laboratories' Non-Linear 2)**: This is a well-known formula that uses audiometric thresholds as well as other factors such as patient age and the hearing aid's input-output characteristics.
- **CAM2 (Client Oriented Scale of Improvement 2)**: CAM2 is designed to be a comprehensive approach that can take into account factors beyond audiometric thresholds, such as listener preferences and communication needs.
- **DSL m[i/o] (Desired Sensation Level multistage [input/output])**: This fitting approach typically uses thresholds and a variety of other inputs to provide a prescribed gain at different input levels for different frequencies.

Based on this information, while all these formulas start with audiometric thresholds, it is likely that one of them may require additional information such as loudness discomfort levels, patient age, or speech dynamic range. Since NAL-NL2 and DSL are known to sometimes require additional inputs, and without the specific details of IHAFF and CAM2, it's difficult to conclusively state which one would be the exception. Typically, DSL m[i/o] is known for its detailed input requirements, including in/out measurements which suggests it could potentially be the answer. However, it's important to refer to the latest documentation for each fitting formula to understand their specific requirements.

For the most accurate answers to both questions, you should refer to the most recent guidelines from the Central Health Insurance Agency for the age definition of "children" for cochlear implants and the documentation for each fitting formula.

You
please give the most suitable answer for Q14&15

ChatGPT
For Question 14, without the specific documentation from the Health and Welfare Department of Taiwan, it would be speculative to provide an exact age defined as "children" for the purposes of cochlear implant adaptation. However, in many medical contexts, the age cut-off for pediatric services is often considered to be 18 years old, as this is the age of majority in many jurisdictions. Therefore, in a general sense, **(D) 18 years old** would often be considered the upper limit for defining pediatric populations. However, the specific age defined by the Taiwan Health and Welfare Department for cochlear implants may be different and would need to be verified with the current local regulations.

For Question 15, regarding nonlinear hearing aid fitting formulas, typically all of these formulas would start with audiometric thresholds, but some may require additional information. The DSL m[i/o] fitting formula is known for its detailed consideration of the hearing aid's input/output characteristics and the wearer's loudness discomfort levels, among other factors. Therefore, the exception, which requires more than just audiometric thresholds, is likely **(D) DSL m[i/o]**, as it is known to require a more complex set of data for its calculations.

You
Please answer the following multiple-choice question as a hearing care professional, providing reasoning and explanation: 16 當助聽器使用者抱怨，在安靜環境下依舊無法理解輕柔的語音，若要解決此一抱怨，下列何者最不適當?
（A）請個案告知是否能聽見低頻線索的詞彙或是高頻線索的語音，以分辨需要提高增益量的頻道
（B）評量時使用的語音應設定在 45 dB SPL 左右
（C）針對受影響頻道降低壓縮閾值和提高壓縮比
（D）增加低音量輸入音的增益

17 嬰幼兒的耳道殘餘容積（耳模前端以內的體積）與成人相比，下列敘述何者錯誤?
（A）嬰幼兒的耳道殘餘容積比成人要小得多
（B）相同音量的聲音刺激傳入嬰幼兒耳朵中的聲壓級高於成人
（C）因為耳道殘餘容積的差異，嬰兒在所有頻率下的聽損都會顯得比成人更嚴重
（D）因為耳道大小的改變，會導致出生之後前幾年，測量的聽閾值可能會發生變化

ChatGPT

Question 16: Inappropriate Action for Complaint of Not Understanding Soft Speech in Quiet

When a hearing aid user complains about not being able to understand soft speech in a quiet environment, the goal is to make sure the hearing aid provides enough amplification, especially for the soft inputs, without causing discomfort or feedback. Here are the considerations for each option:

- **(A)** Asking the patient whether they can hear low-frequency or high-frequency cues can help determine which frequencies may need gain adjustments.
- **(B)** Using speech set at 45 dB SPL for assessment is appropriate because it represents a soft conversational level, helping to evaluate the hearing aid's performance for soft sounds.
- **(C)** Reducing the compression threshold and increasing the compression ratio for affected frequencies might make soft sounds louder but could also make normal conversational speech too loud and uncomfortable.
- **(D)** Increasing the gain for soft inputs, particularly if the user is struggling with soft sounds, is a common and appropriate strategy.

Considering the goal is to improve the understanding of soft speech, the least appropriate action would be **(C) Lowering the compression threshold and increasing the compression ratio for affected channels**, as this could potentially make normal conversational levels too loud and cause discomfort, rather than simply making soft sounds more audible.

Question 17: Incorrect Statement About Ear Canal Volume in Infants vs. Adults

When comparing the residual ear canal volume of infants to adults, the following statements can be considered:

- **(A)** It is true that infants have a much smaller ear canal volume compared to adults.
- **(B)** Because of the smaller volume, the same sound stimulus will produce a higher sound pressure level in an infant's ear compared to an adult's.
- **(C)** The statement that hearing loss would appear more severe in infants at all frequencies due to the difference in ear canal volume is incorrect. While the smaller volume can affect the measurement of hearing thresholds, especially at higher frequencies, it does not inherently make the hearing loss more severe.
- **(D)** The size of the ear canal changes as an infant grows, which can lead to changes in measured hearing thresholds over the first few years of life.

The incorrect statement is **(C) Because of the difference in residual ear canal volume, hearing loss at all frequencies will appear more severe in infants than in adults**. This is a misunderstanding; the smaller residual volume can affect the measurement, but it doesn't cause the hearing loss itself to be more severe. It is essential to account for the size of the ear canal when interpreting audiometric results, especially in infants and young children.

‹ 2 / 2 ›

You
Please answer the following multiple-choice question as a hearing care professional, providing reasoning and explanation: 18 聽能復健計畫（aural rehabilitation program）中涵蓋很多服務項目，下列何者不在其中?
（A）聽力保護 （B）溝通策略訓練 （C）理社會支持 （D）眩暈平衡運動
19 研究顯示可接受噪音值（acceptable noise level, ANL）與下列何者最可能相關?
（A）聽閾值 （B）最大響度不舒適音量（LDL）
（C）性別 （D） ABR 第五波波幅

ChatGPT

Question 18: Service Not Included in Aural Rehabilitation Programs

Aural rehabilitation programs are comprehensive plans designed to help individuals with hearing loss to improve their hearing and communication abilities. Here's how each option fits into such programs:

- **(A) Hearing Protection**: Aural rehabilitation programs often include education on hearing protection to prevent further hearing loss.
- **(B) Communication Strategies Training**: Training in communication strategies is a core component of aural rehabilitation, helping individuals to maximize their communication potential.
- **(C) Psychosocial Support**: Offering support for the psychological and social challenges associated with hearing loss is typically a part of aural rehabilitation.
- **(D) Vestibular Balance Exercises**: While balance issues can be related to the ear and hearing, vestibular balance exercises are usually a part of vestibular rehabilitation, not aural rehabilitation, unless the hearing loss is specifically related to a vestibular disorder.

Given this, the option that is least likely to be a part of a standard aural rehabilitation program is **(D) Vestibular Balance Exercises**, unless the patient's hearing loss is directly associated with a vestibular condition.

Question 19: Factor Most Likely Related to Acceptable Noise Level (ANL)

Acceptable Noise Level (ANL) is the amount of background noise that a person is willing to tolerate while listening to speech. Research has explored various factors that might relate to ANL. Here are the considerations for each option:

- **(A) Hearing Threshold**: While one might assume that hearing threshold levels could influence ANL, studies have not consistently shown a strong correlation between the two.
- **(B) Loudness Discomfort Level (LDL)**: There is evidence to suggest that there could be a relationship between ANL and LDL. People with a lower tolerance for loud sounds might also

have a lower tolerance for background noise.

- **(C) Gender**: There is no strong evidence to suggest that gender has a significant influence on ANL.
- **(D) ABR Wave V Amplitude**: Auditory Brainstem Response (ABR) Wave V Amplitude is an objective measure of auditory function, but it is not typically associated with subjective measures like ANL.

Considering the options, the most likely factor to be related to ANL is **(B) Loudness Discomfort Level (LDL)**. Individuals with lower LDLs might be less tolerant of background noise, which could be reflected in their ANL measurements. This is a topic of research, and conclusions might vary based on different studies.

You

Please answer the following multiple-choice question as a hearing care professional, providing reasoning and explanation: 20　當助聽器佩戴者表示遠處的說話聲比近處的說話聲更清楚，下列何種調整方式最能改善這個問題？
（A）提高最大輸出音量（MPO）　（B）選擇波峰消除法（peak clipping）
（C）將壓縮閾值調降　（D）運用更大的壓縮比
21　OSPL 90 的設定一般原則不包括下列何者？
（A）重要概念是避免不適感與失真
（B）目標是讓大聲拍手的聲音聽起來柔和又舒服
（C）設定太高時，聲音可能會傷害使用者的聽力
（D）設定太低時，可能影響使用者聆聽聲音的響度與範圍

ChatGPT

Question 20: Adjustment to Improve Clarity of Distant Speech for Hearing Aid Users

When a hearing aid wearer reports that distant speech sounds clearer than nearby speech, it suggests an issue with how the hearing aid processes sounds at different distances. Here's an analysis of each option:

- **(A) Increasing Maximum Output Volume (MPO)**: This adjustment increases the loudest sound the hearing aid can produce. While it might make all sounds louder, it doesn't necessarily address the issue of distant speech being clearer.
- **(B) Choosing Peak Clipping**: Peak clipping limits the volume of very loud sounds by clipping the peaks of the audio signal. This method can prevent discomfort from loud sounds but doesn't specifically address the clarity of speech at different distances.
- **(C) Lowering the Compression Threshold**: Lowering the compression threshold means that the hearing aid will start compressing (reducing gain for) sounds at a lower volume level. This might help with making nearer sounds clearer, especially if the issue is related to the hearing aid not

You

Please answer the following multiple-choice question as a hearing care professional, providing reasoning and explanation: 20　當助聽器佩戴者表示遠處的說話聲比近處的說話聲更清楚，下列何種調整方式最能改善這個問題？
（A）提高最大輸出音量（MPO）　（B）選擇波峰消除法（peak clipping）
（C）將壓縮閾值調降　（D）運用更大的壓縮比
21　OSPL 90 的設定一般原則不包括下列何者？
（A）重要概念是避免不適感與失真
（B）目標是讓大聲拍手的聲音聽起來柔和又舒服
（C）設定太高時，聲音可能會傷害使用者的聽力
（D）設定太低時，可能影響使用者聆聽聲音的響度與範圍

ChatGPT

Question 20: Improving Clarity for Distant Speech in Hearing Aid Users

The complaint that distant speech sounds clearer than nearby speech in a hearing aid user might be related to how the hearing aid processes and amplifies sounds at different distances. Let's examine each adjustment option:

- **(A) Increasing Maximum Output Volume (MPO)**: Increasing MPO will make the loudest sounds the hearing aid can produce louder, but it doesn't specifically address the issue of speech clarity at different distances.
- **(B) Choosing Peak Clipping**: Peak clipping limits the volume of very loud sounds. This method prevents discomfort from loud sounds but doesn't directly improve speech clarity, especially for distant sounds.
- **(C) Lowering the Compression Threshold**: Lowering the compression threshold means the hearing aid will start to compress (apply gain reduction) at a lower input level. This can make softer sounds, like distant speech, more audible without overly amplifying louder sounds, like nearby speech.
- **(D) Using a Greater Compression Ratio**: A higher compression ratio reduces the gain for loud sounds more than for soft sounds. This could potentially make distant sounds clearer but might not be the most direct solution.

The most suitable adjustment to specifically address the clarity of distant speech compared to nearby speech would be **(C) Lowering the Compression Threshold**. This adjustment would help in amplifying distant speech more effectively without over-amplifying nearby sounds.

Question 21: Principles Not Included in OSPL 90 Setting

OSPL 90 (Output Sound Pressure Level at 90 dB SPL Input) is a measure of the maximum output of a hearing aid. Let's analyze what principles are generally not included in setting OSPL 90:

- **(A) The key concept is to avoid discomfort and distortion**: This is a principle involved in setting OSPL 90, as it ensures the hearing aid's output does not become uncomfortably loud or distorted.
- **(B) The goal is to make the sound of loud claps soft and comfortable**: The purpose of OSPL 90 is to control the maximum output, but not necessarily to make specific sounds like claps soft and comfortable.
- **(C) Setting too high might damage the user's hearing**: This is true, as an excessively high OSPL 90 can lead to dangerously loud outputs.
- **(D) Setting too low might affect the user's ability to hear loudness and range**: A low OSPL 90 can limit the hearing aid's ability to provide adequate amplification for certain sounds, affecting the user's perception of loudness and sound range.

Considering these points, the principle that is least associated with the general purpose of OSPL 90 is **(B) The goal is to make the sound of loud claps soft and comfortable**. While managing overall loudness is a goal, the specific idea of making loud claps soft and comfortable doesn't directly relate to the primary function of OSPL 90, which is to control the maximum output to prevent discomfort and hearing damage.

You

Please answer the following multiple-choice question as a hearing care professional, providing reasoning and explanation: 22　有關平坦型置入增益之耦合器反應（coupler response for a flat insertion gain, CORFIG）之相關公式， 下列何者錯誤？
（A）　CORFIG = Coupler gain – Insertion gain
（B）　CORFIG = REUG – RECD – MLE
（C）　Insertion gain = Coupler gain – CORFIG + sound bore effect + vent effects
（D）　Coupler gain = Insertion gain –CORFIG + sound bore effect + vent effects
23　若真耳測試儀器的揚聲器持續播放 6000 Hz 純音，緩緩將探管放進正常成人耳道並持續量測耳道內聲壓，過程中確保探管沒有阻塞或其他異常情況，量測到音壓最小值的時候，探管尖端位於那個位置最合理？
（A）距離耳膜小於 5 mm　（B）距離耳膜大約 15 mm
（C）距離耳膜大於 25 mm　（D）量到的聲壓不受探管尖端位置影響

ChatGPT

Question 22: Incorrect Formula Related to CORFIG (Coupler Response for a Flat Insertion Gain)

CORFIG is used to estimate the response of a hearing aid in a coupler based on its performance in the ear (insertion gain). Let's evaluate each formula:

- **(A) CORFIG = Coupler gain – Insertion gain**: This formula seems to align with the basic concept of CORFIG, as CORFIG is intended to represent the difference between coupler gain and insertion gain.
- **(B) CORFIG = REUG (Real-Ear Unaided Gain) – RECD (Real-Ear to Coupler Difference) – MLE (Microphone Location Effect)**: This formula seems accurate as it takes into account the differences in gain measurements at the ear and in a coupler, adjusted for the location of the hearing aid's microphone.
- **(C) Insertion gain = Coupler gain – CORFIG + sound bore effect + vent effects**: This formula suggests that insertion gain is derived by adjusting coupler gain with CORFIG and accounting for sound bore and vent effects, which seems reasonable.
- **(D) Coupler gain = Insertion gain – CORFIG + sound bore effect + vent effects**: This formula incorrectly suggests that coupler gain is derived by subtracting CORFIG from insertion gain, which is not conceptually accurate.

The incorrect formula is **(D) Coupler gain = Insertion gain – CORFIG + sound bore effect + vent effects**. In reality, coupler gain would be more accurately determined by adding CORFIG to insertion gain, not subtracting it.

Question 23: Position of Probe Tube Tip for Minimum Sound Pressure Level at 6000 Hz

When conducting real-ear measurements, the position of the probe tube tip relative to the eardrum can significantly affect the measured sound pressure level, especially at higher frequencies like 6000 Hz. Let's consider the options:

- **(A) Less than 5 mm from the eardrum**: This position is very close to the eardrum and could result in high sound pressure levels due to the proximity to the source of sound transmission (the eardrum).
- **(B) Approximately 15 mm from the eardrum**: At this distance, the probe tube is likely still in the ear canal but not extremely close to the eardrum. This distance might be more likely to result in a minimum sound pressure level due to the standing wave effect, which is pronounced at higher frequencies.
- **(C) More than 25 mm from the eardrum**: This would place the probe tube outside or near the entrance of the ear canal, which is less likely to be the position for minimum sound pressure.
- **(D) The sound pressure measured is not affected by the position of the probe tube tip**: This is incorrect as the position of the probe tube tip, particularly in relation to the eardrum, significantly affects the measured sound pressure level.

The most reasonable position for the probe tube tip to measure the minimum sound pressure level at 6000 Hz is **(B) Approximately 15 mm from the eardrum**. This position is consistent with the location where a standing wave node (point of minimal sound pressure) is likely to occur for a frequency of 6000 Hz in an adult ear canal.

You

Please answer the following multiple-choice question as a hearing care professional, providing reasoning and explanation: 24　對於無法配合行為聽力檢查的嬰幼兒，以電生理檢查的反應閾值 dB nHL 來預估聽閾值，下列那些選配公式可直接套入 dB nHL 的結果計算出目標值？ ①NAL-NL1 ②NAL-NL2 ③DSL[i/o]
④DSLm[i/o]
（A）①③　（B）②③　（C）①④　（D）③④
25　Cochlear Nucleus 6 電池組有多種選擇，不包含下列何種形式？
（A）拋棄式 312 電池組　（B）標準充電電池組　（C）簡易充電電池組　（D）拋棄式 675 電池組

ChatGPT

Question 24: Fitting Formulas Compatible with dB nHL Results for Infants

In pediatric audiology, especially for infants who cannot participate in behavioral hearing tests, electrophysiological tests like ABR (Auditory Brainstem Response) are used, and the results are often in dB nHL (normalized hearing level). Some hearing aid fitting formulas can directly use dB nHL results to calculate target amplification. Let's consider the options:

- **① NAL-NL1 & ② NAL-NL2**: These National Acoustic Laboratories' nonlinear formulas are designed to optimize speech intelligibility and comfort. They typically use audiometric thresholds in dB HL, not dB nHL. While dB nHL can be converted to dB HL, these formulas do not directly use dB nHL.
- **③ DSL[i/o] & ④ DSLm[i/o]**: The Desired Sensation Level (DSL) method, both the input/output and the multistage input/output versions, is commonly used in pediatric fittings. The DSL method is known for its flexibility in accommodating various types of measurements, including dB nHL, making it suitable for use with electrophysiological test results.

Therefore, the fitting formulas that can directly incorporate dB nHL results to calculate target values are **③ DSL[i/o] and ④ DSLm[i/o]**. The answer is **(D) ③④**.

Question 25: Battery Options Not Included with Cochlear Nucleus 6

Cochlear implants like the Cochlear Nucleus 6 offer various power options to cater to different user needs and preferences. To determine which battery form is not included, let's consider the typical options:

- **(A) Disposable 312 Battery Pack**: The 312 zinc-air battery is a common size for hearing aids and some cochlear implant sound processors.
- **(B) Standard Rechargeable Battery Pack**: Rechargeable batteries are often available for modern cochlear implants, including standard size options.
- **(C) Compact Rechargeable Battery Pack**: Compact or smaller rechargeable batteries might also be an option for users who prefer a smaller device, though specific availability can vary.
- **(D) Disposable 675 Battery Pack**: The 675 battery is a larger battery commonly used in high-power hearing aids and some cochlear implants. However, it might not be a standard option for all cochlear implant models.

Given these considerations, while all these options could potentially be available, the least likely to be included with the Cochlear Nucleus 6, based on standard cochlear implant battery offerings, is **(D) Disposable 675 Battery Pack**. This is because the Cochlear Nucleus series typically uses 312 batteries or rechargeable options, and the 675 size is generally larger than what is typically used in such devices. However, for the most accurate and up-to-date information, it's advisable to refer to the specific product details provided by Cochlear.

You

Please answer the following multiple-choice question as a hearing care professional, providing reasoning and explanation: 26　個案為一位家醫科醫師，本身有輕度感音神經性聽力損失，下列何種助聽器外型最適合搭配聽診器使用？
（A）耳掛型助聽器（behind-the-ear, BTE）　（B）耳內型助聽器（in-the-ear, ITE）
（C）耳道型助聽器（in-the-canal, ITC）　（D）深耳道型助聽器（completely-in-the-canal, CIC）
27　關於助聽器之效益驗證，下列敘述何者正確？
（A）助聽器之聲場測試可驗證是否符合公式之目標值
（B）真耳測試驗證之測試音以純音或窄頻聲音為佳，可獨立測試每一頻率的增益
（C）驗證助聽器是否符合目標值，要設定在使用時的狀況，不應關閉噪音抑制和迴授音消除等功能
（D）利用林氏六音測試察覺和複誦，可以簡易驗證對不同頻率聲音的反應

ChatGPT

Question 26: Suitable Hearing Aid Style for a Family Physician with Mild Sensorineural Hearing Loss Using a Stethoscope

For a medical professional like a family physician who uses a stethoscope and has mild sensorineural hearing loss, the choice of hearing aid style is crucial for comfort and functionality. Let's consider each option:

- **(A) Behind-The-Ear (BTE)**: BTE aids sit behind the ear with a tube leading to an earmold in the ear canal. This style can interfere with the placement of a stethoscope around the ear area.
- **(B) In-The-Ear (ITE)**: ITE aids fill the outer portion of the ear canal and part of the outer ear. They are less obtrusive than BTE but might still interfere with a stethoscope.
- **(C) In-The-Canal (ITC)**: ITC aids are smaller than ITE aids and fit partly in the ear canal, offering less interference with external devices like stethoscopes.
- **(D) Completely-In-The-Canal (CIC)**: CIC aids are the smallest and fit entirely within the ear canal, providing the least interference with the use of a stethoscope. They are ideal for users who need to place instruments near their ears frequently.

The most suitable hearing aid style for a physician who regularly uses a stethoscope would be **(D) Completely-In-The-Canal (CIC)**. This style minimizes interference with the stethoscope's placement around the ear.

Question 27: Correct Statement About Hearing Aid Verification

Hearing aid verification involves ensuring that the hearing aid is functioning as intended and meeting the user's needs. Let's analyze the statements:

- **(A)** Sound field testing with a hearing aid can verify if it meets the target values set by a fitting formula. This is a correct use of sound field testing in hearing aid verification.
- **(B)** Real-ear measurement (REM) tests are best conducted with pure tones or narrowband noise to independently test gain at each frequency. This is a standard practice in hearing aid fitting and verification.
- **(C)** When verifying a hearing aid's performance, it should be set to the user's typical settings, including noise suppression and feedback cancellation functionalities. This is because turning these features off might not accurately reflect the user's everyday experience with the device.
- **(D)** The Ling Six Sound Test is a simple and effective way to check the hearing aid's response to different frequencies. It involves the user perceiving and repeating six sounds that span the speech spectrum.

Considering these points, the correct statement about hearing aid verification is **(C) To verify if the hearing aid meets target values, it should be set to the user's typical conditions, and features like noise suppression and feedback cancellation should not be turned off**. This approach ensures that the verification process accurately reflects the real-world use of the hearing aid.

You

Please answer the following multiple-choice question as a hearing care professional, providing reasoning and explanation: 28　評估助聽器的使用、問題和滿意度的問卷，下列敘述何者正確？
（A）　Satisfaction with Amplification in Daily scale（SADL）助聽器滿意度量表，包括評估正向和負面的影響、服務和花費以及自我形象
（B）助聽器使用和滿意度的評估可直接視為評估復健成效的唯一依據
（C）助聽器問題的評估與使用時間和滿意度都是相關的，但與效益不相關
（D）　Hearing Aid Review（HAR）問卷評估包括助聽器使用的時間、使用的問題和生活品質
29　聽覺皮質誘發電位（cortical auditory evoked potential, CAEP）可作為助聽器客觀效益評估的方法之一， 關於 CAEP 的敘述，下列何者正確？
（A）　CAEP 沒反應不表示個案聽不到該聲音
（B）可利用 CAEP 的波形來判別聽力閾值
（C）聽覺輔具在聽損嬰幼兒的介入時間一旦超過 3 歲，其 P1 潛時就完全無法回復至正常值
（D）聽神經病變（auditory neuropathy）的孩童不會呈現 CAEP 的反應

ChatGPT

Question 28: Correct Statement About Questionnaires Assessing Hearing Aid Use, Problems, and Satisfaction

Assessing hearing aid use, problems, and satisfaction often involves questionnaires that provide insights into various aspects of a user's experience. Let's evaluate the statements:

- **(A)** The Satisfaction with Amplification in Daily Life (SADL) questionnaire does indeed include assessments of positive and negative impacts, service and cost, and self-image. It's a comprehensive tool for evaluating user satisfaction with their hearing aids.
- **(B)** While user satisfaction and usage assessments are important, they should not be the sole basis for evaluating rehabilitation effectiveness. Objective measures and other subjective reports are also essential.
- **(C)** The assessment of hearing aid problems is indeed related to usage time and satisfaction. However, it can also be related to the benefits, as unresolved problems can affect the perceived benefit of the hearing aid.
- **(D)** The Hearing Aid Review (HAR) questionnaire does assess the time of use, problems with usage, and aspects of quality of life related to hearing aid use.

The correct statement is **(A) The Satisfaction with Amplification in Daily Life (SADL) questionnaire assesses both positive and negative impacts, service and cost, and self-image**. This reflects the comprehensive nature of SADL

You

Please answer the following multiple-choice question as a hearing care professional, providing reasoning and explanation: 28　評估助聽器的使用、問題和滿意度的問卷，下列敘述何者正確？
（A）　Satisfaction with Amplification in Daily scale（SADL）助聽器滿意度量表，包括評估正向和負面的影響、服務和花費以及自我形象
（B）助聽器使用和滿意度的評估可直接視為評估復健成效的唯一依據
（C）助聽器問題的評估與使用時間和滿意度都是相關的，但與效益不相關
（D）　Hearing Aid Review（HAR）問卷評估包括助聽器使用的時間、使用的問題和生活品質
29　聽覺皮質誘發電位（cortical auditory evoked potential, CAEP）可作為助聽器客觀效益評估的方法之一， 關於 CAEP 的敘述，下列何者正確？
（A）　CAEP 沒反應不表示個案聽不到該聲音
（B）可利用 CAEP 的波形來判別聽力閾值
（C）聽覺輔具在聽損嬰幼兒的介入時間一旦超過 3 歲，其 P1 潛時就完全無法回復至正常值
（D）聽神經病變（auditory neuropathy）的孩童不會呈現 CAEP 的反應

ChatGPT

Question 28: Correct Statement About Questionnaires for Assessing Hearing Aid Use, Problems, and Satisfaction

- **(A) Satisfaction with Amplification in Daily Life (SADL)**: This questionnaire indeed covers a range of aspects including the positive and negative impacts of hearing aid use, costs and services related to the hearing aids, and the impact on self-image. It is a comprehensive tool for assessing user satisfaction.
- **(B)** Assessing hearing aid use and satisfaction is important, but it should not be the sole criterion for evaluating the effectiveness of rehabilitation. Other factors such as objective hearing tests and the user's ability to function in various listening environments should also be considered.
- **(C)** The assessment of hearing aid problems is related to both usage time and satisfaction. It is also likely to be related to the perceived benefits since unresolved issues can negatively impact the user's experience and perceived benefit from the hearing aid.
- **(D) Hearing Aid Review (HAR)**: This questionnaire typically includes assessments of how long and in what situations the hearing aids are used, problems encountered, and the impact on quality of life.

The correct statement is **(A) The Satisfaction with Amplification in Daily Life (SADL) questionnaire assesses both positive and negative impacts, service and cost, and self-image**.

Question 29: Correct Statement About Cortical Auditory Evoked Potentials (CAEP)

- **(A)** Lack of a CAEP response does not necessarily mean that the individual cannot hear the sound. CAEPs measure the brain's electrical response to sound and can be affected by various factors, including attention and neural integrity.
- **(B)** While CAEPs provide valuable information about auditory processing in the brain, they are not typically used to determine hearing thresholds directly. Other tests are better suited for threshold determination.
- **(C)** The timing of intervention with hearing aids in children with hearing loss is crucial. However, the statement that the P1 latency of CAEPs cannot return to normal values if intervention occurs after age 3 is not entirely accurate. While early intervention is key, there can still be significant benefits and neural plasticity beyond this age.
- **(D)** In children with auditory neuropathy, CAEP responses can be absent or abnormal. However, this doesn't mean they are always absent. The response can vary depending on the degree and nature of the neuropathy.

The correct statement is **(A) A lack of CAEP response does not necessarily mean that the individual cannot hear the sound**. CAEPs are complex and can be influenced by many factors beyond the mere presence of sound.

You

Please answer the following multiple-choice question as a hearing care professional, providing reasoning and explanation: 30　有關助聽器佩戴效益評估，下列敘述何者最不適當？
（A）語音清晰度指標（speech intelligibility index）可以使用佩戴前的語音清晰度，來預測佩戴後的語音清晰度
（B）語音理解程度方面，通常在安靜環境中的佩戴效益比吵雜環境要高
（C）客觀測試與主觀認知的佩戴效益通常有著高度相關
（D）臨床測試通常較難準確預測助聽器在實際環境中的佩戴效益
31　下列何者不是直接評估聽覺失能改善程度的問卷？
（A）助聽器性能清單（Hearing Aid Performance Inventory, HAPI）
（B）助聽器使用者問卷（Hearing Aid User's Questionnaire, HAUQ）
（C）格拉斯哥助聽器益處簡介（Glasgow Hearing Aid Benefit Profile, GHABP）
（D）重要他人聽力障礙量表（Significant Other Scale for Hearing Disability, SOS-HEAR）

ChatGPT

Question 30: Least Appropriate Statement About Hearing Aid Benefit Assessment

- **(A)** The Speech Intelligibility Index (SII) can indeed be used to predict speech clarity improvements with hearing aid use. It is a measure that estimates how much of speech is audible.
- **(B)** It is generally true that hearing aids tend to provide more benefit in quiet environments than in noisy ones. This is because background noise can mask speech sounds and make it harder for the hearing aid to effectively amplify speech.
- **(C)** The correlation between objective tests and subjective perceptions of hearing aid benefit can vary. While some users may report subjective benefits that align with objective measurements, others may not due to personal preferences, lifestyle, or specific listening environments.
- **(D)** Clinical tests often have limitations in predicting real-world hearing aid performance. This is because real-world listening environments are more variable and complex than the controlled settings of a clinic.

Given these considerations, the least appropriate statement is **(C) There is usually a high correlation between objective testing and subjective perception of hearing aid benefit**. In reality, the correlation can vary significantly due to various individual factors.

Question 31: Questionnaire Not Directly Assessing Improvement in Hearing Disability

- **(A) Hearing Aid Performance Inventory (HAPI)**: This inventory assesses the performance of hearing aids in various listening situations, which is directly related to hearing disability improvement.
- **(B) Hearing Aid User's Questionnaire (HAUQ)**: This questionnaire is designed to gauge the user's experiences and satisfaction with their hearing aids, which relates to the improvement in hearing ability.
- **(C) Glasgow Hearing Aid Benefit Profile (GHABP)**: GHABP assesses the benefit received from hearing aids in various listening situations, which is a direct measure of hearing disability improvement.
- **(D) Significant Other Scale for Hearing Disability (SOS-HEAR)**: This scale is used to measure the perception of a hearing-impaired person's significant other regarding the hearing disability. While it provides valuable insights, it's an indirect measure of the person's actual hearing improvement as it relies on the observations of another person.

The option that is not a direct assessment of the improvement in hearing disability is **(D) Significant Other Scale for Hearing Disability (SOS-HEAR)**. This scale measures another person's perception of the hearing-impaired individual's disability, rather than directly assessing the individual's own hearing improvement.

You

Please answer the following multiple-choice question as a hearing care professional, providing reasoning and explanation: 32　下列那一族群在人工耳蝸術後較常會出現電流圖起伏變化的狀況？
（A）細菌性腦膜炎　（B）巨細胞病毒感染　（C）聽神經病變　（D）內耳畸形

33　有關人工耳蝸閾值，下列敘述何者正確？
（A）聽力師為所有廠牌的植入者調頻時，都一定要進行閾值（T）的測定
（B）所有人工耳蝸，不分廠牌、型號，對閾值的定義與臨床純音聽檢的定義相同（ > 50%正確率的最小值）
（C）測定閾值時的方式與純音聽檢相同，均以減 10 加 5 做為電流調整的步級
（D）人工耳蝸主要廠牌中，Advanced Bionics 公司的T 值定義與臨床純音聽檢所定義的閾值相同

ChatGPT

Question 32: Group More Likely to Experience Fluctuations in Electric Thresholds After Cochlear Implant Surgery

Each of the conditions mentioned can impact the outcomes of cochlear implant surgery, but they have different implications for post-surgical variability in electric thresholds:

- **(A) Bacterial Meningitis**: Post-meningitic patients, especially those who have experienced ossification (bone growth) in the cochlea as a complication of meningitis, can have fluctuating electric thresholds. The ossification can affect electrode placement and the cochlear implant's performance.
- **(B) Cytomegalovirus (CMV) Infection**: While CMV is a common cause of congenital sensorineural hearing loss, it doesn't specifically lead to fluctuating electric thresholds post-implantation.
- **(C) Auditory Neuropathy**: In auditory neuropathy, the transmission of sound from the inner ear to the brain is disrupted. While cochlear implants can be effective, auditory neuropathy doesn't typically cause fluctuating electric thresholds post-implantation.
- **(D) Inner Ear Malformations**: Patients with inner ear malformations can have variable outcomes with cochlear implants, but these malformations don't typically cause fluctuating electric thresholds post-implantation.

Given these considerations, the group most likely to experience fluctuating electric thresholds after cochlear implant surgery is **(A) individuals who have experienced bacterial meningitis**. The potential ossification in the cochlea can lead to changes in the electrical environment around the implant, causing fluctuations.

Question 33: Correct Statement About Cochlear Implant Thresholds

Cochlear implant thresholds (T-levels) are important for programming cochlear implants but can vary based on manufacturer and implant design:

- **(A)** While measuring T-levels is a standard practice across manufacturers, the exact procedure and importance of T-levels can vary depending on the specific implant and programming philosophy.
- **(B)** The definition of threshold levels (T-levels) in cochlear implants does not necessarily align with the definition used in clinical pure-tone audiometry. Cochlear implant T-levels refer to the minimum level of electrical stimulation that elicits an auditory sensation and can vary based on the implant design and programming approach.
- **(C)** The method of adjusting stimulation levels in cochlear implants (e.g., reducing by 10, increasing by 5) can be different from the method used in pure-tone audiometry. The steps and approach can vary between manufacturers and individual patients.
- **(D)** The definition of T-levels for Advanced Bionics or any other major cochlear implant manufacturer may not be the same as the definition of threshold in clinical pure-tone audiometry. Each manufacturer can have their own specific criteria and methods for determining T-levels.

Considering these points, the correct statement is **(B) The definition of thresholds in all cochlear implants, regardless of brand or model, is not the same as the definition used in clinical pure-tone audiometry (> 50% correct response rate at the minimum level)**. The concept of thresholds in cochlear implants is tailored to the specifics of electrical stimulation, which is different from acoustic stimulation in pure-tone audiometry.

You

Please answer the following multiple-choice question as a hearing care professional, providing reasoning and explanation: 34　下列何者不會影響人工耳蝸植入者對於聲音音量變化的響度感知？
（A）刺激速率（stimulation rate）　（B）無限壓縮（infinite compression）
（C）脈寬（pulse width）　（D）電流級（current unit/ current level）
35　有關人工耳蝸刺激速率，下列敘述何者錯誤？
（A）慢速的刺激速率僅能提供振幅包絡（amplitude envelope）信息，無法提供精細時間結構（fine temporal structure）信息，因此會影響噪音下的語音辨識
（B）慢速的刺激速率，會使得聽神經以高度同步的方式放電，並隨後同時進入不反應期，因此聽覺系統將無法跟上快速變化的聲音刺激
（C）越快的刺激速率越能幫助植入者提高聽覺表現，但大多數的植入者在使用超過 1000～2000 pps 的刺激速率時，聽覺表現卻不再提升
（D）根據研究，每個植入者達到最佳聆聽表現的刺激速率都不同，因此要為植入者選擇能提供最高總刺激速率（total stimulation rate）的產品

ChatGPT

Question 34: Factor Not Affecting Loudness Perception in Cochlear Implant Users

Let's analyze how each factor affects the loudness perception in cochlear implant users:

- **(A) Stimulation Rate**: The rate at which electrical pulses are delivered to the cochlea can impact the perception of sound, including its pitch and loudness.
- **(B) Infinite Compression**: In cochlear implants, compression refers to how changes in input sound levels are mapped to electrical stimulation levels. Infinite compression would imply that all sounds, regardless of their original intensity, are mapped to the same electrical level, affecting loudness perception.
- **(C) Pulse Width**: The width of the electrical pulse affects the amount of charge delivered during each stimulation. This can impact the loudness perception.
- **(D) Current Level**: The level of electrical current directly influences the perceived loudness of the sound in cochlear implants.

Given these considerations, the factor that would least affect loudness perception is **(B) Infinite Compression**. While compression settings can influence how dynamic changes in sound intensity are perceived, the concept of 'infinite compression', where all sounds are compressed to the same level, would essentially eliminate the perception of loudness variations.

Question 35: Incorrect Statement About Cochlear Implant Stimulation Rate

- **(A)** Slow stimulation rates indeed provide mainly amplitude envelope information and may not convey the fine temporal structure effectively. This limitation can affect speech recognition, especially in noise.
- **(B)** While slow stimulation rates can result in more synchronized neural firing, the description of auditory nerves entering a refractory period and being unable to follow rapidly changing sounds is an oversimplification and not entirely accurate in the context of cochlear implants.
- **(C)** Faster stimulation rates can improve auditory performance up to a point. However, increases beyond certain rates (like 1000-2000 pulses per second (pps)) often do not continue to improve performance, which is correct.
- **(D)** While it's true that the optimal stimulation rate varies among individuals, the goal is not necessarily to choose a product with the highest total stimulation rate. Rather, it's important to find the optimal rate for each individual, which might not be the highest possible rate.

The incorrect statement is **(D) According to research, each implant user has a different optimal stimulation rate, so the product providing the highest total stimulation rate should be chosen for the user**. The focus should be on individual optimization rather than merely selecting the highest available rate.

You

Please answer the following multiple-choice question as a hearing care professional, providing reasoning and explanation: 36　下列那些因素會影響人工耳蝸植入體的電極阻抗？ ①電極導絲 ②內耳淋巴液 ③電極周圍組織
④電解質 ⑤耳蝸骨化程度
（A）僅①②④　（B）僅①②③　（C）僅②③⑤　（D）①②③④⑤
37　下列何種編碼策略可採用 Bipolar 的電刺激模式，將電流限制在耳蝸內？
（A）　Advanced Combination Encoder（ACE）　（B）　Continuous Interleaved Sampling（CIS）
（C）　Spectral Peak（SPEAK）　（D）　High Definition CIS（HDCIS）

ChatGPT

Question 36: Factors Affecting Electrode Impedance in Cochlear Implants

Electrode impedance in cochlear implants can be influenced by several factors:

1. **Electrode Lead Wires (①)**: The physical and electrical properties of the electrode lead wires can affect impedance.
2. **Inner Ear Lymph Fluid (②)**: The electrical conductivity of the lymph fluid in the inner ear can impact impedance.
3. **Tissue Around the Electrode (③)**: The nature of the tissue surrounding the electrode, including any fibrous tissue growth or changes post-surgery, can affect impedance.
4. **Electrolytes (④)**: The concentration and type of electrolytes in the inner ear fluid can influence electrical conductivity and impedance.
5. **Degree of Cochlear Ossification (⑤)**: Ossification or bone growth in the cochlea, often as a result of conditions like meningitis, can change the cochlear environment and affect electrode impedance.

All these factors play a role in determining the electrode impedance in cochlear implants. Therefore, the answer is **(D) ①②③④⑤**, as all these factors can influence electrode impedance.

Question 37: Coding Strategy Using Bipolar Stimulation in Cochlear Implants

Bipolar stimulation in cochlear implants involves using closely spaced electrodes to limit the spread of electrical current within the cochlea. Let's consider the coding strategies:

- **(A) Advanced Combination Encoder (ACE)**: ACE uses a multi-channel strategy and can use various stimulation modes, including bipolar.
- **(B) Continuous Interleaved Sampling (CIS)**: CIS is a multi-channel strategy primarily using monopolar stimulation, but it can be adapted for bipolar stimulation in some implant systems.
- **(C) Spectral Peak (SPEAK)**: SPEAK was an earlier strategy that used a limited number of channels and could be programmed for bipolar stimulation.
- **(D) High Definition CIS (HDCIS)**: HDCIS is an advanced version of CIS, typically using monopolar stimulation, but it also can be adapted for bipolar stimulation.

Bipolar stimulation can be used in various strategies to limit the spread of the electrical current within the cochlea. Most modern cochlear implant systems and strategies, including ACE, CIS, and their advanced forms, can be programmed to use bipolar stimulation if needed. Therefore, each of the mentioned strategies **(A) ACE, (B) CIS, (C) SPEAK, and (D) HDCIS** can potentially employ bipolar stimulation, but the specific capability depends on the cochlear implant system and programming.

You

please give the most suitable answer for Q37

ChatGPT

For Question 37, regarding the coding strategy in cochlear implants that can utilize bipolar electrical stimulation, it's important to understand the characteristics of each mentioned strategy:

- **(A) Advanced Combination Encoder (ACE)**: This is a widely used strategy that primarily focuses on delivering rapid and non-sequential stimulation across multiple channels. While it predominantly uses monopolar stimulation, it can potentially be adapted to use bipolar stimulation depending on the cochlear implant system's capabilities.
- **(B) Continuous Interleaved Sampling (CIS)**: CIS is known for its sequential, non-overlapping stimulation of electrodes. It typically uses monopolar stimulation but can be adapted for bipolar stimulation in some cochlear implant systems.
- **(C) Spectral Peak (SPEAK)**: This earlier strategy focuses on a limited number of channels and primarily used monopolar stimulation. Like others, it could potentially be adapted for bipolar

primarily used monopolar stimulation. Like others, it could potentially be adapted for bipolar stimulation depending on the implant system.

- **(D) High Definition CIS (HDCIS)**: An advanced version of CIS, HDCIS also usually employs monopolar stimulation but can be programmed for bipolar stimulation in certain cochlear implant systems.

Given these characteristics, the most suitable answer is that each of these strategies **(A) ACE, (B) CIS, (C) SPEAK, and (D) HDCIS** can potentially employ bipolar stimulation mode, depending on the specific cochlear implant system and its programming capabilities. The choice of stimulation mode (bipolar or monopolar) is often determined by the clinical goals, the characteristics of the patient's hearing loss, and the features of the implant system.

You

Please answer the following multiple-choice question as a hearing care professional, providing reasoning and explanation: 38 關於人工耳蝸術前佩戴助聽器，下列敘述何者正確?
（A）一般建議術前必須佩戴助聽器至少 3 ~ 6 個月，是為了讓植入者養成佩戴聽覺輔具的良好習慣
（B）如果出生 3 個月內就確診為雙側極重度聽損，應直接等著植入人工耳蝸，不需要再買助聽器，以避免造成金錢浪費
（C）先天性聽損的嬰兒，術前佩戴助聽器，主要是為了幫助父母接受孩子有聽損的事實
（D）術前佩戴助聽器是為了確認植入者可否從非植入性的聽覺輔具得到有效的聽覺及語言辨識能力
39 在人工耳蝸術前評估中，透過 MRI 檢查無法確認下列何種病症?
（A） ossification （B） Mondini's dysplasia
（C） cochlear nerve deficiency （D） otosclerosis

ChatGPT

Question 38: Correct Statement About Pre-Cochlear Implant Hearing Aid Use

Let's evaluate each statement:

- **(A)** It's not universally required to wear a hearing aid for 3-6 months before cochlear implant surgery solely to develop a habit of wearing auditory devices. The decision to use a hearing aid pre-surgery depends on several factors, including the potential benefit to the user.
- **(B)** For infants diagnosed with profound bilateral hearing loss, the use of hearing aids before considering cochlear implantation is often recommended. This is to assess any residual hearing and potential benefit from hearing aids. It's not solely a matter of avoiding financial waste; it's about assessing all viable options for auditory stimulation.
- **(C)** While helping parents come to terms with their child's hearing loss is important, the primary purpose of fitting hearing aids before cochlear implantation in congenitally deaf infants is not solely for this psychological aspect. It's more focused on assessing auditory responses and potential benefits from amplification.
- **(D)** Pre-surgical use of hearing aids is indeed to confirm whether the individual can gain sufficient auditory and speech recognition abilities from non-implantable auditory devices. This assessment helps determine if a cochlear implant is the most suitable option.

The correct statement is **(D) Wearing hear[illegible] aids before surgery is to confirm if the implant candidate can achieve effective auditory and speech recognition abilities from non-implantable hearing aids**.

Question 39: Conditions Not Confirmed by MRI in Pre-Cochlear Implant Assessment

Magnetic Resonance Imaging (MRI) is used in pre-cochlear implant assessments to visualize the inner ear and related structures. Let's analyze its applicability:

- **(A) Ossification**: MRI can detect cochlear ossification, which is important for cochlear implant planning.
- **(B) Mondini's Dysplasia**: This congenital inner ear malformation can be diagnosed using MRI, as it visualizes the cochlear structure.
- **(C) Cochlear Nerve Deficiency**: MRI is critical in identifying cochlear nerve anomalies or deficiencies.
- **(D) Otosclerosis**: This condition primarily affects the bones of the middle ear, particularly the stapes. While MRI can provide information about soft tissues and nerve structures, it's not the primary imaging modality for diagnosing [illegible]sclerosis. CT scans are generally more effective for visualizing the bony changes associated with otosclerosis.

The condition that MRI is least likely to confirm in pre-cochlear implant assessments is **(D) Otosclerosis**. MRI is less effective for assessing bone-related conditions compared to CT scans, which are better suited for visualizing the ossicular chain and cochlear bone in otosclerosis.

You

Please answer the following multiple-choice question as a hearing care professional, providing reasoning and explanation: 40 人工耳蝸植入者若抱怨整體音量太小聲，下列何種調頻方式可以改善?
（A）降低 C levels （B）重新測量 T levels （C）調低音量設定 （D）調降靈敏度（sensitivity）

41 人工耳蝸術後復健強調實證操作（evidence-based practice, EBP）的重要，下列何者不屬於其執行的步驟?
（A）提出問題 （B）尋找證據 （C）評估結果 （D）訓練溝通

ChatGPT

Question 40: Adjusting Programming to Increase Overall Volume in Cochlear Implant Users

When a cochlear implant user complains that the overall volume is too low, the goal is to adjust the programming to enhance the loudness without causing discomfort or distortion. Let's analyze the options:

- **(A) Lowering C levels**: The "C level" or "comfort level" is the maximum electrical stimulation level that is comfortable for the user. Lowering this level would actually decrease the volume, which is not the desired outcome.
- **(B) Re-measuring T levels**: "T level" or "threshold level" is the minimum level of electrical stimulation at which sound is just audible. Re-measuring these levels might not directly address the issue of overall volume being too low, although it's important for baseline programming.
- **(C) Lowering the Volume Setting**: Lowering the volume setting would further decrease the loudness, which is opposite to what is needed in this situation.
- **(D) Adjusting Sensitivity**: Sensitivity in a cochlear implant refers to how the device picks up and processes environmental sounds. Loweri[illegible]ensitivity might make the implant less responsive to quieter sounds, which does not directly address the issue of overall volume.

The most appropriate action to increase the overall volume would be to **re-measure the T levels (B)** and possibly adjust the C levels (but by increasing them, not lowering). Reassessing the T levels might help in recalibrating the base level of sound perception, and adjusting the C levels upwards could allow for a higher comfortable volume range. However, it's critical to make these adjustments carefully to avoid discomfort or auditory damage.

Question 41: Step Not Included in Evidence-Based Practice (EBP) for Post-Cochlear Implant Rehabilitation

Evidence-based practice in post-cochlear implant rehabilitation involves several key steps:

- **(A) Formulating a Question**: This involves identifying a specific question or problem to address based on the patient's needs.
- **(B) Searching for Evidence**: This step involves looking for scientific research or clinical data that can help answer the question or solve the problem.
- **(C) Evaluating the Results**: After gatherin[illegible]idence, it's important to assess its quality, relevance, and applicability to the specific clinical situation.
- **(D) Communication Training**: While communication training is a crucial part of cochlear implant rehabilitation, it is not a direct step in the process of evidence-based practice. EBP focuses more on the methodology of integrating clinical expertise, patient values, and the best available evidence in the decision-making process.

Therefore, the step not directly involved in the execution of evidence-based practice is **(D) Communication Training**. While essential in rehabilitation, it is a component of the intervention, not a step in the EBP process itself.

You

Please answer the following multiple-choice question as a hearing care professional, providing reasoning and explanation: 42 語言促進技巧（facilitative language techniques）可以幫助幼童的語言發展，其中不包含下列何種?
（A）自我談話 （B）平行談話 （C）擴展及示範 （D）讀唇訓練
43 在人工耳蝸調頻（Mapping）的過程中，常見的電流刺激模式（stimulation mode），不包含下列何者?
（A） Monopolar（MP） （B） Fine structure processing（FSP）
（C） Bipolar（BP） （D） Bipolar+1（BP+1）

ChatGPT

Question 42: Facilitative Language Technique Not Included in Early Childhood Language Development

Facilitative language techniques are strategies used to enhance language development in children. Let's examine the options:

- **(A) Self-talk**: This involves the adult talking about what they are doing, thinking, or feeling. It's a way to model language use for the child.
- **(B) Parallel talk**: In parallel talk, the adult describes what the child is doing, seeing, or feeling. This helps the child connect words with actions or objects.
- **(C) Expansion and modeling**: This technique involves expanding on the child's speech to make it more grammatically correct or complex, and modeling correct language use.
- **(D) Lip-reading training**: While lip-readin[illegible]n be a useful skill, especially for children with hearing loss, it is not considered a facilitative language technique. Facilitative language techniques are more about verbal language development and interaction.

Therefore, the option that is not a facilitative language technique for early childhood language development is **(D) Lip-reading training**.

Question 43: Uncommon Stimulation Mode in Cochlear Implant Mapping

In cochlear implant mapping, different stimulation modes are used to deliver electrical signals to the auditory nerve. Let's review the options:

- **(A) Monopolar (MP)**: This is a common stimulation mode where one electrode serves as the active electrode, and a remote site (like a ground electrode) serves as the reference.
- **(B) Fine Structure Processing (FSP)**: FSP is a type of signal processing strategy used in cochlear implants to preserve the fine temporal structures of sound. It's not a stimulation mode but rather a processing strategy.
- **(C) Bipolar (BP)**: In this mode, the electrical current is confined between two adjacent electrodes within the cochlea.
- **(D) Bipolar+1 (BP+1)**: This is a variation of [illegible] bipolar mode where the current is confined between one active electrode and a neighboring electrode, which is one electrode removed from the active one.

Given these descriptions, the option that is not a stimulation mode in cochlear implant mapping is **(B) Fine Structure Processing (FSP)**. FSP is a signal processing strategy, not a mode of electrical stimulation.

You

Please answer the following multiple-choice question as a hearing care professional, providing reasoning and explanation: 44 選配跨傳式助聽器（CROS）時，個案抱怨噪音吵雜問題比起未佩戴時更差，下列何種方式可以解決此問題?
（A）實耳測量優耳並降低過度放大頻率的增益
（B）比較佩戴與未佩戴下語音辨識度說明效益
（C）優耳的助聽器應該啟動迴授抵銷（feedback cancellation）功能
（D）優耳使用開放式耳模避免閉塞效應
45 在殘響（reverberation）空間使用方向性麥克風時，下列何種情況最可能降低其效益?
（A）信號和反射背景噪音由同角度進入麥[illegible]
（B）說話者非常靠近助聽器使用者
（C）信號來自麥克風前方最靈敏的角度而背景噪音來自不同角度
（D）信號來自麥克風前方最靈敏的角度而背景噪音來自正後方

ChatGPT

Question 44: Addressing Noise Complaints in CROS Hearing Aid Users

When fitting a Contralateral Routing of Signal (CROS) hearing aid system, especially if the patient complains about increased noise issues compared to not wearing the device, consider the following options:

- **(A) Real-Ear Measurement on the Better Ear and Reduce Over-Amplified Frequencies' Gain**: Real-ear measurements can help in fine-tuning the gain settings of the hearing aid on the better ear. Reducing gain in frequencies where noise is problematic can help, but it might not address the specific issue of noise introduced by the CROS system itself.
- **(B) Compare Speech Recognition with and without the Device to Explain Benefits**: While this can help in understanding the benefits of the C[illegible]OS system, it may not directly solve the noise problem the patient is experiencing.
- **(C) Activate Feedback Cancellation on the Better Ear's Hearing Aid**: Feedback cancellation can improve the sound quality but may not specifically address the increased noise issue unless the noise is feedback-related.
- **(D) Use an Open-Fit Mold on the Better Ear to Prevent Occlusion Effect**: Using an open-fit mold can reduce the occlusion effect and might help with overall sound quality, including reducing the perception of noise. However, it doesn't directly address noise issues specific to the CROS system.

The most suitable approach to addressing the specific complaint of increased noise with a CROS system is **(A) Real-Ear Measurement on the Better Ear and Reduce Over-Amplified Frequencies' Gain**. This method directly targets the issue of noise perception and allows for more precise tuning of the hearing aid settings.

Question 45: Situation Likely to Reduce the Effectiveness of Directional Microphones in Reverberant Spaces

Directional microphones are used in hearir[illegible]ids to enhance speech understanding by focusing on sounds coming from in front of the user. However, their effectiveness can be reduced in certain situations, especially in reverberant spaces:

- **(A) Signal and Reflected Background Noise Entering the Microphone from the Same Angle**: In this scenario, the directional microphone may not effectively distinguish between the desired signal (like speech) and the noise, reducing its effectiveness.
- **(B) The Speaker is Very Close to the Hearing Aid User**: When the speaker is very close, the direct sound may be strong enough that reverberation has less impact, and the directional microphone can still be effective.
- **(C) The Signal Comes from the Most Sensitive Angle of the Microphone and Background Noise Comes from Different Angles**: This is the ideal situation for a directional microphone, where it can focus on the signal and reduce noise from other angles.
- **(D) The Signal Comes from the Most Sensitive Angle of the Microphone and Background Noise Comes from Directly Behind**: Directional microphones are typically less sensitive to sounds from directly behind, so this situation might not significantly reduce their effectiveness.

Given these considerations, the scenario m[illegible]t likely to reduce the effectiveness of directional microphones in reverberant spaces is **(A) S[illegible]nal and Reflected Background Noise Entering the Microphone from the Same Angle**. In this case, the directional microphone may struggle to distinguish between the speech signal and the noise, reducing its effectiveness in enhancing speech understanding.

You

Please answer the following multiple-choice question as a hearing care professional, providing reasoning and explanation: 46 有關骨導型助聽器，下列敘述何者最適當?
（A）將骨導振動器透過手術和頭骨直接連結的方式稱為經皮式耦合（percutaneous coupling）
（B）可以透過 6 c.c.耦合器測量骨導振動器的輸出音量
（C）較適合骨導閾值大於 50 dB HL 以上的患者
（D）與單耳相比，雙耳佩戴骨導型助聽器無法增加聲源定位能力
47 若感應線圈系統（induction loop system）之纜線設置圍繞呈正方形，且纜線圈數與電流保持固定不變， 則纜線圍繞區域正中央的電磁場強度 H 與纜線圍繞區域面積 a 之關係為何?
（A） H 與 a 呈正比 （B） H 與 a 呈反比 （C） H 的平方與 a 呈正比 （D） H 的平方與 a 呈反比

ChatGPT

Question 46: Most Appropriate Statement About Bone Conduction Hearing Aids

Let's analyze each option:

- **(A) Percutaneous Coupling**: This refers to a bone conduction system where the bone conductor (vibrator) is directly attached to the skull through surgical intervention. It's called percutaneous because it involves a small part that penetrates the skin to connect the external processor to the implant.
- **(B) Measuring Output with a 6 cc Coupler**: The 6 cc coupler is typically used for measuring output of air conduction hearing aids, not bone conduction devices. Bone conduction devices would require a different measurement approach, often involving a skull simulator.
- **(C) Suitable for Bone Conduction Thresholds Greater Than 50 dB HL**: Bone conduction hearing aids are generally suitable for individuals with conductive or mixed hearing loss, where the bone conduction threshold is better than 50 dB HL. They are not typically recommended for individuals with a bone conduction threshold greater than 50 dB HL, as this indicates a significant sensorineural component to their hearing loss.
- **(D) Binaural Versus Monaural Bone Cond[illegible]ion Devices**: Wearing two bone conduction devices (binaural fitting) can improve sound localization compared to wearing only one (monaural fitting). This is due to binaural summation and the ability to compare timing and loudness differences between ears.

The most appropriate statement is **(A) The method of connecting the bone conduction vibrator directly to the skull via surgery is known as percutaneous coupling**.

Question 47: Relationship Between Electromagnetic Field Strength and Area in an Induction Loop System

In an induction loop system, the electromagnetic field strength (H) within the loop is related to the loop's area and the current flowing through it. If the loop is square and the number of turns and current remain constant:

- **(A) H Proportional to a**: The field strength is not directly proportional to the area. The relationship involves the dimensions of the loop and the current.
- **(B) H Inversely Proportional to a**: The electromagnetic field strength inside a loop is more closely related to the loop's dimensions (length o[illegible] sides) rather than the area itself. The relationship is not a simple inverse proportionality to the area.
- **(C) H Squared Proportional to a**: The square of the field strength is not typically considered in relation to the area.
- **(D) H Squared Inversely Proportional to a**: Again, the square of the field strength is not the standard consideration in relation to the area.

The relationship between the electromagnetic field strength and the loop's area in an induction loop system is governed by Ampere's Law and the Biot-Savart Law, which state that the magnetic field strength in a loop is proportional to the current and inversely proportional to the distance from the wire. However, it's not a straightforward proportional or inverse relationship with the loop's area. The most accurate interpretation is that the field strength is affected by the loop's dimensions and the current, but it doesn't follow a direct or inverse proportionality with the area of the loop. Therefore, none of the given options accurately describe the relationship between the electromagnetic field strength and the loop area.

You

please give the most suitable answer for Q47

ChatGPT

For Question 47, regarding the relationship between the electromagnetic field strength H in the center of a square induction loop system and the area a of the loop, given that the number of turns and current remain constant:

- **(A) H proportional to a**: This suggests that as the area of the loop increases, the magnetic field strength also increases proportionally. However, this is not accurate. The magnetic field strength inside a loop is determined by factors like the current in the loop and the distance from the coil, not directly by the area of the loop.
- **(B) H inversely proportional to a**: This implies that as the area of the loop increases, the magnetic field strength decreases. According to the principles of electromagnetism, particularly Ampere's Law, the magnetic field strength in a loop is inversely proportional to the distance from the wire. For a square loop, as the area increases, the distance from the center to the sides of the loop also increases, which would theoretically reduce the field strength at the center. This option is more consistent with the principles of e[illegible]romagnetism.
- **(C) H^2 proportional to a** and **(D) H^2 inversely proportional to a**: These options consider the square of the magnetic field strength in relation to the area, which is not a standard approach in electromagnetic field calculations for a loop system.

Given these considerations, the most suitable answer is **(B) H is inversely proportional to a**. This is because the magnetic field strength in the center of a square loop is primarily influenced by the distance from the loop's wire, and as the area of the loop increases, the average distance from the wire to points within the loop also increases, generally leading to a decrease in field strength at the center.

You

Please answer the following multiple-choice question as a hearing care professional, providing reasoning and explanation: 48 人工耳蝸組成包含體外與體內兩部分，下列何者是屬於體外部分?
（A）電極序列 （B）聲音處理器 （C）蝸外參考電極 （D）接收線圈
49 依據美國聽語學會規範調頻系統使用的頸部線圈（neck loop）之評估方法為何?
（A）聲場評估 （B）透過連接 2 c.c.耦合器分析
（C）實耳測量 （D）語音辨識度測驗

ChatGPT

Question 48: Component of Cochlear Implant that is Part of the External (Extracorporeal) Unit

Cochlear implants consist of both internal (implanted) and external components. Let's analyze each option:

- **(A) Electrode Array**: This is part of the internal component of a cochlear implant. The electrode array is surgically inserted into the cochlea.
- **(B) Sound Processor**: This is part of the external component. The sound processor captures sound, processes it, and then sends it to the internal components via a transmitter.
- **(C) Extracochlear Reference Electrode**: The reference electrode is part of the internal component, usually placed under the skin during surgery.
- **(D) Receiving Coil**: The receiving coil is part of the external component, which receives signals from the sound processor and transmits them to the internal implant.

The correct answer is **(B) Sound Processor**, as it is the part of the cochlear implant that resides outside the body and is responsible for capturing and processing sound before sending it to the internal components.

Question 49: Evaluation Method for Neck Loop Used in FM Systems According to American Academy of Audiology Guidelines

Neck loops in FM (Frequency Modulation) systems are used with hearing aids to wirelessly receive audio signals. The American Academy of Audiology provides guidelines for evaluating these systems. Let's evaluate the options:

- **(A) Sound Field Assessment**: This involves evaluating the performance of the FM system in an open area or room. While useful, it might not specifically assess the neck loop's performance with the hearing aid.
- **(B) Analysis Using a 2 cc Coupler**: This method involves connecting the neck loop to a 2 cc coupler to measure its output. It is a standard way to assess the performance of hearing aids and could be used for FM systems as well.
- **(C) Real-Ear Measurement**: Real-ear mea[illegible]ements assess how a hearing aid performs in the user's ear. This method is more focused on the hearing aid's performance rather than the neck loop of the FM system.
- **(D) Speech Recognition Testing**: This test evaluates how well a person can understand speech using the FM system. While it can assess the overall effectiveness of the system, it might not specifically evaluate the neck loop's performance.

Considering these options, the most suitable method for evaluating a neck loop in FM systems as per the American Academy of Audiology's guidelines is **(B) Analysis using a 2 cc coupler**. This method provides a standardized way to measure the output of the neck loop and how it interacts with the hearing aid.

You

Please answer the following multiple-choice question as a hearing care professional, providing reasoning and explanation: 50 跳頻展頻（frequency-hopping spread spectrum, FHSS）是一種調變（modulation）技術，下列何種遠端麥克風系統的訊號傳輸方式即採此技術?
（A）調幅（amplitude modulation, AM） （B）調頻（frequency modulation, FM）
（C）磁感應（magnetic induction） （D）[illegible]牙（bluetooth）

ChatGPT

You

Please answer the following multiple-choice question as a hearing care professional, providing reasoning and explanation: 50 跳頻展頻（frequency-hopping spread spectrum, FHSS）是一種調變（modulation）技術，下列何種遠端麥克風系統的訊號傳輸方式即採此技術?
（A）調幅（amplitude modulation, AM） （B）調頻（frequency modulation, FM）
（C）磁感應（magnetic induction） （D）藍牙（bluetooth）

ChatGPT

Frequency-Hopping Spread Spectrum (FHSS) is a modulation technique where the signal is broadcast over a seemingly random series of radio frequencies, hopping from frequency to frequency at fixed intervals. Let's analyze the options in the context of remote microphone systems:

- **(A) Amplitude Modulation (AM)**: AM involves modulating the amplitude of the carrier signal in proportion to the waveform of the sound being transmitted. It doesn't involve frequency hopping.
- **(B) Frequency Modulation (FM)**: FM systems modulate the frequency of the carrier wave to transmit sound. While FM systems are commonly used in hearing aid technologies, standard FM systems do not typically use frequency hopping as part of their signal transmission.
- **(C) Magnetic Induction**: This method uses electromagnetic fields to transmit audio signals. It's different from FHSS and doesn't involve changing frequencies for signal transmission.
- **(D) Bluetooth**: Bluetooth technology often uses FHSS as part of its standard protocol. In Bluetooth communication, the devices communicate over a wide band by rapidly switching among different frequencies to minimize interference and eavesdropping, making it a robust method for wireless audio transmission.

Given these explanations, the remote microphone system that uses Frequency-Hopping Spread Spectrum (FHSS) as its signal transmission technique is **(D) Bluetooth**. This technology is widely used in various devices, including hearing aids and assistive listening devices, for its secure and efficient wireless communication capabilities.

Message ChatGPT...

ChatGPT can make mistakes. Consider checking important information.
