# Supplementary material for: Exploring the Performance of ChatGPT-4 in the Taiwan Audiologist Qualification Examination: Preliminary Observational Study Highlighting the Potential of AI Chatbots in Hearing Care
Source: JMIR Med Educ. 2024 Apr 26;10:e55595. doi: 10.2196/55595 (PMC11067446; doi:10.2196/55595)
Supplement: Multimedia Appendix 6 [file mededu-v10-e55595-s006.pdf]

You

Please answer the following multiple-choice question as a hearing care professional, providing reasoning and explanation: 1在聽能復健的諮商歷程，引導個案提出明確的目標，且為其分析目標達成方式，讓個案接受問題及承擔挑戰，此屬於下列那一個步驟?
（A）幫助個案說明及釐清溝通問題 （B）幫助個案對自己的聽力問題負起責任
（C）幫助個案了解聽損對個體社會層面的影響 （D）幫助個案擬定復健計畫

ChatGPT

在聽能復健的諮商歷程中，引導個案提出明確的目標，並為其分析目標達成方式，這涉及到讓個案接受問題並承擔挑戰。這通常是一個多步驟的過程，其中包括了解個案的聽力問題、認識到這些問題如何影響他們的日常生活，以及制定一個具體的復健計劃。

結合選項：

- （A）幫助個案說明及釐清溝通問題：這個過程主要關注於識別和理解溝通障礙，但不一定直接涉及到設定具體的復健目標。
- （B）幫助個案對自己的聽力問題負起責任：這可能是整個過程的一部分，但是它更多的是關於心理和情感的接受，而不是實際的復健計劃[illegible]。
- （C）幫助個案了解聽損對個體社會層面的影響：這有助於個案理解他們的情況，但這本身並不涉及具體目標的設定。
- （D）幫助個案擬定復健計畫：這個選項最接近於描述中的活動。它涉及到與個案合作來確定目標，分析如何達到這些目標，並制定具體的行動計劃。

根據這些考慮，最合適的答案是（D）幫助個案擬定復健計畫。这个步骤不仅涉及到目标的设定，还包括了如何达成这些目标的具体分析，这是成功的聽能復健过程的关键部分。

You

please give me the answer in english

ChatGPT

In the context of audiological rehabilitation counseling, guiding a client to set clear goals and analyzing ways to achieve these goals, which involves accepting the problem and embracing challenges, relates to one of the steps in the process. Considering the provided options:

- (A) Helping the client explain and clarify communication issues: This step primarily focuses on identifying and understanding communication barriers but doesn't necessarily directly involve setting specific rehabilitation goals.
- (B) Helping the client take responsibility for their hearing problems: This might be part of the overall process, but it's more about emotional and psychological acceptance rather than the actual planning of rehabilitation.
- (C) Helping the client understand the social impact of hearing loss: This helps the client comprehend their situation, but it doesn't involve setting specific goals.
- (D) Helping the client formulate a rehabilitation plan: This option is the closest to the activities described. It involves collaborating with the client to determine goals, analyzing how to achieve these goals, and developing a specific action plan.

Based on these considerations, the most appropriate answer is (D) Helping the client formulate a rehabilitation plan. This step not only involves setting goals but also includes a detailed analysis of how to achieve these goals, which is a key part of a successful audiological rehabilitation process.

You

Please answer the following multiple-choice question as a hearing care professional, providing reasoning and explanation: 2 根據 Goldstein 與 Stephens（1981）、Stephens 與Kramer（2010）等人對於患者聽覺復健態度的研究，下列敘述何者正確？ ①類型I、II對復健的態度正向，人數比例較多 ②類型III態度負向，但仍有配合意願，必須修正態度，才能進行一般程序的聽能復健 ③類型IV對助聽器與復健過程皆排斥，必須修正態度，才能進行一般程序的聽能復健
（A）僅①② （B）僅①③ （C）僅②③ （D）①②③
3 下列那一個測驗是運用高預測與低預測句子，來評估成人聆聽者的言語知覺能力？
（A） CID Everyday Sentences Test （B） Speech Perception in Noise（SPIN）
（C） Hearing in Noise Test（HINT） （D） Quick Speech in Noise（QuickSIN）

ChatGPT

Let's address each question separately:

Question 2:
The question pertains to Goldstein and Stephens (1981) and Stephens and Kramer (2010) research on patients' attitudes towards auditory rehabilitation. The statements are about different types of attitudes (Type I, II, III, IV) and their impact [illegible]ehabilitation.

- Type I and II individuals have a positive attitude towards rehabilitation and are more numerous.
- Type III individuals have a negative attitude but are still willing to cooperate. Their attitude needs to be corrected for standard auditory rehabilitation to proceed.
- Type IV individuals reject both hearing aids and the rehabilitation process and need an attitude adjustment before standard rehabilitation can proceed.

The correct answer depends on the accuracy of these types and their descriptions as per the research mentioned. Generally, in audiological rehabilitation, patient attitude plays a crucial role. Those with positive attitudes (Type I and II) are more likely to engage successfully in rehabilitation. Type III individuals, though negative, show willingness to cooperate, indicating that attitude modification could lead to successful rehabilitation. Type IV individuals are the most challenging due to their rejection of both aids and the process, requiring significant effort in attitude modification.

Given this understanding, the correct answer would be (D) ①②③ if all these type descriptions align with the research by Goldstein and Stephens and Stephens and Kramer.

Question 3:
This question asks about a test that uses high-predictability and low-predictability sentences to assess adult listeners' speech perception abilities.

- (A) CID Everyday Sentences Test: This test uses everyday sentences but is not specifically known for high and low predictability sentences.
- (B) Speech Perception in Noise (SPIN): SPIN test is known for using high and low predictability sentences in noise, making it suitable for evaluating speech perception abilities in challenging listening environments.
- (C) Hearing in Noise Test (HINT): This test measures the ability to hear speech in noise but doesn't specifically focus on predictability of sentences.
- (D) Quick Speech in Noise (QuickSIN): This test evaluates the ability to hear in noisy environments but, like HINT, doesn't focus on sentence predictability.

Based on this, the correct answer is (B) Speech Perception in Noise (SPIN), as it specifically uses high and low predictability sentences to assess speech perception abilities.

You

Please answer the following multiple-choice question as a hearing care professional, providing reasoning and explanation: 4 成人聽損者的聽能管理，諮詢時會給予聽損者於日常溝通活動時的建議，針對 CLEAR 策略的敘述， 下列何者錯誤?
（A） Control：鼓勵聽損成人能夠練習自我監控適當的溝通情境和狀態
（B） Lipread：對話時鼓勵讀唇和善用視覺線索
（C） Expectations：設定合理期待、避免溝通的阻礙或尋求替代方案
（D） Repair：展現耐心，修補與對話者之間的關係，改善溝通氛圍
5 根據 Schow（2001）的成人聽能復健管理模式（CARE），聽力師向聽損者解釋評估結果，提供以證據為本的治療選項，並共同設定復健目標，這是屬於下列那一個過程?
（A）諮商 / 心理社會（counseling/psychosocial）
（B）可聽度 / 損傷管理（audibility/impairment management）
（C）溝通修補活動（remediate communication activity）
（D）環境協調 / 參與改善（environmental coordination/participation improvement）

ChatGPT

Let's analyze each question separately:

Question 4:
This question asks about the CLEAR strategy for communication advice given to adults with hearing loss. Let's evaluate each option:

- (A) Control: Encourages self-monitoring of appropriate communication situations and states. This is in line with the CLEAR strategy, where 'Control' involves managing the listening environment and situations.
- (B) Lipread: Encourages lip-reading and the use of visual cues during conversations. This is accurate as 'Lipread' in CLEAR stands for using visual cues to aid in understanding speech.
- (C) Expectations: Setting realistic expectations, avoiding communication barriers, or seeking alternatives. This is also correct. 'Expectations' involve having realistic goals and strategies for communication.
- (D) Repair: Demonstrating patience, repairing the relationship with the speaker, and improving the communication atmosphere. This option is slightly off. In the CLEAR strategy, 'Repair' refers to strategies for fixing communication breakdowns, not necessarily about repairing relationships. It's more about clarifying misunderstandings or asking for repetitions.

Given this analysis, the incorrect statement regarding the CLEAR strategy is (D) Repair.

Question 5:
This question is about Schow's (2001) CARE model for adult auditory rehabilitation. The task involves explaining assessment results, providing evidence-based treatment options, and setting rehabilitation goals with the client. Let's review each option:

- (A) Counseling/Psychosocial: This involves addressing the psychological and social aspects of hearing loss, which includes counseling and support.
- (B) Audibility/Impairment Management: This would involve managing the hearing loss itself, possibly through hearing aids or other technological solutions.
- (C) Remediate Communication Activity: This involves working on specific strategies to improve communication skills.
- (D) Environmental Coordination/Participation Improvement: This is about modifying the environment or using strategies to improve participation in various settings.

The process of explaining assessment results, providing treatment options, and setting goals aligns most closely with (A) Counseling/Ps[illegible]osocial. This aspect of the CARE model focuses on the holistic management of hearing loss, including understanding the condition, exploring treatment options, and goal setting, which are essential components of counseling and psychosocial support.

You

Please answer the following multiple-choice question as a hearing care professional, providing reasoning and explanation: 6 根據 Owens 等人的研究，對於聽損者在語音知覺上的困難，下列敘述何者最不適宜?
（A）子音的音素知覺最常見的是構音位置
（B）音素 /ʃ/ 和 / ʧ / 的語音知覺非常依賴於 1000Hz 到 2000 Hz 之間的靈敏度
（C）除了重度到極重度聽損者，大多數聽損者在母音感知方面的困難度較少
（D）音素位於詞首時，知覺錯誤的頻率較音素位於詞尾時更高
7 在上聽語課程時，小乖練習聽「老虎進了山洞」這句話，如果聽成「老鼠進了樹洞」，最可能受到那個聽覺線索因素的影響?
（A）節律線索 （B）上下文訊息 （C）毗鄰詞彙原則 （D）雙字詞構音

ChatGPT

Let's analyze each question separately:

Question 6:
This question asks about the least appropriate statement regarding speech perception difficulties in individuals with hearing loss, based on research by Owens and others. Let's evaluate each option:

- (A) The most common issue in the perception of consonants is the place of articulation. This is typically true as consonants differ mainly in their place of articulation, and individuals with hearing loss often struggle with this aspect.
- (B) The perception of the phonemes /ʃ/ and /ʧ/ heavily relies on sensitivity in the 1000Hz to 2000Hz range. This is accurate as these fricative sounds have their energy primarily in the higher frequency range, and hearing loss in this range can affect their perception.
- (C) Except for those with severe to profound hearing loss, most individuals with hearing loss have fewer difficulties in vowel perception. This is generally true because vowels are usually louder and have lower frequencies, which are often less affected by hearing loss.
- (D) Phonemes at the beginning of words [illegible]ore frequently perceived incorrectly than those at the end. This statement is not typically supported by research. The position of phonemes in words (initial or final) does not inherently make them more difficult to perceive for individuals with hearing loss.

Therefore, the least appropriate statement is (D) Phonemes at the beginning of words are more frequently perceived incorrectly than those at the end.

Question 7:
This question is about auditory cues influencing speech perception, illustrated by the misperception of the sentence "老虎進了山洞" (The tiger entered the cave) as "老鼠進了樹洞" (The mouse entered the tree hole). Let's review each option:

- (A) Rhythm cues: This refers to the pattern of stresses and pauses in speech, which might not be the primary factor in this case.
- (B) Contextual information: This involves using the surrounding context to understand speech. Mishearing "tiger" as "mouse" and "cave" as "tree hole" could be influenced by a lack of contextual understanding.
- (C) The principle of neighboring words: Th[illegible] refers to the influence of adjacent words on the perception of a particular word, which might not be directly relevant in this case.
- (D) Articulation of compound words: This involves how compound words are pronounced, which might not be the key factor here.

Given the misheard words, the most likely influencing factor is (B) Contextual information, as the misunderstanding could stem from a difficulty in using context to accurately perceive the words.

You

Please answer the following multiple-choice question as a hearing care professional, providing reasoning and explanation: 8 王先生為後天致聾（acquired deafened）的成人，佩戴助聽器後接受 Quick SIN 測驗，王先生在這項測驗最可能的結果為何?
（A）與先天性感音型聽損程度相似的成人測驗表現相近
（B）噪音下語音聽辨有困難
（C）安靜情境下的語音聽辨接近正常
（D）一週後接受相同測驗，第二次測驗得分有顯著進步
9 有關聽損者會話風格、溝通行為或溝通策略的敘述，下列何者正確?
（A）在會話溝通時採取逃避、假裝理解的[illegible]，屬於被動會話風格（passive conversational style）
（B）會話當中有意識地、習慣性地主導話題的進行，屬於互動型（interactive）溝通行為
（C）在溝通發生訊息中斷時，聆聽者請說話者換個方式再說一次，屬於非特定修復策略（nonspecific repair strategy）
（D）會談之前，先行準備對話內容與問題，屬於建設性策略（constructive strategy）

ChatGPT

Let's address each question one by one:

Question 8:
This question pertains to the expected results of Mr. Wang, an adult with acquired deafness, who uses hearing aids and takes the QuickSIN test. The QuickSIN (Quick Speech in Noise) test evaluates an individual's ability to understand speech in noisy environments. Let's evaluate each option:

- (A) Similar performance to adults with congenital sensorineural hearing loss: Acquired deafness and congenital hearing loss might present [illegible]ifferently in terms of speech-in-noise understanding, depending on various factors like age of o[illegible]et, duration of deafness, and experience with hearing aids.
- (B) Difficulty in speech recognition in noise: This is a likely outcome, as individuals with acquired deafness often struggle with speech perception in noisy environments, even with hearing aids.
- (C) Near-normal speech recognition in quiet: Individuals with acquired deafness, especially when using hearing aids, may have relatively better speech recognition in quiet environments.
- (D) Significant improvement in the same test after one week: While some learning effect is possible, a significant improvement within a week is unlikely unless there are specific interventions or training between the tests.

Based on this, the most likely result for Mr. Wang in the QuickSIN test is (B) Difficulty in speech recognition in noise, as this is a common challenge for individuals with acquired deafness, even when using hearing aids.

Question 9:
This question is about the conversational styles, communication behaviors, and strategies of individuals with hearing loss. Let's analyze each option:

- (A) Adopting avoidance or pretending to understand strategies in conversation is a passive conversational style. This is correct. A passive style often involves avoiding difficult listening situations or pretending to understand to avoid embarrassment or inconvenience.
- (B) Deliberately and habitually dominating the conversation topic is an interactive communication behavior. This is not typically defined as interactive. Interactive behavior usually involves active participation and exchange in the conversation rather than dominating it.
- (C) Asking the speaker to say something in a different way when there is a communication breakdown is a nonspecific repair strategy. This is not accurate. This would be considered a specific repair strategy as it involves a specific request to enhance understanding.
- (D) Preparing conversation content and questions before a discussion is a constructive strategy. This is correct. Constructive strategies involve planning and controlling the listening environment to facilitate better communication.

Therefore, the correct statements are (A) Passive conversational style involving avoidance and pretend understanding, and (D) Constructive strategy involving preparing conversation content and questions in advance.

You

please give the most suitable answer for Q9

ChatGPT

For Question 9, regarding the conversational styles, communication behaviors, and strategies of individuals with hearing loss:

- (A) Adopting avoidance or pretending to understand strategies in conversation is a passive conversational style. This is correct. In a passive conversational style, individuals might not actively seek clarification and may pretend to understand to avoid the difficulties of communication.
- (B) Deliberately and habitually dominating the conversation topic is an interactive communication behavior. This description is more akin to a controlling or aggressive style rather than an interactive one. Interactive styles involve back-and-forth communication where both parties actively participate.
- (C) Asking the speaker to say something [illegible] different way when there is a communication breakdown is a nonspecific repair strategy. This statement is incorrect. Such a strategy is actually specific, as it involves a direct request for clarification or alteration in the communication method.
- (D) Preparing conversation content and questions before a discussion is a constructive strategy. This is correct. A constructive strategy involves planning and controlling the conversation to mitigate the effects of hearing loss.

Given these descriptions, the most accurate and suitable answer is (D) Preparing conversation content and questions before a discussion is a constructive strategy. This reflects a proactive approach to managing communication challenges associated with hearing loss.

You

Please answer the following multiple-choice question as a hearing care professional, providing reasoning and explanation: 10 下列敘述何者屬於聽損者的接收性修補策略（receptive repair strategies）？ ①聽損者疑惑地向說者表示：「我沒有聽清楚，你說什麼？」 ②聽損者請說者簡化訊息 ③說者為聽損者放慢語速、再重複一次 ④聽損者向說者說：「你可以再說一次嗎？」
（A）僅①④ （B）僅③ （C）僅①②④ （D）①②③④
11 你的個案跟你表示，他經常會聽錯話，[illegible]覺得很尷尬，並舉出最近發生的事：孫子幫忙油漆客廳時說：「爺爺，你幫我拿一些報紙來」，個案驚訝的看著孫子說：「去拿一些包子？我們不是才剛吃過中餐嗎？」針對這個特定例子，你會建議個案在回應孫子前，使用那一個溝通策略?
（A）預期策略（anticipatory strategy） （B）適應策略（adaptive strategy）
（C）建設性策略（constructive strategy） （D）指導策略（instructional strategy）

ChatGPT

Question 10:
Receptive repair strategies are techniques used by individuals with hearing loss to better understand what is being said. Let's evaluate each option:

- ① The listener expressing confusion and asking, "I didn't catch that, what did you say?" This is a receptive repair strategy, as the listener is actively seeking clarification.
- ② The listener asking the speaker to simplify the message. This also falls under receptive repair strategies, as it's a request for clearer communication.
- ③ The speaker slowing down and repeating for the listener. While this aids comprehension, it's an action taken by the speaker, not a strategy employed by the listener, so it's not a receptive repair strategy in the strict sense.
- ④ The listener asking the speaker to repeat something. This is a classic example of a receptive repair strategy.

Given these considerations, the options that fall under receptive repair strategies are ①, ②, and ④. Therefore, the correct answer is (C) Only ①, ②, and ④.

Question 11:
In the given scenario, the client mishears a request for "newspaper" as a request for "steamed buns" ("包子"). The question asks which communication strategy would be best for the client to use before responding to the grandson. Let's evaluate each option:

- (A) Anticipatory Strategy: Involves preparing for potential communication challenges in advance. It might not be as effective in this specific case since the misunderstanding is already occurring in real-time.
- (B) Adaptive Strategy: Involves adapting to the communication situation as it unfolds, which could be helpful but doesn't provide a direct approach to address the specific misunderstanding.
- (C) Constructive Strategy: Involves modifying the environment or context to enhance communication. This could include ensur[illegible]better viewing of the speaker's face for lip-reading, but it might not directly address the specific type of misunderstanding in this scenario.
- (D) Instructional Strategy: Involves directing the speaker on how to make communication easier. This could include asking for clarification or repetition, which would be directly beneficial in this scenario where there's a clear misunderstanding.

The most appropriate strategy for this specific scenario is (D) Instructional Strategy. It directly addresses the issue of mishearing by guiding the speaker (the grandson) on how to communicate more effectively, such as repeating or rephrasing the request more clearly.

You

Please answer the following multiple-choice question as a hearing care professional, providing reasoning and explanation: 12 爸爸、媽媽和孩子一起在玩扮家家酒的遊戲，角色分配為：媽媽煮飯、爸爸去買菜、小朋友當小幫手準備食材。當爸爸假裝出門要去大賣場買菜時，小孩說：「爸爸走了！」，此時媽媽說：「爸爸去賣場了嗎？」，媽媽使用了那種語言促進技巧?
（A）語言映射 （B）自我談話 （C）重構 （D）擴展
13 小美：「我要一杯無糖去冰可樂」，小明：「蛤？」；小美：「我要一杯無糖去冰可樂」，小明：「你是說，你要一杯無糖去冰可樂嗎?[illegible] 小美：「對啊」。以上對話中，何者為小明第二次使用的修補策略（repair strategies）？ ①非特定性溝通修補策略 ②表達性修補策略 ③接收性修補策略 ④重複訊息 ⑤確認內容
（A）②④⑤ （B）③④⑤ （C）僅①④ （D）僅③⑤

ChatGPT

Question 12:
In this scenario, a family is playing a pretend game where the roles are a cooking mom, a grocery-shopping dad, and a child helping prepare food. When the child says, "Dad has left!" and the mom responds with, "Has Dad gone to the store?" she is employing a specific language facilitation technique. Let's review the options:

- (A) Language Mapping: This involves narrating what the child is doing or experiencing. This is not quite what the mother is doing in this scenario.
- (B) Self-talk: This involves the adult talking about what they themselves are doing. Again, this is not what is occurring in the scenario.
- (C) Recasting: This is when the adult repeats the child's sentence but in a modified or corrected form. The mother's response could be se[illegible] a form of recasting, as she is taking the child's statement and turning it into a question to encourage further language use.
- (D) Expansion: This involves taking a child's short or incomplete phrase and expanding it into a more complex sentence. The mother's response does not exactly expand on the child's statement; rather, it alters its form.

Based on this, the most appropriate technique the mother is using is (C) Recasting. She is taking the child's statement and modifying it into a question, which is a form of recasting that encourages further language development.

Question 13:
In this dialogue, Xiao Mei says, "I want a sugar-free, ice-free Coke," and Xiao Ming initially responds with "Huh?" Then Xiao Mei repeats her request, and Xiao Ming confirms by saying, "You mean, you want a sugar-free, ice-free Coke?" Let's evaluate the repair strategies used by Xiao Ming in his second response:

- ① Nonspecific Communication Repair Strategy: This involves a general request for clarification without specifics. Xiao Ming's response is more specific than this.
- ② Expressive Repair Strategy: This involve[illegible]he speaker reformulating their own message, which is not what Xiao Ming is doing.
- ③ Receptive Repair Strategy: This is used by the listener to understand the message better. Xiao Ming's response involves confirming the content, which is a form of receptive repair.
- ④ Repetition of the Message: Xiao Ming is not simply repeating the message; he is seeking confirmation.
- ⑤ Confirming Content: Xiao Ming is confirming the content of Xiao Mei's message, which is a clear use of this strategy.

Therefore, the correct answer for Xiao Ming's second response is (D) Only ③ and ⑤, as he is using a receptive repair strategy and confirming the content of Xiao Mei's request.

You

Please answer the following multiple-choice question as a hearing care professional, providing reasoning and explanation: 14 關於早期療育課程的教練模式（coaching model），下列敘述何者錯誤?
（A）聽語復健專業人員主要藉由在診療室中對親子互動的觀察，提供家長支持與建議
（B）聽語復健專業人員引導家長，將每日[illegible]轉化為孩子學習的機會
（C）家長透過該模式與孩子互動的機會，可以提升自身的自信與賦權
（D）鼓勵使用原本家中有的玩具和素材作為教學工具
15 下列有關聽能訓練（auditory training）活動的描述，何者錯誤?
（A）察覺、分辨、辨識、理解是由易至難的聽覺技巧
（B）當學生總是將「肚子」說成「兔子」時，可嘗試使用「察覺」的活動，釐清學生是聽的問題或是說的問題
（C）對於年幼的初學者，可透過增加訊噪比降低訓練的難度
（D）「察覺」技巧的訓練，可透過玩躲貓貓（Peek-a-boo）的遊戲進行

ChatGPT

Question 14:
This question is about the coaching model in early intervention programs. Let's evaluate each statement for accuracy:

- (A) Auditory rehabilitation professionals primarily offer support and suggestions by observing parent-child interactions in the clinic. This is correct. One of the key aspects of the coaching model is observation and providing feedb[illegible] based on these observations.
- (B) Auditory rehabilitation professionals guide parents to turn everyday activities into learning opportunities for the child. This is also correct and is a fundamental component of the coaching model, which involves integrating learning into daily routines.
- (C) Parents, through this model, get an opportunity to interact with their children, enhancing their own confidence and empowerment. This statement is true. The coaching model aims to empower parents by actively involving them in the intervention process.
- (D) Encourages the use of toys and materials already available at home as teaching tools. This is accurate as well. The coaching model often involves using familiar and readily available resources to facilitate learning.

All these statements accurately describe aspects of the early intervention coaching model. Therefore, none of the options (A, B, C, D) are incorrect.

Question 15:
Now, let's analyze the statements about auditory training activities:

- (A) Awareness, discrimination, identification, comprehension are auditory skills that progress from easy to difficult. This is a correct des[illegible]tion of the hierarchy of auditory skills development.
- (B) When a student consistently mispronounces 'stomach' as 'rabbit,' using 'awareness' activities to clarify whether it's a hearing or speaking issue. This statement seems off. 'Awareness' activities are about detecting the presence or absence of sound, not about distinguishing between similar-sounding words. This scenario would more likely involve 'discrimination' activities.
- (C) For young beginners, the difficulty of training can be reduced by increasing the signal-to-noise ratio. This is correct. Enhancing the signal-to-noise ratio can make it easier for beginners to focus on the target sounds.
- (D) 'Awareness' skill training can be conducted through playing games like Peek-a-boo. This is correct as such games involve detecting the presence or absence of sounds or visual cues.

Given this analysis, the incorrect statement regarding auditory training activities is (B) When a student consistently mispronounces 'stomach' as 'rabbit,' using 'awareness' activities to clarify whether it's a hearing or speaking issue, as it seems to misrepresent the nature of 'awareness' activities in auditory training.

You

please give the most suitable answer for Q14

ChatGPT

For Question 14, regarding the coaching model in early intervention programs, let's reassess the options:

- (A) Auditory rehabilitation professionals primarily offer support and suggestions by observing parent-child interactions in the clinic. While observation is a part of the coaching model, the statement slightly misrepresents the model's essence. The coaching model is more about guiding and empowering parents in natural settings rather than just in the clinical environment.
- (B) Auditory rehabilitation professionals guide parents to turn everyday activities into learning opportunities for the child. This is accurate and a core aspect of the coaching model, where real-life activities are used for learning.
- (C) Parents, through this model, get an opportunity to interact with their children, enhancing their own confidence and empowerment. This is also true and aligns well with the goals of the coaching model.
- (D) Encourages the use of toys and mater[illegible] already available at home as teaching tools. This is correct and reflects the practical and integrative approach of the coaching model.

Considering these points, the most suitable answer for which statement is incorrect about the coaching model in early intervention programs is (A) Auditory rehabilitation professionals primarily offer support and suggestions by observing parent-child interactions in the clinic. This statement is the least representative of the coaching model's emphasis on real-world, family-centered practice. The coaching model extends beyond the clinic, focusing on empowering parents in their home and daily environments, rather than just providing support based on clinical observations.

You

Please answer the following multiple-choice question as a hearing care professional, providing reasoning and explanation: 16 在說話訓練中，治療師指著自己的耳朵和小美說：「你看，這是耳朵！你摸摸看！」。此描述最接近下列何種訓練方法?
（A）聽覺口語法（auditory-verbal method） （B）多感官法（multisensory approach）
（C）手語溝通法（sign language communication） （D）口手標音法（cued speech）
17 聽力師欲為一名植入人工電子耳的聽[illegible]調頻，但發現孩子在調頻過程中無法給予一致的回答，因此安排訓練課程，在課程中，聽力師挑選了三樣樂器：三角鐵、大鼓、響板；後續聽力師輕敲大鼓，詢問孩子這個聲音是「小聲」或「大聲」，之後又加重敲大鼓的力道，再次詢問孩子這個聲音是「小聲」或「大聲」；之後再更換為不同樂器，進行相同練習。聽力師進行的包含那些聽覺訓練?
（A）僅音高辨識 （B）僅音強辨識
（C）包含音高辨識和音強辨識 （D）包含音高辨識和音強察覺

ChatGPT

Question 16:
The scenario describes a therapist pointing to their own ear and encouraging Xiao Mei to look and touch, saying, "Look, this is an ear! You touch and see!" Let's evaluate which training method this description most closely aligns with:

- (A) Auditory-Verbal Method: This method focuses on using hearing as the primary sense for developing spoken language and typically does not emphasize the use of visual or tactile cues to this extent.
- (B) Multisensory Approach: This approac[illegible]corporates multiple senses, including visual, auditory, and tactile cues, to aid in language and speech development. The therapist's action of pointing and encouraging tactile exploration fits well with this approach.
- (C) Sign Language Communication: This method focuses on using sign language as the primary means of communication and does not typically involve tactile exploration of body parts related to hearing.
- (D) Cued Speech: This method involves the use of hand shapes placed near the mouth to aid lip-reading, which is not what is being described here.

Based on this analysis, the description most closely aligns with (B) Multisensory Approach, as it involves using visual and tactile cues alongside auditory information.

Question 17:
In this scenario, an audiologist is conducting auditory training with a child who has a cochlear implant. The activities involve identifying the loudness of sounds made by different musical instruments. Let's evaluate the types of auditory training being conducted:

- (A) Only Pitch Recognition: Pitch recognition involves distinguishing between different frequencies of sound. The description do[illegible]ot indicate that the child is being asked to differentiate between pitches.
- (B) Only Loudness Recognition: The activity described involves asking the child to identify whether the sound is 'soft' or 'loud,' which directly relates to loudness recognition.
- (C) Both Pitch Recognition and Loudness Recognition: There is no mention of pitch recognition in the scenario, only loudness recognition.
- (D) Both Pitch Recognition and Loudness Awareness: Again, the focus is only on loudness, not pitch.

Therefore, the correct answer is (B) Only Loudness Recognition, as the activities described are focused on identifying the loudness (intensity) of the sounds and not their pitch.

You

Please answer the following multiple-choice question as a hearing care professional, providing reasoning and explanation: 18 寶媽帶著三歲的小寶在家裡玩音樂椅（music chair）遊戲來進行聽覺訓練，寶媽用手機播放「妹妹揹著洋娃娃」歌曲，讓小寶繞著椅子走，當音樂切換到「王老先生有塊地」時，小寶就要坐下。這項活動最可能訓練那一項聽覺技巧?
（A）察覺（detection） （B）理解（co[illegible]ehension） （C）辨識（identification） （D）區辨（discrimination）
19 小妤 17 歲，剛植入人工電子耳需要做聽能訓練，為使其聽得比較清楚，在說話慢一點的情況下，下列關於語音時長敘述何者錯誤?
（A）音素與其他音結合時，會產生強度和時長的變化
（B）說話時母音時長比子音長
（C）有聲子音在詞尾時，母音時長會縮短
（D）塞音在母音 - 子音 - 母音模式時，會有時長的轉變

ChatGPT

Question 18:
The described activity involves playing the "music chair" game for auditory training, where the child walks around a chair to one song and sits down when the song changes to another. This activity aims to train a specific auditory skill. Let's review the options:

- (A) Detection: This involves the ability to notice the presence or absence of sound. While detection is a part of this activity, the game requires more than just detecting sound; it involves recognizing when the sound changes.
- (B) Comprehension: This involves understanding the meaning of what is heard, which is not the primary focus of this game.
- (C) Identification: This refers to the ability to recognize and label specific sounds or speech. The activity involves identifying the change in music, which aligns with this skill.
- (D) Discrimination: This involves distinguishing between different sounds or words. The game requires discriminating between two different songs, which is a form of auditory discrimination.

Given these considerations, the most likely auditory skill being trained in this activity is (D) Discrimination, as the child needs to distinguish between two different songs and react accordingly.

Question 19:
This question concerns speech characteristics, particularly in the context of someone with a newly implanted cochlear implant. Let's analyze each statement for accuracy:

- (A) When phonemes combine with others, there are changes in intensity and duration. This is generally true. The combination of sounds in speech can affect their individual characteristics.
- (B) Vowel duration is longer than consona[illegible]uration in speech. This is typically correct; vowels usually have longer durations than consonants in spoken language.
- (C) The duration of a vowel shortens when a voiced consonant follows it at the end of a word. This statement is not accurate according to typical speech patterns. Usually, the presence of a voiced consonant at the end of a word can lengthen the preceding vowel, not shorten it.
- (D) In a vowel-consonant-vowel pattern, stop consonants will show changes in duration. This is correct. The duration of stop consonants (like p, t, k) can vary depending on their position in a word and the surrounding vowels.

Based on this analysis, the incorrect statement regarding speech duration is (C) The duration of a vowel shortens when a voiced consonant follows it at the end of a word, as this typically results in the lengthening, not shortening, of the vowel.

You

Please answer the following multiple-choice question as a hearing care professional, providing reasoning and explanation: 20 小雪 28 歲，因受傷造成陡降型聽損，500 Hz 為 20 dB HL，其他頻率均 80 dB HL 或更重；做聽覺訓練時，[illegible]考慮下列何項?
（A）構音位置的訊息主要來自低頻，是個[illegible]優勢
（B）語音最大能量分布在 1 kHz 以下，此頻率範圍的語音為個案優勢

（C）無聲子音主要分布在低頻，有助於個案聆聽
（D）語音強度範圍約在 65 dB SPL，訓練時說話音量需大於此音量
21 「成功聆聽發展模式（Developmental Approach to Successful Listening II, DASL II）」是一種結構式的聽覺訓練課程，將聽覺技巧拆解成好幾個步驟或層次，協助聽損兒童與成人運用殘存聽力有系統地發展聽覺能力，下列聽覺能力由簡單至困難之排序為何？ ①可察覺環境音的突然停止 ②可察覺音量大的語音或音節 ③可察覺低頻且音量大的環境音 ④可從三個熟悉聲音當中選出唯一不同的聲音
（A）③①②④ （B）③②①④ （C）②①④③ （D）②③①④

ChatGPT

Question 20:

Xiao Xue, 28 years old, has a steeply sloping hearing loss with 500 Hz at 20 dB HL and other frequencies at 80 dB HL or worse. In planning auditory training, we need to consider her hearing profile. Let's evaluate each option:

- (A) Information about the place of articulat[illegible]n mainly comes from low frequencies, which is an advantage for the client. This is true. Low-[illegible]equency sounds carry significant information about the place of articulation, and since Xiao Xue has better hearing at 500 Hz, she might find it easier to perceive these cues.
- (B) The greatest energy in speech is below 1 kHz, which is advantageous for the client. This is not entirely accurate for Xiao Xue. While it's true that much of the energy in speech is in the lower frequencies, her hearing loss is steeply sloping, and she has significant hearing loss at frequencies above 500 Hz.
- (C) Voiceless consonants are primarily in low frequencies, which helps the client in listening. This is incorrect. Voiceless consonants are typically found in higher frequency ranges, which would be a challenge for Xiao Xue.
- (D) Speech intensity is around 65 dB SPL, and training speech volume needs to be above this level. This is generally true for auditory training; however, it should be noted that simply increasing volume might not always be sufficient for someone with a steeply sloping hearing loss like Xiao Xue's.

Based on this analysis, the most suitable consideration for Xiao Xue's auditory training is (A) Information about the place of articulation [illegible]inly comes from low frequencies, which is an advantage for the client.

Question 21:

The "Developmental Approach to Successful Listening II (DASL II)" is a structured auditory training program that breaks down auditory skills into several steps or levels. Let's order the auditory abilities from simplest to most difficult:

- ① Detecting the sudden stop of environmental sounds.
- ② Detecting loud speech or syllables.
- ③ Detecting low-frequency and loud environmental sounds.
- ④ Identifying the one different sound among three familiar sounds.

The progression typically starts with the basic awareness of sound (detection), moves to discrimination of sounds (distinguishing differences), and then to identification and comprehension. Thus, the correct order from simplest to most difficult should start with detecting basic loud sounds (②), detecting sudden stops in sounds (①), recognizing low-frequency sounds (③), and finally distinguishing between familiar sounds (④).

Therefore, the correct order is (C) ②①④③.

You

Please answer the following multiple-choice question as a hearing care professional, providing reasoning and explanation: 22 聽力師為年長者實施聽覺復健團體治療時，把複雜的行為切割成次行為（subbehaviors），此為那一種強化自我效能策略？
（A）精熟的經驗（mastery experience）
（B）替代的經驗（vicarious experience）
（C）口語說服（verbal persuasion）
（D）生理和情緒狀態（physiological and emotional states）
23 小智有重度聽損，說話時總是有鼻音，尤其是說「一」時，特別明顯。治療師或教師可以用那些方法引導其發出正確的音呢？
①加強聽能分辨有無鼻音的「一」音
②運用鏡子，進行視覺提示
③運用已經能正確發音的送氣聲母，結合「一」並拉長母音，引導出沒有鼻音的「一」音後，再略去
聲母
④讓雙唇快速開合，變換不同的口型
（A）僅①③ （B）僅①②③ （C）僅③④ [illegible]）①②③④

ChatGPT

Question 22:

This question is about a strategy to enhance self-efficacy in older adults during group auditory rehabilitation therapy by breaking down complex behaviors into subbehaviors. Let's evaluate each option:

- (A) Mastery Experience: This involves gaining confidence through successfully completing tasks or learning new skills. Breaking down complex behaviors into manageable subbehaviors aligns with this approach, as it allows individuals to master each part step by step.
- (B) Vicarious Experience: This involves learning by observing others, which is not directly indicated in the scenario.
- (C) Verbal Persuasion: This is about encouraging and persuading individuals to believe they can succeed in a task. While important, it's not specifically about breaking down tasks into subbehaviors.
- (D) Physiological and Emotional States: T[illegible]efers to the impact of one's physical and emotional responses on their self-efficacy, which is not the focus of the described strategy.

The most appropriate answer is (A) Mastery Experience. By breaking down complex behaviors into subbehaviors, the audiologist is facilitating mastery experiences, where the older adults can gain confidence by successfully mastering each subbehavior.

Question 23:

This question asks about methods to guide Xiao Zhi, who has severe hearing loss and nasal speech, particularly when saying the word "一" (one). Let's review each method:

- ① Enhance auditory discrimination of the sound "一" with and without nasalization. This is a relevant method, especially for someone with hearing loss, to learn to distinguish between correct and incorrect pronunciations.
- ② Use a mirror for visual cues. This can help Xiao Zhi see the shape of his mouth and position of his tongue, aiding in correct articulation.
- ③ Use aspirated initials that he can pronounce correctly, combine them with "一," elongate the vowel, and then gradually remove the initia[illegible]consonant. This is a technique often used in speech therapy to modify and correct speech sou[illegible]s.
- ④ Have the lips rapidly open and close, changing different mouth shapes. While changing mouth shapes can be helpful in some speech therapy contexts, it may not be directly relevant to correcting nasalization in this specific case.

Given these considerations, the most suitable methods for guiding Xiao Zhi to produce the correct sound are ①, ②, and ③. Therefore, the correct answer is (B) Only ①, ②, and ③. These methods directly address the auditory, visual, and articulatory aspects needed to correct the nasalized pronunciation of "一."

You

Please answer the following multiple-choice question as a hearing care professional, providing reasoning and explanation: 24 有關聲學在聽覺技巧訓練的應用，下列敘述何者錯誤？
（A）母音是依共振峰的不同來區辨
（B）子音是依發音的方法、是否有聲、發音位置的特質來區辨
（C）助聽器的優勢是在低頻率，但這並不是超語段（suprasegmental）、聲調、母音能量分布的主要區域
（D）人工電子耳的優勢在高頻率，這是許[illegible]音能量分布的主要區域
25 有關自我效能（self-efficacy）的敘述，下列何者正確？ ①是非特定領域相關的信念 ②指個人對於自己能成功勝任一項任務的信念 ③聽能復健中個體對於自我管理困難溝通情境能力的信念 ④自我效能問卷的題項主要是以「我將會...」的敘述方式，藉以了解個人的效能
（A）①②④ （B）僅①② （C）僅②③ （D）僅③④

ChatGPT

Question 24:

Let's analyze each statement about the application of acoustics in auditory skills training for its accuracy:

- (A) Vowels are distinguished based on different formants. This is correct. Vowels are characterized by their formant frequencies, which are resonant frequencies of the vocal tract.
- (B) Consonants are distinguished by their manner of articulation, voicing, and place of articulation. This is also correct. Consonants differ in how and where in the vocal tract the airflow is restricted or obstructed, and whether the vocal cords vibrate.
- (C) Hearing aids have advantages in low frequencies, but this is not the main area for suprasegmentals, tone, or vowel energy [illegible]bution. This statement is somewhat misleading. Modern hearing aids are designed to amplify a range of frequencies, including those important for understanding speech, which often extends into the higher frequencies important for suprasegmentals and certain vowel characteristics.
- (D) Cochlear implants have advantages in high frequencies, which is where many consonant energies are concentrated. This is accurate. Cochlear implants are often effective at delivering high-frequency sounds, which are crucial for understanding many consonants.

The incorrect statement is (C) Hearing aids have advantages in low frequencies, but this is not the main area for suprasegmentals, tone, or vowel energy distribution. Modern hearing aids are typically designed to provide a balanced amplification across a wide range of frequencies, including those important for speech understanding.

Question 25:

Now let's evaluate each statement about self-efficacy:

- ① It's a belief not specific to any domain. This is incorrect. Self-efficacy is often specific to particular tasks or contexts.
- ② It refers to an individual's belief in their [illegible]ability to successfully perform a task. This is correct. Self-efficacy is about one's confidence in their ability to execute actions required for specific performances.
- ③ In auditory rehabilitation, it's the individual's belief in their ability to self-manage in difficult communication situations. This is a specific application of self-efficacy in auditory rehabilitation, making it correct.
- ④ Self-efficacy questionnaires primarily use statements like "I will be able to..." to understand an individual's efficacy. This is a typical way of framing self-efficacy questionnaire items, focusing on future capabilities and actions.

Therefore, the correct answer is (C) Only ② and ③. These statements accurately reflect the concept of self-efficacy as it relates to an individual's belief in their capabilities, particularly in the context of auditory rehabilitation.

You

Please answer the following multiple-choice question as a hearing care professional, providing reasoning and explanation: 26 小美在接[illegible]覺口語法（auditory-verbal method）訓練前，語句測驗能辨識出 35%的字詞，訓練後正確辨識率略升為 41%。專業人員宜透過何種數值評估此微小改變值，在改善聽能上是具有意義的？
（A）再測變異性（test-retest variability） （B）臨床顯著性（clinical significance）
（C）學習效應（learning effects） （D）評分者間信度（inter-rater reliability）
27 老年患者除了感音神經性聽損和言語辨識能力下降之外，還可能伴隨聽覺處理（auditory processing）的下降，下列那一個測驗可用來評估老年人的聽覺處理能力？
（A） Speech Sound Pattern Discrimination Test（SSPDT）
（B） Nonsense Syllable Test（NST）
（C） Hearing Problem Inventory（HPI）
（D） Test of Basic Auditory Capabilities（TBAC）

ChatGPT

Question 26:

In this scenario, Xiao Mei's word recognition score in sentence tests improved from 35% to 41% after undergoing auditory-verbal method training. To assess whether this small change is meaningful in terms of auditory improvement, let's evaluate each option:

- (A) Test-Retest Variability: This refers to t[illegible]ariation in test scores across different testing sessions. While it's important to consider, it doesn't directly assess the significance of the change in scores.
- (B) Clinical Significance: Clinical significance evaluates whether the change in scores is meaningful in terms of practical or clinical impact. This is what is needed to assess whether an improvement from 35% to 41% represents a meaningful change in the patient's capabilities.
- (C) Learning Effects: This refers to improvements in test scores due to familiarity with the test rather than actual improvements in the skill being tested. While this is a factor to consider, it doesn't alone assess the significance of the change.
- (D) Inter-Rater Reliability: This is about the consistency of scores when assessed by different evaluators, which is not directly relevant to assessing the significance of the score change in this case.

The most appropriate approach to assess the meaningfulness of the change in Xiao Mei's scores is (B) Clinical Significance. This approach will help determine if the improvement, though seemingly small, is significant in terms of her auditory abilities and practical communication skills.

Question 27:

Regarding the assessment of auditory processing abilities in older adults, let's review each test option:

- (A) Speech Sound Pattern Discrimination Test (SSPDT): This test is designed to assess the ability to discriminate between different speech sound patterns, which is relevant for auditory processing, especially in older adults.
- (B) Nonsense Syllable Test (NST): This test assesses the ability to recognize and repeat nonsense syllables, which can be indicative of auditory processing capabilities, but it may be more focused on speech perception.
- (C) Hearing Problem Inventory (HPI): This is typically a questionnaire assessing the impact of hearing loss on daily life, not a direct measure of auditory processing abilities.
- (D) Test of Basic Auditory Capabilities (TBAC): This test evaluates basic auditory skills, which may include some aspects of auditory processing, but it is not specifically designed to assess complex auditory processing abilities typical in older adults.

Considering the specific need to assess auditory processing abilities in older adults, the most relevant test would be (A) Speech Sound P[illegible]ern Discrimination Test (SSPDT). This test is specifically designed to evaluate the ability [illegible] discriminate between different speech sounds, which is a key aspect of auditory processing, particularly relevant in the context of aging.

You

Please answer the following multiple-choice question as a hearing care professional, providing reasoning and explanation: 28 聽力師做助聽器諮詢時，病患擔心女友會認為戴助聽器看起來很老，所以決定不戴。依據「理性情緒行為治療法（rational emotive behavior therapy, REBT）」，下列何者是首先要採取的步驟？
（A）告知：如果不戴可能會產生溝通不良的狀況
（B）提出質疑：女友會不會認為戴助聽器的都是老人
（C）舉證：很多傑出的人也一樣有戴助聽器
（D）說明：有的助聽器體積很小，外觀上看不出來有佩戴
29 陳先生在工廠工作，領取基本工資，在聽覺復健諮詢時提到工作環境又吵又熱，經常無法佩戴助聽器工作，以致聽不到危險訊號的播放，必須透過同事提醒。此時宜優先考慮下列何者？
（A）選用較高階、防水性較佳、且能容易戴上與取下之助聽器
（B）儘速為他安排聽覺復健課程，提升他在吵雜環境的讀話能力
（C）除了工作場所已有的聽覺警示系統，也加裝視覺警示系統
（D）改用手語溝通，以解決不戴助聽器就[illegible]不到訊號的問題

ChatGPT

Question 28:

Rational Emotive Behavior Therapy (REBT) is a form of cognitive-behavioral therapy that focuses on identifying and changing irrational beliefs. The patient's concern about appearing old due to wearing hearing aids is an irrational belief that REBT would aim to challenge. Let's analyze each option:

- (A) Informing the patient about potential communication difficulties if they don't wear hearing aids addresses practical consequences but doesn't directly challenge the irrational belief.
- (B) Questioning whether the girlfriend would actually think that all people who wear hearing aids are old directly targets the irrational belief. This approach is consistent with REBT, which encourages questioning and challenging irrational thoughts.
- (C) Providing evidence that many accomplished people wear hearing aids also challenges the irrational belief, but it's more about offering counterexamples rather than directly questioning the belief.
- (D) Explaining that some hearing aids are [illegible]ll and unnoticeable addresses the concern but does not challenge the underlying belief about age and hearing aids.

The first step according to REBT would be to directly challenge the irrational belief, which is best done in option (B) Questioning whether the girlfriend would actually think that all people who wear hearing aids are old.

Question 29:

In this scenario, Mr. Chen works in a noisy and hot factory and cannot wear his hearing aids, which affects his ability to hear danger signals. Let's consider the best course of action:

- (A) Choosing higher-end, waterproof hearing aids that are easy to wear and remove might help, but it doesn't address the issue of not being able to hear danger signals while not wearing them.
- (B) Arranging auditory rehabilitation to improve speech understanding in noisy environments is beneficial for overall communication but doesn't solve the immediate safety concern of hearing danger signals at work.
- (C) Installing a visual warning system in addition to the auditory warning system at the workplace addresses the specific safety concern dire[illegible]ly. This ensures that Mr. Chen can be alerted to dangers even when he can't wear his hear[illegible] aids.
- (D) Using sign language for communication doesn't address the issue of hearing danger signals in the workplace.

The priority should be ensuring Mr. Chen's safety in the workplace, which is best achieved by option (C) Installing a visual warning system in addition to the auditory warning system. This approach directly addresses the immediate concern of detecting danger signals when he cannot wear his hearing aids.

You

Please answer the following multiple-choice question as a hearing care professional, providing reasoning and explanation: 30 Musiek 和 Chermak（1994）建議評估中樞聽覺處理障礙（central auditory processing disorder, CAPD）的項目為何？ ①聽覺型態（時間性的）辨識 ②涉及雙聽（雙耳的）分開與整合的任務 ③背景競爭中的單耳任務
（A）僅① （B）僅①② （C）僅②③ （D）①②③
31 關於兒童聽覺處理障礙（auditory processing disorder）的篩檢，下列敘述何者較適當？
（A）聽覺處理障礙可能伴隨學習障礙，應於學齡前進行篩檢及診斷並儘快進行早期療育
（B）聽覺處理障礙可以用耳聲傳射（OAE[illegible]自動聽性腦幹反應（aABR）來做為篩檢轉介標準
（C）聽覺行為量表（Scale of Auditory Behaviors, SAB）為聽覺處理障礙的行為篩檢工具
（D）聽覺處理障礙篩檢應在純音聽力篩檢未通過後再進行，可以降低轉介率及醫療成本

ChatGPT

Question 30:

Musiek and Chermak's suggestions for assessing Central Auditory Processing Disorder (CAPD) involve various tasks that test different aspects of auditory processing. Let's examine each option:

- ① Auditory pattern (temporal) recognition: This involves recognizing patterns in sounds over time, which is a crucial aspect of central auditory processing.
- ② Tasks involving binaural (both ears) separation and integration: This refers to the ability to separate and integrate sounds coming into both ears, a key component of auditory processing.
- ③ Monaural tasks in competing background noise: This involves understanding sounds from one ear when there is competing noise, which is also an important aspect of CAPD assessment.

All these tasks are relevant to CAPD assessment as they test different capabilities of central auditory processing. Therefore, the correc[illegible]swer is (D) ①②③, as all these tasks collectively contribute to a comprehensive assessment of CAPD.

Question 31:

Now, let's consider the most appropriate statement regarding the screening of Auditory Processing Disorder (APD) in children:

- (A) APD often co-occurs with learning disabilities, and early screening and diagnosis before school age are recommended for early intervention. This is accurate as early detection and intervention are crucial, especially since APD can impact learning.
- (B) APD can be screened using Otoacoustic Emissions (OAE) and automated Auditory Brainstem Responses (aABR). This statement is incorrect. OAE and aABR tests are used to assess peripheral hearing function, not central auditory processing.
- (C) The Scale of Auditory Behaviors (SAB) is a behavioral screening tool for APD. This is correct. SAB is a questionnaire that helps in identifying behaviors associated with auditory processing difficulties.
- (D) APD screening should be conducted after failing pure-tone hearing screenings to reduce referral rates and medical costs. This is a [illegible]ctical approach, but it's not necessarily the best practice for identifying APD, which may occur even with normal peripheral hearing.

Considering the specific nature of APD, the most appropriate statement is (C) The Scale of Auditory Behaviors (SAB) is a behavioral screening tool for APD, as it directly addresses the behavioral aspects of APD, which are crucial for its identification and subsequent management.

You

Please answer the following multiple-choice question as a hearing care professional, providing reasoning and explanation: 32 左腦腫瘤患者雙耳純音聽閾值於正常範圍，接受雙耳異訊數字測試（dichotic digits listening test）發現僅左耳有缺失的臨床解釋，下列敘述何者最恰當？
（A）因為左耳的聽覺訊息大部分是直接傳到左腦 （B）因為多數人的主管聽語功能是位於左腦顳葉
（C）合併有周邊聽力問題，如耳蝸或耳蝸後病變 （D）腫瘤影響胼胝體功能，影響半球間傳輸功能
33 小夏，小學三年級，課業成績低落，專注力也比較不足，帶至醫院做各項檢查後，下列那個結果之組合，最可能懷疑他是中樞聽覺處理障礙（CAPD）個案？ ①PTA 檢查雙耳正常 ②OAE 雙耳未通過， ABR 檢查結果雙耳有聽損 ③噪音環境的語詞分辨測試為 60% ④耳蝸電圖（EcochG）出現耳蝸微音（CM）
（A）僅①③ （B）①③④ （C）僅②④ [illegible]①②③

ChatGPT

Question 32:

In a patient with a left brain tumor who has normal pure-tone hearing thresholds but shows deficits only in the left ear on a dichotic digits listening test, let's analyze the most appropriate clinical explanation:

- (A) Because most of the auditory information from the left ear is directly sent to the left brain. This is not entirely accurate. While each ear sends information to both sides of the brain, the predominant pathway is contralateral (to the opposite side of the brain).
- (B) Because the dominant auditory language functions are located in the left temporal lobe for most people. This is a more accurate explanation. In most right-handed individuals (and many left-handed individuals), the left hemisphere of the brain, especially the temporal lobe, plays a crucial role in processing auditory language information. A tumor in the left brain could disrupt this processing, especially in dichotic listening tasks.
- (C) Combined peripheral hearing problen[illegible]uch as cochlear or retrocochlear lesions. This is unlikely since the pure-tone thresholds are normal.
- (D) The tumor affects the corpus callosum, impacting interhemispheric transfer. While this could be a factor, the dichotic digits test primarily assesses the ability of the auditory cortices to process information, with the left hemisphere typically being more crucial for language.

The most appropriate explanation is (B) Because the dominant auditory language functions are located in the left temporal lobe for most people. This accounts for the observed deficit in the dichotic listening test due to the left-brain tumor.

Question 33:

For Xiao Xia, a third-grader with poor academic performance and attention issues, let's evaluate which combination of results could suggest Central Auditory Processing Disorder (CAPD):

- ① Normal bilateral Pure-Tone Audiometry (PTA). CAPD can occur even with normal peripheral hearing.
- ② Failed Otoacoustic Emissions (OAE) in both ears and Auditory Brainstem Response (ABR) showing bilateral hearing loss. This sugge[illegible] a peripheral hearing problem rather than CAPD.
- ③ Word discrimination in noise at 60%. Di[illegible]culty understanding speech in noise is a common feature of CAPD.
- ④ Electrocochleography (EcochG) showing cochlear microphonics (CM). This is more indicative of cochlear function and does not necessarily point towards CAPD.

Considering these options, the most likely combination that could suggest CAPD in the context of normal peripheral hearing is (A) Only ① and ③. Normal PTA results combined with difficulty in speech-in-noise discrimination is more indicative of a central processing issue rather than a peripheral hearing problem.

You

Please answer the following multiple-choice question as a hearing care professional, providing reasoning and explanation: 34 關於耳鳴減敏療法（Tinnitus Retraining Therapy, TRT），下列敘述何者錯誤？
（A）心理諮詢與聲音治療結合的模式
（B）以中樞神經系統的適應改變原理，讓耳鳴習慣化而減少對耳鳴的反應
（C）使用噪聲產生器遮蔽耳鳴聲音，讓患者無法感知耳鳴
（D）制約患者使其以正向的方式面對耳鳴[illegible]，不要用情緒化的方式反應
35 下列何者最不可能是客觀耳鳴（objective tinnitus）常見原因之一？
（A）聽神經譜系病變（auditory neuropathy spectrum disorder）
（B）良性顱內壓升高（benign intracranial hypertension）
（C）中耳副神經節瘤（paraganglioma of middle ear）
（D）動靜脈瘻管（arteriovenous shunt）

ChatGPT

Question 34:

Tinnitus Retraining Therapy (TRT) is a specific approach to managing tinnitus. Let's evaluate each statement for its accuracy:

- (A) A combination of psychological counseling and sound therapy. This is correct. TRT combines these two elements to help patients habituate to their tinnitus.
- (B) Based on the principle of adaptation changes in the central nervous system to habituate tinnitus and reduce the reaction to it. This is also correct. The goal of TRT is to achieve habituation to tinnitus.
- (C) Uses noise generators to mask the tin[illegible]s sound, making it imperceptible to the patient. This statement is not entirely accurate. TRT uses sound therapy, but the goal is not necessarily to mask the tinnitus so that it's completely imperceptible; rather, it's to help the brain learn to ignore it.
- (D) Conditioning the patient to confront tinnitus in a positive way and not react emotionally. This is correct as TRT includes components that help change the patient's reaction and relationship to their tinnitus.

Therefore, the incorrect statement about Tinnitus Retraining Therapy is (C) Uses noise generators to mask the tinnitus sound, making it imperceptible to the patient. The primary goal of TRT is not to mask the tinnitus but to help the brain habituate to it and diminish its impact.

Question 35:

Now let's look at potential causes of objective tinnitus, which is a type of tinnitus that can actually be heard by an examiner:

- (A) Auditory Neuropathy Spectrum Disorder: This is a disorder of the auditory nerve and is more likely associated with subjective rather tha[illegible]objective tinnitus.
- (B) Benign Intracranial Hypertension: This [illegible]an cause objective tinnitus due to increased pressure within the skull affecting blood flow and other structures.
- (C) Paraganglioma of Middle Ear: A tumor in the middle ear can create sounds that might be heard by both the patient and an examiner, potentially leading to objective tinnitus.
- (D) Arteriovenous Shunt: Abnormal connections between arteries and veins can create turbulent blood flow, which can cause sounds that contribute to objective tinnitus.

Given these options, the least likely cause of objective tinnitus is (A) Auditory Neuropathy Spectrum Disorder. This condition is more associated with subjective tinnitus due to its nature as a nerve disorder, as opposed to the vascular or mechanical causes typically associated with objective tinnitus.

You

Please answer the following multiple-choice question as a hearing care professional, providing reasoning and explanation: 36 下列那一個解剖構造只存在於球囊（saccule）與橢圓囊（utricle）內？
（A）壺腹（ampulla） （B）動纖毛（kin[illegible]lium）
（C）靜纖毛（stereocilia） （D）耳石膜 [illegible]tolithic membrane）
37 在前庭系統健全的前提下，以聲音刺激進行頸前庭誘發肌電位檢查（cervical vestibular evoked myogenic potential, cVEMP），下列何種情況最不影響肌電位誘發成功的比率（response rate of cVEMP）？
（A）耳膜破損 60%的無耳漏慢性中耳炎
（B）中耳積液（otitis media with effusion）併有耳膜塌陷
（C）感覺神經性聽力障礙
（D）中後期的耳硬化症（otosclerosis）

ChatGPT

Question 36:

In considering the anatomical structures that are unique to the saccule and utricle:

- (A) Ampulla: The ampulla is a structure found in the semicircular canals, not in the saccule or utricle.
- (B) Kinocilium: Kinocilia are part of the hair cells found in various parts of the inner ear, including the saccule and utricle, but they are not exclusive to these structures.
- (C) Stereocilia: Stereocilia are also part of [illegible]hair cells in the inner ear, but like kinocilia, they are not unique to the saccule and utricle.
- (D) Otolithic Membrane: The otolithic membrane is a structure found in the saccule and utricle, containing otoliths (ear stones) that contribute to the sense of gravity and linear acceleration. This membrane is specific to the saccule and utricle.

The correct answer is (D) Otolithic Membrane, as it is a structure that exists only in the saccule and utricle.

Question 37:

Considering factors that might not significantly affect the response rate of cervical Vestibular Evoked Myogenic Potential (cVEMP) testing in a person with an intact vestibular system:

- (A) A 60% tympanic membrane perforation with non-oozing chronic otitis media: This could potentially affect the transmission of sound to the inner ear, impacting cVEMP responses.
- (B) Otitis media with effusion and tympanic membrane retraction: This condition could also affect sound transmission and thus cVEMP responses.
- (C) Sensorineural hearing loss: cVEMP prim[illegible]rily assesses the saccule and the inferior vestibular nerve, which may not be significantly affec[illegible]d by sensorineural hearing loss, especially if the loss is not severe.
- (D) Advanced otosclerosis: This condition primarily affects the stapes and the oval window and can impact the transmission of sound to the inner ear, potentially affecting cVEMP responses.

Given these options, the condition that is least likely to affect the response rate of cVEMP is (C) Sensorineural hearing loss, especially if the hearing loss is not severe. cVEMP testing is less dependent on the cochlear function and more on the saccular and inferior vestibular nerve function.

You

Please answer the following multiple-choice question as a hearing care professional, providing reasoning and explanation: 38 動態姿態評估檢查（dynamic posturography）時，當視訊不斷移動，下列那一項維持平衡的機制無法有效啟動？
（A）本體感覺 （B）前庭功能 （C）視覺系統 （D）前庭脊椎反射
39 除了中樞性前庭障礙之外，下列那一種疾病較可能產生純粹向下眼振（pure down-beat nystagmus）？
（A）雙側上半規管間隙症（superior cana[illegible]hiscence）
（B）雙側後半規管良性陣發姿勢性眩暈（benign paroxysmal positional vertigo）
（C）前庭神經炎急性期
（D）刺激期（irritative phase）之單側梅尼爾氏症（Meniere's disease）

ChatGPT

Question 38:

In dynamic posturography, when visual surroundings are continuously moving, the effectiveness of certain balance mechanisms is challenged. Let's analyze each option:

- (A) Proprioception: Proprioception refers to the body's ability to perceive its own position in space. This mechanism does not rely on visual input, so it should still be able to function effectively even when the visual surroundings are moving.
- (B) Vestibular Function: The vestibular system, located in the inner ear, helps maintain balance and spatial orientation. It functions independently of visual input and should remain effective.
- (C) Visual System: The visual system plays a critical role in maintaining balance by providing external spatial information. When visual surroundings are continuously moving, it can be challenging for the visual system to provi[illegible]ccurate information for balance, making it less effective.
- (D) Vestibulospinal Reflex: This reflex helps maintain posture and balance in response to changes detected by the vestibular system. It should still function effectively in the presence of moving visual surroundings.

Given this analysis, the mechanism that would be least effective in maintaining balance when visual surroundings are continuously moving is (C) the Visual System, as it relies on stable visual cues to assist in balance and orientation.

Question 39:

Regarding the condition most likely to cause pure down-beat nystagmus, a specific type of eye movement, aside from central vestibular disorders:

- (A) Bilateral Superior Canal Dehiscence: This condition typically causes symptoms like vertigo and oscillopsia, but it's not commonly associated with pure down-beat nystagmus.
- (B) Bilateral Posterior Canal Benign Paroxysmal Positional Vertigo (BPPV): BPPV typically causes episodic vertigo related to head position [illegible]nges, and the nystagmus pattern is usually not pure down-beat.
- (C) Acute Phase of Vestibular Neuritis: This condition can cause nystagmus, but it's typically horizontal or torsional, not pure down-beat.
- (D) Irritative Phase of Unilateral Meniere's Disease: Meniere's disease is associated with episodic vertigo, hearing loss, and tinnitus, but pure down-beat nystagmus is not a typical manifestation.

None of these conditions are typically associated with pure down-beat nystagmus, which is more commonly linked with central nervous system disorders. However, if one had to be chosen as the most likely among these options, it would be (A) Bilateral Superior Canal Dehiscence, given its direct involvement with the vestibular system, though it's important to note that this association is not strong.

You

Please answer the following multiple-choice question as a hearing care professional, providing reasoning and explanation: 40 下列關於冰水溫差測試（ice water caloric test）之敘述，何者錯誤？
（A）耳鏡檢查發現耳膜穿孔者應避免冰水[illegible]測試
（B）一般可用乾淨或無菌水加冰塊備置約 2 c.c.用空針灌注耳道 20s
（C）如果沒有誘發眼振，代表前庭系統完全失去功能
（D）改趴睡出現小振幅眼振反向或消失，代表測試耳前庭尚有殘存功能

41 對於左側前庭功能喪失而右側前庭功能健全的年輕人，若施行右側頭部脈衝測試時，前庭眼反射增益值（VOR gain）最可能為下列何者？
（A） 1 < VOR gain < 2 （B） VOR gain = 1 （C） 0.5 < VOR gain < 1 （D） VOR gain = 0

ChatGPT

Question 40:

Let's analyze each statement about the ice water caloric test to identify the incorrect one:

- (A) Otoscopic examination finding a tympanic membrane perforation is a contraindication for ice water caloric testing. This is correct. Performing the test with a perforated eardrum can introduce water into the middle ear, which can be harmful.
- (B) Typically, clean or sterile water with ice cubes is prepared to about 2 c.c. and instilled in the ear canal for 20 seconds using a syringe. This is a standard procedure for the ice water caloric test and is correct.
- (C) If nystagmus is not induced, it indicates complete loss of vestibular function. This statement is misleading. While failure to induce nystagmus may suggest impaired vestibular function, it does not conclusively indicate a complete loss. Other factors could also affect the test results.
- (D) If a change in position to prone causes the small-amplitude nystagmus to reverse direction or disappear, it indicates some residual vestibular function in the tested ear. This is correct. The response to position changes can provide information about residual vestibular function.

The incorrect statement about the ice water caloric test is (C) If nystagmus is not induced, it indicates complete loss of vestibular function. The absence of nystagmus may suggest impaired function, but it does not conclusively indicate a total loss of vestibular function.

Question 41:

Considering the vestibulo-ocular reflex (VOR) gain in a young individual with loss of left vestibular function but intact right vestibular function, during a head impulse test to the right side:

- (A) 1 < VOR gain < 2: This would indicate an overly compensatory or hyperactive response, which is unlikely in this scenario.
- (B) VOR gain = 1: This represents a normal VOR gain, which is expected when the head impulse test is conducted towards the healthy (right) side.
- (C) 0.5 < VOR gain < 1: This would indicate a reduced VOR gain, more likely if the test were conducted towards the affected (left) side.
- (D) VOR gain = 0: This would indicate a complete lack of vestibular response, which is not expected when testing the healthy side.

Given this individual's vestibular condition, the most likely VOR gain during a head impulse test to the right (healthy) side would be (B) VOR gain = 1, indicating a normal vestibular response on the healthy side.

You

Please answer the following multiple-choice question as a hearing care professional, providing reasoning and explanation: 42 在外耳道給予壓力刺激下引起頭暈的現象，被稱為下列何者？
（A） Tumarkin 危機（Tumarkin crisis） （B）漢尼拔徵候（Hennebert sign）
（C）卡哈氏凹陷（Carhart notch） （D） Tullio 現象（Tullio phenomenon）
43 進行旋轉椅測試時，前庭眼反射增益值[illegible]OR gain）的計算是指下列何者？
（A）相同方向的頭部轉速與眼睛轉速的比值 （B）相反方向的頭部轉速與眼睛轉速的比值
（C）左右眼轉速差異與總合的比值 （D）左右頭部轉速差異與總合的比值

ChatGPT

Question 42:

The phenomenon where dizziness is induced by pressure stimulation in the external auditory canal is known as:

- (A) Tumarkin's crisis: This term refers to sudden falls without loss of consciousness in patients with Ménière's disease, not specifically related to pressure changes in the ear canal.
- (B) Hennebert's sign: This sign is indicative of dizziness or nystagmus induced by pressure changes in the ear canal, often associated with conditions like syphilis or perilymph fistula.
- (C) Carhart's notch: This refers to a specific type of hearing loss dip seen in audiograms, typically associated with otosclerosis, not with dizziness induced by ear canal pressure.
- (D) Tullio phenomenon: This phenomenon involves dizziness or nystagmus induced by loud sounds, not by pressure changes in the ear canal.

The correct answer is (B) Hennebert's sign, which is associated with dizziness or nystagmus triggered by pressure changes in the external auditory canal.

Question 43:

In rotational chair testing, the calculation of Vestibular-Ocular Reflex (VOR) gain refers to:

- (A) The ratio of the head's rotational velocity in the same direction as the eye's rotational velocity. This option does not accurately describe the calculation of VOR gain. VOR typically involves eye movements in the opposite direction of head movements to maintain visual stability.
- (B) The ratio of the head's rotational velocity in the opposite direction as the eye's rotational velocity. VOR gain is calculated as the ratio of eye velocity to head velocity, where eye movements occur in the opposite direction to the head's movement for visual stabilization.
- (C) The difference in rotational velocity between the left and right eyes relative to their combined total. This description does not accurately represent VOR gain calculation.
- (D) The difference in rotational velocity between the left and right sides of the head relative to their combined total. This is not relevant to VOR gain calculation.

The correct answer is (B) The ratio of the h[illegible]s rotational velocity in the opposite direction as the eye's rotational velocity. This reflects the fundamental principle of the vestibulo-ocular reflex, where the eyes move in the opposite direction to the head to maintain a stable gaze during head movements.

You

Please answer the following multiple-choice question as a hearing care professional, providing reasoning and explanation: 44 下列有關跳視（saccade）的敘述，何者錯誤?
（A）正常時平均的潛時值（latency）為 150~200 ms
（B）跳視的速度指的是眼球移動過程中的最大速度值（peak velocity）
（C）過衝（over-shoot）一再反覆出現時，應考慮小腦病變之可能性
（D）出現失準（saccade inaccuracy），即可視為中樞性前庭病變
45 測試者在進行耳溫差測驗（caloric test），會建議其平躺並調整頭部為多少角度，使其水平半規管與水平線成 90 度夾角?
（A）後仰 30 度 （B）上抬 30 度 （C）後仰 60 度 （D）上抬 60 度

ChatGPT

Question 44:

Let's analyze each statement about saccades for accuracy:

- (A) Normal average latency values are 150-200 ms. This is correct. The latency for saccades, which is the time from the stimulus onset to the beginning of the eye movement, typically falls within this range.
- (B) Saccade velocity refers to the maximum speed of the eye during movement. This is also correct. Saccade velocity is often measured in terms of peak velocity during the eye movement.
- (C) Persistent overshoot should raise the possibility of cerebellar lesions. This is accurate. Overshoots in saccades, where the eyes move beyond the target, can be indicative of cerebellar dysfunction.
- (D) The presence of saccade inaccuracy alone is indicative of central vestibular lesions. This statement is overly simplistic and potentially misleading. While saccade inaccuracy can be seen in central vestibular disorders, it can also be present in other conditions. Therefore, saccade inaccuracy on its own is not sufficient to diagnose a central vestibular disorder.

The incorrect statement is (D) The presence of saccade inaccuracy alone is indicative of central vestibular lesions. Saccade inaccuracy ne[illegible]o be interpreted in the broader context of clinical and diagnostic findings.

Question 45:

Regarding the caloric test and the position of the head to align the horizontal semicircular canal with the horizontal plane:

- (A) Tilting the head backward 30 degrees. This is incorrect. A 30-degree backward tilt does not align the horizontal semicircular canal with the horizontal plane.
- (B) Raising the head 30 degrees. This is the correct position. Elevating the head by 30 degrees during the caloric test aligns the horizontal semicircular canal with the horizontal plane, optimizing the test's effectiveness.
- (C) Tilting the head backward 60 degrees. This position would overly tilt the semicircular canals, making them less responsive to the stimuli in a caloric test.
- (D) Raising the head 60 degrees. This position also overly tilts the semicircular canals and is not the standard position for a caloric test.

The correct answer is (B) Raising the head [illegible] degrees. This position aligns the horizontal semicircular canal with the horizontal plane, which is the desired orientation for an effective caloric test.

You

Please answer the following multiple-choice question as a hearing care professional, providing reasoning and explanation: 46 下列有關平衡系統之耳溫差測試（caloric test）之敘述，何者錯誤?
（A）兩耳之慢速相速率（slow phase velocity）相差大於 20%~30%，即認定為單側衰弱（unilateral weakness）
（B） unilateral weakness 之患者，認定可能具有周邊平衡病變（peripheral vestibular dysfunction）
（C） caloric test 是測試 vestibulo-ocular reflex（VOR）之生理
（D） caloric test 為 unilateral weakness 之患者，一定是內耳半規管病變
47 下列關於內耳外淋巴瘻管（perilymphatic fistula）的敘述，何者錯誤?
（A）可能伴隨突發性聽損 （B） 75%臨床患者有耳鳴症狀
（C）姿勢變換過程中，可觀察到眼振 （D）病患必須藉由中耳手術修補瘻管才能痊癒

ChatGPT

Question 46:

Let's analyze each statement about the caloric test of the vestibular system for

You

Please answer the following multiple-choice question as a hearing care professional, providing reasoning and explanation: 46 下列有關平衡系統之耳溫差測試（caloric test）之敘述，何者錯誤?
（A）兩耳之慢速相速率（slow phase velocity）相差大於 20%~30%，即認定為單側衰弱（unilateral weakness）
（B） unilateral weakness 之患者，認定可能具有周邊平衡病變（peripheral vestibular dysfunction）
（C） caloric test 是測試 vestibulo-ocular reflex（VOR）之生理
（D） caloric test 為 unilateral weakness 之患者，一定是內耳半規管病變
47 下列關於內耳外淋巴瘻管（perilymphatic fistula）的敘述，何者錯誤?
（A）可能伴隨突發性聽損 （B） 75%臨床患者有耳鳴症狀
（C）姿勢變換過程中，可觀察到眼振 （D）病患必須藉由中耳手術修補瘻管才能痊癒

ChatGPT

Question 46:

Let's evaluate each statement regarding the caloric test, which is part of the balance system assessment:

- (A) A difference in slow phase velocity (SPV) of more than 20%-30% between the ears is considered indicative of unilateral weakness. This is correct. Such a difference in SPV is a standard criterion for diagnosing unilateral weakness.
- (B) Unilateral weakness in the caloric test suggests the possibility of peripheral vestibular dysfunction. This is also correct. Unilateral weakness typically points to a problem in the peripheral vestibular system of the weaker side.
- (C) The caloric test assesses the physiology of the vestibulo-ocular reflex (VOR). This is accurate. The caloric test stimulates the vestibular system and measures the induced eye movements (VOR) to assess vestibular function.
- (D) Unilateral weakness in the caloric test definitively indicates a disorder of the inner ear semicircular canals. This statement is misleading. While unilateral weakness often points to a problem in the peripheral vestibular syste[illegible] does not definitively diagnose a specific type of disorder like semicircular canal pathology. Other causes and assessments need to be considered.

The incorrect statement is (D) Unilateral weakness in the caloric test definitively indicates a disorder of the inner ear semicircular canals. It is an oversimplification and does not consider other potential causes.

Question 47:

Regarding perilymphatic fistula, let's evaluate the accuracy of each statement:

- (A) May be accompanied by sudden hearing loss. This is correct. A perilymphatic fistula can cause sudden sensorineural hearing loss.
- (B) 75% of clinical patients have tinnitus symptoms. Tinnitus is a common symptom of perilymphatic fistula, although the exact percentage might vary.
- (C) Nystagmus can be observed during postural changes. This is also correct. Changes in pressure within the inner ear due to a fistula can cause nystagmus during certain movements or postural changes.
- (D) Patients must undergo middle ear surgery to repair the fistula for recovery. This statement is not always true. While surgery is a treatment option for perilymphatic fistula, not all cases require surgical intervention. Some cases may resolve spontaneously or be managed with conservative treatments.

The incorrect statement about perilymphatic fistula is (D) Patients must undergo middle ear surgery to repair the fistula for recovery. Surgery is not always necessary for treatment and recovery.

You

Please answer the following multiple-choice question as a hearing care professional, providing reasoning and explanation: 48 Epley 在 1992 年提出的「耳石復位術（canalith repositioning procedure, CRP）」，若患者的患側在右側，則施作的第一步驟為下列何者?
（A）身體向後仰躺，後頸放鬆延伸 （B）頭部轉向右側 45 度向後躺
（C）頭部轉向左側 45 度向後躺 （D）仰躺且頭部上抬與水平成 30 度
49 下列何項因素最不會對前庭障礙患者的前庭代償能力以及前庭復健的結果及預後產生負面影響?
（A）同時患有糖尿病併發周邊神經病變
（B）前庭障礙確診為單側前庭神經炎
（C）前庭障礙確診為單側梅尼爾氏症，會反覆發作，時好時壞
（D）同時患有僵直性脊椎炎

ChatGPT

Question 48:

Epley's Canalith Repositioning Procedure (CRP), developed in 1992, is a series of movements used to treat benign paroxysmal positional vertigo (BPPV), particularly of the posterior semicircular canal. For a patient with the affected side on the right, the first step in the procedure is:

- (A) Lying back with the head extended and neck relaxed. This is a part of the procedure, but not typically the first step.
- (B) Turning the head to the right side at 45 degrees and then lying back. This is the correct first step for a right-sided BPPV. The head is turned to the affected side (right side in this case) at a 45-degree angle and then the patient is brought to a lying down position.
- (C) Turning the head to the left side at 45 degrees and then lying back. This would be the first step if the left side were the affected side, [illegible] the right.
- (D) Lying on the back with the head raised 30 degrees from horizontal. This is not the first step of the Epley maneuver.

The correct answer is (B) Turning the head to the right side at 45 degrees and then lying back.

Question 49:

Factors that might negatively impact vestibular compensation and the outcomes and prognosis of vestibular rehabilitation in patients with vestibular disorders include:

- (A) Concurrent diabetes with peripheral neuropathy. This can negatively impact vestibular compensation due to additional sensory deficits and reduced proprioceptive feedback.
- (B) Diagnosis of unilateral vestibular neuritis. This condition typically responds well to vestibular rehabilitation, and unilateral loss often leads to good compensation.
- (C) Diagnosis of unilateral Ménière's disease with recurrent attacks. The fluctuating nature of Ménière's disease can make compensation more challenging and unpredictable.
- (D) Concurrent ankylosing spondylitis. This could potentially limit the effectiveness of vestibular rehabilitation due to physical limitations a[illegible] pain.

The factor least likely to have a negative impact on vestibular compensation and rehabilitation outcomes is (B) Diagnosis of unilateral vestibular neuritis. This condition often has a favorable prognosis with rehabilitation, as the vestibular system can adapt well to the unilateral loss.

You

Please answer the following multiple-choice question as a hearing care professional, providing reasoning and explanation: 50 下列關於處理急性前庭功能症候群（acute vestibular syndrome）病患的敘述何者錯誤?
（A）一般病患在 2-3 天後即可以開始活動以利早期適應
（B）如果患者一開始活動便嘔吐不已，仍應繼續活動
（C）即便在發病初期，前庭系統就已開始產生適應現象
（D）應儘早鼓勵患者轉動頭部及利用視覺來適應

ChatGPT

Question 50:

Let's assess each statement about manag[illegible] patients with acute vestibular syndrome for accuracy:

- (A) Patients can generally start activities after 2-3 days to facilitate early adaptation. This is correct. Early mobilization and activity are encouraged in acute vestibular syndrome to promote vestibular compensation.
- (B) If patients start to vomit when they begin activities, they should still continue activities. This statement is problematic. While activity is encouraged, if a patient is experiencing severe symptoms like uncontrollable vomiting, it may be necessary to modify or temporarily reduce activity levels for patient safety and comfort.
- (C) Even in the early stages of the illness, the vestibular system begins to adapt. This is correct. The process of vestibular compensation can start soon after the onset of symptoms.
- (D) Patients should be encouraged to move their heads and use their vision to adapt as soon as possible. This is also correct. Head movements and visual cues are important for facilitating vestibular compensation and adaptation.

The incorrect statement regarding the management of acute vestibular syndrome is (B) If patients start to vomit when they begin activities, they should still continue activities. It's important to balance the encouragement of activity with the patient's tolerance and comfort, especially if they are experiencing severe symptoms like vomiting.

Message ChatGPT...

ChatGPT can make mistakes. Consider checking important information.
